# A strategy of microglia replacement alleviates microgliopathy in a *CSF1R* I794T hotspot mutation mouse model of CSF1R-related disorder

**DOI:** 10.1016/j.xcrm.2026.102641

**Published:** 2026-02-27

**Authors:** Xin Li, Banglian Hu, Chujun Wu, Ziwei Wang, Hanzheng Fan, Xiaoyan Guan, Sulan Xie, Dadian Chen, Xiaohua Huang, Hao Sun, Yanfang Li, Xian Zhang, Guojun Bu, Zhanxiang Wang, Yun-Wu Zhang, Li Zhong, Zaiqiang Zhang, Honghua Zheng

**Affiliations:** 1 Xiamen Key Laboratory of Brain Center, The First Affiliated Hospital of Xiamen University, Fujian Key Laboratory of Neurodegenerative Disease and Aging Research, Institute of Neuroscience, School of Medicine, Xiamen University, Xiamen, Fujian 361102, China; 2 Department of Neurology, China National Clinical Research Center for Neurological Diseases, Beijing Tiantan Hospital, Capital Medical University, Beijing 100070, China; 3 Basic Medical Sciences, School of Medicine, Xiamen University, Xiamen, Fujian 361102, China; 4 Division of Life Science and State Key Laboratory of Nervous System Disorders, The Hong Kong University of Science and Technology, Clear Water Bay, Hong Kong, China; 5 Department of Neurosurgery, Xiamen Key Laboratory of Brain Center, The First Affiliated Hospital of Xiamen University, Xiamen, Fujian 361003, China

**Keywords:** CSF1R, CSF1R-RD, leukoencephalopathy, microglia, microglia replacement

## Abstract

The I794T hotspot mutation in the colony-stimulating factor 1 receptor (CSF1R) gene is associated with primary microgliopathy manifesting as leukoencephalopathy. In this study, we identify three Chinese probands harboring the CSF1R p.I794T variant and characterize their clinical and neuroimaging profiles. To elucidate disease mechanisms and explore therapeutic avenues, we generate a *Csf1r*^I792T/+^ knockin mouse model that carries this human mutation. These *Csf1r*^I792T/+^ mice exhibit hallmark features of CSF1R-related disorder (CSF1R-RD), including cognitive deficits, ventricular enlargement, reduced microglia, axonal spheroids, and demyelination. Transcriptomic analysis reveals that *Csf1r*^I792T/+^ microglia adopt an activated and disease-associated microglia (DAM)-like phenotype. Crucially, we develop and test a microglia replacement strategy, termed “duplicate-cyclic microglial depletion for transplantation” (DCMDT), which significantly ameliorates neuropathological deficits in *Csf1r*^I792T/+^ mice. Our findings highlight the pathological significance of the CSF1R p.I794T mutation and propose DCMDT as a promising therapeutic approach for neurodegenerative disorders driven by microglial dysfunction.

## Highlights

• CSF1R p.I794T is a global hotspot mutation underlying CSF1R-RD
• *Csf1r*^I792T/+^ mice recapitulate key clinical and pathological features of CSF1R-RD
• *Csf1r*^I792T/+^ microglia exhibit an activated and DAM-like phenotype
• A microglial replacement strategy attenuates the deficits in *Csf1r*^I792T/+^ mice

## Graphical abstract

**Figure:**
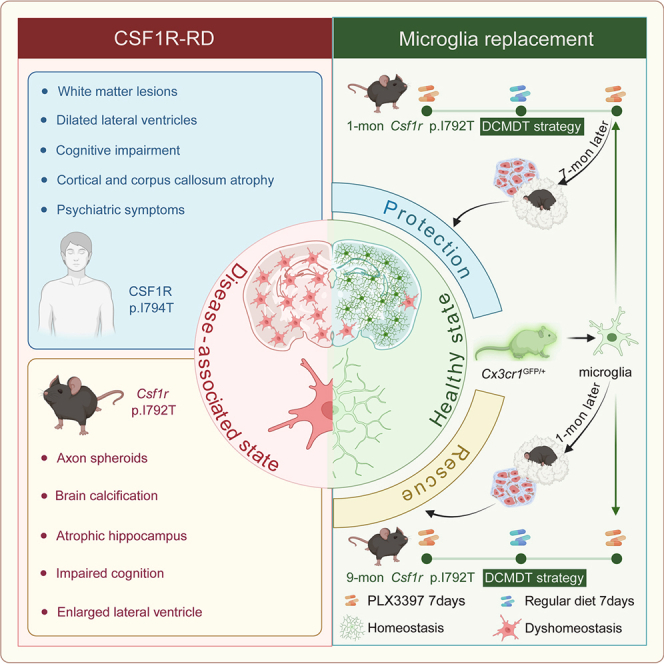

## Teaser

Li et al. define the clinical features of patients carrying the CSF1R p.I794T variant and establish a corresponding knockin mouse model. They show that *Csf1r*^I792T/+^ microglia adopt a disease-associated state and that a microglial replacement strategy, DCMDT, mitigates cognitive and neuropathological deficits in CSF1R-related disorder.

## Introduction

The colony-stimulating factor 1 receptor (CSF1R) is a tyrosine kinase receptor predominantly expressed on microglia within the central nervous system (CNS). Autosomal dominant mutations in *CSF1R* cause a progressive leukodystrophy known as adult-onset leukoencephalopathy with axonal spheroids and pigmented glia (ALSP),^1^^,^^2^^,^^3^^,^^4^ characterized by dementia with motor impairments and neuropsychiatric deficits. The disease has recently been reclassified under the broader term “CSF1R-related disorder” (CSF1R-RD).^5^

To date, more than 140 mutations in CSF1R have been implicated in CSF1R-RD, although the true prevalence may be underestimated due to insufficient genetic screening or incomplete penetrance.^6^ One recurrent global hotspot mutation, c.2381T > C (p.I794T), is frequently associated with this disorder.^7^ CSF1R plays a critical role in microglial survival, development, and homeostasis.^8^ Mouse models with haploinsufficiency for *Csf1r* (*Csf1r*^+/−^) mimic key neuropathological and behavioral features of ALSP,^9^ including progressive microglial dysfunction, which has been recognized as a central driver of disease.^10^

Various therapeutic strategies have been explored in preclinical studies. Pharmacological interventions, such as glucocorticoid treatment, the CSF1R inhibitor PLX5622, and the microglial suppressor minocycline, have shown promise in mitigating the pathological features of *Csf1r*^+/−^ mice.^11^^,^^12^^,^^13^^,^^14^ Hematopoietic stem cell transplantation (HSCT) has also been applied in CSF1R-RD patients, with mixed outcomes, ranging from disease stabilization to continued progression,^15^^,^^16^ highlighting the need to identify predictors of therapeutic response. More recently, microglial replacement has emerged as a potential therapeutic strategy. In particular, studies using *Csf1r*^ΔFIRE/ΔFIRE^ mice, which model many aspects of human CSF1R-RD, demonstrated that replacing microglia can reverse key pathological features of CSF1R-RD.^17^^,^^18^ These findings provide a rationale for further development of microglial replacement approaches to treat primary microgliopathies such as CSF1R-RD.

In this study, we identified three unrelated Chinese probands carrying the heterozygous CSF1R p.I794T variant and summarized the clinical and neuroimaging characteristics of this globally relevant mutation. To model the human disease, we generated *Csf1r* p.I792T knockin mice using homologous recombination. These mice exhibited microglial reduction and functional alterations, including increased proinflammatory signaling and phagocytosis, paralleling key human pathological features. Most importantly, we developed a strategy for therapeutic microglia replacement, termed “duplicate-cyclic microglial depletion for transplantation” (DCMDT). This approach substantially attenuated both behavioral deficits and neuropathologies in the *Csf1r*^I792T/+^ model. Our findings confirm that the CSF1R p.I794T heterozygous mutation is sufficient to drive the pathological and clinical hallmarks of CSF1R-RD. Furthermore, we establish DCMDT as a viable and efficient therapeutic strategy for mitigating microglial dysfunction in CSF1R-RD and potentially other neurodegenerative diseases with a similar etiology.

## Results

### CSF1R p.I794T is a global hotspot mutation underlying CSF1R-RD

**Figure 1:**
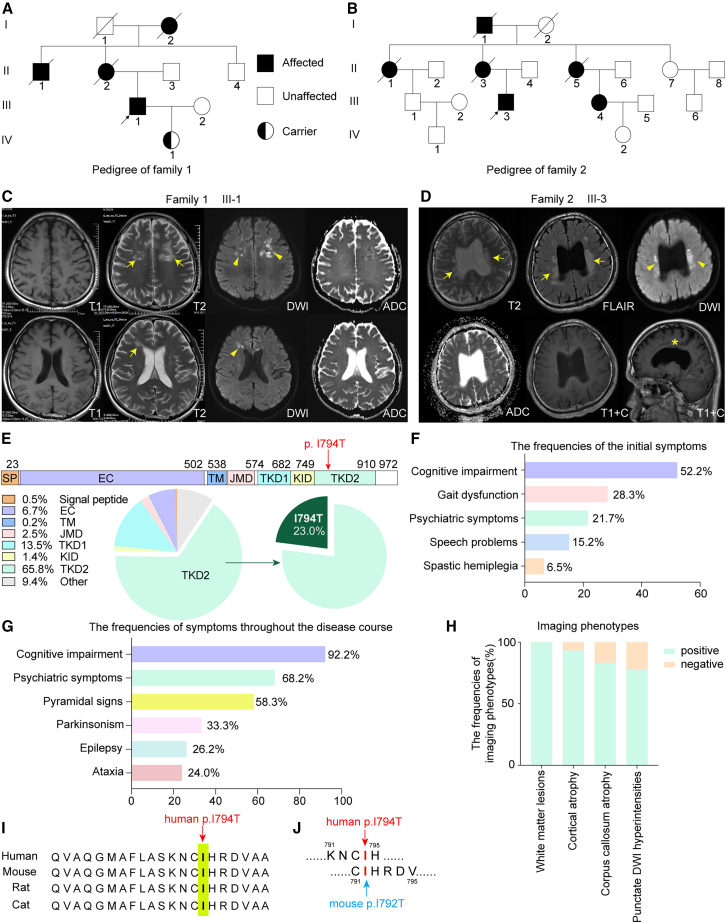
Clinical and imaging features of patients carrying the CSF1R p.I794T mutation (A and B) Pedigrees of two families affected by CSF1R-RD carrying the CSF1R p.I794T mutation. Affected individuals are denoted by black-filled symbols. The probands are indicated with black arrows (III-1 in Family 1; III-3 in Family 2). Carriers of the CSF1R p.I794T mutation are represented by half-filled symbols. White-filled symbols denote unaffected individuals, while diagonal slashes indicate deceased individuals. Squares and circles represent males and females, respectively. (C) Brain MRI of proband III-1 (Family 1) revealed periventricular WMLs in the frontal lobes (arrows), lateral ventricular enlargement, and cortical atrophy predominantly affecting the frontal and parietal lobes. DWI showed characteristic punctate hyperintensities (arrowheads) within the WMLs. (D) MRI of proband III-3 (Family 2) showed confluent and patchy periventricular and deep WMLs (arrows), along with enlarged lateral ventricles and corpus callosum atrophy (asterisk). Punctate DWI hyperintensities were also observed (arrowheads). (E) To date, 222 CSF1R mutations have been identified worldwide, with CSF1R p.I794T being a mutational hotspot (66/436 probands). Most mutations cluster within the intracellular tyrosine kinase domain 2 (TKD2) of CSF1R. (F) Frequencies of initial clinical symptoms in patients with the CSF1R p.I794T mutation. (G) Frequencies of symptoms throughout the disease course in affected individuals with the CSF1R p.I794T mutation. (H) Frequencies of characteristic imaging features among patients with the CSF1R p.I794T mutation. (I) The p.I794T residue in CSF1R is evolutionarily conserved across vertebrate species. (J) The human *CSF1R* p.I794T variant corresponds to *Csf1r* p.I792T in mice. EC, extracellular domain; TM, transmembrane domain; JMD, juxtamembrane domain; TKD1/2, tyrosine kinase domain 1/2.

We identified three Chinese probands from unrelated families, each harboring a heterozygous *CSF1R* p.I794T variant confirmed by genetic testing (Figures S1A–S1D). One proband had no known family history, while the other two belonged to pedigrees with clinical and radiological features characteristic of CSF1R-RD (Figures 1A and 1B), including magnetic resonance imaging (MRI) findings of leukoencephalopathy (Figures 1C and 1D).

The first proband (F1-III1) presented with memory impairment at age 35, followed by dysphasia 1 year later. Neurological examination revealed brisk tendon reflexes, and his Montreal Cognitive Assessment score was 24. Brain MRI demonstrated periventricular white matter lesions (WMLs) in the frontal lobes, dilated lateral ventricles, and a thinned corpus callosum. Punctate diffusion-weighted imaging (DWI) hyperintensities were evident within WMLs (Figure 1C). His condition progressively deteriorated, culminating in dysphagia, tetraparesis, and cachexia-related death at age 39. A strong family history was noted: his mother (F1-II2), maternal uncle (F1-II1), and maternal grandmother (F1-I2) all exhibited motor dysfunction, late-onset cognitive decline, and dysphasia in their 50s. The proband’s daughter, now aged 14, also carries the CSF1R p.I794T variant but remains asymptomatic.

The second proband (F2-III3) developed right lower limb weakness at age 27, progressing to right-hand weakness within a year. Physical examination revealed hypertonia and brisk reflexes in all limbs. MRI revealed periventricular WMLs, thinning of the corpus callosum, dilated ventricles, and punctate DWI hyperintensities (Figure 1D). Computed tomography (CT) imaging did not show white matter calcification. The proband’s maternal grandfather (F2-I1) developed ataxia at age 66. His mother (F2-II3) experienced cognitive impairment, and two maternal aunts (F2-II1, F2-II5) presented with ataxia in their 40s. The proband’s cousin (F2-III4) is asymptomatic but showed ventricular dilation on MRI.

The third proband initially presented with psychiatric symptoms—anxiety, paranoia, and irritability at age 39. Over the next 3 years, he developed dysphasia, dysphagia, and cognitive decline. His mother had a history of epilepsy. Upon clinical assessment, his Mini-Mental State Examination score was 3, with signs of aphasia and bilateral pyramidal tract involvement. MRI showed dilated lateral ventricles, frontoparietal WMLs, and punctate DWI hyperintensities.

Since CSF1R mutations were first reported by Rademakers et al. in 2011 in 14 families,^19^ the number of identified cases has steadily increased across diverse populations.^6^^,^^20^^,^^21^ To date, over 222 distinct CSF1R mutations have been reported in 436 probands globally. Most mutations (∼65.8%) are located in the intracellular tyrosine kinase domain 2 (TKD2) of CSF1R. Notably, the p.I794T missense variant alone accounts for 23.0% of TKD2 mutations, making it the most frequent single variant (Figure 1E). We comprehensively reviewed the clinical and imaging characteristics of 66 probands with heterozygous CSF1R p.I794T mutations, including 63 previously reported cases and the three described in the present study (Figures 1F–1H). Clinical and radiological details for 66 of these probands are summarized in Tables S1 and S2. A majority (77.3%, 51/66) originated from East Asia. Among 58 probands with available demographic data, the clinically recorded gender distribution was equal (1:1). The mean age at symptom onset was 40.8 ± 9.9 years (mean ± SD; range, 20–60 years), with male patients presenting at a mean age of 43.9 ± 8.8 years (range, 27–60 years) and female patients at 37.8 ± 10.3 years (range, 20–60 years). Disease progression was rapid, with an average clinical duration of 3.3 ± 1.0 years (range, 2–5 years; 3.0 ± 1.0 years in males, range, 2–4 years; 3.7 ± 1.2 years in females, range, 3–5 years), resulting in death at a mean age of 42.7 ± 4.1 years (range, 39–49 years; 42.3 ± 5.8 years in males, range, 39–49 years; 43.0 ± 3.0 years in females, range, 40–46 years). No significant difference was observed in the age of onset (40.8 years vs. 41.9 years, *p* = 0.270) between patients harboring the p.I794T mutation (Tables S1 and S2) and those with other mutations (Table S3). Initial neurological symptoms were available for 46 individuals: cognitive impairment (52.2%) was the most common, followed by gait dysfunction (28.3%), psychiatric symptoms (21.7%), speech problems (15.2%), and spastic hemiplegia (6.5%) (Figure 1F). Over the disease course, cognitive impairment was nearly universal (92.2%), accompanied by psychiatric symptoms (68.2%), pyramidal signs (58.3%), parkinsonism (33.3%), epilepsy (26.2%), and ataxia (24.0%) (Figure 1G).

Neuroimaging data revealed that all patients exhibited WMLs, with 93.3% also showing cortical atrophy, preferentially affecting the frontal and parietal lobes (Figure 1H). Corpus callosum atrophy and punctate DWI hyperintensities were present in 83.3% and 78.3% of patients, respectively (Figure 1H). Among 20 patients who underwent CT scans, white matter calcifications were detected in 11. The p.I794 residue in CSF1R is highly conserved across vertebrate species (Figure 1I). In mice, the corresponding mutation is *Csf1r* p.I792T (Figure 1J), enabling the development of a relevant murine model for mechanistic studies and therapeutic intervention in CSF1R-RD.

### The *Csf1r*^I792T/+^ mouse model recapitulates clinical and pathological features of CSF1R-RD

**Figure 2:**
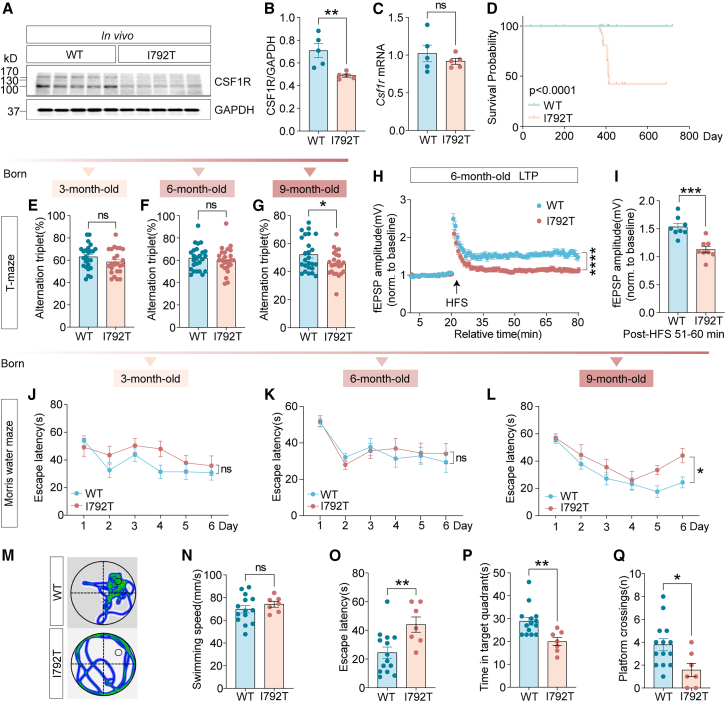
Impaired cognitive performance in 9-month-old *Csf1r*^I792T/+^ mice The *Csf1r*^I792T/+^ (I792T) mouse model was generated via homologous recombination, introducing the I792T point mutation into the murine *Csf1r* gene. (A–C) CSF1R expression in the cortex of 9-month-old *Csf1r*^I792T/+^ mice was analyzed via western blot (A and B) and qPCR (C), *n* = 5 per group. Unpaired two-tailed Student’s *t* test. (D) *Csf1r*^I792T/+^ mice exhibited significantly reduced survival compared to wild-type controls (*n* = 50 per group). (E–G) Working memory performance at 3, 6, and 9 months was assessed using the T-maze spontaneous alternation task. *Csf1r*^I792T/+^ mice (*n* = 24) demonstrated progressive deficits in spontaneous alternation relative to control littermates (*n* = 25). (H) LTP was evaluated in hippocampal CA1 region from 6-month-old mice. Time-series plots show fEPSPs following HFS. (I) Quantification of fEPSP amplitude averaged over the final 10 min of LTP recording. WT (*n* = 5 mice, 10 slices), *Csf1r*^I792T/+^ (*n* = 5 mice, 8 slices). (J–L) Spatial learning and memory were assessed using the MWM at 3, 6, and 9 months. Escape latency over a 6-day training period revealed impaired learning in 9-month-old *Csf1r*^I792T/+^ mice. (M) Representative swim paths from 9-month-old mice show impaired spatial navigation in *Csf1r*^I792T/+^ mice. (N–Q) Quantitative metrics from MWM testing: swimming speed (N), escape latency (O), time spent in the target quadrant (P), and platform crossings (Q) showed significant deficits in 9-month-old *Csf1r*^I792T/+^ mice compared to controls. WT, *n* = 14; *Csf1r*^I792T/+^, *n* = 7. Unpaired two-tailed Student’s *t* test. Data are expressed as mean ± SEM. ∗*p* < 0.05, ∗∗*p* < 0.01, ∗∗∗*p* < 0.001, ∗∗∗∗*p* < 0.0001; ns, not significant. I792T, *Csf1r*^I792T/+^.

Studies have previously reported that single-allele heterozygous mutations in CSF1R have been clinically linked to CSF1R-RD.^7^^,^^19^^,^^20^^,^^21^ To investigate the pathogenic mechanisms and explore potential therapeutic strategies, we generated a knockin mouse model harboring the *Csf1r* p.I792T variant via homologous recombination-based gene editing (Figures S2A–S2C). We first assessed CSF1R expression in these mice. While CSF1R transcript levels remained unchanged, protein levels were markedly reduced in the brains of *Csf1r*^I792T/+^ mice relative to control littermates (Figures 2A–2C). This reduction was also observed in primary microglia isolated from neonatal *Csf1r*^I792T/+^ mice (Figures S2D–S2F). Homozygous *Csf1r*^I792T/I792T^ mice displayed perinatal lethality similar to *Csf1r*^−/−^ mice, with most failing to survive beyond 24 h.^8^ Moreover, heterozygous *Csf1r*^I792T/+^ mice exhibited significantly reduced overall survival compared to wild-type (WT) littermates (Figure 2D).

Given that cognitive decline is often the initial clinical symptom in CSF1R-RD patients with the p.I794T variant, we next examined whether *Csf1r*^I792T/+^ mice exhibit analogous cognitive impairments. In spontaneous alternation T-maze tests, 9-month-old *Csf1r*^I792T/+^ mice but not 3- or 6-month-old *Csf1r*^I792T/+^ mice showed significant working memory deficits compared with age-matched *Csf1r*^+/+^ littermates (Figures 2E–2G). To assess hippocampal synaptic function, long-term potentiation (LTP) was induced by high-frequency stimulation (HFS) in CA1 regions of 6-month-old *Csf1r*^I792T/+^ and *Csf1r*^+/+^ mice. The amplitude of field excitatory postsynaptic potential (fEPSP) was substantially reduced in *Csf1r*^I792T/+^ mice (Figures 2E–2G). Spatial learning and memory were further evaluated using the Morris water maze (MWM). At 3 and 6 months, escape latency did not differ between genotypes (Figures 2J and 2K). However, 9-month-old *Csf1r*^I792T/+^ mice showed significantly longer escape latencies during the 6-day training period (Figure 2L). On test day (day 7), swim speeds were comparable (Figures 2M and 2N), yet *Csf1r*^I792T/+^ mice still demonstrated prolonged escape latency (Figure 2O), spent less time in the target quadrant (Figure 2P), and had fewer platform crossings (Figure 2Q). Emotional performance was also evaluated using the open field test (Figures S2G–S2J) and the elevated plus maze test (Figures S2K–S2N), revealing no anxiety-like behaviors at 3, 6, 9, or 12 months. Together, these findings demonstrate that the *Csf1r* p.I792T variant leads to age-dependent cognitive impairments.

**Figure 3:**
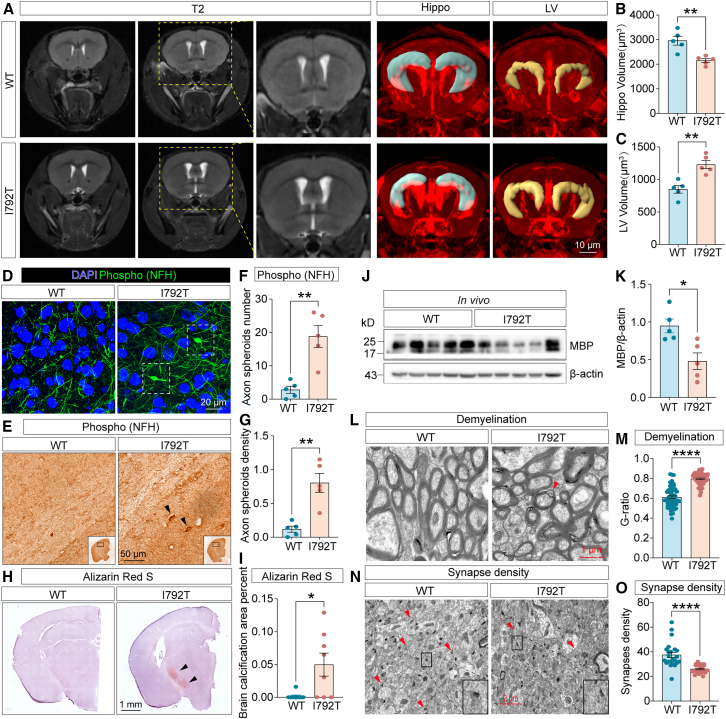
The 9-month-old *Csf1r*^I792T/+^ mouse model recapitulates key pathological and imaging features of CSF1R-RD (A) T2-weighted MRI of 9-month-old I792T mice showed enlarged LVs. Three-dimensional reconstructions highlight the LV (yellow) and hippocampus (blue). Scale bar, 10 μm. (B) Decreased hippocampal volumes examined in 9-month-old *Csf1r*^I792T/+^ mouse brains, *n* = 5 mice per group. (C) Increased LV volumes observed in 9-month-old *Csf1r*^I792T/+^ mice, *n* = 5 mice per group. (D) Axonal spheroids in 9-month-old *Csf1r*^I792T/+^ mouse brains were observed using immunofluorescent labeling of phospho-NFH (green, white squares). Scale bar, 20 μm. (E) Axonal spheroids in 9-month-old *Csf1r*^I792T/+^ mouse brains were observed using immunohistochemical labeling of phospho-NFH (dark arrowheads). Scale bar, 50 μm. (F and G) Quantification of the number (F) and density (G) of axonal spheroids, *n* = 3 mice per group. (H) Alizarin red S staining revealed cerebral calcification in 9-month-old *Csf1r*^I792T/+^ mice (black arrowheads). Scale bar, 1 mm. (I) Quantification of cerebral calcifications, *n* = 3 mice per group. (J) Representative western blots of MBP expression in 9-month-old *Csf1r*^+/+^ or *Csf1r*^I792T/+^ mouse brains. (K) MBP protein assessed using densitometry relative to β-actin, *n* = 5 mice per group. (L) Transmission electron microscopy demonstrated demyelination in 9-month-old *Csf1r*^I792T/+^ mice. Scale bar, 1 μm. (M) The G-ratio (ratio of inner to outer myelin sheath diameter) was significantly increased in 9-month-old *Csf1r*^I792T/+^ mice, *n* = 4 mice per group. (N) Ultrastructural analysis of the synapse in 9-month-old *Csf1r*^I792T/+^ mice by electron microscopy. Scale bar, 2 μm. (O) The synaptic density was reduced in 9-month-old *Csf1r*^I792T/+^ mouse brains, *n* = 4 mice per group. Data are presented as mean ± SEM. Unpaired two-tailed Student’s *t* test. ∗*p* < 0.05, ∗∗*p* < 0.01, ∗∗∗∗*p* < 0.0001. WT, wild-type; I792T, *Csf1r*^I792T/+^.

MRI T2 imaging revealed hippocampal atrophy and lateral ventricle (LV) enlargement in 9-month-old *Csf1r*^I792T/+^ mice (Figures 3A–3C). Immunohistochemical and immunofluorescent staining for phosphorylated neurofilament heavy chain (phospho-NFH), a marker of axonal damage,^22^ revealed axonal spheroids in the brains of these mice (Figures 3D–3G). Alizarin red S staining also detected brain calcifications at 9 months (Figures 3H and 3I). Furthermore, the G-ratio of myelin sheaths was significantly increased, indicating demyelination, a result corroborated by western blot analysis of myelin basic protein (MBP) expression (Figures 3J–3M). Electron microscopy revealed decreased synaptic density in the brains of *Csf1r*^I792T/+^ mice (Figures 3N and 3O). Collectively, these results demonstrate that the heterozygous 9-month-old *Csf1r* p.I792T variant is sufficient to recapitulate the core clinical and pathological features of CSF1R-RD.

### The *Csf1r* p.I792T variant reduces microglial numbers and promotes an activated and disease-associated microglia-like phenotype

**Figure 4:**
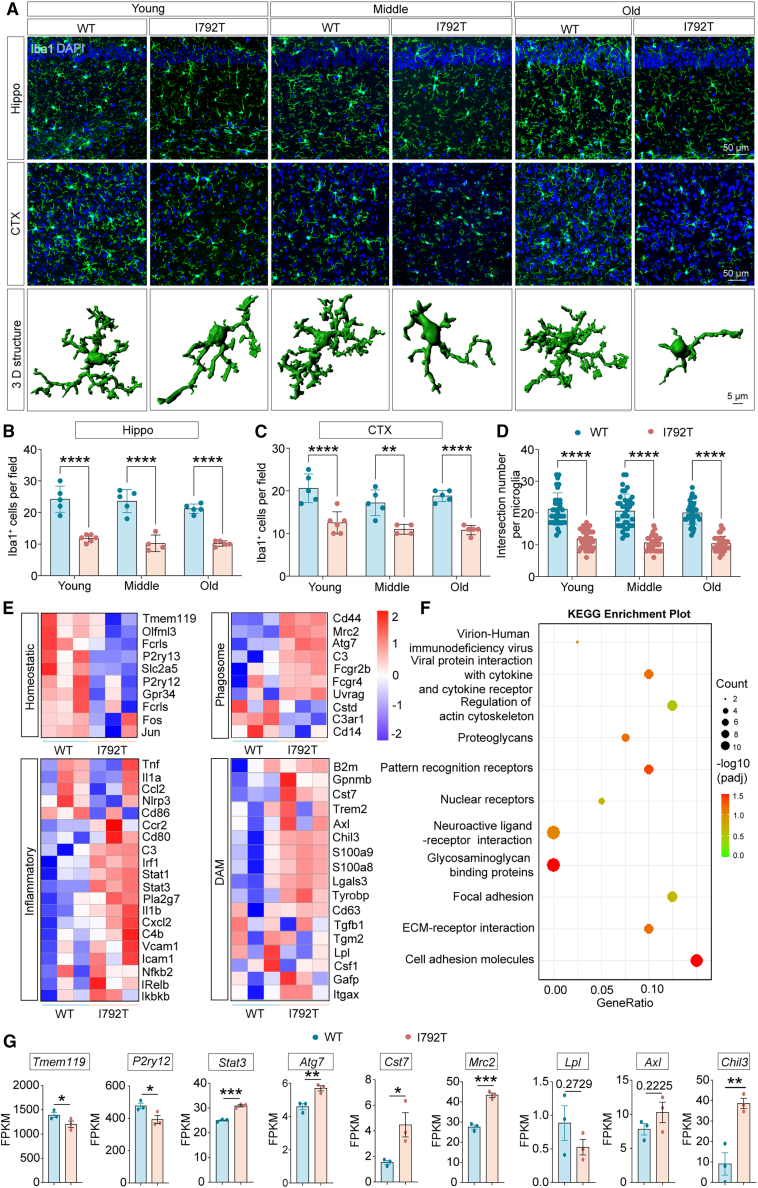
Adult *Csf1r*^I792T/+^ microglia are reduced and show an activated and DAM-like phenotype (A) Representative Iba1 (green) immunofluorescent images and corresponding 3D reconstructions of microglia in the hippocampus and cortex of *Csf1r*^+/+^(WT) and *Csf1r*^I792T/+^ (I792T) mice at young (3–5 months), middle (10–14 months), and old (20–24 months) stages. Microglia in *Csf1r*^I792T/+^ brains exhibit dystrophic morphology, characterized by enlarged, rounded soma and reduced branching. Scale bars: 50 and 5 μm. (B and C) Quantification revealed a significant reduction in Iba1^+^ microglia in both the hippocampus (B) and cortex (C) of *Csf1r*^I792T/+^ mice. (D) Sholl analysis demonstrated markedly decreased process complexity in *Csf1r*^I792T/+^ microglia compared to WT. Branch intersections were quantified at 5-μm intervals from the soma. (E) Heatmap of DEGs from bulk RNA-seq of microglia isolated from 9-month-old *Csf1r*^*+/+*^ or *Csf1r*^I792T/+^ mice. (F) KEGG pathway enrichment analysis of DEGs revealed the 11 significantly altered pathways associated with activated microglia. (G) Representative expression of homeostatic, inflammatory, phagosome, and DAM genes from bulk RNA-seq of microglia isolated from 9-month-old *Csf1r*^+/+^ or *Csf1r*^I792T/+^ mice. FPKM, fragments per kilobase of transcript per million mapped fragments. Data are presented as mean ± SEM. *n* = 4∼6 mice per group. Unpaired two-tailed Student’s *t* test. ∗*p* < 0.05, ∗∗*p* < 0.01, ∗∗∗*p* < 0.001, ∗∗∗∗*p* < 0.0001. WT, wild-type; I792T, *Csf1r*^I792T/+^.

Given previous findings of microgliosis in *Csf1r*^+/−^ mice^9^ and decreased microglial density in *Csf1r*^E631K/+^ mice,^23^ we examined whether the *Csf1r* p.I792T variant affects microglial populations. Quantification of Iba1-positive microglia (Iba1^+^) revealed significantly reduced microglial density in the hippocampus and cortex of *Csf1r*^I792T/+^ mice relative to WT controls (Figures 4A–4C). Morphological analysis showed marked reductions in microglial process length and branching in both the cortex and hippocampus of *Csf1r*^I792T/+^ mice (Figure 4D), indicating a shift toward a dystrophic and dysfunctional phenotype.

As CSF1R-RD is now classified as a primary CNS microgliopathy, we sought to further characterize the molecular consequences of the *Csf1r* p.I792T variant on microglial function. Whole-transcriptome RNA sequencing (RNA-seq) was performed on microglia isolated from postnatal day 0–3 *Csf1r*^+/+^ and *Csf1r*^I792T/+^ mice. Differentially expressed genes (DEGs) were identified and hierarchically clustered based on established analytical frameworks.^24^^,^^25^^,^^26^ Heatmap visualization revealed that DEGs were enriched in pathways related to the cell cycle, phagosome formation, inflammation, and cellular homeostasis (Figure S3A). Kyoto Encyclopedia of Genes and Genomes (KEGG) pathway analysis of gene sets from 0- to 3-day-old *Csf1r*^+/+^ and *Csf1r*^I792T/+^ microglia identified significant enrichment of genes involved in phagosome activity, complement and coagulation cascades, chemokine and Toll-like receptor signaling, antigen presentation, cytokine-cytokine receptor interaction, and tumor necrosis factor signaling (Figure S3B). Gene set enrichment analysis (GSEA) further demonstrated downregulation of cell cycle-associated genes and upregulation of inflammatory and microglial activation signatures in 0- to 3-day-old *Csf1r*^I792T/+^ microglia (Figures S3C–S3E).

Given that microglia from 0- to 3-day-old mice might not reflect their states in adult mice and to better reveal how microglia dysregulation in adult mice accounted for progressive leukoencephalopathy, we isolated microglia from 9-month-old WT or *Csf1r*^I792T/+^ mice by fluorescence-activated cell sorting (FACS) and subjected them to bulk RNA-seq. Heatmap visualization revealed that DEGs in 9-month-old WT or *Csf1r*^I792T/+^ microglia were enriched in pathways related to the disease-associated microglia (DAM), phagosome, inflammation, and cellular homeostasis (Figure 4E). KEGG pathway analysis of gene sets from those *Csf1r*^+/+^ and *Csf1r*^I792T/+^ microglia identified significant enrichment of genes involved in pattern recognition receptors, cytokine-cytokine receptor interaction, and cell adhesion molecules (Figure 4F). Furthermore, the expression of representative inflammatory, remodeling, phagosome, or DAM genes, including *Stat3*, *Mrc2*, *Chil3*, *Atg7*, and *Cst7* levels, were increased, while the homeostatic genes *Tmem119* and *P2ry12* were significantly reduced in 9-month-old *Csf1r*^I792T/+^ microglia relative to WT controls (Figure 4G), suggesting an activated and DAM-like phenotype in microglia from 9-month-old *Csf1r*^I792T/+^ mice relative to WT controls.

Given that elevated early expression of *Csf2* and *Csf3* has been reported in 7-week-old *Csf1r*^+/−^ mice and in CSF1R-RD patients,^27^^,^^28^ we sought to determine whether a similar dysregulation occurs in 9-month-old *Csf1r*^I792T/+^ mice. Unexpectedly, *Csf2* and *Csf3* levels were significantly reduced or exhibited a downward trend in 9-month-old *Csf1r*^I792T/+^ mouse brains compared to WT controls (Figure S4). Furthermore, given that DAM are a hallmark of neurodegeneration,^29^^,^^30^^,^^31^ we assessed the expression of DAM-related genes, including *P2ry12*, *Tmem119*, *Axl*, *H2-Ab1*, *Lpl*, and *Cst7* in 9-month-old *Csf1r*^I792T/+^ mouse brains by quantitative reverse-transcription PCR. While *P2ry12*, *Axl*, *Lpl*, and *Cst7* levels remained unchanged, *Tmem119* and *H2-Ab1* expression was significantly reduced in 9-month-old *Csf1r*^I792T/+^ mouse brains relative to WT controls (Figure S4), further indicating an activated and DAM-like phenotype of microglia in *Csf1r*^I792T/+^ mouse brain.

Collectively, these results indicate that the *Csf1r* p.I792T variant reduces microglial number and induces a shift toward an activated, DAM-like phenotype. This dysfunctional microglial state likely disrupts CNS homeostasis, contributing to the structural and functional brain abnormalities characteristic of progressive leukoencephalopathy.

### Establishment of the DCMDT strategy for brain-wide microglial transplantation in *Csf1r*^I792T/+^ mice

Given the reduction and dysfunction of microglia in *Csf1r*^I792T/+^ mice, we next investigated whether microglial replacement could reverse the behavioral and pathological deficits associated with this CSF1R-RD model. Building on our recent development of an efficient microglial depletion and transplantation method that restored pathological phenotypes in an Alzheimer’s disease mouse model,^32^ we optimized transplantation conditions for *Csf1r*^I792T/+^ mice. One-month-old *Csf1r*^I792T/+^ mice were administered PLX3397 (600 mg/kg, orally) for 3, 5, or 7 days to deplete resident microglia,^33^ followed by a 1-week recovery period on a regular diet to allow for microglial repopulation (Figure S5A). PLX3397 treatment resulted in near-complete microglial depletion at all time groups (Figures S5B–5D). However, the repopulation capacity of endogenous microglia in *Csf1r*^I792T/+^ mice was significantly impaired compared to WT (*Csf1r*^+/+^) controls, particularly in the cortex and in the 7-day treatment group (Figure S5C).

**Figure 5:**
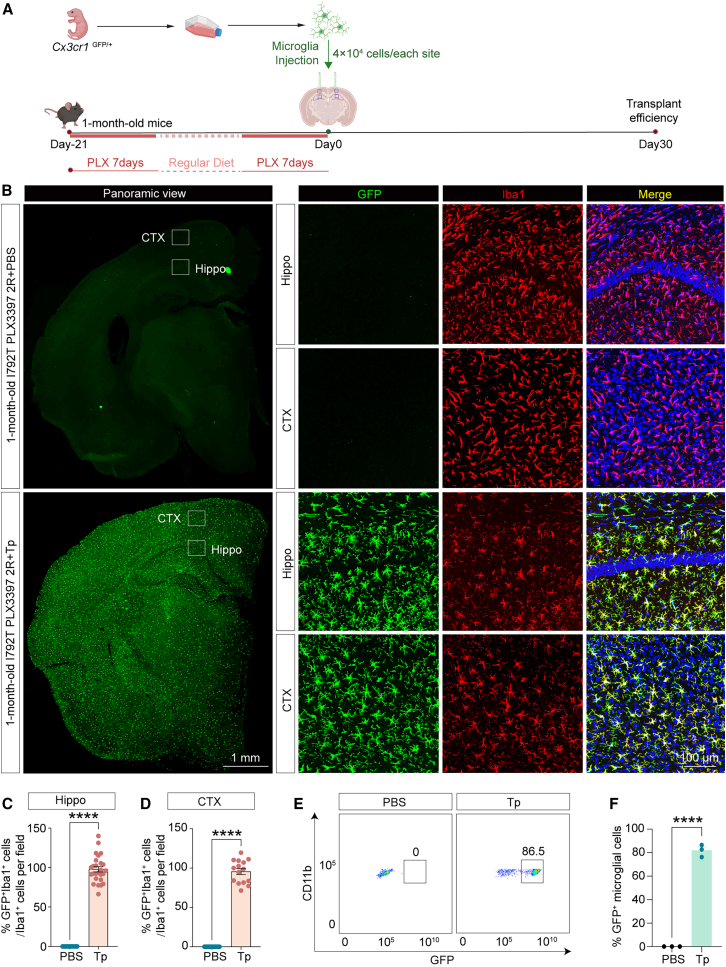
Efficient replacement of endogenous microglia by transplantation in 1-month-old *Csf1r*^I792T/+^ mice (A) Schematic of the microglia depletion and transplantation protocol. Endogenous microglia in 1-month-old *Csf1r*^I792T/+^ mice were ablated via two 7-day cycles of oral PLX3397 administration. (B) Immunofluorescent staining demonstrated robust engraftment of GFP^+^Iba1^+^ donor microglia from *Cx3cr1*^GFP/+^ mice across nearly the entire brain of *Csf1r*^I792T/+^ recipients. Scale bars: 1 mm and 100 μm. (C) Quantification of the percentage of GFP^+^Iba1^+^ microglia relative to Iba1^+^ cells per high field in the hippocampus of *Csf1r*^I792T/+^ mouse brains. (D) Quantification of the percentage of GFP^+^Iba1^+^ microglia relative to Iba1^+^ cells per high field in the cortex of *Csf1r*^I792T/+^ mouse brains. (E) Representative flow cytometry dot plots showing GFP^+^CD11b^+^ donor-derived microglia in transplanted *Csf1r*^I792T/+^ brains. (F) Transplanted microglia accounted for up to 86.5% of total CD11b^+^ microglia. Data are presented as mean ± SEM, *n* = 3 mice per group. ∗∗∗∗*p* < 0.0001. Tp, *Csf1r*^I792T/+^ mice transplanted with microglia.

To assess engraftment efficiency, we transplanted GFP^+^Iba1^+^ microglia derived from *Cx3cr1*^GFP/+^ mice into 1-month-old *Csf1r*^I792T/+^ brains immediately following 7 days of PLX3397 treatment (Figure S6A). One month post-transplantation, only 61.2% of the transplanted microglia successfully populated the brain (Figures S6B–S6F). To enhance engraftment, we applied a two-cycle depletion protocol: one-month-old *Csf1r*^I792T/+^ mice received two consecutive 7-day PLX3397 treatments (600 mg/kg), separated by a 7-day regular diet to deplete resident microglia (Figure 5A). GFP^+^Iba1^+^ microglia from *Cx3cr1*^GFP/+^ mice were then transplanted into the brain. Immunofluorescent staining (Figures 5B–5D) and GFP^+^-based flow cytometry (Figures 5E and 5F) revealed robust and widespread colonization (>86%), with the exception of marginal brain regions distal to the injection site.

Thus, for the *Csf1r*^I792T/+^ mouse model of CSF1R-RD, we developed DCMDT, a simple strategy for microglial depletion that can be used to achieve efficient microglia replacement.

### Preventive microglial replacement protects against cognitive and pathological deficits in 1-month-old *Csf1r*^I792T/+^ mice

**Figure 6:**
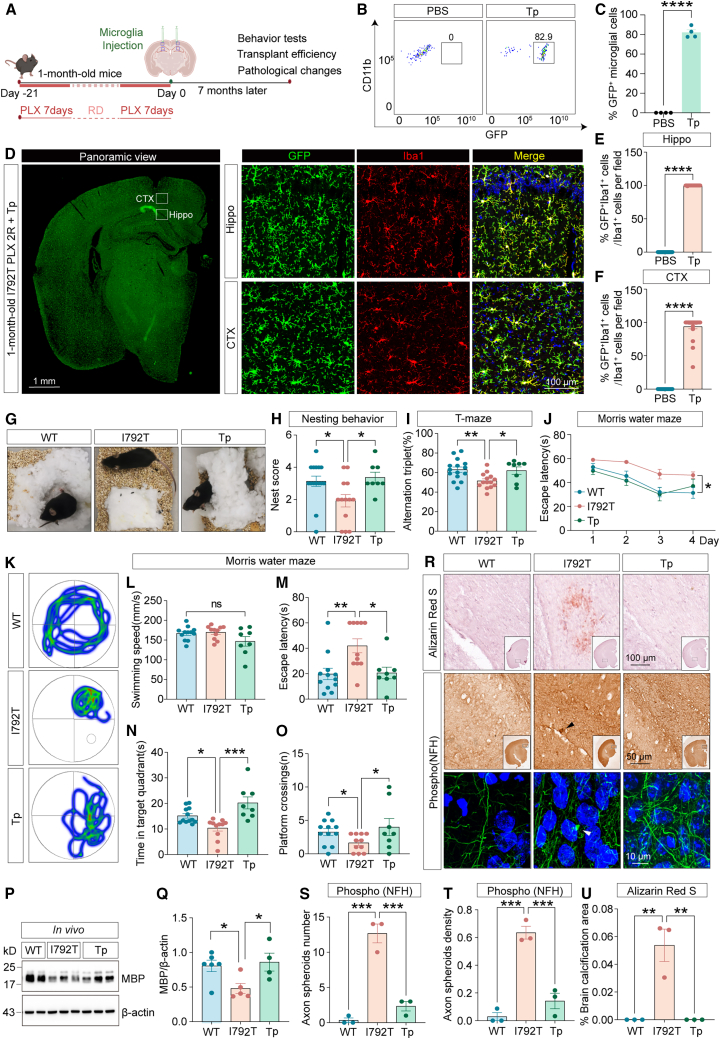
Preventive microglia replacement halts cognitive and pathological decline in *Csf1r*^I792T/+^ mice (A) Workflow of the preventive microglia replacement strategy. (B) Representative flow cytometry dot plots of GFP^+^CD11b^+^ microglia in transplanted *Csf1r*^I792T/+^ brains. (C) Donor-derived GFP^+^CD11b^+^ cells comprised up to 82.9% of the total CD11b^+^ cells (Tp mice). (D) Confocal images showed widespread colonization of GFP^+^ (green) donor microglia co-labeled with Iba1 (red) throughout Tp brain. Scale bars: 1 mm and 100 μm. (E and F) Quantification of the percentage of GFP^+^Iba1^+^ microglia relative to Iba1^+^ cells per high field in the hippocampus (E) and cortex (F) of *Csf1r*^I792T/+^ mouse brain (*n* = 3 per group). (G) Nest-building behavior was assessed in WT, I792T, and Tp mice as an indicator of cognitive performance. (H) Nesting scores were significantly improved in Tp mice compared to I792T mice (WT, *n* = 15; I792T, *n* = 14; Tp, *n* = 8). (I) In the T-maze test, Tp mice exhibited significantly increased spontaneous alternation compared to I792T mice (WT, *n* = 15; I792T, *n* = 14; Tp, *n* = 8). (J) In the MWM, Tp mice, similar to WT controls, showed shorter escape latency by day 4 of training compared to *Csf1r*^I792T/+^ mice (WT, *n* = 15; I792T, *n* = 14; Tp, *n* = 8). (K) Representative swimming trajectories during the MWM test for WT, I792T, and Tp mice. (L–O) Analysis of swimming speed (L), escape latency (M), time spent in the target quadrant (N), and number of platform crossings (O) on the test day confirmed improved spatial memory in Tp mice (WT, *n* = 15; I792T, *n* = 14; Tp, *n* = 8). (P) Representative western blot analysis of MBP expression in *Csf1r*^+/+^, *Csf1r*^I792T/+^, and Tp mouse brains. (Q) Quantification of MBP protein levels normalized to β-actin (*n* = 5 per group). (R–U) Representative images of brain calcifications (alizarin red S staining) and axonal spheroids (phospho-NFH immunostaining) in *Csf1r*^+/+^, *Csf1r*^I792T/+^, and Tp mice (R). Scale bars: 100, 50, and 10 μm. Axonal spheroid counts (S) and densities (T), as well as calcification areas (U), were quantified in WT, I792T, and Tp mice (*n* = 3 per group). Unpaired two-tailed Student’s *t* test (two groups); one-way ANOVA post-Dunnett’s multiple comparisons test (more than two groups). Data are presented as mean ± SEM. ∗*p* < 0.05, ∗∗*p* < 0.01, ∗∗∗*p* < 0.001, ∗∗∗∗*p* < 0.0001; ns, not significant. I792T, *Csf1r*^I792T/+^; Tp, *Csf1r*^I792T/+^ mice transplanted with microglia.

To determine whether DCMDT-based microglial transplantation could prevent the development of cognitive and pathological symptoms, 1-month-old *Csf1r*^I792T/+^ mice underwent two rounds of PLX3397 treatment followed by transplantation of GFP^+^ microglia (Figure 6A). Behavioral and pathological assessments were conducted 7 months later, at an age when cognitive deficits typically emerge. Flow cytometry (Figures 6B and 6C) and immunofluorescence (Figures 6D–6F) confirmed widespread engraftment of GFP^+^ microglia (82.9%). Transplanted mice exhibited significant improvements in cognitive performance, as demonstrated by higher nesting scores (Figures 6G and 6H) and improved performance in T-maze tests (Figure 6I). In the MWM, microglia-transplanted *Csf1r* ^I792T/+^ (Tp) mice showed significantly reduced escape latencies during the 4-day training session compared to non-transplanted controls (I792T) (Figure 6J). Swim speeds were comparable across groups during the probe trial (Figures 6K and 6L). However, Tp mice exhibited shorter escape latencies (Figure 6M), spent more time in the target quadrant (Figure 6N), and had increased platform crossings (Figure 6O), all indicating improved spatial memory. Importantly, western blot analysis revealed increased MBP in transplanted mice (Figures 6P and 6Q), suggesting prevention of demyelination. Transplantation also ameliorated key pathological hallmarks of CSF1R-RD, including axonal spheroids and cerebral calcification (Figures 6R–6U). Together, these findings demonstrate that early microglial replacement via DCMDT can prevent or delay the onset of cognitive and neuropathological deficits in young *Csf1r*^I792T/+^ mice.

### Therapeutic microglial replacement reverses cognitive and pathological deficits in 9-month-old *Csf1r*^I792T/+^ mice

**Figure 7:**
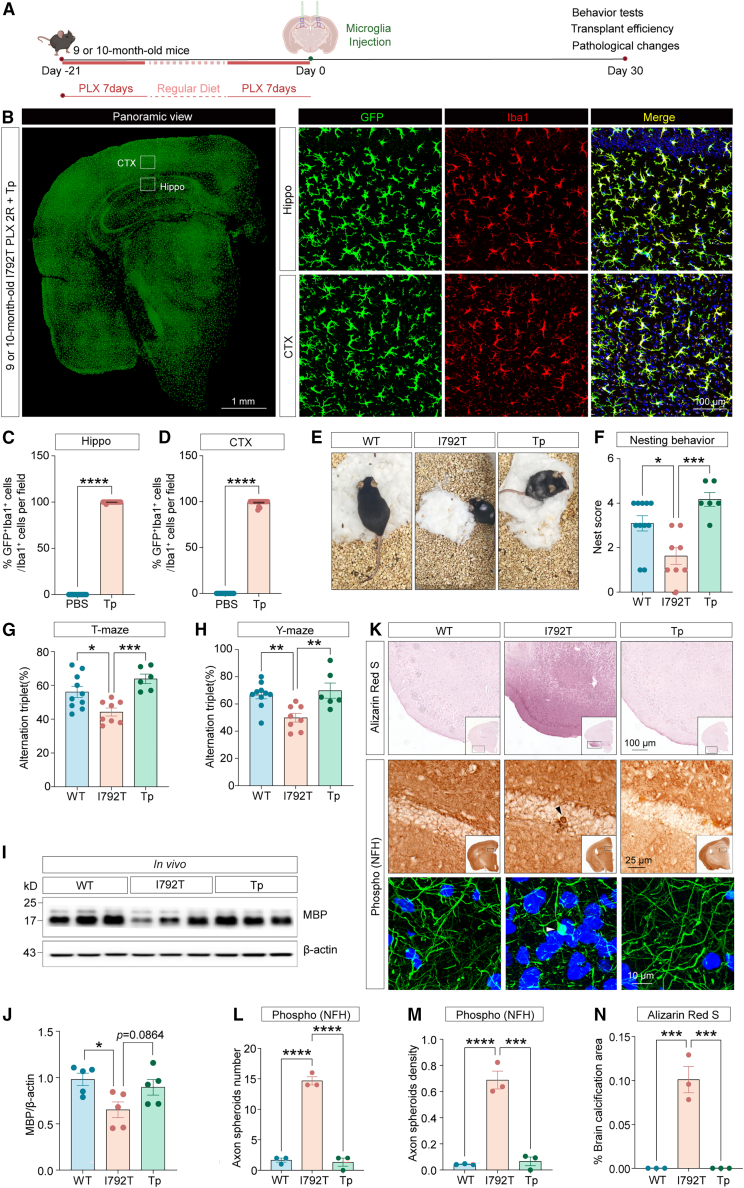
Therapeutic microglial replacement reverses cognitive and pathological deficits in *Csf1r*^I792T/+^ mice (A) Schematic overview of the therapeutic microglial transplantation strategy. (B) Representative confocal images of coronal brain sections showing widespread engraftment of GFP^+^ (green) Iba1^+^ (red) microglia in the brains of transplanted *Csf1r*^I792T/+^ mice. Scale bars: 1 mm and 100 μm. (C and D) Quantification of GFP^+^Iba1^+^ microglia as a percentage of total Iba1^+^ cells in the hippocampus (C) and cortex (D) of *Csf1r*^I792T/+^ mice. *n* = 4∼5 mice. (E) Representative images of nests built by *Csf1r*^+/+^, *Csf1r*^I792T/+^, or transplanted *Csf1r*^I792T/+^ mice. Nest quality was used as a proxy for cognitive performance. (F) Nesting scores quantified across groups: *Csf1r*^+/+^ (*n* = 10), *Csf1r*^I792T/+^ mice (*n* = 8), and transplanted *Csf1r*^I792T/+^ mice (*n* = 6). (G and H) T/Y-maze tests evaluating spontaneous alternation behavior. The percentage of alternation triplets was calculated for *Csf1r*^+/+^ (*n* = 10) and *Csf1r*^I792T/+^ mice (*n* = 8) and I792T mice transplanted with GFP^+^Iba1^+^ microglia (*n* = 6). (I) Representative western blot analysis of MBP expression in *Csf1r*^+/+^, *Csf1r*^I792T/+^, and transplanted *Csf1r*^I792T/+^ mouse brains. (J) Quantification of MBP protein levels normalized to β-actin (*n* = 5 mice). (K) Representative images of brain calcifications (alizarin red S staining) and axonal spheroids (phospho-NFH immunostaining) in WT, I792T, and Tp mice (*n* = 3 per group). Scale bars: 100, 25, and 10 μm. (L–N) Axonal spheroid counts (L) and densities (M), as well as calcification areas (N), were quantified in WT, I792T, or Tp mice. Unpaired two-tailed Student’s *t* test (two groups); one-way ANOVA post-Dunnett’s multiple comparisons test (more than two groups). Data are presented as mean ± SEM. ∗*p* < 0.05, ∗∗*p* < 0.01, ∗∗∗*p* < 0.001, ∗∗∗∗*p* < 0.000. RD, regular diet. WT, wild-type; I792T, *Csf1r*^I792T/+^; Tp, *Csf1r*^I792T/+^ mice transplanted with microglia.

Given the protective effects of preventive microglial replacement and the adult-onset nature of CSF1R-RD, we next assessed whether therapeutic microglial transplantation could reverse established cognitive and pathological deficits in 9-month-old *Csf1r*^I792T/+^ mice. We applied the DCMDT protocol to 9-month-old *Csf1r*^I792T/+^ mice (Figure 7A). Following treatment, transplanted GFP^+^Iba1^+^ microglia robustly colonized nearly the entire brain, as confirmed by immunofluorescence (Figures 7B–7D). Behavioral deficits were significantly improved: nesting performance (Figures 7E and 7F) and T/Y-maze scores (Figures 7G and 7H) were restored. While the increase in MBP levels did not reach statistical significance, a trend toward demyelination recovery was observed (Figures 7I and 7J). Furthermore, axonal spheroids and cerebral calcifications were ameliorated in transplanted mice (Figures 7K–7N).

These results indicate that even at advanced disease stages, microglial replacement using the DCMDT strategy can significantly reverse cognitive impairments and key pathological features in the *Csf1r*^I792T/+^ model of CSF1R-RD. These findings highlight the translational potential of therapeutic microglial transplantation for adult-onset microgliopathies.

To determine whether transplanted microglia re-establish a functional phenotype in *Csf1r* ^I792T/+^ mice and to elucidate the mechanisms by which microglial transplantation mitigates microgliopathy, we performed RNA-seq analysis on mouse brains (Figure S7A). Strikingly, *Csf1r* ^I792T/+^ mice exhibited downregulation of genes associated with inflammation (such as *ApoE*, *Tlr2*, and *Tgfbr1*), axonal regeneration, and memory-related processes (such as *Bex1*, *Bex2*, *Cckbr*, and *Rab3b*) compared to *Csf1r*^+/+^ controls.

To further explore the transcriptional impact of microglial transplantation, we conducted weighted gene co-expression network analysis (WGCNA) and KEGG pathway enrichment on RNA-seq data from the three experimental groups. WGCNA identified two key modules (Blue and Turquoise) that strongly correlated with pathological severity and responded to transplantation (Figure S7B). KEGG analysis revealed that genes within the Blue module were enriched in pathways related to “cellular senescence,” “cell cycle,” and “virus infection” (Figure S7C), while those in the Turquoise module were associated with “axon guidance,” “pathways of neurodegeneration,” and “multiple diseases” processes (Figure S7D). These data suggest that microglial transplantation may exert protective effects by reprogramming dysfunctional gene networks implicated in CSF1R-RD.

Notably, transplantation of WT microglia significantly restored or exhibited a recovery trend in the expression of *Csf2/3* and DAM genes, including *Tmem119*, *Cst7*, and *H2-Ab1* in *Csf1r* ^I792T/+^ mice (Figure S7E), indicating a transcriptional reversion toward the WT profile. Together, these findings indicate that DCMDT-based microglial transplantation effectively reverses both cognitive impairments and neuropathological hallmarks in the *Csf1r*^I792T/+^ mouse model, highlighting its potential as a therapeutic strategy for CSF1R-RD.

## Discussion

CSF1R-RD is increasingly recognized as a globally prevalent condition,^6^^,^^20^ with numerous variants and cases reported in diverse populations.^7^^,^^34^^,^^35^^,^^36^^,^^37^^,^^38^^,^^39^ A comprehensive review by Dulski et al. detailed the clinical heterogeneity of CSF1R-RD, noting its occurrence across a wide age range, from early onset (<18 years) to late onset (≥18 years), yet sharing a similarly progressive neurodegenerative course.^39^ Common clinical manifestations include spasticity, rigidity, cognitive decline, and eventual loss of voluntary movement and speech. Pneumonia and secondary infections are the leading causes of death in affected individuals. To date, over 500 cases have been documented worldwide.^39^ Among known mutations, the c.2381T > C (p.I794T) variant has emerged as a mutational hotspot, particularly prevalent in Chinese patients.^7^ The high frequency of this variant underscores the urgent need for targeted therapies addressing shared pathogenic mechanisms in CSF1R-RD.

In the present study, we report three probands carrying the CSF1R p.I794T variant. By reviewing all published CSF1R mutations, we confirm that p.I794T is one of the most common disease-causing variants globally. Our analysis of clinical and neuroimaging data from CSF1R p.I794T patients reveals that cognitive impairment is the most frequent initial symptom. In parallel, we generated a *Csf1r*^I792T/+^ knockin mouse model via homologous recombination. This model replicates the clinical and pathological features of CSF1R-RD with early synaptic dysfunction evident at 6 months, preceding detectable cognitive deficits at 9 months, a timeline that aligns with human disease progression. Thus, this model provides a reliable platform for dissecting the role of microglial dysfunction in CSF1R-RD pathogenesis. The underlying cause of the approximately 50% survival rate observed in *Csf1r*^I792T/+^ mice remains unclear. Interestingly, we found that the surviving mice show significantly reduced CSF1R protein levels at an aged time point (20–24 months, data not shown). This suggests that a subset of mice might possess compensatory mechanisms that allow them to tolerate the chronically low level of CSF1R signaling.

Consistent with findings by Biundo et al., who demonstrated that heterozygous deletion of *Csf1r* in microglia but not in neurons causes neurodegeneration,^10^ our results reinforce the classification of CSF1R-RD as a primary microgliopathy. While earlier studies have described microgliosis in *Csf1r*^+/−^ mice and microglial deletion (M*Csf1r*^het^) mice,^9^^,^^10^^,^^28^ decreased microglial density has also been reported in inducible *Csf1r*^+/−^ models,^11^ *Csf1r*^E631K/+^ mice,^23^ *Csf1r* mutant zebrafish,^40^ and *Csf1r*^+/−^ mice,^12^ as well as in CSF1R-RD patients.^9^^,^^41^^,^^42^^,^^43^^,^^44^ Consistent with these studies, our data demonstrate that the p.I794T variant causes a marked reduction in microglial cell numbers.

Although numerous models (mice, rats, and zebrafish) have been employed to study the loss of one *Csf1r* allele or CSF1R mutations, the molecular mechanisms underlying disease pathogenesis remain incompletely understood. Given that CSF1R-RD is a dominantly inherited leukoencephalopathy, we hypothesized that microglial dysfunction may emerge early and drive progressive white matter pathology. To test this, we performed microglial RNA-seq and identified dysregulated molecular pathways in microglia from both 0-to 3-day-old and 9-month-old *Csf1r*^I792T/+^ mice. Notably, altered expression of cell cycle-related genes in 0- to 3-day-old *Csf1r*^I792T/+^ microglia may underlie reduced microglial numbers. Moreover, activated transcriptional signatures in both 0- to 3-day-old and 9-month-old *Csf1r*^I792T/+^ microglia suggest a pathologically primed microglial state, an observation consistent with prior studies in CSF1R-deficient models.^12^ Additionally, while Chitu et al. reported elevated *Csf2* and *Csf3* expression in both 7-week-old *Csf1r*^+/−^ mouse model and CSF1R-RD patients,^27^^,^^28^ this upregulation was not expected to occur in the 9-month-old *Csf1r*^I792T/+^ mice in this study, as *Csf2*, at least, is predominantly expressed by non-microglial cells, secondary to changes in microglia and may develop later.

Pathway analyses (GSEA and KEGG) further revealed that the *Csf1r* p.I792T variant in 0- to 3-day-old microglia activates genes involved in cytokine-receptor interactions, processes known to contribute to phagosome, neuroinflammation, and microglial dysfunction. This neonatal microglial dataset, while not directly applicable to adult pathology, provides valuable developmental insights and a foundation for future studies investigating microglial biology during early brain maturation. Notably, microglia isolated from 9-month-old *Csf1r*^I792T/+^ mice exhibited an activated and DAM-like phenotype, marked by significantly increased *Stat3*, *Mrc2*, *Atg7*, *Chil3*, and *Cst7* expression, as well as reduced *Tmem119* and *P2ry12* expression, consistent with established neurodegenerative profiles.^29^^,^^30^^,^^31^ Complementary WGCNA and KEGG analysis of whole-brain RNA-seq data in middle-aged *Csf1r*^I792T/+^ mice revealed enrichment in pathways related to cellular senescence, cell cycle, and virus infection, mirroring findings from the microglial transcriptome. These data support the conclusion that the *Csf1r* p.I792T variant induces a proinflammatory, phagocytic microglial phenotype that is central to CSF1R-RD pathogenesis. Furthermore, transcriptional analysis of DAM from whole-brain tissue revealed their heterogeneous nature. The finding of divergent DAM markers in the brain, as confirmed by qPCR, indicates that the *Csf1r* p.I792T mutation may drive microglia into a mixed activation state rather than a canonical DAM phenotype or a homogeneous one. These results are, in fact, consistent with those from a recent publication, in which several microglia/macrophage clusters were identified in the mutant mouse brains by single-cell RNA-seq.^16^ This complex response likely manifests as distinct functional subpopulations across the brain. This interpretation also aligns with a recent study reporting loss of homeostatic microglia in white matter from intermediate and late-stage CSF1R-RD postmortem brains.^40^ Therefore, future investigations must employ advanced techniques such as single-cell RNA-seq and spatial transcriptomics, which are critical to resolve the precise heterogeneity of mutant microglia, to define the unique molecular states, and to map their distribution across different brain regions. For example, understanding the functional consequences of white versus gray matter microglial heterogeneity for neuronal function represents a compelling frontier for future studies. Altogether, our findings demonstrate that microglial dysfunction alone is sufficient to drive the behavioral deficits and neuropathological features observed in the *Csf1r*^I792T/+^ mouse model of CSF1R-RD.

Due to the rapidly progressive nature of CSF1R-RD, there is an urgent need to develop effective early interventions. While HSCT has been proposed as a disease-modifying therapy,^16^ its benefits appear limited, particularly in patients with advanced cognitive decline, yielding variable and often unsatisfactory outcomes.^15^ Recently, the transplantation of human induced pluripotent stem cell (iPSC)-derived microglial (iMG) progenitors has emerged as a promising alternative approach.^17^ Nevertheless, there remains a significant lack of effective therapeutic options for CSF1R-RD to date. Notably, we found that the endogenous microglial repopulation capacity was markedly impaired in 1-month-old *Csf1r*^I792T/+^ mice, highlighting a proliferation defect intrinsic to the CSF1R p.I794T variant. Given the observed reduction in both microglial numbers and function in the brains of *Csf1r*^I792T/+^ mice, we investigated whether replacing dysfunctional microglia with healthy ones could mitigate disease progression. Our findings demonstrate that early microglial replacement significantly delayed or prevented the onset of cognitive and pathological deficits in young *Csf1r*^I792T/+^ mice. Remarkably, this intervention also reversed behavioral and neuropathological impairments in 9-month-old *Csf1r*^I792T/+^ mice that manifested CSF1R-RD-like symptoms. We noted the discrepancy in the MWM baseline, where the I792T learning deficit appears earlier in the rescue cohort (days 2–3) compared to the initial characterization (day 5). In addition to the microinjection procedure, the experimental and biological variabilities can also contribute to these differences. For example, subtle differences in the testing environment between two experimental batches could influence the sensitivity and performance outcomes of the MWM assay. Moreover, variations in handling, experimenter influence, inter-trial intervals, or time of day for testing are all known factors that can alter learning curves. Furthermore, because the I792T mutation might not cause a perfectly uniform phenotype, animals may respond differently to procedural or environmental stressors, leading to apparent timing discrepancies in learning deficits.

Transcriptomic profiling following microglial transplantation revealed, via WGCNA and KEGG analyses, the enrichment of pathways related to axon guidance, neurodegeneration, and other multiple diseases processes, underscoring the therapeutic potential of microglial replacement in attenuating CSF1R-RD neurodegeneration.^28^ These results provide a compelling rationale for microglial transplantation as a therapeutic strategy not only for early-stage intervention but also in symptomatic individuals.

To develop a more clinically feasible microglial transplantation approach, we also attempted direct microglial transplantation and a single-round microglia depletion followed by transplantation in 9-month-old *Csf1r*^I792T/+^ mice. We initially tried to isolate microglia from postnatal day 1 mice using FACS for transplantation. However, this approach yielded limited cell numbers with poor viability as demonstrated by CCK-8 assays (Figure S8A). Furthermore, the sorted microglia failed to migrate from the injection site, indicating their unsuitability for transplantation studies (Figures S8B and S8C). We next attempted two transplantation approaches in 9-month-old *Csf1r*^I792T/+^ mice: (1) direct transplantation without prior microglial depletion (Figures S9A–S9C) and (2) transplantation following single microglial depletion (Figures S9D–S9F). Both strategies proved unsuccessful for microglial replacement in aged *Csf1r*^I792T/+^ mice. While a recent study achieved successful transplantation in 16-month-old mice with a 14-day PLX5622 regimen,^16^ in this study, a single 7-day administration of PLX3397 failed to achieve complete microglial clearance in 9-month-old *Csf1r*^I792T/+^ mice (data not shown) and was, therefore, inadequate for supporting subsequent efficient engraftment. Two non-mutually exclusive hypotheses may explain the low engraftment rate (1.12%) in 9-month-old symptomatic *Csf1r*^I792T/+^ mice after a 7-day PLX treatment. First, aged microglia themselves may be more resistant to depletion, creating a crowded niche that impedes donor cell entry. Second, the aged brain may constitute a dysfunctional niche, potentially deficient in critical nutrients or trophic factors, thereby impairing the adaptation and survival of transplanted microglia. The failure of these protocols to achieve therapeutic engraftment may highlight the necessity of creating an optimal niche for donor cell engraftment in aged mice. Our data indicate that for more robust efficacy across both preventive and therapeutic settings, two alternating rounds of depletion are required. Therefore, two-phase microglia depletion may ensure complete niche clearance, critical for donor cell engraftment, and may overcome the competitive advantage of resident dysfunctional microglia, a challenge not addressed by single-dose regimens. Thus, the developed depletion and replacement strategy termed “DCMDT” in this study may overcome these limitations and enable efficient microglial turnover in both the young and old *Csf1r*^I792T/+^ mouse model.

Although a recent study demonstrated that transplantation of iMG progenitors could prevent pathological progression in a CSF1R-FIRE-deficient mouse model,^17^ and other approaches have also shown promise,^45^^,^^46^ further clinical trials are essential to evaluate the efficacy and long-term safety of these therapies. Additionally, the recent generation of iMGs from a CSF1R-RD patient carrying a pathogenic variant, achieved by optimizing an existing iPSC-derived microglia protocol, offers tools to explore human-specific disease mechanisms and therapeutic responses.^47^ From a translational perspective, future studies should explore alternative delivery methods such as intravenous or intrathecal injection for microglial replacement therapy.

Collectively, our findings identify microglial dysfunction as a central pathogenic mechanism in CSF1R-RD and provide strong evidence that restoring functional microglial populations can ameliorate disease. This study elucidates the molecular underpinnings of CSF1R variant-mediated microglial impairment and establishes microglial replacement as a viable therapeutic strategy. Moreover, the clinical approval status of PLX3397 renders it more suitable for translational applications, thereby potentially accelerating the clinical adoption of DCMDT for treating CSF1R-RD patients. Ultimately, our work lays the groundwork for future efforts aimed at mitigating microglia-associated neurodegeneration in CSF1R-RD.

### Limitations of the study

Several limitations warrant consideration in this study. First, CSF1R-RD represents a rare neurodegenerative leukoencephalopathy. Despite efforts to expand the sample number in this study, the limited cohort may impede a comprehensive assessment of the clinical heterogeneity associated with the CSF1R p.I794T mutation. Second, although the *Csf1r*^I792T/+^ mouse model generated via homologous recombination recapitulates core cognitive pathological features observed in human CSF1R-RD, inherent interspecies differences in neuroanatomical structure, immune function, and disease progression may constrain direct translatability between observed phenotypes and clinical pathology. Third, although the DCMDT strategy demonstrates neuroprotective effects in *Csf1r*^I792T/+^ mouse model, its clinical translational potential requires further validation. Before clinical application, key challenges, including immune compatibility, engraftment efficiency, and technical complexity, must be addressed. Finally, transcriptomic analysis suggests a DAM-like phenotype in *Csf1r*^I792T/+^ microglia; however, how *Csf1r*^I792T/+^ microglia contribute to leukoencephalopathy remains to be elucidated and the specific molecular mechanisms need to be fully defined.

## Resource availability

### Lead contact

Further information and requests for resources and reagents should be directed to and will be fulfilled by the lead contact, Honghua Zheng (honghua@xmu.edu.cn).

### Materials availability

This study did not generate new unique reagents.

### Data and code availability

• Raw RNA-seq data used in the study are available at https://db.cngb.org/ using accession code CNP0007429. All data reported in this paper will be shared by the lead contact upon request.
• This paper does not report original code.
• Any additional information required to reanalyze the data reported in this paper is available from the lead contact upon request.

## Acknowledgments

This work was supported by grants from the 10.13039/501100001809National Natural Science Foundation of China (82271219, 91949129, 32471045, and 82522029) and the program of Lin Gang Laboratory LGL-3142-ADB120102 (to H.Z.). This work was also supported by a grant from the 10.13039/501100003452Innovation and Technology Commission (ITCPD/17-9) (to G.B.). The authors thank Zicheng Huang from the Center for Molecular Imaging and Translational Medicine of Xiamen University for her professional help in MRI analysis. The authors also thank the colleagues from the Biomedical Shared Research Platform of Xiamen University for their technical help: Haiping Zheng for flow cytometry, Baoying Xie for behavioral tests, Xiang You and Jingru Huang for imaging, and Luming Yao for electron microscopy.

## Author contributions

H.Z., L.Z., Y.-W. Z., and Z.Z. contributed to the conception and design of the study; C.W. provided the pedigree and imaging data of the patients in this study; X.L. and H.F. collected and analyzed the clinical information from all patients carrying the CSF1R I794T mutation, both in this study and those reported in the literature. The behavior test, western blot, immunofluorescence staining, flow cytometry, MRI scanning, and electron microscopy experiments were conducted by X.L. and B.H.; alizarin red S staining and NFH immunohistochemical staining were carried out by X.L.; RNA sequencing data were analyzed by Z.W.; electrophysiological experiments were performed by B.H.; additionally, X.H., L.Z., and Y.L. contributed unpublished reagents/analytic tools; G.B. contributed to conceptual and experimental advice on the project; H.Z., X.L., B.H., C.W., Y.L., X.Z., and L.Z. prepared the figures. X.L. wrote the STAR Methods section. C.W. documented the clinical and imaging characteristics of the patients. H.Z. wrote the main text. All authors commented on the manuscript and approved the final manuscript.

## Declaration of interests

The authors declare no competing interests.

## STAR★Methods

### Key resources table

**Table:** 

REAGENT or RESOURCE	SOURCE	IDENTIFIER
**Antibodies**		

Anti-GAPDH antibody	Abcam	Cat. #ab8245; RRID: AB_2630358 RRID: AB_2630358
Anti-CSF1R	R&D Systems	Cat. #AF3818; RRID: AB_884158
Iba1/AIF-1 (E4O4W) XP® Rabbit mAb (17198)	Cell Signaling Technology	Cat. #17198S; RRID: AB_2820254
Donkey anti-Rabbit IgG (H + L) Highly Cross-Adsorbed Secondary Antibody, Alexa Fluor™ 488 Anti-Rabbit Recombinant Secondary Antibody (H + L)	Invitrogen	Cat. #A21206; RRID: AB_2535792
Anti-Neurofilament H Phospho (phos-NFH) Antibody	Biolegend	Cat. #840801; RRID: AB_2565456
Donkey anti-Rabbit IgG (H + L) Highly Cross-Adsorbed Secondary Antibody, Alexa Fluor™ 568 Cross-Adsorbed Secondary Antibody, Alexa Fluor™ 568	Invitrogen	Cat. #A10042; RRID: AB_2534017
MBP antibody (F-6)	Santa_Cruz	Cat. #sc-271524; RRID: AB_10655672
CD11b Monoclonal Antibody (M1/70), APC	Invitrogen	Cat. #17-0112-83; RRID: AB_469344 AB_
CD45 Monoclonal Antibody (30-F11), FITC	Invitrogen	Cat. #11-0451-82; RRID: AB_465050
Iba-1 antibody	Wako	Cat. # 019–19741; RRID: AB_839504
IBA1 antibody guinea pig	SYSY	Cat. # 234 004; RRID: AB_2493179
CD45 Monoclonal Antibody (30-F11), PE-Cyanine7 eBioscience™ (30-F11), PE-Cyanine7, eBioscience™	Invitrogen	Cat. # A18710; RRID: AB_2535494, RRID: AB_2535494 AB_
Anti-β-actin antibody	Abcam Cat. #ab8226,	Cat. #ab8226; RRID: AB_306371 AB_306371
Goat anti-Guinea Pig IgG (H + L) Highly Cross-Adsorbed Secondary Antibody, Alexa Fluor™ 647	Invitrogen	Cat. #A-21450; RRID: AB_2535867

**Chemicals, peptides, and recombinant proteins**		

GM-CSF	novoprotein	Cat. #CK02
PLX3397	MedChemExpress	Cat. #HY-16749
Poly-L-lysine	Sigma	Cat. #P1274
Bovine Serum Albumin V	Solarbio	Cat. #A8020
Isoflurane	RWD	Cat. #R510-22-10
DAPI	Yeasen	Cat. #40728ES03
RIPA buffer	LABLEAD	Cat. #R1091

**Critical commercial assays**		

RNA isolater Total RNA Extraction Reagent	Vazyme	Cat. #R401-01
HIScript III All-in-one RT SuperMix Perfect for qPCR	Vazyme	Cat. #R333-01
HamQ Universal SYBR qPCR Master Mix	Vazyme	Cat. #q711-03
Protein Quantification Kit (BCA Assay)	Abbkine	Cat. #KTD3010-CN
SuperKine ECL detection kit	Abbkine	Cat. #BMU102-CN
Biotin-labeled antibodies, anti-streptomycin peroxidase	MXB	Cat. #Kit-9270
DAB Kit	MXB	Cat. #DAB0031
Triton™ X-100	Sigma	Cat. #T8787
Alizarin Red S	Solarbio	Cat. #G1450
100×proteinase inhibitor cocktail	APE×BIO	Cat. #k1007
100×phosphatase inhibitor cocktail	APE×BIO	Cat. #k1015
DMEM	Pricella	Cat. #PM150210)
1% penicillin/streptomycin	BasalMedia	Cat. #S110JV
Fetal bovine serum	ExCell Bio	Cat. #FSD500

**Experimental models: Cell lines**		

Primary microglia This study N/A	This study	N/A

**Experimental models: Organisms/strains**		

*Csf1r*^I792T/+^ knock-in mice (C57BL/6J)	GemPharmatech	Cat. #GPS00001710
*Cx3cr1*^GFP/+^ mice	Jackson Laboratory	Cat. #008451

**Deposited data**		

Raw RNA-seq data	This paper; CNP0007429	https://db.cngb.org/
Raw data from Figures 1, 2, 3, 4, 5, 6, and 7	This paper; Mendeley Data	https://doi.org/10.17632/j7n9jhnv5j.1
Raw data from Figures S1–S9	This paper; Mendeley Data	https://doi.org/10.17632/j7n9jhnv5j.1

**Software and algorithms**		

Prism 10.0.2	GraphPad	https://www.graphpad.com
Fiji	National Institute of Health USA	https://imagej.net/software/fiji
Excel	Microsoft Office	N/A
FV10-ASW 4.2 Viewer	Olympus	N/A
ImageScope x64	Leica	N/A
ZEISS ZEN 3.11	ZEISS	N/A
FlowJo_V10.exe	BD Life Sciences	N/A
Imaris 9.2.1	Bitplane	N/A
BioRender	BioRender	https://www.biorender.com/

### Experimental model and study participant details

#### Patients with CSF1R-RD

Clinical data from 3 genetically diagnosed probands and their family members who met published criteria for definite CSF1R-RD were obtained from the Department of Neurology, Beijing Tiantan Hospital, Capital Medical University (Medical ethics permission number KY2020-105-02). This observational study adhered to the relevant Strobe guidelines/requirements for cohort studies. Written informed consent was obtained from all participants.

#### Mice

*Csf1r*^I792T/+^ knock-in mice (C57BL/6J) were customized by GemPharmatech Co. Ltd using homologous recombination-based gene editing (project number: GPS00001710)*.* Mice were bred by heterozygous self-crossing. *Cx3cr1*^GFP/+^ (stock 008451) mice were purchased from the Jackson Laboratory. All mice were housed in Xiamen University Laboratory Animal Center, with 12 h of alternating light/darkness, an ambient temperature of 18°C–29°C, relative humidity of 40–70%, and free access to water and food. Mice were randomly assigned for biological analyses, and the researchers performed double-blind analysis during the experiments and results evaluation. All animal experiments complied with the relevant regulations of the Laboratory Animal Management and Ethics Committee of Xiamen University (XMULAC20220235).

### Method details

#### Flow cytometry sorting of microglia

For the transcriptomic analysis of adult mouse microglia, 9-month-old WT or *Csf1r*^I792T/+^ mice were perfused with pre-cooled PBS and the single-cell suspensions of the brain tissues were then mechanically prepared using a Dounce homogenizer. The suspensions were subjected to centrifugation (800 g, 10 min, 4°C) over a 30% Percoll (GE Healthcare) density gradient. The myeloid cell-enriched fractions were harvested from the interphase layer. Cells were stained with CD11b-APC and CD45-FITC antibodies. Microglia (CD11b^+^CD45^low^) were finally sorted using a MoFlo Astrios EQ 2 (Beckman, USA) cell sorter for subsequent transcriptomic analysis.

For the confirmation of engraftment of GFP^+^ microglia, transplanted mice were perfused with pre-cooled 1×PBS and brain tissues were then dissociated enzymatically and homogenized into a single-cell solution in pre-cooled HBSS mixed with 1% FBS using a glass Dounce homogenizer. Single cell suspensions were centrifuged on a discontinuous Percoll (GE Healthcare) gradient. Monocytes were isolated from the interphase of these layers. Cells were stained with CD11b-APC and CD45-PeCy7 for sorting CD11b^+^CD45^low^ microglia and CD11b^+^CD45^low^GFP^+^ transplanted microglia. Cells were isolated by using CytoFlex S (Beckman, USA).

#### Primary microglia isolation

Primary microglial cultures were prepared as previously described.^12^ Brains from postnatal day 1 (P1) or P2 *Csf1r*^GFP/+^, *Csf1r*^+/+^ or *Csf1r*^I792T/+^ mice were mechanically dissociated. The mixed brain cells were resuspended by DMEM containing 10% FBS and 1% Penicillin/Streptomycin (P/S) (100×) and plated onto a poly-L-lysine-coated 175 cm^2^ flask. After two days, the medium was replaced with the complete DMEM containing 25 ng/mL GM-CSF. Subsequently, 5 mL GMCSF-containing medium was added three days later. Primary microglia were harvested by shaking the flask at 200 rpm for 20 min. This step was repeated for further microglia collection every 3 days. Isolated primary microglia were then cultured in a 6-well plate at the density of 2 × 10^6^ cells/well for further experiments. Cells were cultured in a humidified chamber with 5% CO_2_ at 37°C. Cells from the first harvest with viability >95% were used for subsequent transplantation experiments.

#### Microglia depletion and repopulation

To deplete resident microglia in the brain, mice were fed freely with PLX3397-containing chow (SHUYISHUER BIO) at a dose of 600 mg/kg for 3, 5, or 7 days. The microglia were then repopulated by resuming regular diet (RD) for 7 days. Microglia elimination was verified by Iba1 immunofluorescent staining. Mice that subjected to one or two cycles of PLX3397 treatment were used for *Cx3cr1*^GFP/+^ microglia transplantation.

#### Microglia transplantation

Microglial transplantation was performed as follows. Briefly, 1-month-old or 9-month-old mice received a volume of 2 μL *Cx3cr1*^GFP/+^ microglia at a density of 2 × 10^4^ cells/μL at each injection site (four sites, hippocampus, X/Y/Z, ±1.75/-2.0/-2.0 mm; cortex, X/Y/Z, ±1.75/-2.0/-0.85 mm) for a total of 8 × 10^4^ cells/mouse at a rate of 0.5 μL/min with a 10 μL microinjector. After each injection, the microinjector remained at the injection site for four minutes to ensure that the grafted microglia entered the brain tissue efficiently. Microglia replacement strategies in different figures were listed in Table S4.

#### RNA-sequencing

The transcriptome RNA libraries were from brain (*n* = 5 per group) or microglia (*n* = 3 per group) derived from *Csf1r*^+/+^ or *Csf1r*^I792T/+^ mice. The RNA-seq experiment was performed using the PE150 strategy on the Illumina HiSeq 2500/4000 platform, yielding an average of 10 G reads per sample. Differentially expressed genes (DEGs) between *Csf1r*^+/+^ and *Csf1r*^I792T/+^ microglia were identified and compared using the DESeq2 (v1.30.1) software package (adjusted *p*-value <0.05, |log_2_ fold change| > 1). Enrichment analysis of DEGs, including Gene Ontology (GO) analysis, Kyoto Encyclopedia of Genes and Genomes (KEGG), or Gene Set Enrichment Analysis (GSEA), was performed to identify the significant biological pathways or highlighting biological processes with high confidence by using clusterProfiler (v3.18.1) (adjusted *p*-value <0.05 and false discovery rate (FDR) q-value <0.05) in those genes.^48^^,^^49^ The ggplot2 (3.5.1) package was used for visualizing the DEGs and enriched pathways. Data from mouse brain samples were used for detailed gene set enrichment analysis and weighted gene co-expression network analysis (WGCNA) (v1.70.3). All analyses were conducted in R version 4.0 or above.

#### Electrophysiological recording

Mice were deeply anesthetized with isoflurane and the brain was collected and immediately placed in ice-cold artificial cerebrospinal fluid containing the following composition: 64 mM NaCl, 2.5 mM KCl, 1.25 mM NaH_2_PO_4_, 10 mM MgSO_4_, 0.5 mM CaCl_2_, 26 mM NaHCO_3_, 10 mM D-glucose, and 120 mM Sucrose (pH 7.4, 290–310 mOsm). Coronal brain slices (400 μm) were made with a Leica VT1200S vibrating microtome and transferred to a chamber containing artificial cerebral spinal fluid (126 mM NaCl, 3.5 mM KCl, 1.25 mM NaH_2_PO_4_, 1.3 mM MgSO_4_, 2.5 mM CaCl_2_, 11 mM NaHCO_3_, and 10 mM Glucose, pH 7.4, 290–300 mOsm) for 1 h at 32°C. The slices were recovered at room temperature (RT) for at least 1 h before recordings were performed. All solutions were bubbled with 5% CO_2_/95% O_2_. Field potential recordings were recorded by Axon Digidata 1550 (USA) and Diaphragm clamp Amplifier (Axon CNS, Multiclamp 700B, USA). Schafer peripheral inputs in CA3 were stimulated by Concentric Bipolar Electrode (FHC, CBARC75, Inc. Bowdoin, ME, USA). Field excitatory postsynaptic potentials (fEPSPs) of Schaffer lateral pathways in the CA1 were recorded with 800 kΩ-2 MΩ epoxy glass (Sutter Instrument, BF150-86–10, USA). After a 20-min stable baseline recording, long-term potentiation (LTP) was induced by high-frequency stimulation (HFS, two trains of 100-Hz stimuli with an interval of 30 s), followed by continued recording for 60 min.

#### T/Y-maze test

Mice were allowed to free access to T-maze arms (30 cm × 6 cm × 10 cm) or all three 120° arms of the Y-maze (8 cm × 30 cm × 15 cm) for 5 min. Mice were placed in the center for all tests and the trajectory of mice was collected by Clever Sys system for 5 min. An alternation was defined as a consecutive entry in all three arms and was calculated automatically using CleverSys TopScan Lite, an automated video analysis system (Clever Sys., Inc. Reston, Virginia, USA).

#### Open-field test

We followed a protocol that has been previously described.^12^ Mice were carried to the behavior room at least 30 min before starting the test to habituate to the environment. Mice were placed into the open-field arena (50 cm × 50 cm) to explore freely. Spontaneous activities were monitored for 10 min and the percentage of duration in the center (24 cm × 24 cm) was analyzed using CleverSys TopScan Lite, an automated video analysis system (Clever Sys., Inc. Reston, Virginia, USA).

#### High plus maze test

The high plus maze contains 2 open arms (35 × 5 cm) and 2 enclosed arms (35 × 5 cm) extending from a central platform measuring (7.5 × 7.5 cm). Tests were carried out in a quiet and dimly lit environment. The apparatus was wiped clean with 75% ethanol between tests. Mice were placed in the center of the plus-maze, facing one of the open arms. Spontaneous activities were monitored for 10 min and the duration in the open arms was analyzed using CleverSys TopScan Lite, an automated video analysis system (Clever Sys., Inc. Reston, Virginia, USA).

#### Morris water maze test (MWM)

The water maze consists of a pool (110 cm in diameter) (water temperature was kept at 22 ± 1°C) and a platform (10 cm in diameter) submerged 1.0 cm under the water. The MWM test consisted of 6- days of training trials and 1 day of test. During the training trial, mice were allowed to swim for 1 min to arrive at the hidden platform and stay on it for at least 10 s to remember the position of the platform. Mice that were unable to find the platform were guided to it. Mice were trained for 6 consecutive days. On the 7^th^ day, the platform was removed and the test was performed. The duration in the target quarter, platform crossings and swimming speed were recorded and analyzed using CleverSys TopScan Lite, an automated video analysis system (Clever Sys., Inc. Reston, Virginia, USA).

#### Nesting behavior test

*Csf1r*^+/+^ or *Csf1r*^I792T/+^ mice were isolated in separate cages prior to the start of the test for 1 day. Nesting behaviors were manually scored according to the standard scale of 0–5.^50^^,^^51^ 0, no nest shredding (flat nest); 1, not noticeable nest shredding (>90% nestlet untorn); 2, partially nest shredding (50%–90% nestlet untorn); 3, mostly nest shredding (<50% nestlet untorn); 4, recognizable but flat nest shredding (<10% nestlet untorn), 5, high-sided nest with all material shredded (“brooding” nest).

#### *In vivo* MRI scanning

Mice were anesthetized with isoflurane and fixed in a magnetic resonance imaging (MRI) matched brain coil (9.4T MRI, Bruker). Mice were maintained anesthesia at 37°C during the scan using 1.5%–2% isoflurane in oxygen/air (50/50, 1 L/min). Two-dimensional 15- to 30-level T2-weighted scans were performed to identify areas where the hippocampus and lateral ventricle were located (0.5 mm slice thickness, image size/data matrix = 256 × 256 pixels, field of view = 30 × 30 mm). The volume of the hippocampus and lateral ventricle were analyzed with the Imaris software (Bitplane, Belfast, UK, version 9.0.1).

#### Electron microscopy

For electron microscopy, animals were quickly decapitated. The corpus callosum and hippocampus were dissected and shaped into trapezoidal sections. Tissues were then fixed in the electron microscope buffer at 4°C overnight, followed by ethanol dehydration and uranium-saturated solution with Leica EM TP (Leica, Germany). After being embedded with Spurr’s resin, ultra-structures of the corpus callosum and hippocampus were visualized and captured by using a transmission electron microscope Hitachi HT-7800 (Japan). The G-ratio of myelinated fiber was quantified by calculating the ratio of the axonal diameter to the myelinated axon diameter by using ImageJ win64 software. At least 100 myelinated axons were calculated and the number of synapses per high-power field (HPF) in each mouse was counted.

#### Alizarin red S staining

Mice were anesthetized and perfused with pre-cooled PBS, post-fixed in 4% paraformaldehyde (PFA), and then cryo-embedded. Brains were cut coronally into 15-μm-thick slices on a freezing microtome (Leica, CM1950). For alizarin red S staining, samples were incubated in alizarin red S solution for 10 min. The samples were then rinsed with 1×PBS, sealed with resin, and scanned with Leica Aperio Versa 200 to visualize brain calcification.

#### Immunofluorescent staining

Mouse brain sections (30 μm) were fixed with 4% paraformaldehyde for 30 min, washed with 1×PBS three times, treated with blocking buffer (5% BSA with 0.5% Triton X-100) at room temperature for 1 h, and then incubated with phos-NFH Antibody (1:200 dilution) or Iba1 antibody (1:200 dilution) overnight at 4°C. Brain sections were then stained with Alexa 488 (green) secondary antibodies (1:500) or Alexa 568 (red) conjugated secondary antibodies (1:500). All fluorescent images were obtained with an Olympus FV1000MPE-B confocal microscope (Japan). The number and the branches of Iba1^+^ microglia were counted by ImageJ-win64 software (Sholl analysis). The 3D structure of Iba1^+^ microglial morphology was reconstructed by the Imaris software (Bitplane, Belfast, UK, version 9.0.1).

#### NFH immunohistochemical staining

Immunohistochemical staining was performed using Biotin-labelled antibodies and an anti-streptomycin peroxidase kit (MXB, Kit-9270) according to the specification. Non-specific binding was blocked by incubating the sections with reagents A and B for ten minutes, respectively. Brain sections were then incubated overnight at 4°C with phos-NFH primary antibody. Sections were then rinsed with 1×PBS and incubated with biotinylated anti-rabbit IgG antibody at room temperature for 10 min (Reagent C). Sections were finally stained with Diaminobenzidine (DAB, MXB, DAB0031, 20 ×) at room temperature. Brain axonal spheroids were visualized and analyzed using Leica Aperio Versa 200.

#### Western blot

Primary microglia or brain tissues were lysed in RIPA buffer (LABLEAD) containing a 100×proteinase inhibitor cocktail (APE×BIO) and 100×phosphatase inhibitor cocktail (APE×BIO). The total proteins (20 μg for microglia lysates, 50 μg for brain lysates) quantified by BCA Assay kit (Abbkine) were loaded in 8% SDS-PAGE and transferred to 0.22 μm PVDF membranes (Millipore, IPVH00010). The membranes were sequentially incubated with primary or secondary antibodies. Proteins were then visualized by SuperKine ECL detection kit and the blots were quantified by Chemiluminescent Imaging System Azure 300 (USA).

#### RNA extraction and quantitative polymerase chain reaction (qPCR)

RNA was extracted from brain tissues or primary microglia using RNA isolater Total RNA Extraction Reagent (Vazyme). A total of one microgram RNA was reverse-transcribed into complementary DNA (cDNA) using HIScript III All-in-one RT SuperMix Perfect for qPCR (Vazyme). Target genes were amplified using HamQ Universal SYBR qPCR Master Mix (Vazyme) on the LightCycler 480 SYBR Green I Master (Roche, Mannheim, Germany). Fold change of the target gene mRNA level was calculated using the 2^−ΔΔCT^ method with *Actb* for internal control. The primer sequences are listed in Table S5.

#### Statistical analysis

Graphical and statistical analyses were performed in a double-blinded manner by GraphPad Prism software (San Diego, CA, USA, version 9.5.1). Distributed data are expressed as the mean ± SEM. The unpaired two-tailed Student’s *t* test was used for the comparison of two groups. One-way ANOVA post-Dunnett’s multiple comparisons test was used for the comparison of more than two groups. *p* value <0.05 was considered to be statistically significant.

## Supplemental information

Document S1. Figures S1–S9 and Tables S1–S5

Document S2. Article plus supplemental information
