# Supplementary material for: A strategy of microglia replacement alleviates microgliopathy in a CSF1R I794T hotspot mutation mouse model of CSF1R-related disorder
Source: Cell Rep Med. 2026 Feb 27;7(3):102641. doi: 10.1016/j.xcrm.2026.102641 (PMC13006430; doi:10.1016/j.xcrm.2026.102641)
Supplement: Document S1. Figures S1–S9 and Tables S1–S5 [file mmc1.pdf]

Cell Reports Medicine, Volume *7*

Supplemental information

A strategy of microglia replacement alleviates microgliopathy in a *CSF1R* I794T hotspot mutation mouse model of CSF1R-related disorder

Xin Li, Banglian Hu, Chujun Wu, Ziwei Wang, Hanzheng Fan, Xiaoyan Guan, Sulan Xie, Dadian Chen, Xiaohua Huang, Hao Sun, Yanfang Li, Xian Zhang, Guojun Bu, Zhanxiang Wang, Yun-Wu Zhang, Li Zhong, Zaiqiang Zhang, and Honghua Zheng

Supplementary Figures and figure legends

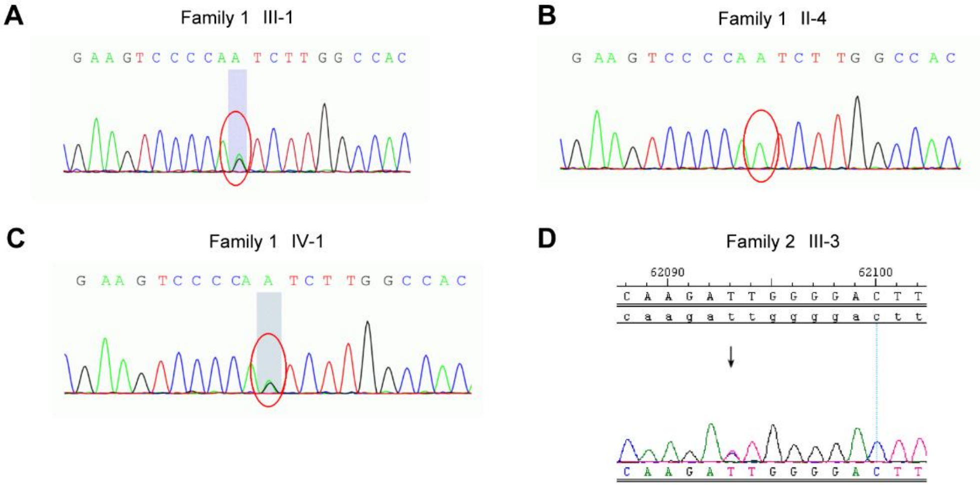

Figure S1. Heterozygous c2381 T>C (p.I794T) missense mutation of the CSF1R gene. Related to Figure 1.

DNA sequencing from four individuals, Family 1 III-1 (A), Family 1 II-4 (B, normal), Family 1 IV-1 (C), and Family 2 III-3 (D) revealed a heterozygous mutation (c2381 T>C/p.I794T) in the *CSF1R* gene. A-C, template strand; D, coding strand.

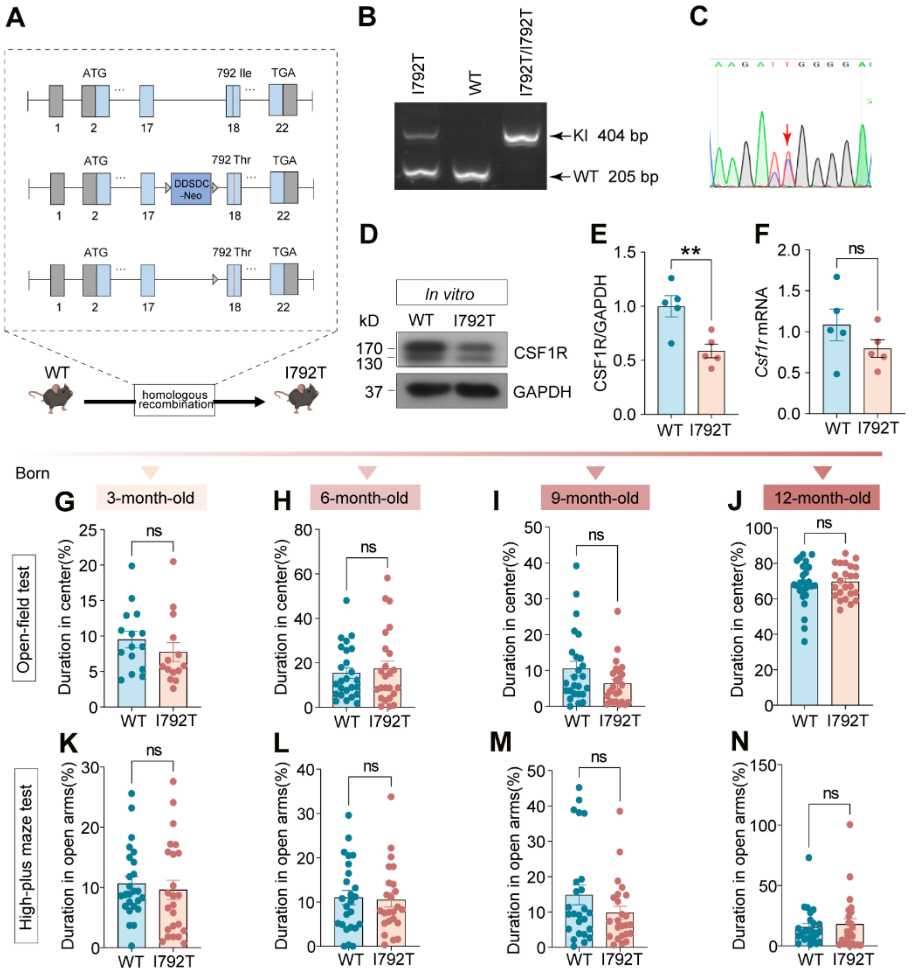

Figure S2. Generation and behavior evaluation of *Csf1r*$^{\mathrm{I792T/+}}$ mice. Related to Figure 2.

(A) Illustration of *Csf1r*$^{\mathrm{I792T/+}}$ mice generation using point mutation homologous recombination-based gene editing.

(B) Genotyping of WT, *Csf1r*$^{\mathrm{I792T/+}}$(I792T) and *Csf1r*$^{\mathrm{I792T/I792T}}$(I792T/I792T) mice by PCR. Wild-type (WT): 205 bp, I792T Knock-in (KI): 404 bp.

(C) DNA sequencing revealed a heterozygous mutation (p.I792T, indicated by a red arrow) in the *Csf1r* gene.

(D) Representative images of Western blot showing the expression of CSF1R in *Csf1r*$^{+/+}$ or *Csf1r*$^{\mathrm{I792T/+}}$ microglia.

(E) CSF1R protein levels quantified by densitometry with GAPDH for comparison, n=5 independent experiments per group, unpaired two-tailed Student's *t*-test.

(F) CSF1R mRNA levels quantified by quantitative PCR with GAPDH for comparison, n=5 independent experiments per group, unpaired two-tailed Student's *t*-test.

(G-J) Behavioral performances in 3-, 6-, 9-, 12-month-old *Csf1r*$^{+/+}$ or *Csf1r*$^{\mathrm{I792T/+}}$ mice assessed by open field test.

(K-N) Behavioral performances in 3-, 6-, 9-, 12-month-old *Csf1r*$^{+/+}$ or *Csf1r*$^{\mathrm{I792T/+}}$ mice assessed by elevated plus maze test. *Csf1r*$^{+/+}$, n =25; *Csf1r*$^{\mathrm{I792T/+}}$, n=24. Unpaired two-tailed Student's *t*-test. All data are presented as mean ± SEM. $^{**}p < 0.01$; ns, not significant.

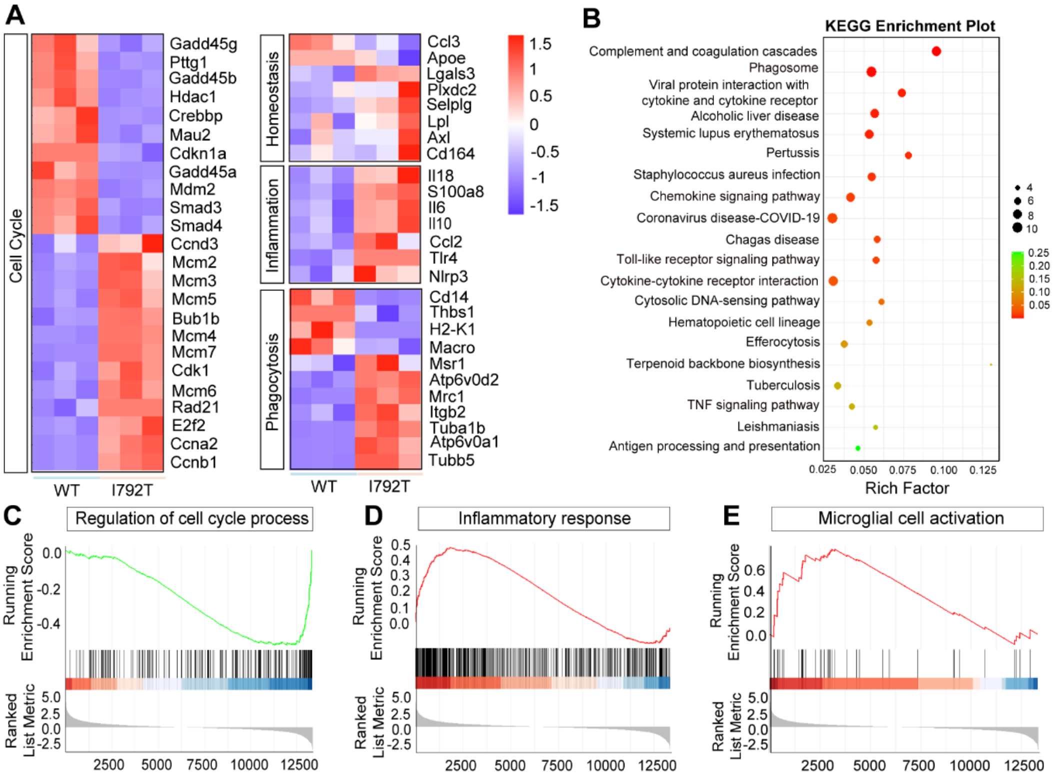

Figure S3. Newborn (0-3 day) *Csf1r* $^{\mathrm{I792T/+}}$ microglia show a phagocytic and proinflammatory phenotype. Related to Figure 4.

(A) Heatmap of differentially expressed genes (DEGs) from bulk RNA-seq of microglia isolated from *Csf1r*$^{+/+}$ or *Csf1r*$^{\mathrm{I792T/+}}$.

(B) Kyoto Encyclopaedia of Genes and Genomes (KEGG) pathway enrichment analysis of DEGs revealed the top twenty significantly altered pathways.

(C-E) Gene set enrichment analysis (GSEA) based on Gene Ontology Biological Process (GOBP) terms showed significant downregulation of cell cycle-related genes (C), and upregulation of genes involved in inflammatory response (D) and microglial activation (E).

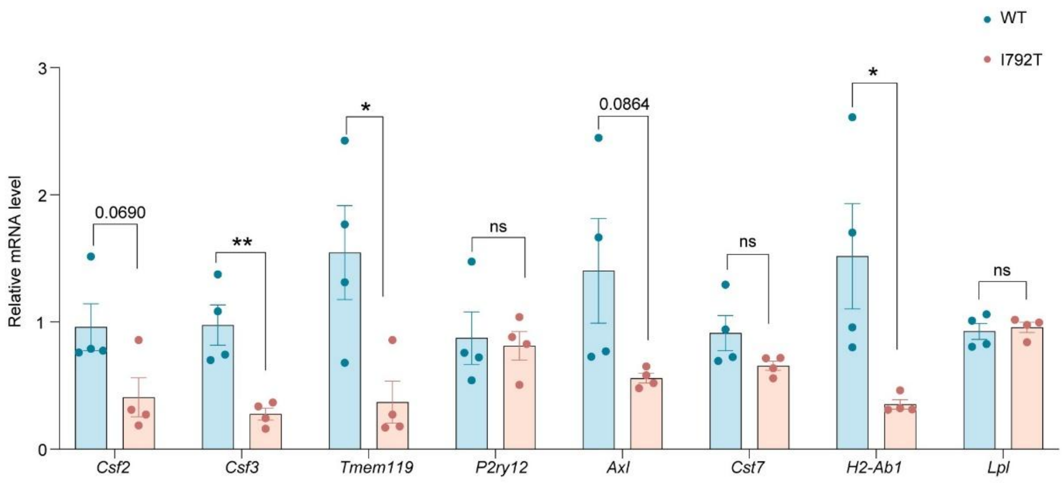

Figure S4. A phenotype of disease-associated microglia was observed in 9-month-old *Csf1r*$^{I792T/+}$ mouse brain. Related to Figure 4.

The mRNA levels of *Csf2*, *Csf3*, and genes indicating DAM including of *P2ry12*, *Tmem119*, *Axl*, *H2-Ab1*, *Lpl*, and *Cst7* were quantified by quantitative PCR. n=4 mice per group, data are presented as mean ± SEM, unpaired two-tailed Student's *t*-test. *p < 0.05; **p < 0.01; ns, not significant.

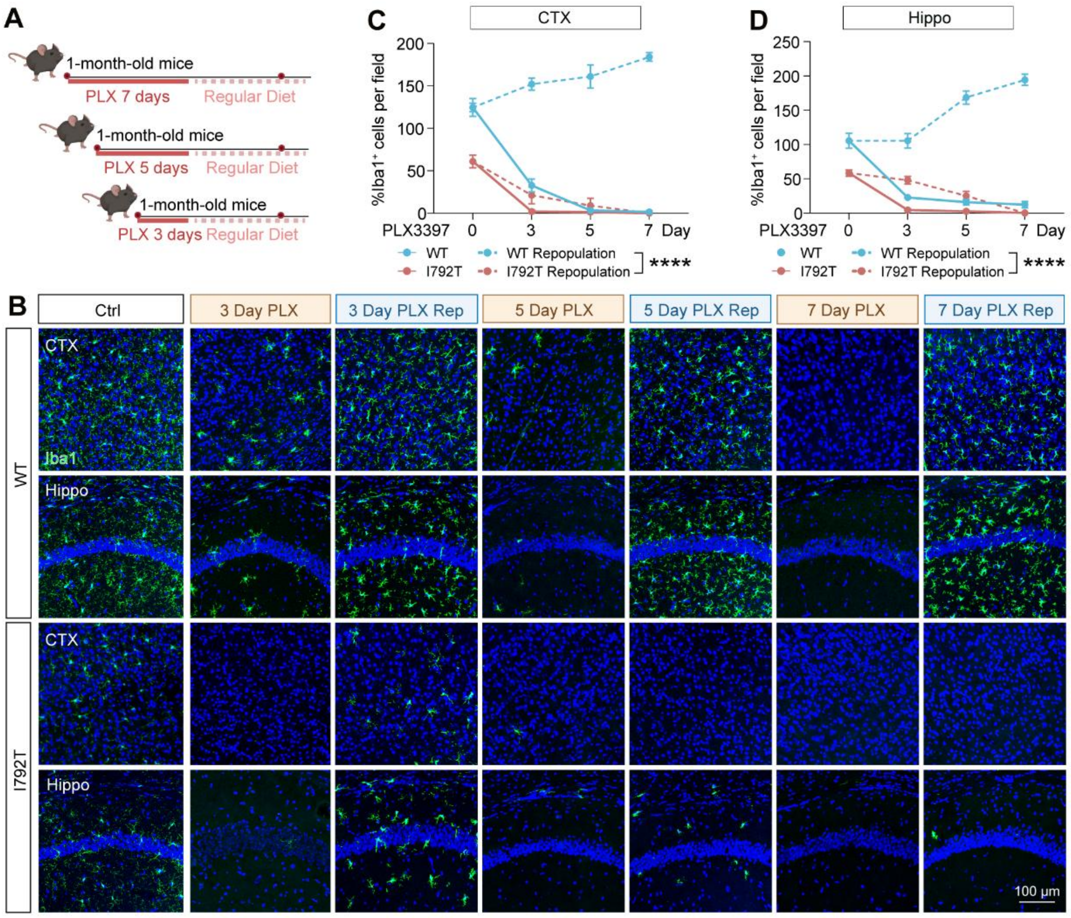

Figure S5. Determination of microglia transplantation window in *Csf1r* $^{I792T/+}$ mouse model. Related to Figure 5.

(A) Schematic diagram of *Csf1r* $^{I792T/+}$(I792T) microglia elimination strategy. One-month-old *Csf1r*$^{+/+}$ or *Csf1r*$^{I792T/+}$ male mice were orally treated with 600 mg/kg PLX3397 for three, five, or seven days, followed by microglia repopulation for one week (7-day regular diet). Mice were sacrificed and the brains were subjected to Iba1 immunofluorescent staining.

(B) Representative microglia (Iba1^{+}, green) immunofluorescent images of coronal brain slices from those one-month-old *Csf1r*$^{+/+}$ or *Csf1r*$^{I792T/+}$ male mice. Scale bar, 100 μm.

(C) The number of Iba1^{+} microglia was quantified in the cortex of those one-month-old *Csf1r*$^{+/+}$ or *Csf1r* $^{I792T/+}$ male mice.

(D) The number of Iba1^{+} microglia was quantified in the hippocampus of those one-month-old *Csf1r*$^{+/+}$ or *Csf1r*$^{I792T/+}$ male mice. One-way ANOVA post-Dunnett's multiple comparisons test. ****p < 0.0001. n=3 mice per group, data are presented as mean ± SEM.

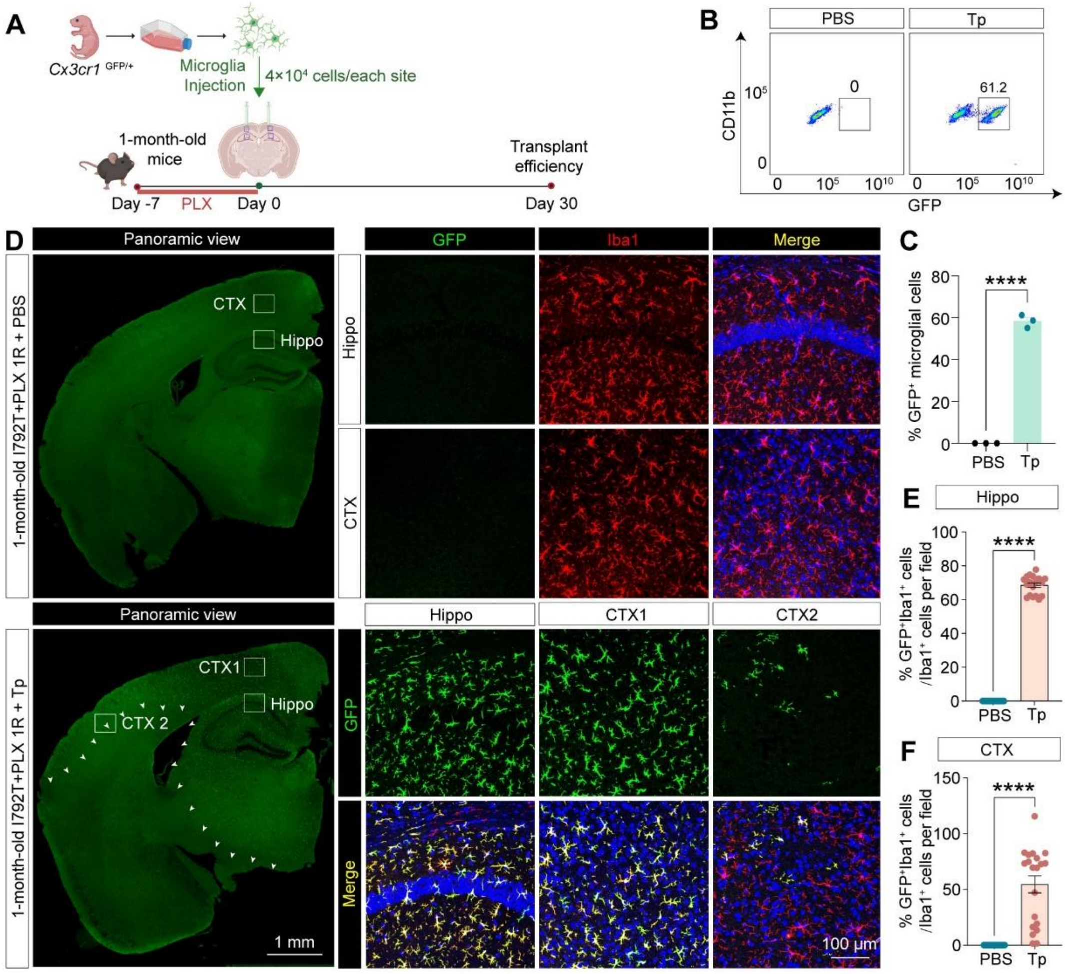

Figure S6. Determination of microglia transplantation efficiency in *Csf1r* $^{\mathrm{I792T/+}}$ mouse model. Related to Figure 5.

(A) Workflow of microglia elimination and transplantation strategy. One-month-old *Csf1r*$^{+/+}$ or *Csf1r*$^{\mathrm{I792T/+}}$ male mice were orally treated with 600 mg/kg PLX3397 for seven days, followed by microglia repopulation for one week. Mice *Cx3cr1*$^{\mathrm{GFP/+}}$ microglia were then transplanted into the brain of those mice. Mice were sacrificed one month later and the brains were subjected to Iba1 immunofluorescent staining (red).

(B) Representative dot plots of GFP$^+$Iba1$^+$ microglia isolated from *Csf1r* $^{\mathrm{I792T/+}}$ mouse brain transplanted with GFP$^+$Iba1$^+$ microglia or PBS via flow cytometry.

(C) The percentage of GFP$^+$Iba1$^+$ microglia relative to the total Iba1$^+$ microglia was only up to 61.2% in *Csf1r* $^{\mathrm{I792T/+}}$ mouse brain transplanted with GFP$^+$ microglia.

(D) Representative GFP$^+$Iba1$^+$ microglia immunofluorescent images of coronal brain slices of two-month-old *Csf1r*$^{+/+}$ or *Csf1r*$^{\mathrm{I792T/+}}$ male mice, one month post- intracerebral injection with PBS, or *Cx3cr1*$^{\mathrm{GFP/+}}$ microglia (Tp).

(E) Quantification of the percentage of GFP$^+$Iba1$^+$ microglia relative to Iba1$^+$ cells per field in the hippocampus of *Csf1r*$^{\mathrm{I792T/+}}$ mouse brain.

(F) Quantification of the percentage of GFP$^+$Iba1$^+$ microglia relative to Iba1$^+$ cells per field in the cortex of *Csf1r*$^{\mathrm{I792T/+}}$ mouse brain. Unpaired two-tailed Student's *t*-test. n=3 mice per group, data are presented

as mean ± SEM. ****$p < 0.0001$.

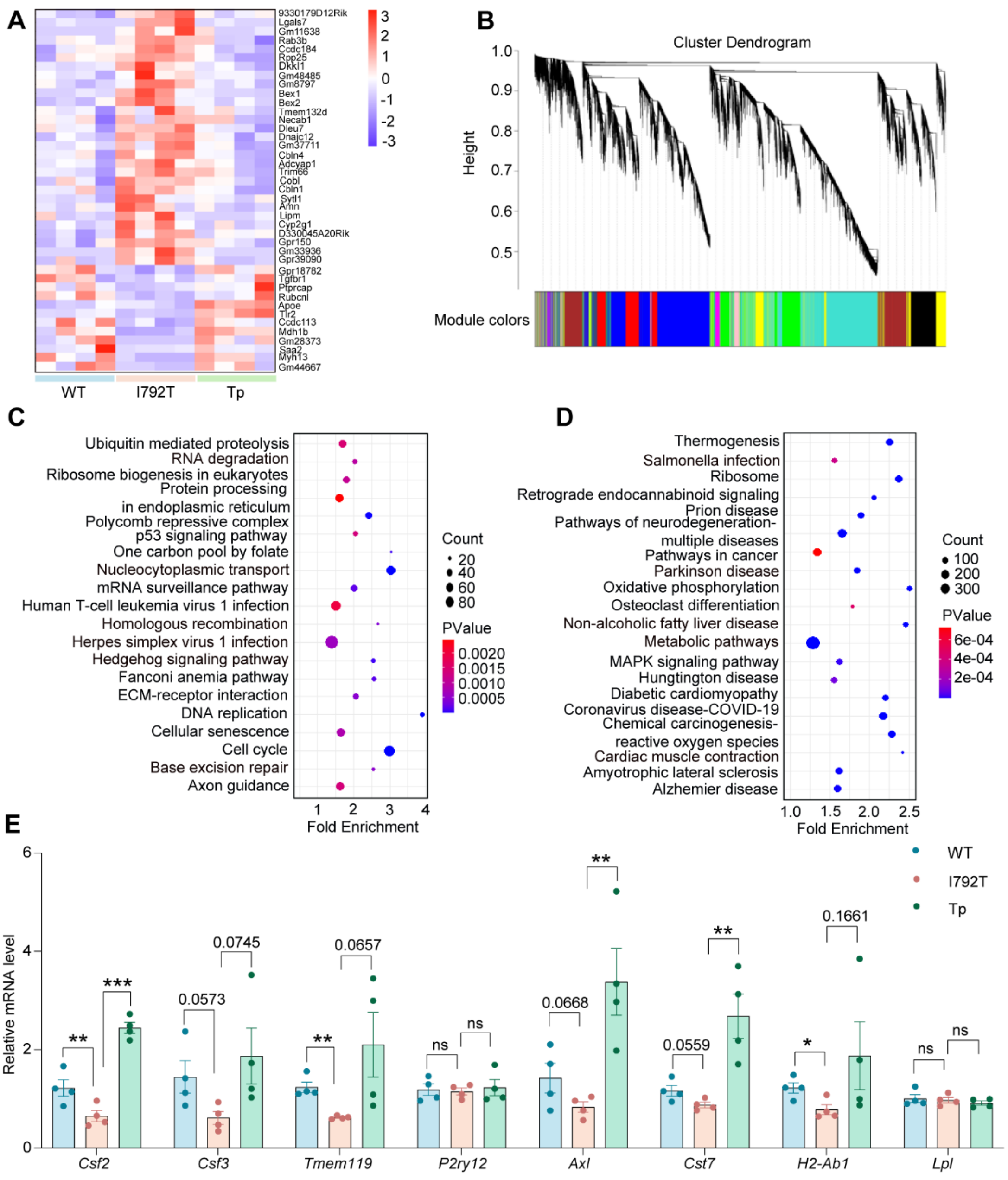

Figure S7. The gene sets in *Csf1r*$^{I792T/+}$ mice transplanted with microglia are similar to those in *Csf1r*$^{+/+}$ mice. Related to Figure 7.

(A) The heatmap of differentially expressed genes (DEGs) in bulk RNA-seq from *Csf1r*$^{+/+}$ brain, *Csf1r* $^{I792T/+}$ brain with or without microglial transplantation. Genes related to disease-associated microglia are highlighted in red.

(B) WGCNA in the forebrain of *Csf1r*$^{+/+}$ brain, *Csf1r* $^{I792T/+}$ brain with or without microglial transplantation. Color bands show gene modules obtained by automatic single-block analysis.

(C-D) KEGG plots of DEGs in the two most representative modules in the WGCNA dendrogram. (C) The top twenty high-confidence KEGG terms in the blue module are shown. (D) The top twenty high-confidence KEGG terms in the turquoise module are shown.

(E) The transcriptional levels of *Csf2/3* and DAM genes including *Tmem119*, *Axl*, *Cst7* in those mice

brains were quantified by RT-qPCR. Indicated decreased genes (*Csf2/3*, *Tmem119*, *Axl*) in *Csf1r*$^{I792T/+}$ brain were reversed by microglial transplantation. WT, *Csf1r*$^{+/+}$ mice; I792T, *Csf1r*$^{I792T/+}$ mice; Tp, *Csf1r*$^{I792T/+}$ mice transplanted with microglia; WGCNA, weighted gene co-expression network analysis; KEGG, Kyoto Encyclopedia of Genes and Genomes. n=4 mice per group, data are presented as mean ± SEM. One-way ANOVA post-Dunnett's multiple comparisons test. *$p < 0.05$; **$p < 0.01$; ***$p < 0.001$; ns, not significant.

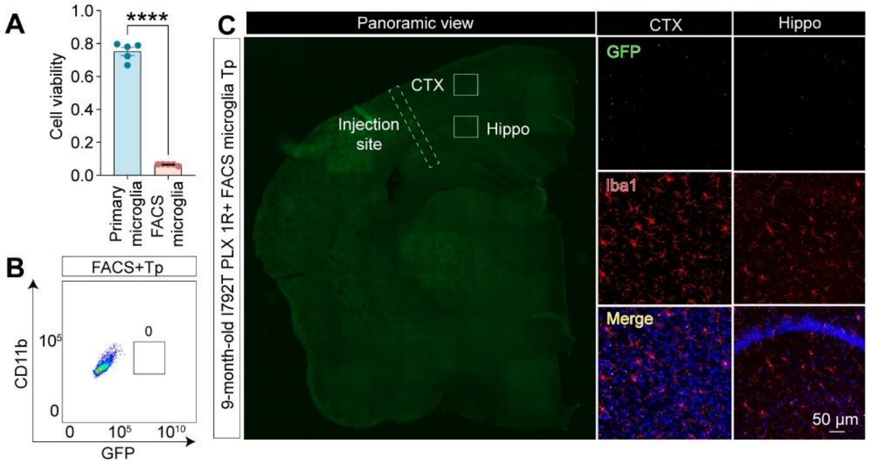

Figure S8. Isolated microglia from P1 mice via fluorescence-activated cell sorting (FACS) were unsuitable for transplantation experiments. Related to Figures 5-7.

(A) The sorted microglia population exhibited low viability in CCK8 assays compared with primary cultured microglia.

(B) GFP^+CD11b^+ microglia were isolated from P1 *Cx3cr1*$^{GFP/+}$ mice via FACS and these microglia were transplanted into *Csf1r* $^{I792T/+}$ mouse brain. One month later, the brains were dissected and no GFP^+CD11b^+ microglia were isolated from those transplanted *Csf1r* $^{I792T/+}$ mouse brain via flow cytometry.

(C) No GFP^+Iba1^+ microglia were observed in the immunofluorescent images of coronal brain slices from those *Csf1r*$^{I792T/+}$ mice, one month post- intracerebral injection with isolated GFP^+Iba1^+ microglia. Unpaired two-tailed Student's t-test. n=5 experiments or mice per group, data are presented as mean ± SEM. ****$p < 0.0001$.

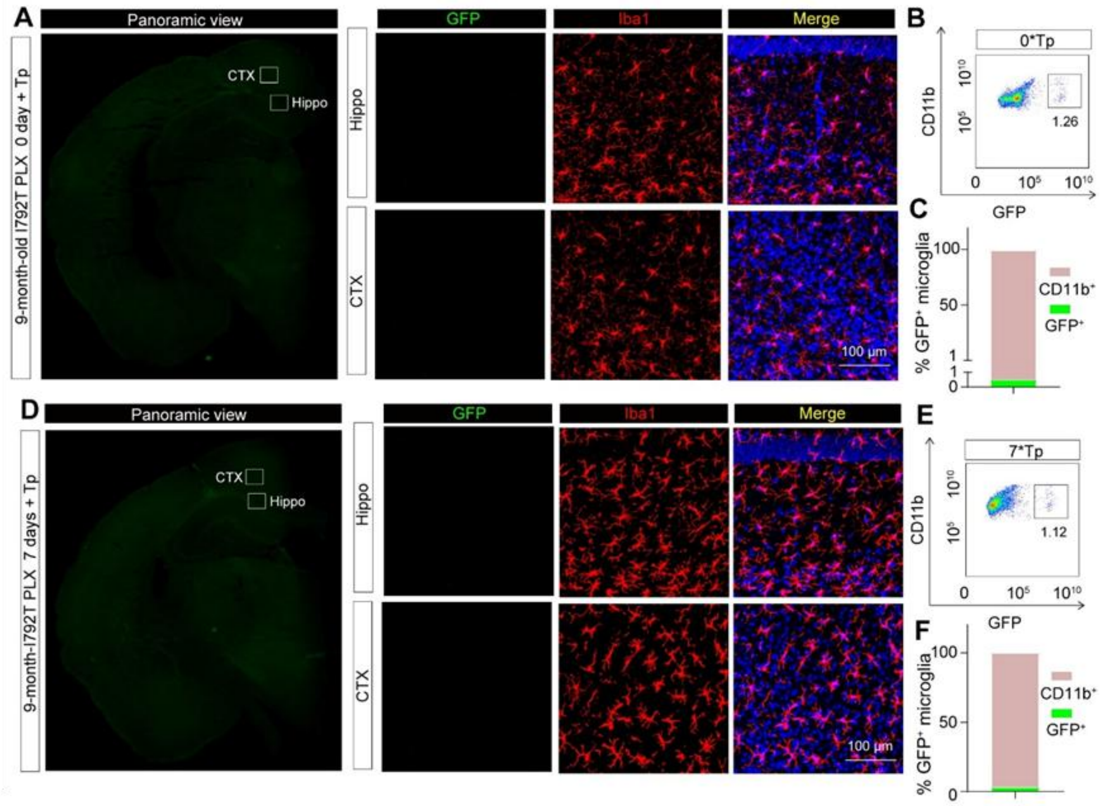

Figure S9. Transplanted GFP$^+$Iba1$^+$ microglia failed to localized into brains of 9-month-old *Csf1r*$^{I792T/+}$ mice with no microglial depletion or with single microglial depletion. Related to Figures 5-7.

(A) GFP$^+$Iba1$^+$ microglia were transplanted into *Csf1r* $^{I792T/+}$ mice without microglial depletion. One month later, the brains were dissected and few GFP$^+$Iba1$^+$ microglia were observed in the immunofluorescent images of coronal brain slices from those *Csf1r*$^{I792T/+}$ mice.

(B-C) Few GFP$^+$CD11b$^+$ microglia were isolated from those transplanted *Csf1r*$^{I792T/+}$ mouse brain without microglial depletion via flow cytometry.

(D) GFP$^+$Iba1$^+$ microglia were transplanted into *Csf1r*$^{I792T/+}$ mice with single microglial depletion. One month later, the brains were dissected and few GFP$^+$Iba1$^+$ microglia were observed in the immunofluorescent images of coronal brain slices from those *Csf1r*$^{I792T/+}$ mice.

(E-F) Few GFP$^+$CD11b$^+$ microglia were isolated from those transplanted *Csf1r*$^{I792T/+}$ mouse brain with single microglial depletion via flow cytometry.

Data are represented as mean ± SEM. 0*Tp: GFP$^+$Iba1$^+$ microglia were transplanted into *Csf1r*$^{I792T/+}$ mice without microglial depletion; 7*Tp: GFP$^+$Iba1$^+$ microglia were transplanted into *Csf1r*$^{I792T/+}$ mice with single microglial depletion.

Table S1. Clinical details of CSF1R-RD patients with CSF1R p.I794T variants. Related to Figure 1.

Patient	Clinically recorded gender	Ethnicity	AAO	AAE	AAD	Disease duration (years)
1 (This study)	M	Chinese	35	36	39	4
2 (This study)	M	Chinese	27	28	Alive	Alive
3 (This study)	M	Chinese	39	42	Alive	Alive
4	F	Chinese	32	34	Alive	Alive
5	F	Chinese	41	NA	46	5
6	M	Chinese	37	NA	39	2
7	M	Chinese	43	NA	Alive	Alive
8	M	Chinese	42	43	Alive	3/Alive
9	F	Chinese	42	44	Alive	2/Alive
10	F	Chinese	35	37	Alive	2/Alive
11	M	Chinese	37	38	Alive	1/Alive
12	M	Chinese	39	39	Alive	1/Alive
13	F	Chinese	35	37	Alive	2/Alive
14	F	Chinese	42	43	Alive	1/Alive
15	F	Chinese	41	42	Alive	14M/Alive
16	M	Chinese	37	38	Alive	1/Alive
17	F	Chinese	41	43	Alive	2/Alive
18	M	Chinese	46	46+4M	Alive	4M/Alive
19	F	Chinese	NA	46	Alive	NA
20	F	Chinese	35	37	Alive	2/Alive
21	M	Chinese	40	42	Alive	2/Alive
22	M	Chinese	51	53	Alive	2/Alive
23	M	Chinese	60	62	Alive	2/Alive
24	F	Chinese	42	43	Alive	1/Alive
25	M	Chinese	31	31	Alive	1/Alive
26	M	Japanese	60	61	Alive	8M/Alive
27	M	Japanese	46	48	Alive	2/Alive
28	F	Japanese	33	36	Alive	3/Alive
29	M	Japanese	49	50	Alive	1/Alive
30	F	Japanese	34	35	Alive	1/Alive
31	F	Japanese	60	70	Alive	Alive
32	M	Japanese	40	46	Alive	6/Alive
33	F	Japanese	55	NA	Alive	14/Alive
34	F	Japanese	28	28+6M	Alive	6M/Alive
35	F	Japanese	27	NA	Alive	Alive
36-41	7NA	Japanese	NA	NA	NA	NA
42	F	Japanese	20	26	Alive	6/Alive

43	F	Japanese	20	20+5M	Alive	5M/Alive
44	M	Japanese	40	46	Alive	6/Alive
45	M	Japanese	40	42	Alive	2/Alive
46	M	Korean	51	55	Alive	4/Alive
47	M	Korean	60	61	Alive	1/Alive
48	F	Korean	51	55	Alive	4/Alive
49	F	Korean	60	61	Alive	1/Alive
50	M	Korean	51	52	Alive	1/Alive
51	M	Indian	40	40+9M	Alive	9M/Alive
52	F	Indian	33	34	Alive	1/Alive
53	F	American	33	36	Alive	3/Alive
54	M	American	43	56	Alive	13/Alive
55	F	American	37	NA	40	3
56	F	American	35	36	Alive	Alive
57	NA	American	35	NA	Alive	Alive
58	M	American	60	63	Alive	3/Alive
59	M	French	57	5	NA	NA
60	M	French	28	32	NA	NA
61	F	French	28	4	NA	NA
62	F	Germany	40	NA	43	3
63	F	Germany	29	29+4M	Alive	4M/Alive
64	M	British	52	NA	Alive	Alive
65	M	The Netherlands	46	NA	49	3
66	M	Ireland	41	41+4M	Alive	4M/Alive

Abbreviations: CSF1R-RD = CSF1R-related disorder; AAO = Age at onset(Y); AAE = Age at examination(Y); AAD = Ag
patients were unable to participate in the test.

Cognitive performance		Family history	Initial symptoms	Cognitive impairment	Psychiatric symptoms	Parkinsonism
MMSE	MoCA					
NA	24	+	Cognitive impairment	+	-	-
NA	NA	+	Spastic hemiplegia	-	-	-
3	NA	+	Psychiatric symptoms	+	+	-
23	NA	+	NA	+	+	-
NA	NA	+	Cognitive impairments	+	NA	+
NA	NA	+	Cognitive impairment	+	NA	-
27	23	-	Walking difficulties	-	NA	-
14	12	+	Depression	+	+	-
15	NA	+	Cognitive impairments, gait dysfunction	+	+	+
15	19	+	Dysarthria, gait dysfunction	+	+	+
NA	NA	+	Cognitive decline, gait dysfunction	+	+	-
28	25	+	Memory loss	+	-	-
19	15	NA	Cognitive impairments, gait dysfunction, slurred speech	+	NA	+
15	NA	NA	mpairments, gait [illegible]	+	NA	-
NA	NA	-	NA	+	-	-
NA	NA	+	NA	+	+	-
15	13	+	Apathy	+	+	-
NA	NA	NA	Personality change	NA	+	NA
NA	NA	NA	NA	NA	NA	NA
19	15	NA	NA	+	+	+
28	25	NA	NA	+	+	-
21	14	NA	NA	+	-	+
15	12	NA	NA	+	+	-
NA	NA	-	Apathy, gait dysfunction, urinary incontinence, memory loss	+	+	-
NA	23	NA	NA	+	-	-
29	27	+	Gait dysfunction	-	-	-
NA	NA	+	Depression	+	+	-
NA	NA	+	Gait dysfunction	+	-	+
NA	NA	+	Aphasia	+	-	-
NA	NA	-	Cognitive impairment, depression	+	+	+
NA	NA	+	Forgetfulness	+	+	-
NA	NA	-	Cognitive impairment	+	+	-
NA	NA	+	Cognitive impairment	+	+	+
14	NA	-	Gait dysfunction,	+	NA	-
NA	NA	-	NA	+	+	NA
NA	NA	NA	NA	NA	NA	NA
NA	NA	NA	Cognitive impairment	NA	NA	NA

30	NA	+	Progressive right spastic hemiplegia	-	+	-
NA	NA	-	Cognitive impairments	+	-	-
NA	NA	NA	NA	NA	NA	NA
NA	NA	NA	NA	+	+	+
NA	NA	NA	NA	+	+	-
NA	NA	-	Cognitive impairment	+	+	-
NA	NA	-	Cognitive impairment, gait dysfunction	+	-	+
NA	NA	-	Personality change	+	+	+
NA	NA	-	Cognitive impairments	+	+	-
NA	NA	NA	Progressive apathy, executive dysfunction, and memory	-	+	-
NA	NA	-	Weakness in the right hand	+	-	-
NA	NA	-	Apathy	+	NA	-
NA	NA	+	Cognitive impairments	+	+	-
NA	NA	+	Cognitive impairments	+	+	+
NA	NA	NA	Cognitive impairments	+	NA	±
NA	NA	+	Gait dysfunction	+	+	-
NA	NA	+	Slurred speech	NA	NA	NA
NA	NA	+	Gait dysfunction	NA	NA	NA
NA	NA	-	Gait dysfunction	NA	NA	NA
NA	NA	+	Personality change, apathy, clumsiness	NA	+	NA
NA	NA	-	ysarthria and cogı	+	-	-
NA	NA	-	Cognitive impairments	+	NA	+
NA	NA	-	NA	+	-	-
NA	26	-	Aphasia and bifrontal headache	+	-	+

e at death(Y); MMSE = Mini Mental Status Examination; MoCA = Montreal Cognitive Assessment; + means positive; – me

Ataxia	Pyramidal signs	Epilepsy	Ref.
-	+	NA	This study
-	+	NA	This study
-	+	NA	This study
+	+	-	1
NA	-	-	2
NA	+	-	2
NA	+	-	2
-	-	-	3
NA	NA	-	4
NA	NA	-	4
NA	NA	-	4
NA	NA	-	4
+	NA	-	5
NA	NA	-	5
-	+	-	6
NA	NA	-	7
-	-	+	8
NA	NA	NA	9
NA	NA	NA	10
+	NA	NA	11
-	NA	NA	11
-	NA	NA	11
+	NA	NA	11
-	-	-	12
-	-	-	13
-	-	-	14
NA	-	NA	15
NA	-	NA	15
NA	-	NA	15
NA	-	NA	15
NA	NA	-	16
NA	+	+	17
NA	+	+	17
+	+	-	18
NA	+	+	18
NA	NA	NA	19
NA	NA	NA	20

-	+	-	21
-	+	-	22
NA	NA		23
NA	NA	+	24
-	-	+	24
NA	-	-	25
NA	-	+	25
NA	-	+	25
-	-	-	26
NA	NA	NA	27
-	+	-	28
NA	+	+	28
-	-	-	29
NA	NA	+	30
NA	+	-	31
-	NA	-	32
NA	NA	NA	33
NA	NA	NA	33
NA	NA	NA	33
NA	+	+	34
+	+	-	35
NA	+	NA	36
-	-	-	37
-	+	-	38

eans negative; ± means probable; NA = not available, because the

Table S2. Imaging details of CSF1R-RD patients with CSF1R p.I794T variants. Related to Figure 1.

Patient	Clinically recorded gender	Ethnicity	White matter lesions	Dilated lateral ventricles	Corpus callosum atrophy	Cortical atrophy predominantly in frontal/parietal lobes
1 (This study)	M	Chinese	+	+	+	NA
2 (This study)	M	Chinese	+	+	+	NA
3 (This study)	M	Chinese	+	+	+	NA
4	F	Chinese	+	-	-	+
5	F	Chinese	+	-	+	+
6	M	Chinese	+	-	+	+
7	M	Chinese	+	-	+	+
8	M	Chinese	+	+	NA	+
9	F	Chinese	+	NA	+	-
10	F	Chinese	+	NA	+	+
11	M	Chinese	+	NA	+	+
12	M	Chinese	+	NA	-	-
13	F	Chinese	+	+	+	+
14	F	Chinese	+	+	+	+
15	F	Chinese	+	NA	NA	NA
16	M	Chinese	+	-	-	+
17	F	Chinese	+	-	+	+
18	M	Chinese	NA	NA	+	NA
19	F	Chinese	NA	NA	NA	NA
20	F	Chinese	NA	NA	NA	+
21	M	Chinese	NA	NA	NA	+
22	M	Chinese	NA	NA	NA	+
23	M	Chinese	NA	NA	NA	+
24	F	Chinese	+	NA	NA	NA
25	M	Chinese	+	-	+	+
26	M	Japanese	+	+	+	+
27	M	Japanese	±	+	-	NA
28	F	Japanese	+	+	+	NA
29	M	Japanese	+	+	+	NA
30	F	Japanese	+	+	+	NA
31	F	Japanese	+	NA	NA	+
32	M	Japanese	+	NA	+	NA
33	F	Japanese	+	NA	+	NA
34	F	Japanese	+	NA	+	+
35	F	Japanese	NA	NA	+	+
36-41	7NA	Japanese	NA	NA	NA	NA
42	F	Japanese	NA	NA	NA	NA
43	F	Japanese	+	-	NA	+
44	M	Japanese	+	+	+	+
45	M	Japanese	+	+	+	NA
46	M	Korean	+	+	+	+
47	M	Korean	+	+	+	+
48	F	Korean	NA	NA	NA	NA
49	F	Korean	NA	NA	NA	NA
50	M	Korean	NA	NA	NA	NA
51	M	Indian	+	+	+	NA
52	F	Indian	+	-	+	NA
53	F	American	+	NA	-	NA
54	M	American	+	NA	+	NA
55	F	American	+	+	+	+
56	F	American	+	+	NA	+
57	NA	American	NA	NA	NA	NA
58	M	American	+	NA	+	+
59	M	French	NA	NA	NA	NA
60	M	French	NA	NA	NA	NA

61	F	French	NA	NA	NA	NA
62	F	Germany	+	NA	+	NA
63	F	Germany	+	NA	NA	NA
64	M	British	NA	NA	NA	NA
65	M	The Netherlands	+	-	-	NA
66	M	Ireland	+	-	NA	+

Abbreviations: DWI = Diffusion weighted imaging, CT = Computed Tomography, + means positive, – means negative, NA [illegible]
test

Punctate DWI hyperintensities	White matter calcifications (on CT scan)	Fulfilling diagnostic criteria according to Konno et al.[39]	Ref.
+	NA	Probable	This study
+	-	Probable	This study
+	NA	Probable	This study
+	+	Probable	1
+	NA	Probable	2
-	-	Probable	2
+	+	Probable	2
+	NA	Probable	3
NA	-	Probable	4
NA	-	Probable	4
NA	-	Probable	4
NA	-	Probable	4
NA	NA	Probable	5
NA	NA	Probable	5
+	+	Probable	6
NA	NA	Probable	7
+	NA	Probable	8
-	NA	Probable	9
NA	NA	Probable	10
NA	NA	Probable	11
NA	NA	Probable	11
NA	NA	Probable	11
NA	NA	Probable	11
+	NA	Probable	12
+	NA	Probable	13
NA	-	Probable	14
-	+	Possible	15
-	+	Probable	15
-	-	Possible	15
+	+	Probable	15
NA	NA	Probable	16
NA	+	Probable	17
NA	NA	Probable	17
NA	NA	Probable	18
NA	NA	Probable	18
NA	NA	Probable	19
NA	NA	Probable	20
+	NA	Probable	21
NA	+	Probable	22
NA	+	Probable	23
NA	NA	Probable	24
NA	NA	Probable	24
NA	NA	Probable	25
NA	NA	Probable	25
NA	NA	Probable	25
-	NA	Probable	26
+	NA	Probable	27
+	+	Probable	28
+	NA	Probable	28
NA	NA	Probable	29
NA	NA	Probable	30
NA	NA	Probable	31
NA	NA	Probable	32
NA	NA	Probable	33
NA	NA	Probable	33

NA	NA	Probable	33
NA	NA	Probable	34
NA	+	Probable	35
NA	NA	Probable	36
NA	NA	Probable	37
+	NA	Probable	38

[illegible] = not available, because the patients were unable to participate in the

Table S3. Age of onset and disease duration in non-I794T patients. Related to Figure 1.

No.	Cases	Protein domain	CSF1R	AO	cally recorded ge	Duration
1	1	Signal peptide	p.G17C	25	M	6
	2		p.G17C	NA	NA	NA
2	3	EC	p.P54Q	51	F	NA
3	4	EC	p.S66G	NA	F	NA
4	5	EC	p.A74T	58	F	10/Alive
5	6	EC	p.T79M	86	M	1
6	7	EC	p.A96Pfs*17	NA	NA	NA
7	8	EC	p.P104Lfs*8	22	F	2/Alive
8	9	EC	p.P132L	NA	M	5/Alive
9	10	EC	p.Q176*	55	F	1/Alive
10	11	EC	p.H239fs	49	F	4/Alive
11	12	EC	p.C278*	47	F	2/Alive
12	13	EC	p.V279M	14	M	8/Alive
13	14	EC	p.C653Y	48	F	15
	15		p.C653Y	48	F	15
14	16	EC	p.H362R	60	M	NA
14	17		p.H362R	51	M	NA
14	18		p.H362R	56	M	NA
14	19		p.H362R	51	F	NA
14	20		p.H362R	41	F	NA
14	21		p.H362R	60	F	NA
14	22		p.H362R	25	M	4/Alive
15	23	EC	p.T363N	45	M	5/Alive
16	24	EC	p.V383L	45	M	9M/Alive
17	25	EC	p.E403Rfs*37	47	F	3/Alive
18	26	EC	p.L418X	68	F	5/Alive
19	27	EC	p.Q426fs	54	F	1/Alive
20	28	EC	p.Q433*	22	F	5/Alive
21	29	EC	p.E478K	NA	NA	NA
22	30	EC	p.Q481*	NA	M	5/Alive
23	31	EC	p.T507_H508InsP	NA	NA	NA
24	32	TM	p.L536V	55	F	NA
25	33	JMD	p.Y540*	NA	NA	NA
26	34	JMD	p.R549H	64	NA	NA
27	35	JMD	p.D565N	NA	NA	NA
28	36	JMD	p.T567fs*44	24	F	29
29	37	JMD	p.T567M	34	F	NA
30	38	JMD	p.E573K	NA	M	NA
30	39		p.E573K	78	NA	NA
31	40	JMD	p.R579Q	54	F	NA
31	41		p.R579Q	41	M	14M/Alive
32	42	JMD	p.R579W	39	M	2
33	43	JMD	p.N581Kfs	42	F	3/Alive
34	44	TKD1	p.L582P	45	M	9M
34	45		p.L582P	NA	M	NA
35	46	TKD1	p.G585_K619delinsA	36	F	4
36	47	TKD1	p.K586*	79	M	3
37	48	TKD1	p.G589E	60	M	10
37	49		p.G589E	58	M	3
37	50		p.G589E	47	F	11

37	51		p.G589E	58	M	2/Alive
37	52		p.G589E	47	F	5/Alive
38	53	TKD1	p.G589R	48	F	NA
38	54		p.G589R	37	F	NA
38	55		p.G589R	40	M	NA
38	56		p.G589R	27	F	30M/Alive
38	57		p.G589R	37	F	4/Alive
38	58		p.G589R	44	F	3/Alive
38	59		p.G589R	43	F	3/Alive
38	60		p.G589R	30	M	3/Alive
38	61		p.G589R	39	F	1/Alive
38	62		p.G589R	NA	NA	NA
38	63		p.G589R	NA	NA	NA
38	64		p.G589R	NA	F	NA
38	65		p.G589R	NA	F	NA
39	66	TKD1	p.E596M	25	F	10
40	67	TKD1	p.E598del	43	F	1/Alive
41	68	TKD1	p.A601P	29	F	2/Alive
42	69	TKD1	p.V613L	51	F	NA
43	70	TKD1	p.V613M	46	M	2/Alive
43	71		p.V613M	51	F	3/Alive
44	72	TKD1	p.L618del	39	F	1/Alive
45	73	TKD1	p.S620delins40	28	F	11/Alive
46	74	TKD1	p.K627del	28	F	11/Alive
47	75	TKD1	p.L630R	45	M	7
48	76	TKD1	p.E633K	44	F	6M/Alive
48	77		p.E633K	46	F	8
48	78		p.E633K	42	F	4
48	79		p.E633K	41	F	4/Alive
48	80		p.E633K	29	NA	NA
48	81		p.E633K	NA	F	NA
48	82		p.E633K	73	M	2/Alive
49	83	TKD1	p.L634R	53	F	NA
50	84	TKD1	p.I636N	29	M	2
51	85	TKD1	p.H643Q	12	M	12/Alive
52	86	TKD1	p.V647del	35	M	0.75/Alive
53	87	TKD1	p.G651E	40	M	3M/Alive
53	88		p.G651E	45	F	1/Alive
53	89	TKD1	p.G651E	40	M	3/Alive
54	90	TKD1	p.G651R	40	F	1/Alive
55	91	TKD1	p.A652P	30	F	1/Alive
55	92		p.A652P	NA	NA	NA
56	93	TKD1	p.C653R	43	F	5/Alive
57	94	TKD1	p.P658Sfs*24	NA	M	NA
58	95	TKD1	p.L660P	47	M	2/Alive
59	96	TKD1	p.I662T	40	M	6/Alive
60	97	TKD1	p.E664K	35	F	NA
61	98	TKD1	p.C666R	37	M	12/Alive
62	99	TKD1	p.D670N	53	F	4/Alive
63	100	TKD1	p.R676*	30	F	2
63	101		p.R676*	31	F	1
63	102		p.R676*	30	F	2
64	103	KID	p.S688Efs*13	41	F	13

65	104	KID	p.Q691H	82	F	7
66	105	KID	p.E694K	NA	NA	NA
67	106	KID	p.H703Y	79	NA	3
68	107	KID	p.R710H	NA	NA	NA
69	108	KID	p.G747R	NA	NA	NA
70	109	TKD2	p.L755P	27	F	31M
71	110	TKD2	p.L756P	34	M	16M/Alive
72	111	TKD2	p.F758S	23	F	NA
73	112	TKD2	p.S759F	55	M	7
73	113		p.S759F	NA	NA	NA
73	114		p.S759F	NA	NA	NA
74	115	TKD2	p.A763P	45	M	6
75	116	TKD2	p.G765C	NA	NA	NA
76	117	TKD2	p.G765D	44	M	3/Alive
76	118		p.G765D	37	F	5/Alive
76	119		p.G765D	NA	NA	NA
77	120	TKD2	p.G765S	33	M	1/Alive
78	121	TKD2	p.M766T	40	F	10
78	122		p.M766T	NA	F	NA
78	123		p.M766T	18	F	NA
78	124		p.M766T	18	F	12/Alive
78	125		p.M766T	46	M	3/Alive
79	126	TKD2	p.M766V	39	M	NA
80	127	TKD2	p.F768L	40	M	3/Alive
81	128	TKD2	p.A770P	52	M	11
81	129		p.A770P	NA	NA	NA
82	130	TKD2	p.C774_N814del	50	M	5
83	131	TKD2	p.C774_N814delinsQGLQSHVGPSLPSSSPQAQ	23	F	NA
84	132	TKD2	p.I775N	48	M	NA
85	133	TKD2	p.R777Q	60	F	5
85	134		p.R777Q	21	F	18M
85	135		p.R777Q	24	M	8/Alive
85	136		p.R777Q	38	F	7/Alive
85	137		p.R777Q	40	F	1M/Alive
85	138		p.R777Q	40	F	24/Alive
85	139		p.R777Q	41	F	3
85	140		p.R777Q	43	F	4/Alive
85	141		p.R777Q	33	F	1/Alive
85	142		p.R777Q	22	F	1/Alive
85	143		p.R777Q	33	F	1/Alive
86	144	TKD2	p.R777W	46	F	6
86	145		p.R777W	52	M	4/Alive
86	146		p.R777W	38	M	NA
86	147		p.R777W	40	M	NA
86	148		p.R777W	53	M	56
86	149		p.R777W	NA	NA	NA
86	150		p.R777W	NA	NA	NA
87	151	TKD2	p.D778E	60	F	6/Alive
88	152	TKD2	p.V779M	NA	NA	NA
89	153	TKD2	p.A781E	46	M	4

89	154	TKD2	p.A781E	36	F	3/Alive
90	155	TKD2	p.A781V	54	M	6
90	156		p.A781V	50	M	2/Alive
90	157		p.A781V	44	M	5
90	158		p.A781V	41	F	NA
90	159		p.A781V	32	F	2/Alive
90	160		p.A781V	36	F	2
90	161		p.A781V	30	M	3
90	162		p.A781V	NA	F	NA
90	163		p.A781V	43	F	2/Alive
90	164		p.A781V	38	M	3/Alive
90	165		p.A781V	45	M	2/Alive
90	166		p.A781V	34	F	4/Alive
91	167	TKD2	p.A781V_N783 del	NA	NA	NA
92	168	TKD2	p.I782T	28	F	1/Alive
93	169	TKD2	p.R782C	45	M	2/Alive
93	170		p.R782C	50	M	4
93	171		p.R782C	NA	NA	NA
94	172	TKD2	p.R782G	43	F	NA
94	173		p.R782G	33	F	NA
94	174		p.R782G	NA	F	1
94	175		p.R782G	38	M	2
94	176		p.R782G	37	M	1/Alive
95	177	TKD2	p.R782H	61	M	NA
95	178		p.R782H	44	M	NA
95	179		p.R782H	37	F	5
95	180		p.R782H	51	F	5/Alive
95	181		p.R782H	46	M	NA
95	182		p.R782H	51	F	2/Alive
95	183		p.R782H	NA	F	NA
95	184		p.R782H	61	F	2/Alive
95	185		p.R782H	51	F	NA
95	186		p.R782H	30	F	3M/Alive
95	187		p.R782H	46	F	2/Alive
96	188	TKD2	p.R782L	50	F	1
97	189	TKD2	p.R782W	51	F	1
98	190	TKD2	p.N783D	43	F	1/Alive
99	191	TKD2	p.V784M	39	F	6/Alive
100	192	TKD2	p.A792D	41	M	2/Alive
100	193		p.A792D	35	M	2/Alive
100	194		p.A792D	NA	NA	NA
100	195		p.A792D	41	M	5/Alive
101	196	TKD2	p.K793E	29	F	0.5/Alive
101	197		p.K793E	32	M	2/Alive
102	198	TKD2	p.K793T	40	M	1/Alive
102	199		p.K793T	41	M	1
102	200		p.K793T	41	M	6M/Alive
102	201		p.K793T	NA	NA	NA
103	202	TKD2	p.I794F	56	M	1/Alive
104	203	TKD2	p.I794N	29	F	0.5/Alive
105	204	TKD2	p.G795V	45	F	2/Alive
106	205	TKD2	p.F797C	40	M	1/Alive

107	206	TKD2	p.F797I	31	F	1/Alive
108	207	TKD2	p.F797L	51	F	1/Alive
109	208	TKD2	p.G798A	NA	NA	NA
110	209	TKD2	p.Y809S	37	M	1/Alive
111	210	TKD2	p.L817P	21	M	15
112	211	TKD2	p.L817Q	43	F	9/Alive
113	212	TKD2	p.K820N	45	F	1/Alive
114	213	TKD2	p.W821*	56	M	5/Alive
115	214	TKD2	p.W821C	35	F	15M/Alive
116	215	TKD2	p.W821R	32	F	NA
117	216	TKD2	p.M822I	44	F	1/Alive
118	217	TKD2	p.A823T	46	F	1/Alive
119	218	TKD2	p.A823D	NA	M	NA
119	219		p.A823D	47	M	2
120	220	TKD2	p.A823V	50	F	NA
120	221		p.A823V	46	M	NA
120	222		p.A823V	51	F	18M/Alive
121	223	TKD2	p.P824S	45	F	1/Alive
122	224	TKD2	p.P824R	NA	NA	NA
123	225	TKD2	p.E825K	42	F	NA
123	226		p.E825K	NA	NA	NA
123	227		p.E825K	NA	NA	NA
124	228	TKD2	p.E825Q	46	F	2/Alive
125	229	TKD2	p.I827N	36	M	2/Alive
126	230	TKD2	p.I827T	42	F	2/Alive
127	231	TKD2	p.F828S	39	F	NA
127	232		p.F828S	47	F	NA
128	233	TKD2	p.D829fs	48	M	1/Alive
129	234	TKD2	p.T833K	30	F	NA
130	235	TKD2	p.T833M	2	F	10
130	236		p.T833M	1M	M	9
131	237	TKD2	p.T833R	41	M	2/Alive
131	238		p.T833R	51	F	2/Alive
132	239	TKD2	p.D837H	47	M	18M/Alive
133	240	TKD2	p.D837Y	43	F	2/Alive
134	241	TKD2	p.V838L	51	M	4/Alive
134	242		p.V838L	53	M	2/Alive
134	243		p.V838L	54	M	2/Alive
134	244		p.V838L	NA	M	NA
134	245		p.V838L	NA	M	NA
135	246	TKD2	p.W839C	46	M	3/Alive
136	247	TKD2	p.Y841C	47	F	NA
137	248	TKD2	p.Y841H	NA	NA	NA
138	249	TKD2	p.Y841S	53	M	3/Alive
139	250	TKD2	p.G842V	60	M	NA
140	251	TKD2	p.I843_L844delinsGI	42	F	1/Alive
141	252	TKD2	p.I843F	55	M	4/Alive
142	253	TKD2	p.I843N	40	F	3/Alive
143	254	TKD2	p.I843T	42	F	2/Alive
144	255	TKD2	p.L845P	29	F	2/Alive
144	256	TKD2	p.L845P	36	M	NA
145	257	TKD2	p.W846L	41	M	2/Alive

146	258	TKD2	p.E847D	44	F	5/Alive
146	259		p.E847D	32	F	NA
146	260		p.E847D	NA	NA	NA
147	261	TKD2	p.E847K	34	M	1/Alive
147	262		p.E847K	49	F	12
148	263	TKD2	p.E847V	33	F	2/Alive
149	264	TKD2	p.F849del	44	F	NA
149	265		p.F849del	63	F	4
149	266		p.F849del	24	F	16M/Alive
150	267	TKD2	p.F849I	42	M	1/Alive
151	268	TKD2	p.F849S	46	F	5
151	269		p.F849S	58	F	1/Alive
152	270	TKD2	p.S850L	47	F	1/Alive
153	271	TKD2	p.L851P	NA	F	NA
153	272		p.L851P	28	F	7/Alive
153	273		p.L851P	27	F	8/Alive
153	274		p.L851P	24	F	3
154	275	TKD2	p.L851R	56	M	5
155	276	TKD2	p.G852Dfs	43	F	7/Alive
156	277	TKD2	p.N854K	44	F	5/Alive
156	278		p.N854K	29	F	NA
157	279	TKD2	p.P855T	41	F	8
157	280		p.P855T	34	M	NA
157	281		p.P855T	50	F	1
157	282		p.P855T	30	F	NA
158	283	TKD2	p.Y856H	39	F	3
158	284		p.Y856H	42	F	NA
159	285	TKD2	p.Y856S	37	F	2/Alive
160	286	TKD2	p.P857L	38	F	NA
161	287	TKD2	p.L868P	60	M	NA
161	288		p.L868P	55	F	8
162	289	TKD2	p.L868R	64	M	11
163	290	TKD2	p.V869G	45	M	NA
163	291		p.V869G	39	F	1/Alive
164	292	TKD2	p.M875I	40	F	43
165	293	TKD2	p.M875R	NA	NA	NA
166	294	TKD2	p.M875T	NA	F	NA
166	295		p.M875T	58	M	8
167	296	TKD2	p.A876Vfs	55	F	1
168	297	TKD2	p.Q877*	28	M	2/Alive
169	298	TKD2	p.P878H	53	F	5/Alive
170	299	TKD2	p.P878A	57	M	5/Alive
170	300		p.P878A	60	M	4/Alive
171	301	TKD2	p.P878S	45	M	10/Alive
171	302		p.P878S	45	F	NA
172	303	TKD2	p.P878T	39	F	10
173	304	TKD2	p.A881fs	73	M	1/Alive
174	305	TKD2	p.A881V	44	M	NA
175	306	TKD2	p.P882Pfs*70	24	M	1/Alive
176	307	TKD2	p.K883del	42	F	1/Alive
177	308	TKD2	c.2652T>G	40	F	1/Alive
178	309	TKD2	p.Y886Qfs*55	46	M	1/Alive
179	310	TKD2	c.889+1G>C	26	M	1/Alive

180	311	TKD2	p.C892_A894del	52	M	4/Alive
180	312		p.C892_A894del	52	M	7/Alive
181	313	TKD2	p.894_897del	38	M	1/Alive
182	314	TKD2	p.895_897del	37	M	2/Alive
183	315	TKD2	p.H899fs	42	M	1/Alive
184	316	TKD2	p.R900K	60	NA	NA
184	317		p.R900K	51	M	2/Alive
184	318		p.R900K	52	F	1/Alive
185	319	TKD2	p.R900T	45	F	2/Alive
186	320	TKD2	p.P901S	20	NA	25
186	321		p.P901S	39	F	2/Alive
187	322	TKD2	p.T902Sfs	49	F	2/Alive
188	323	TKD2	p.I906T	36	M	1/Alive
189	324	TKD2	p.L910P	36	F	1
190	325	TKD2	p.A914T	NA	NA	NA
191	326	TKD2	p.E916K	NA	NA	NA
192	327	TKD2	p.E920D	NA	NA	NA
193	328	TKD2	p.G936S	29	M	1/Alive
194	329	TKD2	p.G957R	49	M	8
195	330	Others	p.F971Sfs*7	46	M	NA
196	331	Others	c.49 G>T	26	M	2/Alive
197	332	Others	c.1754-3C>G	43	F	0.75/Alive
198	333	Others	c.1754-1G>C	0	M	10M
199	334	Others	c.1754-10 T > A	42	M	2M/Alive
200	335	Others	c.1858+1G>T	37	M	7M/Alive
201	336	Others	c.1858+5G>A	41	F	3/Alive
201	337		c.1858+5G>A	44	F	2/Alive
202	338	Others	c.1969+115_1969+116del	23	M	5
203	339	Others	c.2133_2919del heterozygous	37	F	3/Alive
204	340	Others	c.2319+1C>A	40	M	3
204	341		c.2319+1C>A	54	M	4
205	342	Others	c.2320-2A>G	50	M	5
206	343	Others	c.2442+1G>A	43	NA	NA
206	344		c.2442+1G>A	20	F	2/Alive
206	345		c.2442+1G>A	22.9	F	1.75/Alive
206	346		c.2442+1G>A	38.5	F	1.01/Alive
206	347	Others	c.2442+1G>T	47	F	NA
206	348		c.2442+1G>T	53	M	4
206	349		c.2442+1G>T	NA	NA	NA
207	350	Others	c.2442+2T>C	39	M	5
208	351	Others	c.2442+4A>G	38	F	2/Alive
209	352	Others	c.2442+5G>A	27	F	1/Alive
209	353		c.2442+5G>A	58	M	3/Alive
209	354		c.2442+5G>A	60	F	1/Alive
209	355		c.2442+5G>A	51	F	2/Alive
210	356	Others	c.2442+5G>C	23	F	1/Alive
211	357	Others	c.2442+2_2442+3dupT	41	F	NA
212	358	Others	c.2654+1G>A	39	M	4/Alive
213	359	Others	c.2563C＞A	45	M	18M/Alive

214	360	Others	c.2654_2654+3 del	43	F	7/Alive
215	361	Others	c.2655-1G>C	42	F	1/Alive
215	362		c.2655-1G>C	28	M	1/Alive
216	363	Others	c.2655-2A>G	50	F	2
216	364		c.2655-2A>G	42.5	F	1.5/Alive
217	365	Others	c.2763+1G>T (chr5:149433884C>A)	9	F	1/Alive
218	366	Others	c2909_2910insAT^cAC	29	M	NA
218	367		c2909_2910insAT^cAC	30	M	2/Alive
219	368	Others	delCTC	39	F	9M/Alive
220	369	Others	Deletion #1	43	F	6/Alive
221	370	Others	Deletion #2	37	M	4/Alive

EC: extracellular domain; TM: transmembrane domain; JMD: Juxtamembrane domain; TKD1/2: tyrosine kinase domain

Ref
2
40
41
42
42
43
15
28
44
42
6
11
6
16
45
41
41
41
41
41
41
28
42
46
15
6
42
47
43
44
43
41
48
49
50
37
51
52
49
2
53
42
11
46
54
30
43
55
30
56

57
57
58
59
59
60
57
61
43
15
42
19
19
62
62
63
42
47
2
64
64
42
44
44
37
65
37
30
46
63
52
66
36
4
67
42
40
42
5
47
57
62
68
44
42
28
66
69
42
4
5
5
17

50
50
50
30
52
70
71
72
73
62
19
63
19
28
17
62
47
55
74
30
57
66
75
64
30
62
30
30
30
37
76
77
34
78
79
43
47
42
42
42
37
80
16
81
81
19
19
28
19
4
17

82
34
75
75
83
4
5
54
42
42
42
42
84
18
85
43
19
58
58
78
78
15
59
59
75
73
36
74
86
87
73
88
47
89
42
15
90
91
92
19
20
42
47
93
81
93
19
28
42
64
42
42

42
19
11
37
69
15
42
94
2
95
15
4
5
58
59
96
17
84
63
19
19
42
97
37
29
29
42
98
99
100
11
47
101
30
102
34
46
54
103
64
33
19
42
33
104
68
68
47
5
33
11
37

105
19
106
107
108
59
30
6
15
30
1
42
4
109
42
42
42
7
110
111
112
40
40
40
37
33
42
36
55
30
50
113
15
114
19
30
115
42
34
66
28
66
28
59
30
6
64
70
15
116
28
117
24

118
11
42
119
120
121
122
6
37
42
42
68
42
43
50
50
42
50
59
12
42
67
123
124
125
126
127
64
128
42
40
63
129
116
116
130
17
19
131
42
57
57
24
47
30
2
126
6
7

42
47
37
42
132
2
12
6
15
15
6

1/2; AO: Age of

Table S4. Microglia replacement strategies in different figures. Related to Figures 5-7.

Figure	Age of mice at baseline	Microglia depletion	Transplanted microglia derivation	Brain collection
**5B**	One-month old	Two rounds of orally administrating PLX3397 for 7 days	Cultured GFP^+ microglia from newborn *Cx3cr1*$^{GFP/+}$ mice	Mouse brains were collected one-month after transplantation
**S4D**	One-month old	Orally administrating PLX3397 for seven days	Cultured GFP^+ microglia from newborn *Cx3cr1*$^{GFP/+}$ mice	Mouse brains were collected one-month after transplantation
**6B**	One-month old	Two rounds of orally administrating PLX3397 for 7 days	Cultured GFP^+ microglia from newborn *Cx3cr1*$^{GFP/+}$ mice	Mouse brains were collected seven-months after transplantation
**7B**	Nine or ten-month old	Two rounds of orally administrating PLX3397 for 7 days	Cultured GFP^+ microglia from newborn *Cx3cr1*$^{GFP/+}$ mice	Mouse brains were collected one-month after transplantation
**S7C**	Nine or ten-month old	Orally administrating PLX3397 for seven days	FACS-isolated GFP^+ microglia from newborn *Cx3cr1*$^{GFP/+}$ mice	Mouse brains were collected one-month after transplantation
**S8A**	Nine or ten-month old	0*Tp: Without PLX3397 administration	Cultured GFP^+ microglia from newborn *Cx3cr1*$^{GFP/+}$ mice	Mouse brains were collected one-month after transplantation
**S8D**	Nine or ten-month old	7*Tp: Orally administrating PLX3397 for seven days	Cultured GFP^+ microglia from newborn *Cx3cr1*$^{GFP/+}$ mice	Mouse brains were collected one-month after transplantation

Table S5. The primer sequences for qRT-PCR experiments. Related to STAR Methods. Related to Figure 4.

Name	GenBank	Forward primer (5'−3')	Reversed primer (5'−3')
Actb	NM_11461	TCTTGGGTATGGAATCCTGTGGCA	TCTTGGGTATGGAATCCTGTGGCA
Csf1r	NM_12978	GGTTGTAGAGCGGGTGAAA	AAGAGTGGGCCGATCTTTG
Csf3	NM_ 12985	TATAAAGGCCCCCTGGAGCTG	GCTGCAGGGCCATTAGCTTC
Csf2	NM_12981	GGCATTGTGGTCTACAGCCT	TGAAATTGCCCCGTAGACCC
Tmem119	NM_231633	GTCACTCCATCCCAGTTTCAC	CAGGGGACCATGTTGAGCTAT
P2ry12	NM_70839	CACTTTCCCGTATCCAGGGT	GCCTTGAGTGTTTCTGTAGGGTA
Axl	NM_26362	TCCTCCAAGGGATGGGTCAT	CTCACAGTGGGGAAACCGAA
Cst7	NM_13011	TGCTTACCAGCGAGCAGATT	GCTTCCCACACTACCACCTT
H2-Ab1	NM_14961	GCTTGAACAGCCCAATGTCG	CGCACTTTGATCTTGGCTGG
Lpl	NM_16956	AGGCATACAGGTGCAACTCC	TAGGGCATCTGAGAGCGAGT

Supplemental References

1. Lan, M.Y., Liu, J.S., Chang, C.C., Chen, Y.F., Su, C.S., Peng, C.H., and Chang, Y.Y. (2016). Clinicopathologic and Genetic Studies of 2 Patients With Hereditary Diffuse Leukoencephalopathy With Axonal Spheroids. Alzheimer Dis Assoc Disord *30*, 73-76. 10.1097/WAD.0000000000000067.
2. Chu, M., Wang, D.X., Cui, Y., Kong, Y., Liu, L., Xie, K.X., Xia, T.X., Zhang, J., Gao, R., Zhou, A.H., et al. (2021). Three novel mutations in Chinese patients with CSF1R-related leukoencephalopathy. Ann Transl Med *9*, 1072. 10.21037/atm-21-217.
3. Bai, Y., Lu, L., Cui, Y., Li, J., Liu, Y., Liu, L., Dong, J., Wang, Q., Qin, W., Wu, L., and Jia, J. (2018). Analysis of clinical and neuroimaging features in a Chinese family with hereditary diffuse leukoencephalopathy with neuroaxonal spheroids. Chinese journal of neurology *51*, 877-881.
4. Tian, W.T., Zhan, F.X., Liu, Q., Luan, X.H., Zhang, C., Shang, L., Zhang, B.Y., Pan, S.J., Miao, F., Hu, J., et al. (2019). Clinicopathologic characterization and abnormal autophagy of CSF1R-related leukoencephalopathy. Transl Neurodegener *8*, 32. 10.1186/s40035-019-0171-y.
5. Zhan, F.X., Zhu, Z.Y., Liu, Q., Zhou, H.Y., Luan, X.H., Huang, X.J., Liu, X.L., Tian, W.T., Wang, S.G., Song, X.X., et al. (2021). Altered structural and functional connectivity in CSF1R-related leukoencephalopathy. Brain Imaging Behav *15*, 1655-1666. 10.1007/s11682-020-00360-0.
6. Mao, C., Zhou, L., Zhou, L., Yang, Y., Niu, J., Li, J., Huang, X., Ren, H., Zhao, Y., Peng, B., and Gao, J. (2020). Biopsy histopathology in the diagnosis of adult-onset leukoencephalopathy with axonal spheroids and pigmented glia (ALSP). Neurol Sci *41*, 403-409. 10.1007/s10072-019-04116-7.
7. Xie, J.J., Ni, W., Wei, Q., Ma, H., Bai, G., Shen, Y., and Wu, Z.Y. (2020). New clinical characteristics and novel pathogenic variants of patients with hereditary leukodystrophies. CNS Neurosci Ther *26*, 567-575. 10.1111/cns.13284.
8. Liu, Q., Guo, X.N., Liu, C.Y., and Xu, W.H. (2020). A proposed synergistic effect of CSF1R and NMUR2 variants contributes to binge eating in hereditary diffuse leukoencephalopathy with spheroids. Ann Transl Med *8*, 7. 10.21037/atm.2019.11.30.
9. 王康, 彭国平, and 罗本燕 (2015). CSF1R 基因突变所致的伴球状体遗传性弥漫性白质脑病一例. held in 中国浙江舟山, pp. 2.
10. Wu, J., Tian, W., Zhan, F., Luan, X., and Cao, L. (2021). Generation of an human induced pluripotent stem cell JTUi007-A from a patient with CSF1R-related leukoencephalopathy carrying heterozygous p.Ile794Thr mutation in CSF1R gene. Stem Cell Res *57*, 102593. 10.1016/j.scr.2021.102593.
11. Wu, J., Cao, Y., Li, M., Li, B., Jia, X., and Cao, L. (2022). Altered intrinsic brain activity in patients with CSF1R-related leukoencephalopathy. Brain Imaging Behav *16*, 1842-1853. 10.1007/s11682-022-00646-5.
12. Wu, L., Liu, J., Sha, L., Wang, X., Li, J., Dong, J., and Jia, J. (2017). Sporadic Cases with Novel Mutations and Pedigree in Hereditary Leukoencephalopathy with Axonal Spheroids. J Alzheimers Dis *56*, 893-898. 10.3233/JAD-161193.
13. Wu, D., Zhao, J., and Zheng, L. (2024). Adult-onset leukoencephalopathy with persistent diffusion restriction dot lesions. Neurological Sciences *45*, 1797-1798. 10.1007/s10072-023-07212-x.

14. Komatsu, T., Takahashi, M., Omoto, S., and Iguchi, Y. (2023). Asymmetric focal cortical atrophy in CSF1R-related leukoencephalopathy; case report. Acta Neurol Belg *123*, 2001-2003. 10.1007/s13760-022-02065-1.

15. Ishiguro, T., Konno, T., Hara, N., Zhu, B., Okada, S., Shibata, M., Saika, R., Kitano, T., Toko, M., Nezu, T., et al. (2023). Novel partial deletions, frameshift and missense mutations of CSF1R in patents with CSF1R-related leukoencephalopathy. Eur J Neurol *30*, 1861-1870. 10.1111/ene.15796.

16. Mitsui, J., Matsukawa, T., Ishiura, H., Higasa, K., Yoshimura, J., Saito, T.L., Ahsan, B., Takahashi, Y., Goto, J., Iwata, A., et al. (2012). CSF1R mutations identified in three families with autosomal dominantly inherited leukoencephalopathy. Am J Med Genet B Neuropsychiatr Genet *159B*, 951-957. 10.1002/ajmg.b.32100.

17. Konno, T., Tada, M., Tada, M., Koyama, A., Nozaki, H., Harigaya, Y., Nishimiya, J., Matsunaga, A., Yoshikura, N., Ishihara, K., et al. (2014). Haploinsufficiency of CSF-1R and clinicopathologic characterization in patients with HDLS. Neurology *82*, 139-148. 10.1212/WNL.0000000000000046.

18. Saitoh, B.Y., Yamasaki, R., Hayashi, S., Yoshimura, S., Tateishi, T., Ohyagi, Y., Murai, H., Iwaki, T., Yoshida, K., and Kira, J. (2013). A case of hereditary diffuse leukoencephalopathy with axonal spheroids caused by a de novo mutation in CSF1R masquerading as primary progressive multiple sclerosis. Mult Scler *19*, 1367-1370. 10.1177/1352458513489854.

19. Kondo, Y., Matsushima, A., Nagasaki, S., Nakamura, K., Sekijima, Y., and Yoshida, K. (2020). Factors predictive of the presence of a CSF1R mutation in patients with leukoencephalopathy. Eur J Neurol *27*, 369-375. 10.1111/ene.14086.

20. Hamatani, M., Yamashita, H., Ochi, H., Ashida, S., Hashi, Y., Okada, Y., Fujii, C., Kawamura, K., Kitazawa, R., Nakagawa, M., et al. (2020). Altered features of monocytes in adult onset leukoencephalopathy with axonal spheroids and pigmented glia: A clue to the pathomechanism of microglial dyshomeostasis. Neurobiol Dis *140*, 104867. 10.1016/j.nbd.2020.104867.

21. Kitani-Morii, F., Kasai, T., Tomonaga, K., Saito, K., Mizuta, I., Yoshioka, A., Nakagawa, M., and Mizuno, T. (2014). Hereditary diffuse leukoencephalopathy with spheroids characterized by spastic hemiplegia preceding mental impairment. Intern Med *53*, 1377-1380. 10.2169/internalmedicine.53.1932.

22. 田中賢, 田中晋, 田中弘, 五十嵐勝, and 池内健 (2018). 上顎洞内に骨破壊と異所性石灰化像を認めた神経軸索スフェロイドを伴う遺伝性白質脳症 (HDLS) の 1 症例.

23. 池内, 健. (2014). 神経軸索スフェロイド形成をともなう遺伝性びまん性白質脳症（HDLS）の臨床像と画像所見. 臨床神経学 *54*, 1158-1161. 10.5692/clinicalneurol.54.1158.

24. Kim, M., Lee, H., Cho, H.J., Young Chun, S., Shin, J.H., Kim, E.J., Woo Ahn, J., Huh, G.Y., Baek, S.Y., and Lee, J.H. (2017). Pathologic Correlation of Paramagnetic White Matter Lesions in Adult-Onset Leukoencephalopathy With Axonal Spheroids and Pigmented Glia. J Neuropathol Exp Neurol *76*, 924-928. 10.1093/jnen/nlx086.

25. Kim, S.J., Cho, W., Kim, H.J., Na, D.L., Seo, S.W., Jung, N.Y., Lee, J.H., Lee, M.J., Kang, H., Seong, J.K., and Kim, E.J. (2024). Distinct patterns of white matter hyperintensity and cortical thickness of CSF1R-related leukoencephalopathy compared with subcortical ischemic vascular dementia. PLoS One *19*, e0308989. 10.1371/journal.pone.0308989.

26. Reddy Tallapalli, A.V., Nashi, S., Kamath, S.D., Srijithesh, P.R., Kulkarni, G.B., and Alladi, S.

(2022). A Rare Genetic Cause of Young Onset Rapidly Progressive Dementia- First Report from India. Neurol India *70*, 781-783. 10.4103/0028-3886.344666.

27. Rudrabhatla, P., Sabarish, S., Ramachandran, H., and Nair, S.S. (2021). Teaching NeuroImages: Rare Adult-Onset Genetic Leukoencephalopathy. Neurology *96*, e2561-e2562. 10.1212/WNL.0000000000011233.

28. Miura, T., Mezaki, N., Konno, T., Iwasaki, A., Hara, N., Miura, M., Funayama, M., Unai, Y., Tashiro, Y., Okita, K., et al. (2018). Identification and functional characterization of novel mutations including frameshift mutation in exon 4 of CSF1R in patients with adult-onset leukoencephalopathy with axonal spheroids and pigmented glia. J Neurol *265*, 2415-2424. 10.1007/s00415-018-9017-2.

29. Kleinfeld, K., Mobley, B., Hedera, P., Wegner, A., Sriram, S., and Pawate, S. (2013). Adult-onset leukoencephalopathy with neuroaxonal spheroids and pigmented glia: report of five cases and a new mutation. J Neurol *260*, 558-571. 10.1007/s00415-012-6680-6.

30. Rademakers, R., Baker, M., Nicholson, A.M., Rutherford, N.J., Finch, N., Soto-Ortolaza, A., Lash, J., Wider, C., Wojtas, A., DeJesus-Hernandez, M., et al. (2011). Mutations in the colony stimulating factor 1 receptor (CSF1R) gene cause hereditary diffuse leukoencephalopathy with spheroids. Nat Genet *44*, 200-205. 10.1038/ng.1027.

31. Van Gerpen, J.A., Wider, C., Broderick, D.F., Dickson, D.W., Brown, L.A., and Wszolek, Z.K. (2008). Insights into the dynamics of hereditary diffuse leukoencephalopathy with axonal spheroids. Neurology *71*, 925-929. 10.1212/01.wnl.0000325916.30701.21.

32. Sharma, R., Graff-Radford, J., Rademakers, R., Boeve, B.F., Petersen, R.C., and Jones, D.T. (2019). CSF1R mutation presenting as dementia with Lewy bodies. Neurocase *25*, 17-20. 10.1080/13554794.2019.1601230.

33. Codjia, P., Ayrignac, X., Mochel, F., Mouzat, K., Carra-Dalliere, C., Castelnovo, G., Ellie, E., Etcharry-Bouyx, F., Verny, C., Belliard, S., et al. (2018). Adult-Onset Leukoencephalopathy with Axonal Spheroids and Pigmented Glia: An MRI Study of 16 French Cases. AJNR Am J Neuroradiol *39*, 1657-1661. 10.3174/ajnr.A5744.

34. Karle, K.N., Biskup, S., Schule, R., Schweitzer, K.J., Kruger, R., Bauer, P., Bender, B., Nagele, T., and Schols, L. (2013). De novo mutations in hereditary diffuse leukoencephalopathy with axonal spheroids (HDLS). Neurology *81*, 2039-2044. 10.1212/01.wnl.0000436945.01023.ac.

35. Meyer-Ohlendorf, M., Braczynski, A., Al-Qaisi, O., Gessler, F., Biskup, S., Weise, L., Steinbach, J.P., Wagner, M., Mittelbronn, M., and Bahr, O. (2015). Comprehensive diagnostics in a case of hereditary diffuse leukodystrophy with spheroids. BMC Neurol *15*, 103. 10.1186/s12883-015-0368-3.

36. Lynch, D.S., Rodrigues Brandao de Paiva, A., Zhang, W.J., Bugiardini, E., Freua, F., Tavares Lucato, L., Macedo-Souza, L.I., Lakshmanan, R., Kinsella, J.A., Merwick, A., et al. (2017). Clinical and genetic characterization of leukoencephalopathies in adults. Brain *140*, 1204-1211. 10.1093/brain/awx045.

37. Guerreiro, R., Kara, E., Le Ber, I., Bras, J., Rohrer, J.D., Taipa, R., Lashley, T., Dupuits, C., Gurunlian, N., Mochel, F., et al. (2013). Genetic analysis of inherited leukodystrophies: genotype-phenotype correlations in the CSF1R gene. JAMA Neurol *70*, 875-882. 10.1001/jamaneurol.2013.698.

38. Molloy, A., Williams, L., Farrell, M., and O'Riordan, S. (2014). Hereditary Diffuse Leucoencephalopathy and Spheroids Resulting From a Mutation in CSF1R: A Rare Cause of

Parkinsonism. Mov Disord Clin Pract *1*, 132-133. 10.1002/mdc3.12033.

39. Konno, T., Yoshida, K., Mizuta, I., Mizuno, T., Kawarai, T., Tada, M., Nozaki, H., Ikeda, S.I., Onodera, O., Wszolek, Z.K., and Ikeuchi, T. (2018). Diagnostic criteria for adult-onset leukoencephalopathy with axonal spheroids and pigmented glia due to CSF1R mutation. Eur J Neurol *25*, 142-147. 10.1111/ene.13464.

40. Zhuang, L.P., Liu, C.Y., Li, Y.X., Huang, H.P., and Zou, Z.Y. (2020). Clinical features and genetic characteristics of hereditary diffuse leukoencephalopathy with spheroids due to CSF1R mutation: a case report and literature review. Ann Transl Med *8*, 11. 10.21037/atm.2019.12.17.

41. Giau, V.V., Senanarong, V., Bagyinszky, E., An, S.S.A., and Kim, S. (2019). Analysis of 50 Neurodegenerative Genes in Clinically Diagnosed Early-Onset Alzheimer's Disease. Int J Mol Sci *20*. 10.3390/ijms20061514.

42. Wu, J., Cheng, X., Ji, D., Niu, H., Yao, S., Lv, X., Wang, J., Li, Z., Zheng, H., Cao, Y., et al. (2024). The Phenotypic and Genotypic Spectrum of CSF1R-Related Disorder in China. Mov Disord *39*, 798-813. 10.1002/mds.29764.

43. Tsai, P.C., Fuh, J.L., Yang, C.C., Chang, A., Lien, L.M., Wang, P.N., Lai, K.L., Tsai, Y.S., Lee, Y.C., and Liao, Y.C. (2021). Clinical and genetic characterization of adult-onset leukoencephalopathy caused by CSF1R mutations. Ann Clin Transl Neurol *8*, 2121-2131. 10.1002/acn3.51467.

44. Guo, L., Bertola, D.R., Takanohashi, A., Saito, A., Segawa, Y., Yokota, T., Ishibashi, S., Nishida, Y., Yamamoto, G.L., Franco, J., et al. (2019). Bi-allelic CSF1R Mutations Cause Skeletal Dysplasia of Dysosteosclerosis-Pyle Disease Spectrum and Degenerative Encephalopathy with Brain Malformation. Am J Hum Genet *104*, 925-935. 10.1016/j.ajhg.2019.03.004.

45. Riku, Y., Ando, T., Goto, Y., Mano, K., Iwasaki, Y., Sobue, G., and Yoshida, M. (2014). Early pathologic changes in hereditary diffuse leukoencephalopathy with spheroids. J Neuropathol Exp Neurol *73*, 1183-1190. 10.1097/NEN.0000000000000139.

46. Schuberth, M., Levin, J., Sawalhe, D., Schwarzkopf, R., von Baumgarten, L., Ertl-Wagner, B., Rominger, A., Arzberger, T., Kretzschmar, H.A., Frobose, T., et al. (2014). [Hereditary diffuse leukencephalopathy with spheroids: a microgliopathy due to CSF1 receptor impairment]. Nervenarzt *85*, 465-470. 10.1007/s00115-014-4052-4.

47. Hu, B., Zhou, Y., Wu, C., Xiao, N., Li, J., Li, X., Li, Y., Zhang, X., Huang, X., Song, Y., et al. (2025). Evaluation of Soluble Colony Stimulating Factor 1 Receptor (CSF1R) in Peripheral Blood as a Diagnostic Marker of CSF1R-Related Disorder (CSF1R-RD) in a Murine Model and CSF1R-RD Patients. Ann Neurol *97*, 397-403. 10.1002/ana.27147.

48. Monies, D., Maddirevula, S., Kurdi, W., Alanazy, M.H., Alkhalidi, H., Al-Owain, M., Sulaiman, R.A., Faqeih, E., Goljan, E., Ibrahim, N., et al. (2017). Autozygosity reveals recessive mutations and novel mechanisms in dominant genes: implications in variant interpretation. Genet Med *19*, 1144-1150. 10.1038/gim.2017.22.

49. Sassi, C., Capozzo, R., Hammer, M., Zecca, C., Federoff, M., Blauwendraat, C., Bernstein, N., Ding, J., Gibbs, J.R., Price, T., et al. (2021). Exploring dementia and neuronal ceroid lipofuscinosis genes in 100 FTD-like patients from 6 towns and rural villages on the Adriatic Sea cost of Apulia. Sci Rep *11*, 6353. 10.1038/s41598-021-85494-x.

50. Sassi, C., Nalls, M.A., Ridge, P.G., Gibbs, J.R., Lupton, M.K., Troakes, C., Lunnon, K., Al-Sarraj, S., Brown, K.S., Medway, C., et al. (2018). Mendelian adult-onset leukodystrophy genes in Alzheimer's disease: critical influence of CSF1R and NOTCH3. Neurobiol Aging *66*, 179

e117-179 e129. 10.1016/j.neurobiolaging.2018.01.015.

51. Chen, Z., Tan, Y.J., Lian, M.M., Tandiono, M., Foo, J.N., Lim, W.K., Kandiah, N., Tan, E.K., and Ng, A.S.L. (2021). High Diagnostic Utility Incorporating a Targeted Neurodegeneration Gene Panel With MRI Brain Diagnostic Algorithms in Patients With Young-Onset Cognitive Impairment With Leukodystrophy. Front Neurol *12*, 631407. 10.3389/fneur.2021.631407.

52. Konno, T., Miura, T., Harriott, A.M., Mezaki, N., Edwards, E.S., Rademakers, R., Ross, O.A., Meschia, J.F., Ikeuchi, T., and Wszolek, Z.K. (2018). Partial loss of function of colony-stimulating factor 1 receptor in a patient with white matter abnormalities. Eur J Neurol *25*, 875-881. 10.1111/ene.13611.

53. Ghadiri, M., Buckland, M.E., Sutton, I.J., Al Jahdhami, S., Flanagan, S., Heard, R., Barnett, Y., Brennan, J., and Barnett, M.H. (2014). Progressive neuropsychiatric symptoms and motor impairment. JAMA Neurol *71*, 794-798. 10.1001/jamaneurol.2013.6308.

54. Hofer, T.P., Zawada, A.M., Frankenberger, M., Skokann, K., Satzl, A.A., Gesierich, W., Schuberth, M., Levin, J., Danek, A., Rotter, B., et al. (2015). slan-defined subsets of CD16-positive monocytes: impact of granulomatous inflammation and M-CSF receptor mutation. Blood *126*, 2601-2610. 10.1182/blood-2015-06-651331.

55. Blauwendraat, C., Pletnikova, O., Geiger, J.T., Murphy, N.A., Abramzon, Y., Rudow, G., Mamais, A., Sabir, M.S., Crain, B., Ahmed, S., et al. (2019). Genetic analysis of neurodegenerative diseases in a pathology cohort. Neurobiol Aging *76*, 214 e211-214 e219. 10.1016/j.neurobiolaging.2018.11.007.

56. Fujioka, S., Broderick, D.F., Sundal, C., Baker, M.C., Rademakers, R., and Wszolek, Z.K. (2013). An adult-onset leukoencephalopathy with axonal spheroids and pigmented glia accompanied by brain calcifications: a case report and a literature review of brain calcifications disorders. J Neurol *260*, 2665-2668. 10.1007/s00415-013-7093-x.

57. Konno, T., Broderick, D.F., Mezaki, N., Isami, A., Kaneda, D., Tashiro, Y., Tokutake, T., Keegan, B.M., Woodruff, B.K., Miura, T., et al. (2017). Diagnostic Value of Brain Calcifications in Adult-Onset Leukoencephalopathy with Axonal Spheroids and Pigmented Glia. AJNR Am J Neuroradiol *38*, 77-83. 10.3174/ajnr.A4938.

58. Abe, T., Kawarai, T., Fujita, K., Sako, W., Terasawa, Y., Matsuda, T., Sakai, W., Tsukamoto-Miyashiro, A., Matsui, N., Izumi, Y., et al. (2017). MR Spectroscopy in Patients with Hereditary Diffuse Leukoencephalopathy with Spheroids and Asymptomatic Carriers of Colony-stimulating Factor 1 Receptor Mutation. Magn Reson Med Sci *16*, 297-303. 10.2463/mrms.mp.2016-0016.

59. Kim, J.R., Lee, S., Seo, S.W., Jang, J.H., Suh, Y.L., Park, J.H., Lee, S.Y., Son, H.J., Kwon, H.J., Kim, E.J., et al. (2025). Clinical spectrum of adult-onset leukoencephalopathy with axonal spheroids and pigmented glia in individuals of Korean ancestry. Sci Rep *15*, 1857. 10.1038/s41598-024-84665-w.

60. Ho, V.M., Hovsepian, D.A., and Shieh, P.B. (2019). Myelopathy in a patient with leukodystrophy due to CSF1R mutation. Neurol Genet *5*, e376. 10.1212/NXG.0000000000000376.

61. Daida, K., Nishioka, K., Li, Y., Nakajima, S., Tanaka, R., and Hattori, N. (2017). CSF1R Mutation p.G589R and the Distribution Pattern of Brain Calcification. Intern Med *56*, 2507-2512. 10.2169/internalmedicine.8462-16.

62. Konno, T., Yoshida, K., Mizuno, T., Kawarai, T., Tada, M., Nozaki, H., Ikeda, S.I., Nishizawa,

M., Onodera, O., Wszolek, Z.K., and Ikeuchi, T. (2017). Clinical and genetic characterization of adult-onset leukoencephalopathy with axonal spheroids and pigmented glia associated with CSF1R mutation. Eur J Neurol *24*, 37-45. 10.1111/ene.13125.

63. Lynch, D.S., Jaunmuktane, Z., Sheerin, U.M., Phadke, R., Brandner, S., Milonas, I., Dean, A., Bajaj, N., McNicholas, N., Costello, D., et al. (2016). Hereditary leukoencephalopathy with axonal spheroids: a spectrum of phenotypes from CNS vasculitis to parkinsonism in an adult onset leukodystrophy series. J Neurol Neurosurg Psychiatry *87*, 512-519. 10.1136/jnnp-2015-310788.

64. Schmitz, A.S., Raju, J., Kohler, W., Klebe, S., Cheheb, K., Reschke, F., Biskup, S., Haack, T.B., Roeben, B., Kellner, M., et al. (2024). Novel variants in CSF1R associated with adult-onset leukoencephalopathy with axonal spheroids and pigmented glia (ALSP). J Neurol *271*, 6025-6037. 10.1007/s00415-024-12557-0.

65. Bayat, M., Shekhrajka, N., and Bayat, A. (2019). Hereditary leukodystrophy with axonal spheroids (HDLS) presenting subacutely: a CNS-vasculitis mimic. Acta Neurol Belg *119*, 633-635. 10.1007/s13760-019-01096-5.

66. Chitu, V., Biundo, F., Shlager, G.G.L., Park, E.S., Wang, P., Gulinello, M.E., Gokhan, S., Ketchum, H.C., Saha, K., DeTure, M.A., et al. (2020). Microglial Homeostasis Requires Balanced CSF-1/CSF-2 Receptor Signaling. Cell Rep *30*, 3004-3019 e3005. 10.1016/j.celrep.2020.02.028.

67. Oosterhof, N., Chang, I.J., Karimiani, E.G., Kuil, L.E., Jensen, D.M., Daza, R., Young, E., Astle, L., van der Linde, H.C., Shivaram, G.M., et al. (2019). Homozygous Mutations in CSF1R Cause a Pediatric-Onset Leukoencephalopathy and Can Result in Congenital Absence of Microglia. Am J Hum Genet *104*, 936-947. 10.1016/j.ajhg.2019.03.010.

68. Battisti, C., Di Donato, I., Bianchi, S., Monti, L., Formichi, P., Rufa, A., Taglia, I., Cerase, A., Dotti, M.T., and Federico, A. (2014). Hereditary diffuse leukoencephalopathy with axonal spheroids: three patients with stroke-like presentation carrying new mutations in the CSF1R gene. J Neurol *261*, 768-772. 10.1007/s00415-014-7257-3.

69. Gelfand, J.M., Greenfield, A.L., Barkovich, M., Mendelsohn, B.A., Van Haren, K., Hess, C.P., and Mannis, G.N. (2020). Allogeneic HSCT for adult-onset leukoencephalopathy with spheroids and pigmented glia. Brain *143*, 503-511. 10.1093/brain/awz390.

70. Du, Q., Chen, H., Shi, Z., Zhang, Y., Wang, J., and Zhou, H. (2019). A novel mutation in the CSF1R gene causes hereditary diffuse leukoencephalopathy with axonal spheroids. Neurol Sci *40*, 1287-1290. 10.1007/s10072-018-3693-7.

71. Huang, H., Cao, L., and Chen, H. (2021). Dynamic analysis of CSF1R-related leukoencephalopathy on magnetic resonance imaging: a case report. BMC Neurol *21*, 156. 10.1186/s12883-021-02182-z.

72. Konno, T., Kasanuki, K., Ikeuchi, T., Dickson, D.W., and Wszolek, Z.K. (2018). CSF1R-related leukoencephalopathy: A major player in primary microgliopathies. Neurology *91*, 1092-1104. 10.1212/WNL.0000000000006642.

73. Kinoshita, M., Yoshida, K., Oyanagi, K., Hashimoto, T., and Ikeda, S. (2012). Hereditary diffuse leukoencephalopathy with axonal spheroids caused by R782H mutation in CSF1R: case report. J Neurol Sci *318*, 115-118. 10.1016/j.jns.2012.03.012.

74. Nicholson, A.M., Baker, M.C., Finch, N.A., Rutherford, N.J., Wider, C., Graff-Radford, N.R., Nelson, P.T., Clark, H.B., Wszolek, Z.K., Dickson, D.W., et al. (2013). CSF1R mutations link

POLD and HDLS as a single disease entity. Neurology *80*, 1033-1040. 10.1212/WNL.0b013e31828726a7.

75. Kim, E.J., Shin, J.H., Lee, J.H., Kim, J.H., Na, D.L., Suh, Y.L., Hwang, S.J., Lee, J.H., Lee, Y.M., Shin, M.J., et al. (2015). Adult-onset leukoencephalopathy with axonal spheroids and pigmented glia linked CSF1R mutation: Report of four Korean cases. J Neurol Sci *349*, 232-238. 10.1016/j.jns.2014.12.021.

76. Hoffmann, S., Murrell, J., Harms, L., Miller, K., Meisel, A., Brosch, T., Scheel, M., Ghetti, B., Goebel, H.H., and Stenzel, W. (2014). Enlarging the nosological spectrum of hereditary diffuse leukoencephalopathy with axonal spheroids (HDLS). Brain Pathol *24*, 452-458. 10.1111/bpa.12120.

77. Inui, T., Kawarai, T., Fujita, K., Kawamura, K., Mitsui, T., Orlacchio, A., Kamada, M., Abe, T., Izumi, Y., and Kaji, R. (2013). A new CSF1R mutation presenting with an extensive white matter lesion mimicking primary progressive multiple sclerosis. J Neurol Sci *334*, 192-195. 10.1016/j.jns.2013.08.020.

78. Foulds, N., Pengelly, R.J., Hammans, S.R., Nicoll, J.A., Ellison, D.W., Ditchfield, A., Beck, S., and Ennis, S. (2015). Adult-Onset Leukoencephalopathy with Axonal Spheroids and Pigmented Glia Caused by a Novel R782G Mutation in CSF1R. Sci Rep *5*, 10042. 10.1038/srep10042.

79. Yokote, A., Ouma, S., Takahashi, K., Hara, F., Yoshida, K., and Tsuboi, Y. (2020). [A case of hereditary diffuse leukoencephalopathy with spheroids and pigmented glia presenting with long-term mild psychiatric symptoms]. Rinsho Shinkeigaku *60*, 420-424. 10.5692/clinicalneurol.60.cn-001370.

80. Lubomski, M., Buckland, M.E., Sy, J., Wei, H., Tan, I.Y.L., Kane, B., and Spring, P.J. (2018). Adult-onset leukoencephalopathy with neuroaxonal spheroids and pigmented glia mimicking systemic lupus erythematosus cerebral vasculitis. J Neurol Sci *395*, 25-28. 10.1016/j.jns.2018.09.025.

81. Kinoshita, M., Kondo, Y., Yoshida, K., Fukushima, K., Hoshi, K., Ishizawa, K., Araki, N., Yazawa, I., Washimi, Y., Saitoh, B., et al. (2014). Corpus callosum atrophy in patients with hereditary diffuse leukoencephalopathy with neuroaxonal spheroids: an MRI-based study. Intern Med *53*, 21-27. 10.2169/internalmedicine.53.0863.

82. Ahmed, R., Guerreiro, R., Rohrer, J.D., Guven, G., Rossor, M.N., Hardy, J., and Fox, N.C. (2013). A novel A781V mutation in the CSF1R gene causes hereditary diffuse leucoencephalopathy with axonal spheroids. J Neurol Sci *332*, 141-144. 10.1016/j.jns.2013.06.007.

83. Prieto-Morin, C., Ayrignac, X., Ellie, E., Tournier-Lasserve, E., and Labauge, P. (2016). CSF1R-related leukoencephalopathy mimicking primary progressive multiple sclerosis. J Neurol *263*, 1864-1865. 10.1007/s00415-016-8197-x.

84. Delaney, C., Farrell, M., Doherty, C.P., Brennan, K., O'Keeffe, E., Greene, C., Byrne, K., Kelly, E., Birmingham, N., Hickey, P., et al. (2021). Attenuated CSF-1R signalling drives cerebrovascular pathology. EMBO Mol Med *13*, e12889. 10.15252/emmm.202012889.

85. 于晓攀, 崔荣荣, 孙静, 刘潇, 李福锦, and 周畅 (2017). 遗传性弥漫性白质脑病合并轴索球样变的临床和影像学特点(附 1 例报告). 临床神经病学杂志 *30*, 464-467.

86. Robinson, J.L., Suh, E., Wood, E.M., Lee, E.B., Coslett, H.B., Raible, K., Lee, V.M., Trojanowski, J.Q., and Van Deerlin, V.M. (2015). Common neuropathological features underlie distinct clinical presentations in three siblings with hereditary diffuse leukoencephalopathy with

spheroids caused by CSF1R p.Arg782His. Acta Neuropathol Commun *3*, 42. 10.1186/s40478-015-0219-x.

87. Stoiloudis, P., Parissis, D., Smyrni, N., Stardeli, T., Afrantou, T., Konstantinopoulou, E., Grigoriadis, N., and Ioannidis, P. (2021). Hereditary diffuse leukoencephalopathy with spheroids mimicking primary progressive aphasia: report of a Greek case. Neurol Sci *42*, 3431-3433. 10.1007/s10072-021-05257-4.

88. Shu, Y., Long, L., Liao, S., Yang, J., Li, J., Qiu, W., Yang, Y., Bao, J., Wu, A., Hu, X., and Lu, Z. (2016). Involvement of the optic nerve in mutated CSF1R-induced hereditary diffuse leukoencephalopathy with axonal spheroids. BMC Neurol *16*, 171. 10.1186/s12883-016-0694-0.

89. Bonvegna, S., Straccia, G., Golfre Andreasi, N., Elia, A.E., Marucci, G., Di Bella, D., Cilia, R., and Eleopra, R. (2020). Parkinsonism and Nigrostriatal Damage Secondary to CSF1R-Related Primary Microgliopathy. Mov Disord *35*, 2360-2362. 10.1002/mds.28290.

90. La Piana, R., Webber, A., Guiot, M.C., Del Pilar Cortes, M., and Brais, B. (2014). A novel mutation in the CSF1R gene causes a variable leukoencephalopathy with spheroids. Neurogenetics *15*, 289-294. 10.1007/s10048-014-0413-1.

91. Ueda, S., Yamashita, H., Hikiami, R., Sawamoto, N., Yoshida, K., and Takahashi, R. (2015). A novel A792D mutation in the CSF1R gene causes hereditary diffuse leukoencephalopathy with axonal spheroids characterized by slow progression. eNeurologicalSci *1*, 7-9. 10.1016/j.ensci.2015.07.001.

92. Zur-Wyrozumska, K., Kaczmarska, P., and Mensah-Glanowska, P. (2021). Adult-onset leukoencephalopathy with axonal spheroids and pigmented glia associated with an A792D mutation in the CSF1R gene in a Polish patient. Neurol Neurochir Pol *55*, 322-324. 10.5603/PJNNS.a2021.0012.

93. Kondo, Y., Kinoshita, M., Fukushima, K., Yoshida, K., and Ikeda, S. (2013). Early involvement of the corpus callosum in a patient with hereditary diffuse leukoencephalopathy with spheroids carrying the de novo K793T mutation of CSF1R. Intern Med *52*, 503-506. 10.2169/internalmedicine.52.8879.

94. Chen, J., Luo, S., Li, N., Li, H., Han, J., and Ling, L. (2020). A Novel Missense Mutation of the CSF1R Gene Causes Incurable CSF1R-Related Leukoencephalopathy: Case Report and Review of Literature. Int J Gen Med *13*, 1613-1620. 10.2147/IJGM.S286421.

95. Coomans, C., Sieben, A., Lammens, M., Ceuterick-de Groote, C., Vandenbroecke, C., Goethals, I., Van Melkebeke, D., and Hemelsoet, D. (2018). Early-onset dementia, leukoencephalopathy and brain calcifications: a clinical, imaging and pathological comparison of ALSP and PLOSL/Nasu Hakola disease. Acta Neurol Belg *118*, 607-615. 10.1007/s13760-018-1023-8.

96. Terasawa, Y., Osaki, Y., Kawarai, T., Sugimoto, T., Orlacchio, A., Abe, T., Izumi, Y., and Kaji, R. (2013). Increasing and persistent DWI changes in a patient with hereditary diffuse leukoencephalopathy with spheroids. J Neurol Sci *335*, 213-215. 10.1016/j.jns.2013.08.027.

97. Garg, D., Vaingankar, A., Gupta, A., Rajan, R., Garg, A., Agarwal, A., Mustafa, F., Radhakrishnan, D.M., Pandit, A.K., Vishnu, V.Y., et al. (2025). CSF1R-related adult-onset leukoencephalopathy with axonal spheroids: A case series of four Asian Indian patients. J Mov Disord. 10.14802/jmd.25004.

98. Mochel, F., Delorme, C., Czernecki, V., Froger, J., Cormier, F., Ellie, E., Fegueux, N., Lehericy, S., Lumbroso, S., Schiffmann, R., et al. (2019). Haematopoietic stem cell transplantation in

CSF1R-related adult-onset leukoencephalopathy with axonal spheroids and pigmented glia. J Neurol Neurosurg Psychiatry *90*, 1375-1376. 10.1136/jnnp-2019-320701.

99. Tamhankar, P.M., Zhu, B., Tamhankar, V.P., Mithbawkar, S., Seabra, L., Livingston, J.H., Ikeuchi, T., and Crow, Y.J. (2020). A Novel Hypomorphic CSF1R Gene Mutation in the Biallelic State Leading to Fatal Childhood Neurodegeneration. Neuropediatrics *51*, 302-306. 10.1055/s-0040-1702161.

100. Daghagh, H., Rahbar Kafshboran, H., Daneshmandpour, Y., Nasiri Aghdam, M., Talebian, S., Nouri Nojadeh, J., Hamzeiy, H., Biskup, S., and Sakhinia, E. (2023). Homozygous mutation in CSF1R causes brain abnormalities, neurodegeneration, and dysosteosclerosis (BANDDOS). Bioimpacts *13*, 183-190. 10.34172/bi.2022.23528.

101. Misirocchi, F., Zilioli, A., Benussi, A., Capellari, S., Mutti, C., Florindo, I., Spallazzi, M., and Parrino, L. (2023). A Novel CSF1R Mutation Mimicking Frontotemporal Dementia: A Glimpse into a Microgliopathy. Can J Neurol Sci *50*, 642-644. 10.1017/cjn.2022.265.

102. Hayer, S.N., Schelling, Y., Hoeflinger, P., Hauser, S., and Schols, L. (2018). Generation of an induced pluripotent stem cell line from a patient with adult-onset leukoencephalopathy with axonal spheroids and pigmented glia (ALSP): HIHCNi003-A. Stem Cell Res *30*, 206-209. 10.1016/j.scr.2018.06.011.

103. Levin, J., Tiedt, S., Arzberger, T., Biskup, S., Schuberth, M., Stenglein-Krapf, G., Kreth, F.W., Hogen, T., la Fougere, C., Linn, J., et al. (2014). Diffuse leukoencephalopathy with spheroids: biopsy findings and a novel mutation. Clin Neurol Neurosurg *122*, 113-115. 10.1016/j.clineuro.2014.04.022.

104. Kraya, T., Quandt, D., Pfirrmann, T., Kindermann, A., Lampe, L., Schroeter, M.L., Kohlhase, J., Stoevesandt, D., Hoffmann, K., and Villavicencio-Lorini, P. (2019). Functional characterization of a novel CSF1R mutation causing hereditary diffuse leukoencephalopathy with spheroids. Mol Genet Genomic Med *7*, e00595. 10.1002/mgg3.595.

105. Blume, J., and Weissert, R. (2017). Suspected Perinatal Depression Revealed to be Hereditary Diffuse Leukoencephalopathy with Spheroids. J Mov Disord *10*, 59-61. 10.14802/jmd.16050.

106. Di Donato, I., Stabile, C., Bianchi, S., Taglia, I., Mignarri, A., Salvatore, S., Giorgio, E., Brusco, A., Simone, I., Dotti, M.T., and Federico, A. (2015). A Novel CSF1R Mutation in a Patient with Clinical and Neuroradiological Features of Hereditary Diffuse Leukoencephalopathy with Axonal Spheroids. J Alzheimers Dis *47*, 319-322. 10.3233/JAD-150097.

107. Kim, S.I., Jeon, B., Bae, J., Won, J.K., Kim, H.J., Yim, J., Kim, Y.J., and Park, S.H. (2019). An Autopsy Proven Case of CSF1R-mutant Adult-onset Leukoencephalopathy with Axonal Spheroids and Pigmented Glia (ALSP) with Premature Ovarian Failure. Exp Neurobiol *28*, 119-129. 10.5607/en.2019.28.1.119.

108. Gore, E., Manley, A., Dees, D., Appleby, B.S., and Lerner, A.J. (2016). A young-onset frontal dementia with dramatic calcifications due to a novel CSF1R mutation. Neurocase *22*, 257-262. 10.1080/13554794.2016.1175635.

109. Wang, M., and Zhang, X. (2019). A novel CSF-1R mutation in a family with hereditary diffuse leukoencephalopathy with axonal spheroids misdiagnosed as hydrocephalus. Neurogenetics *20*, 155-160. 10.1007/s10048-019-00579-0.

110. Granberg, T., Hashim, F., Andersen, O., Sundal, C., and Karrenbauer, V.D. (2016). Hereditary diffuse leukoencephalopathy with spheroids - a volumetric and radiological comparison with multiple sclerosis patients and healthy controls. Eur J Neurol *23*, 817-822. 10.1111/ene.12948.

111. Sundal, C., Baker, M., Karrenbauer, V., Gustavsen, M., Bedri, S., Glaser, A., Myhr, K.M., Haugarvoll, K., Zetterberg, H., Harbo, H., et al. (2015). Hereditary diffuse leukoencephalopathy with spheroids with phenotype of primary progressive multiple sclerosis. Eur J Neurol *22*, 328-333. 10.1111/ene.12572.

112. Cheng, X., Shen, W., Zou, H., Shen, L., Gu, X., Huang, D., Sun, Y., Wang, B., Tian, Q., and Xu, J. (2015). [Analysis of CSF1R gene mutation in a Chinese family with hereditary diffuse leukoencephalopathy with neuroaxonal spheroids]. Zhonghua Yi Xue Yi Chuan Xue Za Zhi *32*, 208-212. 10.3760/cma.j.issn.1003-9406.2015.02.012.

113. Oboudiyat, C., Bigio, E.H., Bonakdarpour, B., Baker, M.C., Rademakers, R., Weintraub, S., and Mesulam, M.M. (2015). Diffuse leukoencephalopathy with spheroids presenting as primary progressive aphasia. Neurology *85*, 652-653. 10.1212/WNL.0000000000001851.

114. Tipton, P.W., Stanley, E.R., Chitu, V., and Wszolek, Z.K. (2021). Is Pre-Symptomatic Immunosuppression Protective in CSF1R-Related Leukoencephalopathy? Mov Disord *36*, 852-856. 10.1002/mds.28515.

115. Sundal, C., Van Gerpen, J.A., Nicholson, A.M., Wider, C., Shuster, E.A., Aasly, J., Spina, S., Ghetti, B., Roeber, S., Garbern, J., et al. (2012). MRI characteristics and scoring in HDLS due to CSF1R gene mutations. Neurology *79*, 566-574. 10.1212/WNL.0b013e318263575a.

116. Saitoh, B.Y., Yamasaki, R., Hiwatashi, A., Matsushita, T., Hayashi, S., Mitsunaga, Y., Maeda, Y., Isobe, N., Yoshida, K., Ikeda, S.I., and Kira, J.I. (2019). Discriminative clinical and neuroimaging features of motor-predominant hereditary diffuse leukoencephalopathy with axonal spheroids and primary progressive multiple sclerosis: A preliminary cross-sectional study. Mult Scler Relat Disord *31*, 22-31. 10.1016/j.msard.2019.03.008.

117. Kunii, M., Doi, H., Ishii, Y., Ohba, C., Tanaka, K., Tada, M., Fukai, R., Hashiguchi, S., Kishida, H., Ueda, N., et al. (2018). Genetic analysis of adult leukoencephalopathy patients using a custom-designed gene panel. Clin Genet *94*, 232-238. 10.1111/cge.13371.

118. Kim, E.J., Kim, Y.E., Jang, J.H., Cho, E.H., Na, D.L., Seo, S.W., Jung, N.Y., Jeong, J.H., Kwon, J.C., Park, K.H., et al. (2018). Analysis of frontotemporal dementia, amyotrophic lateral sclerosis, and other dementia-related genes in 107 Korean patients with frontotemporal dementia. Neurobiol Aging *72*, 186 e181-186 e187. 10.1016/j.neurobiolaging.2018.06.031.

119. Shi, T., Li, J., Tan, C., and Chen, J. (2019). Diagnosis of hereditary diffuse leukoencephalopathy with neuroaxonal spheroids based on next-generation sequencing in a family: Case report and literature review. Medicine (Baltimore) *98*, e15802. 10.1097/MD.0000000000015802.

120. Cochran, J.N., McKinley, E.C., Cochran, M., Amaral, M.D., Moyers, B.A., Lasseigne, B.N., Gray, D.E., Lawlor, J.M.J., Prokop, J.W., Geier, E.G., et al. (2019). Genome sequencing for early-onset or atypical dementia: high diagnostic yield and frequent observation of multiple contributory alleles. Cold Spring Harb Mol Case Stud *5*. 10.1101/mcs.a003491.

121. Kortvelyessy, P., Krageloh-Mann, I., Mawrin, C., Heinze, H.J., Bittner, D., Wieland, I., Zenker, M., and Nestor, P. (2015). Hereditary diffuse leukoencephalopathy with spheroids (HDLS) with a novel CSF1R mutation and spinal cord involvement. J Neurol Sci *358*, 515-517. 10.1016/j.jns.2015.09.370.

122. Chen, L., Xu, H., and Lu, Z. (2025). Late-onset CSF1R-related Disorder: A Case Report. Cogn Behav Neurol *38*, 16-20. 10.1097/WNN.0000000000000385.

123. Wu, X., Sun, C., Wang, X., Liu, Y., Wu, W., and Jia, G. (2022). Identification of a de novo splicing mutation in the CSF1R gene in a Chinese patient with hereditary diffuse

leukoencephalopathy with spheroids. Neurol Sci *43*, 3265-3272. 10.1007/s10072-021-05755-5.

124. Yang, X., Huang, P., Tan, Y., and Xiao, Q. (2019). A Novel Splicing Mutation in the CSF1R Gene in a Family With Hereditary Diffuse Leukoencephalopathy With Axonal Spheroids. Front Genet *10*, 491. 10.3389/fgene.2019.00491.

125. Shixing, X., Wei, W., Xueyan, H., and Wei, T. (2022). Pathogenicity analysis and a novel case report of intronic mutations in CSF1R gene. Neurocase *28*, 251-257. 10.1080/13554794.2022.2071625.

126. Jiang, J., Li, W., Wang, X., Du, Z., Chen, J., Liu, Y., Li, W., Lu, Z., Wang, Y., and Xu, J. (2022). Two Novel Intronic Mutations in the CSF1R Gene in Two Families With CSF1R-Microglial Encephalopathy. Front Cell Dev Biol *10*, 902067. 10.3389/fcell.2022.902067.

127. Beerepoot, S., Verbeke, J., Plantinga, M., Nierkens, S., Pouwels, P.J.W., Wolf, N.I., Simons, C., and van der Knaap, M.S. (2024). Leukoencephalopathy with calcifications, developmental brain abnormalities and skeletal dysplasia due to homozygosity for a hypomorphic CSF1R variant: A report of three siblings. Am J Med Genet A *194*, e63800. 10.1002/ajmg.a.63800.

128. Leng, C., Lu, L., Wang, G., Zhang, Y., Xu, Y., Lin, X., Shen, N., Xu, X., Qun, S., Sun, M., and Ge, W. (2019). A novel dominant-negative mutation of the CSF1R gene causes adult-onset leukoencephalopathy with axonal spheroids and pigmented glia. Am J Transl Res *11*, 6093-6101.

129. Saitoh, B.Y., Yoshida, K., Hayashi, S., Yamasaki, R., Sato, S., Kamada, T., Suzuki, S.O., Murai, H., Iwaki, T., Ikeda, S.I., and Kira, J.I. (2013). Sporadic hereditary diffuse leukoencephalopathy with axonal spheroids showing numerous lesions with restricted diffusivity caused by a novel splice site mutation in the CSF1R gene. Clinical and Experimental Neuroimmunology *4*, 76-81. 10.1111/cen3.12076.

130. Lee, D., Yun, J.Y., Jeong, J.H., Yoshida, K., Nagasaki, S., and Ahn, T.B. (2015). Clinical evolution, neuroimaging, and volumetric analysis of a patient with a CSF1R mutation who presented with progressive nonfluent aphasia. Parkinsonism Relat Disord *21*, 817-820. 10.1016/j.parkreldis.2015.04.010.

131. Kawakami, I., Iseki, E., Kasanuki, K., Minegishi, M., Sato, K., Hino, H., Shibuya, K., Fujisawa, K., Higashi, S., Akiyama, H., et al. (2016). A family with hereditary diffuse leukoencephalopathy with spheroids caused by a novel c.2442+2T>C mutation in the CSF1R gene. J Neurol Sci *367*, 349-355. 10.1016/j.jns.2016.06.013.

132. Kindis, E., Simsek-Kiper, P.O., Kosukcu, C., Taskiran, E.Z., Gocmen, R., Utine, E., Haliloglu, G., Boduroglu, K., and Alikasifoglu, M. (2021). Further expanding the mutational spectrum of brain abnormalities, neurodegeneration, and dysosteosclerosis: A rare disorder with neurologic regression and skeletal features. Am J Med Genet A *185*, 1888-1896. 10.1002/ajmg.a.62179.
