# Supplementary material for: A strategy of microglia replacement alleviates microgliopathy in a CSF1R I794T hotspot mutation mouse model of CSF1R-related disorder
Source: Cell Rep Med. 2026 Feb 27;7(3):102641. doi: 10.1016/j.xcrm.2026.102641 (PMC13006430; doi:10.1016/j.xcrm.2026.102641)
Supplement: Document S2. Article plus supplemental information [file mmc2.pdf]

Cell Reports
Medicine

Article

A strategy of microglia replacement alleviates microgliopathy in a *CSF1R* I794T hotspot mutation mouse model of CSF1R-related disorder

Graphical abstract

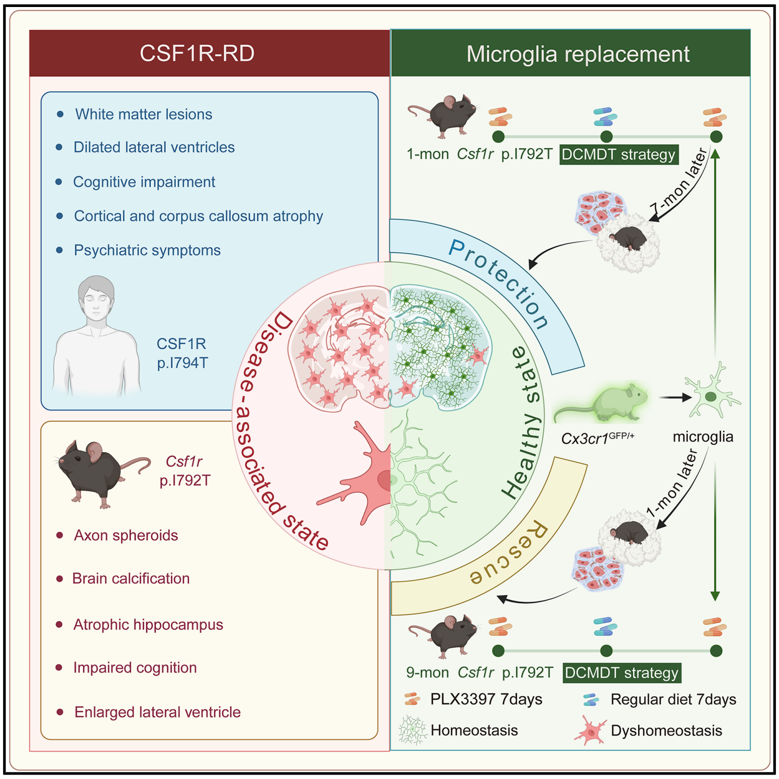

Authors

Xin Li, Banglian Hu, Chujun Wu, ..., Li Zhong, Zaiqiang Zhang, Honghua Zheng

Correspondence

yunzhang@xmu.edu.cn (Y.-W.Z.), zhongli@xmu.edu.cn (L.Z.), ttyy0142011@126.com (Z.Z.), honghua@xmu.edu.cn (H.Z.)

In brief

Li et al. define the clinical features of patients carrying the CSF1R p.I794T variant and establish a corresponding knockin mouse model. They show that *Csf1r*$^{I792T/+}$ microglia adopt a disease-associated state and that a microglial replacement strategy, DCMDT, mitigates cognitive and neuropathological deficits in CSF1R-related disorder.

Highlights

- CSF1R p.I794T is a global hotspot mutation underlying CSF1R-RD
- *Csf1r*$^{I792T/+}$ mice recapitulate key clinical and pathological features of CSF1R-RD
- *Csf1r*$^{I792T/+}$ microglia exhibit an activated and DAM-like phenotype
- A microglial replacement strategy attenuates the deficits in *Csf1r*$^{I792T/+}$ mice

Li et al., 2026, Cell Reports Medicine 7, 102641
March 17, 2026 © 2026 The Author(s). Published by Elsevier Inc.
https://doi.org/10.1016/j.xcrm.2026.102641

CellPress

Cell Reports Medicine

CellPress
OPEN ACCESS

Article

A strategy of microglia replacement alleviates microgliopathy in a *CSF1R* I794T hotspot mutation mouse model of CSF1R-related disorder

Xin Li,[1,6] Banglian Hu,[1,6] Chujun Wu,[2,6] Ziwei Wang,[1,6] Hanzheng Fan,[1,6] Xiaoyan Guan,[1] Sulan Xie,[1] Dadian Chen,[1] Xiaohua Huang,[3] Hao Sun,[1] Yanfang Li,[1] Xian Zhang,[1] Guojun Bu,[4] Zhanxiang Wang,[5] Yun-Wu Zhang,[1,*] Li Zhong,[1,*] Zaiqiang Zhang,[2,*] and Honghua Zheng[1,3,7,*]

[1]Xiamen Key Laboratory of Brain Center, The First Affiliated Hospital of Xiamen University, Fujian Key Laboratory of Neurodegenerative Disease and Aging Research, Institute of Neuroscience, School of Medicine, Xiamen University, Xiamen, Fujian 361102, China
[2]Department of Neurology, China National Clinical Research Center for Neurological Diseases, Beijing Tiantan Hospital, Capital Medical University, Beijing 100070, China
[3]Basic Medical Sciences, School of Medicine, Xiamen University, Xiamen, Fujian 361102, China
[4]Division of Life Science and State Key Laboratory of Nervous System Disorders, The Hong Kong University of Science and Technology, Clear Water Bay, Hong Kong, China
[5]Department of Neurosurgery, Xiamen Key Laboratory of Brain Center, The First Affiliated Hospital of Xiamen University, Xiamen, Fujian 361003, China
[6]These authors contributed equally
[7]Lead contact
*Correspondence: yunzhang@xmu.edu.cn (Y.-W.Z.), zhongli@xmu.edu.cn (L.Z.), ttyy0142011@126.com (Z.Z.), honghua@xmu.edu.cn (H.Z.)
https://doi.org/10.1016/j.xcrm.2026.102641

SUMMARY

The I794T hotspot mutation in the colony-stimulating factor 1 receptor (CSF1R) gene is associated with primary microgliopathy manifesting as leukoencephalopathy. In this study, we identify three Chinese probands harboring the CSF1R p.I794T variant and characterize their clinical and neuroimaging profiles. To elucidate disease mechanisms and explore therapeutic avenues, we generate a *Csf1r*$^{I792T/+}$ knockin mouse model that carries this human mutation. These *Csf1r*$^{I792T/+}$ mice exhibit hallmark features of CSF1R-related disorder (CSF1R-RD), including cognitive deficits, ventricular enlargement, reduced microglia, axonal spheroids, and demyelination. Transcriptomic analysis reveals that *Csf1r*$^{I792T/+}$ microglia adopt an activated and disease-associated microglia (DAM)-like phenotype. Crucially, we develop and test a microglia replacement strategy, termed "duplicate-cyclic microglial depletion for transplantation" (DCMDT), which significantly ameliorates neuropathological deficits in *Csf1r*$^{I792T/+}$ mice. Our findings highlight the pathological significance of the CSF1R p.I794T mutation and propose DCMDT as a promising therapeutic approach for neurodegenerative disorders driven by microglial dysfunction.

INTRODUCTION

The colony-stimulating factor 1 receptor (CSF1R) is a tyrosine kinase receptor predominantly expressed on microglia within the central nervous system (CNS). Autosomal dominant mutations in *CSF1R* cause a progressive leukodystrophy known as adult-onset leukoencephalopathy with axonal spheroids and pigmented glia (ALSP),[1–4] characterized by dementia with motor impairments and neuropsychiatric deficits. The disease has recently been reclassified under the broader term "CSF1R-related disorder" (CSF1R-RD).[5]

To date, more than 140 mutations in CSF1R have been implicated in CSF1R-RD, although the true prevalence may be underestimated due to insufficient genetic screening or incomplete penetrance.[6] One recurrent global hotspot mutation, c.2381T > C (p.I794T), is frequently associated with this disorder.[7] CSF1R plays a critical role in microglial survival, development, and homeostasis.[8] Mouse models with haploinsufficiency for *Csf1r* (*Csf1r*$^{+/-}$) mimic key neuropathological and behavioral features of ALSP,[9] including progressive microglial dysfunction, which has been recognized as a central driver of disease.[10]

Various therapeutic strategies have been explored in preclinical studies. Pharmacological interventions, such as glucocorticoid treatment, the CSF1R inhibitor PLX5622, and the microglial suppressor minocycline, have shown promise in mitigating the pathological features of *Csf1r*$^{+/-}$ mice.[11–14] Hematopoietic stem cell transplantation (HSCT) has also been applied in CSF1R-RD patients, with mixed outcomes, ranging from disease stabilization to continued progression,[15,16] highlighting the need to identify predictors of therapeutic response. More recently, microglial replacement has emerged as a potential therapeutic strategy. In particular, studies using *Csf1r*$^{\Delta FIRE/\Delta FIRE}$ mice, which model many aspects of human CSF1R-RD, demonstrated that replacing microglia can reverse key pathological features of

This is an open access article under the CC BY license (http://creativecommons.org/licenses/by/4.0/).

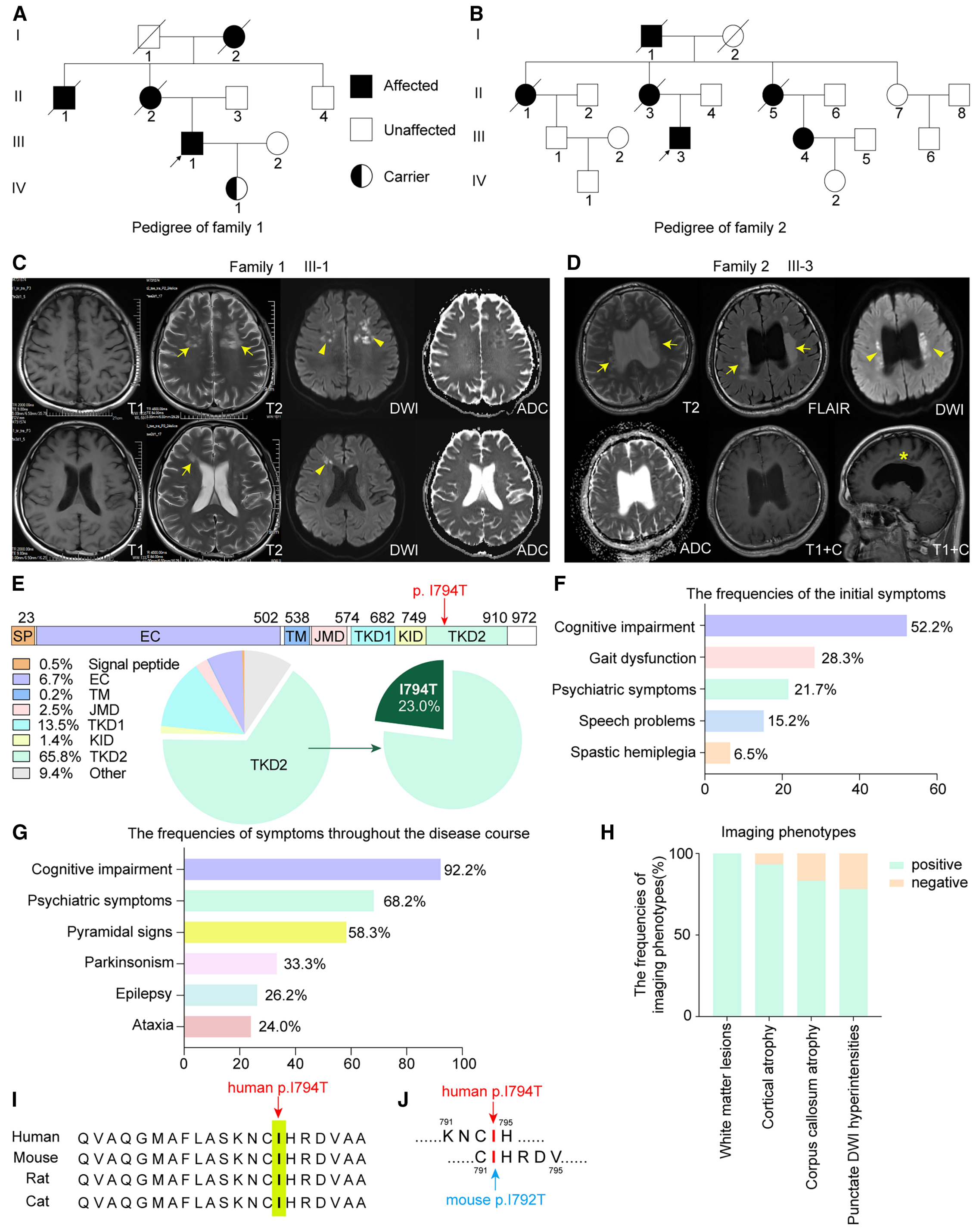

Figure 1. Clinical and imaging features of patients carrying the CSF1R p.I794T mutation

(A and B) Pedigrees of two families affected by CSF1R-RD carrying the CSF1R p.I794T mutation. Affected individuals are denoted by black-filled symbols. The probands are indicated with black arrows (III-1 in Family 1; III-3 in Family 2). Carriers of the CSF1R p.I794T mutation are represented by half-filled symbols. White-filled symbols denote unaffected individuals, while diagonal slashes indicate deceased individuals. Squares and circles represent males and females, respectively.

(C) Brain MRI of proband III-1 (Family 1) revealed periventricular WMLs in the frontal lobes (arrows), lateral ventricular enlargement, and cortical atrophy predominantly affecting the frontal and parietal lobes. DWI showed characteristic punctate hyperintensities (arrowheads) within the WMLs.

(D) MRI of proband III-3 (Family 2) showed confluent and patchy periventricular and deep WMLs (arrows), along with enlarged lateral ventricles and corpus callosum atrophy (asterisk). Punctate DWI hyperintensities were also observed (arrowheads).

(legend continued on next page)

CSF1R-RD.[17,18] These findings provide a rationale for further development of microglial replacement approaches to treat primary microgliopathies such as CSF1R-RD.

In this study, we identified three unrelated Chinese probands carrying the heterozygous CSF1R p.I794T variant and summarized the clinical and neuroimaging characteristics of this globally relevant mutation. To model the human disease, we generated *Csf1r* p.I792T knockin mice using homologous recombination. These mice exhibited microglial reduction and functional alterations, including increased proinflammatory signaling and phagocytosis, paralleling key human pathological features. Most importantly, we developed a strategy for therapeutic microglia replacement, termed "duplicate-cyclic microglial depletion for transplantation" (DCMDT). This approach substantially attenuated both behavioral deficits and neuropathologies in the $Csf1r^{I792T/+}$ model. Our findings confirm that the CSF1R p.I794T heterozygous mutation is sufficient to drive the pathological and clinical hallmarks of CSF1R-RD. Furthermore, we establish DCMDT as a viable and efficient therapeutic strategy for mitigating microglial dysfunction in CSF1R-RD and potentially other neurodegenerative diseases with a similar etiology.

RESULTS

CSF1R p.I794T is a global hotspot mutation underlying CSF1R-RD

We identified three Chinese probands from unrelated families, each harboring a heterozygous *CSF1R* p.I794T variant confirmed by genetic testing (Figures S1A–S1D). One proband had no known family history, while the other two belonged to pedigrees with clinical and radiological features characteristic of CSF1R-RD (Figures 1A and 1B), including magnetic resonance imaging (MRI) findings of leukoencephalopathy (Figures 1C and 1D).

The first proband (F1-III1) presented with memory impairment at age 35, followed by dysphasia 1 year later. Neurological examination revealed brisk tendon reflexes, and his Montreal Cognitive Assessment score was 24. Brain MRI demonstrated periventricular white matter lesions (WMLs) in the frontal lobes, dilated lateral ventricles, and a thinned corpus callosum. Punctate diffusion-weighted imaging (DWI) hyperintensities were evident within WMLs (Figure 1C). His condition progressively deteriorated, culminating in dysphagia, tetraparesis, and cachexia-related death at age 39. A strong family history was noted: his mother (F1-II2), maternal uncle (F1-II1), and maternal grandmother (F1-I2) all exhibited motor dysfunction, late-onset cognitive decline, and dysphasia in their 50s. The proband's daughter, now aged 14, also carries the CSF1R p.I794T variant but remains asymptomatic.

The second proband (F2-III3) developed right lower limb weakness at age 27, progressing to right-hand weakness within a year. Physical examination revealed hypertonia and brisk reflexes in all limbs. MRI revealed periventricular WMLs, thinning of the corpus callosum, dilated ventricles, and punctate DWI hyperintensities (Figure 1D). Computed tomography (CT) imaging did not show white matter calcification. The proband's maternal grandfather (F2-I1) developed ataxia at age 66. His mother (F2-II3) experienced cognitive impairment, and two maternal aunts (F2-II1, F2-II5) presented with ataxia in their 40s. The proband's cousin (F2-III4) is asymptomatic but showed ventricular dilation on MRI.

The third proband initially presented with psychiatric symptoms—anxiety, paranoia, and irritability at age 39. Over the next 3 years, he developed dysphasia, dysphagia, and cognitive decline. His mother had a history of epilepsy. Upon clinical assessment, his Mini-Mental State Examination score was 3, with signs of aphasia and bilateral pyramidal tract involvement. MRI showed dilated lateral ventricles, frontoparietal WMLs, and punctate DWI hyperintensities.

Since CSF1R mutations were first reported by Rademakers et al. in 2011 in 14 families,[19] the number of identified cases has steadily increased across diverse populations.[6,20,21] To date, over 222 distinct CSF1R mutations have been reported in 436 probands globally. Most mutations (~65.8%) are located in the intracellular tyrosine kinase domain 2 (TKD2) of CSF1R. Notably, the p.I794T missense variant alone accounts for 23.0% of TKD2 mutations, making it the most frequent single variant (Figure 1E). We comprehensively reviewed the clinical and imaging characteristics of 66 probands with heterozygous CSF1R p.I794T mutations, including 63 previously reported cases and the three described in the present study (Figures 1F–1H). Clinical and radiological details for 66 of these probands are summarized in Tables S1 and S2. A majority (77.3%, 51/66) originated from East Asia. Among 58 probands with available demographic data, the clinically recorded gender distribution was equal (1:1). The mean age at symptom onset was 40.8 ± 9.9 years (mean ± SD; range, 20–60 years), with male patients presenting at a mean age of 43.9 ± 8.8 years (range, 27–60 years) and female patients at 37.8 ± 10.3 years (range, 20–60 years). Disease progression was rapid, with an average clinical duration of 3.3 ± 1.0 years (range, 2–5 years; 3.0 ± 1.0 years in males, range, 2–4 years; 3.7 ± 1.2 years in females, range, 3–5 years), resulting in death at a mean age of 42.7 ± 4.1 years (range, 39–49 years; 42.3 ± 5.8 years in males, range, 39–49 years; 43.0 ± 3.0 years in females, range, 40–46 years). No significant difference was observed in the age of onset

(E) To date, 222 CSF1R mutations have been identified worldwide, with CSF1R p.I794T being a mutational hotspot (66/436 probands). Most mutations cluster within the intracellular tyrosine kinase domain 2 (TKD2) of CSF1R.
(F) Frequencies of initial clinical symptoms in patients with the CSF1R p.I794T mutation.
(G) Frequencies of symptoms throughout the disease course in affected individuals with the CSF1R p.I794T mutation.
(H) Frequencies of characteristic imaging features among patients with the CSF1R p.I794T mutation.
(I) The p.I794T residue in CSF1R is evolutionarily conserved across vertebrate species.
(J) The human *CSF1R* p.I794T variant corresponds to *Csf1r* p.I792T in mice.
EC, extracellular domain; TM, transmembrane domain; JMD, juxtamembrane domain; TKD1/2, tyrosine kinase domain 1/2.

(40.8 years vs. 41.9 years, $p = 0.270$) between patients harboring the p.I794T mutation (Tables S1 and S2) and those with other mutations (Table S3). Initial neurological symptoms were available for 46 individuals: cognitive impairment (52.2%) was the most common, followed by gait dysfunction (28.3%), psychiatric symptoms (21.7%), speech problems (15.2%), and spastic hemiplegia (6.5%) (Figure 1F). Over the disease course, cognitive impairment was nearly universal (92.2%), accompanied by psychiatric symptoms (68.2%), pyramidal signs (58.3%), parkinsonism (33.3%), epilepsy (26.2%), and ataxia (24.0%) (Figure 1G).

Neuroimaging data revealed that all patients exhibited WMLs, with 93.3% also showing cortical atrophy, preferentially affecting the frontal and parietal lobes (Figure 1H). Corpus callosum atrophy and punctate DWI hyperintensities were present in 83.3% and 78.3% of patients, respectively (Figure 1H). Among 20 patients who underwent CT scans, white matter calcifications were detected in 11. The p.I794 residue in CSF1R is highly conserved across vertebrate species (Figure 1I). In mice, the corresponding mutation is *Csf1r* p.I792T (Figure 1J), enabling the development of a relevant murine model for mechanistic studies and therapeutic intervention in CSF1R-RD.

The *Csf1r*$^{I792T/+}$ mouse model recapitulates clinical and pathological features of CSF1R-RD

Studies have previously reported that single-allele heterozygous mutations in CSF1R have been clinically linked to CSF1R-RD.[7,19–21] To investigate the pathogenic mechanisms and explore potential therapeutic strategies, we generated a knockin mouse model harboring the *Csf1r* p.I792T variant via homologous recombination-based gene editing (Figures S2A–S2C). We first assessed CSF1R expression in these mice. While CSF1R transcript levels remained unchanged, protein levels were markedly reduced in the brains of *Csf1r*$^{I792T/+}$ mice relative to control littermates (Figures 2A–2C). This reduction was also observed in primary microglia isolated from neonatal *Csf1r*$^{I792T/+}$ mice (Figures S2D–S2F). Homozygous *Csf1r*$^{I792T/I792T}$ mice displayed perinatal lethality similar to *Csf1r*$^{-/-}$ mice, with most failing to survive beyond 24 h.[8] Moreover, heterozygous *Csf1r*$^{I792T/+}$ mice exhibited significantly reduced overall survival compared to wild-type (WT) littermates (Figure 2D).

Given that cognitive decline is often the initial clinical symptom in CSF1R-RD patients with the p.I794T variant, we next examined whether *Csf1r*$^{I792T/+}$ mice exhibit analogous cognitive impairments. In spontaneous alternation T-maze tests, 9-month-old *Csf1r*$^{I792T/+}$ mice but not 3- or 6-month-old *Csf1r*$^{I792T/+}$ mice showed significant working memory deficits compared with age-matched *Csf1r*$^{+/+}$ littermates (Figures 2E–2G). To assess hippocampal synaptic function, long-term potentiation (LTP) was induced by high-frequency stimulation (HFS) in CA1 regions of 6-month-old *Csf1r*$^{I792T/+}$ and *Csf1r*$^{+/+}$ mice. The amplitude of field excitatory postsynaptic potential (fEPSP) was substantially reduced in *Csf1r*$^{I792T/+}$ mice (Figures 2E–2G). Spatial learning and memory were further evaluated using the Morris water maze (MWM). At 3 and 6 months, escape latency did not differ between genotypes (Figures 2J and 2K). However, 9-month-old *Csf1r*$^{I792T/+}$ mice showed significantly longer escape latencies during the 6-day training period (Figure 2L). On test day (day 7), swim speeds were comparable (Figures 2M and 2N), yet *Csf1r*$^{I792T/+}$ mice still demonstrated prolonged escape latency (Figure 2O), spent less time in the target quadrant (Figure 2P), and had fewer platform crossings (Figure 2Q). Emotional performance was also evaluated using the open field test (Figures S2G–S2J) and the elevated plus maze test (Figures S2K–S2N), revealing no anxiety-like behaviors at 3, 6, 9, or 12 months. Together, these findings demonstrate that the *Csf1r* p.I792T variant leads to age-dependent cognitive impairments.

MRI T2 imaging revealed hippocampal atrophy and lateral ventricle (LV) enlargement in 9-month-old *Csf1r*$^{I792T/+}$ mice (Figures 3A–3C). Immunohistochemical and immunofluorescent staining for phosphorylated neurofilament heavy chain (phospho-NFH), a marker of axonal damage,[22] revealed axonal spheroids in the brains of these mice (Figures 3D–3G). Alizarin red S staining also detected brain calcifications at 9 months (Figures 3H and 3I). Furthermore, the G-ratio of myelin sheaths was significantly increased, indicating demyelination, a result corroborated by western blot analysis of myelin basic protein (MBP) expression (Figures 3J–3M). Electron microscopy revealed decreased synaptic density in the brains of *Csf1r*$^{I792T/+}$ mice (Figures 3N and 3O). Collectively, these results demonstrate that the heterozygous 9-month-old *Csf1r* p.I792T variant is sufficient to recapitulate the core clinical and pathological features of CSF1R-RD.

The *Csf1r* p.I792T variant reduces microglial numbers and promotes an activated and disease-associated microglia-like phenotype

Given previous findings of microgliosis in *Csf1r*$^{+/-}$ mice[9] and decreased microglial density in *Csf1r*$^{E631K/+}$ mice,[23] we examined whether the *Csf1r* p.I792T variant affects microglial populations. Quantification of Iba1-positive microglia (Iba1^{+}) revealed significantly reduced microglial density in the hippocampus and cortex of *Csf1r*$^{I792T/+}$ mice relative to WT controls (Figures 4A–4C). Morphological analysis showed marked reductions in microglial process length and branching in both the cortex and hippocampus of *Csf1r*$^{I792T/+}$ mice (Figure 4D), indicating a shift toward a dystrophic and dysfunctional phenotype.

As CSF1R-RD is now classified as a primary CNS microgliopathy, we sought to further characterize the molecular consequences of the *Csf1r* p.I792T variant on microglial function. Whole-transcriptome RNA sequencing (RNA-seq) was performed on microglia isolated from postnatal day 0–3 *Csf1r*$^{+/+}$ and *Csf1r*$^{I792T/+}$ mice. Differentially expressed genes (DEGs) were identified and hierarchically clustered based on established analytical frameworks.[24–26] Heatmap visualization revealed that DEGs were enriched in pathways related to the cell cycle, phagosome formation, inflammation, and cellular homeostasis (Figure S3A). Kyoto Encyclopedia of Genes and Genomes (KEGG) pathway analysis of gene sets from 0- to 3-day-old *Csf1r*$^{+/+}$ and *Csf1r*$^{I792T/+}$ microglia identified significant enrichment of genes involved in phagosome activity, complement and coagulation cascades, chemokine and Toll-like receptor signaling, antigen presentation, cytokine-cytokine receptor interaction, and tumor necrosis factor signaling (Figure S3B). Gene set enrichment analysis (GSEA) further demonstrated

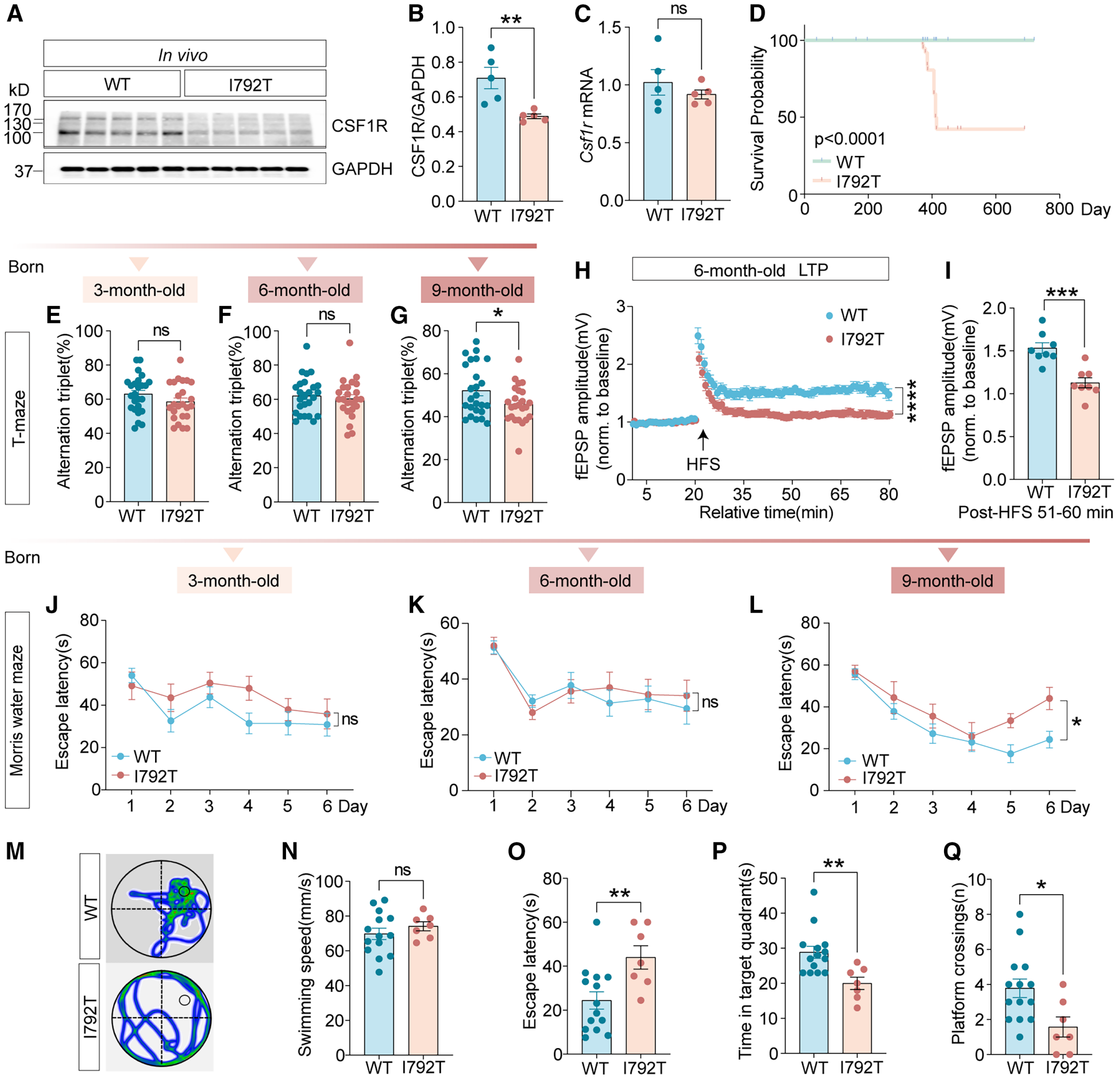

Figure 2. Impaired cognitive performance in 9-month-old *Csf1r* $^{I792T/+}$ mice

The *Csf1r* $^{I792T/+}$ (I792T) mouse model was generated via homologous recombination, introducing the I792T point mutation into the murine *Csf1r* gene.

(A–C) CSF1R expression in the cortex of 9-month-old *Csf1r* $^{I792T/+}$ mice was analyzed via western blot (A and B) and qPCR (C), $n = 5$ per group. Unpaired two-tailed Student's t test.

(D) *Csf1r* $^{I792T/+}$ mice exhibited significantly reduced survival compared to wild-type controls ($n = 50$ per group).

(E–G) Working memory performance at 3, 6, and 9 months was assessed using the T-maze spontaneous alternation task. *Csf1r* $^{I792T/+}$ mice ($n = 24$) demonstrated progressive deficits in spontaneous alternation relative to control littermates ($n = 25$).

(H) LTP was evaluated in hippocampal CA1 region from 6-month-old mice. Time-series plots show fEPSPs following HFS.

(I) Quantification of fEPSP amplitude averaged over the final 10 min of LTP recording. WT ($n = 5$ mice, 10 slices), *Csf1r* $^{I792T/+}$ ($n = 5$ mice, 8 slices).

(J–L) Spatial learning and memory were assessed using the MWM at 3, 6, and 9 months. Escape latency over a 6-day training period revealed impaired learning in 9-month-old *Csf1r* $^{I792T/+}$ mice.

(M) Representative swim paths from 9-month-old mice show impaired spatial navigation in *Csf1r* $^{I792T/+}$ mice.

(N–Q) Quantitative metrics from MWM testing: swimming speed (N), escape latency (O), time spent in the target quadrant (P), and platform crossings (Q) showed significant deficits in 9-month-old *Csf1r* $^{I792T/+}$ mice compared to controls. WT, $n = 14$; *Csf1r* $^{I792T/+}$, $n = 7$.

Unpaired two-tailed Student's t test. Data are expressed as mean ± SEM. *$p < 0.05$, **$p < 0.01$, ***$p < 0.001$, ****$p < 0.0001$; ns, not significant. I792T, *Csf1r* $^{I792T/+}$.

downregulation of cell cycle-associated genes and upregulation of inflammatory and microglial activation signatures in 0- to 3-day-old *Csf1r* $^{I792T/+}$ microglia (Figures S3C–S3E).

Given that microglia from 0- to 3-day-old mice might not reflect their states in adult mice and to better reveal how microglia dysregulation in adult mice accounted for progressive leukoencephalopathy, we isolated microglia from 9-month-old WT or *Csf1r* $^{I792T/+}$ mice by fluorescence-activated cell sorting (FACS) and subjected them to bulk RNA-seq. Heatmap visualization revealed that DEGs in 9-month-old WT or

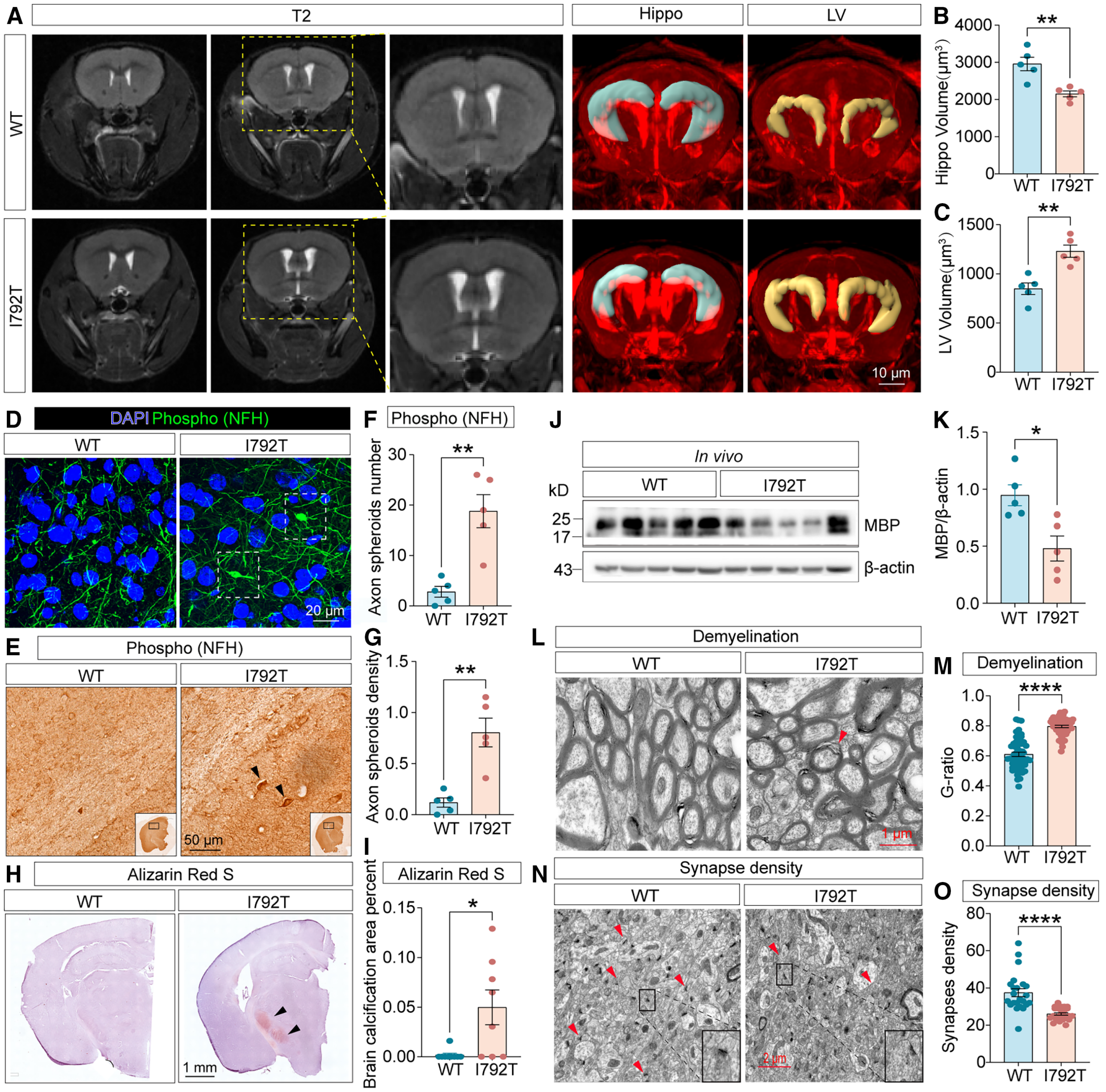

Figure 3. The 9-month-old *Csf1r* $^{I792T/+}$ mouse model recapitulates key pathological and imaging features of CSF1R-RD

(A) T2-weighted MRI of 9-month-old I792T mice showed enlarged LVs. Three-dimensional reconstructions highlight the LV (yellow) and hippocampus (blue). Scale bar, 10 μm.

(B) Decreased hippocampal volumes examined in 9-month-old *Csf1r* $^{I792T/+}$ mouse brains, *n* = 5 mice per group.

(C) Increased LV volumes observed in 9-month-old *Csf1r* $^{I792T/+}$ mice, *n* = 5 mice per group.

(D) Axonal spheroids in 9-month-old *Csf1r* $^{I792T/+}$ mouse brains were observed using immunofluorescent labeling of phospho-NFH (green, white squares). Scale bar, 20 μm.

(E) Axonal spheroids in 9-month-old *Csf1r* $^{I792T/+}$ mouse brains were observed using immunohistochemical labeling of phospho-NFH (dark arrowheads). Scale bar, 50 μm.

(F and G) Quantification of the number (F) and density (G) of axonal spheroids, *n* = 3 mice per group.

(H) Alizarin red S staining revealed cerebral calcification in 9-month-old *Csf1r* $^{I792T/+}$ mice (black arrowheads). Scale bar, 1 mm.

(I) Quantification of cerebral calcifications, *n* = 3 mice per group.

(J) Representative western blots of MBP expression in 9-month-old *Csf1r* $^{+/+}$ or *Csf1r* $^{I792T/+}$ mouse brains.

(K) MBP protein assessed using densitometry relative to β-actin, *n* = 5 mice per group.

(L) Transmission electron microscopy demonstrated demyelination in 9-month-old *Csf1r* $^{I792T/+}$ mice. Scale bar, 1 μm.

(M) The G-ratio (ratio of inner to outer myelin sheath diameter) was significantly increased in 9-month-old *Csf1r* $^{I792T/+}$ mice, *n* = 4 mice per group.

(N) Ultrastructural analysis of the synapse in 9-month-old *Csf1r* $^{I792T/+}$ mice by electron microscopy. Scale bar, 2 μm.

(O) The synaptic density was reduced in 9-month-old *Csf1r* $^{I792T/+}$ mouse brains, *n* = 4 mice per group.

Data are presented as mean ± SEM. Unpaired two-tailed Student's *t* test. *$p < 0.05$, **$p < 0.01$, ****$p < 0.0001$. WT, wild-type; I792T, *Csf1r* $^{I792T/+}$.

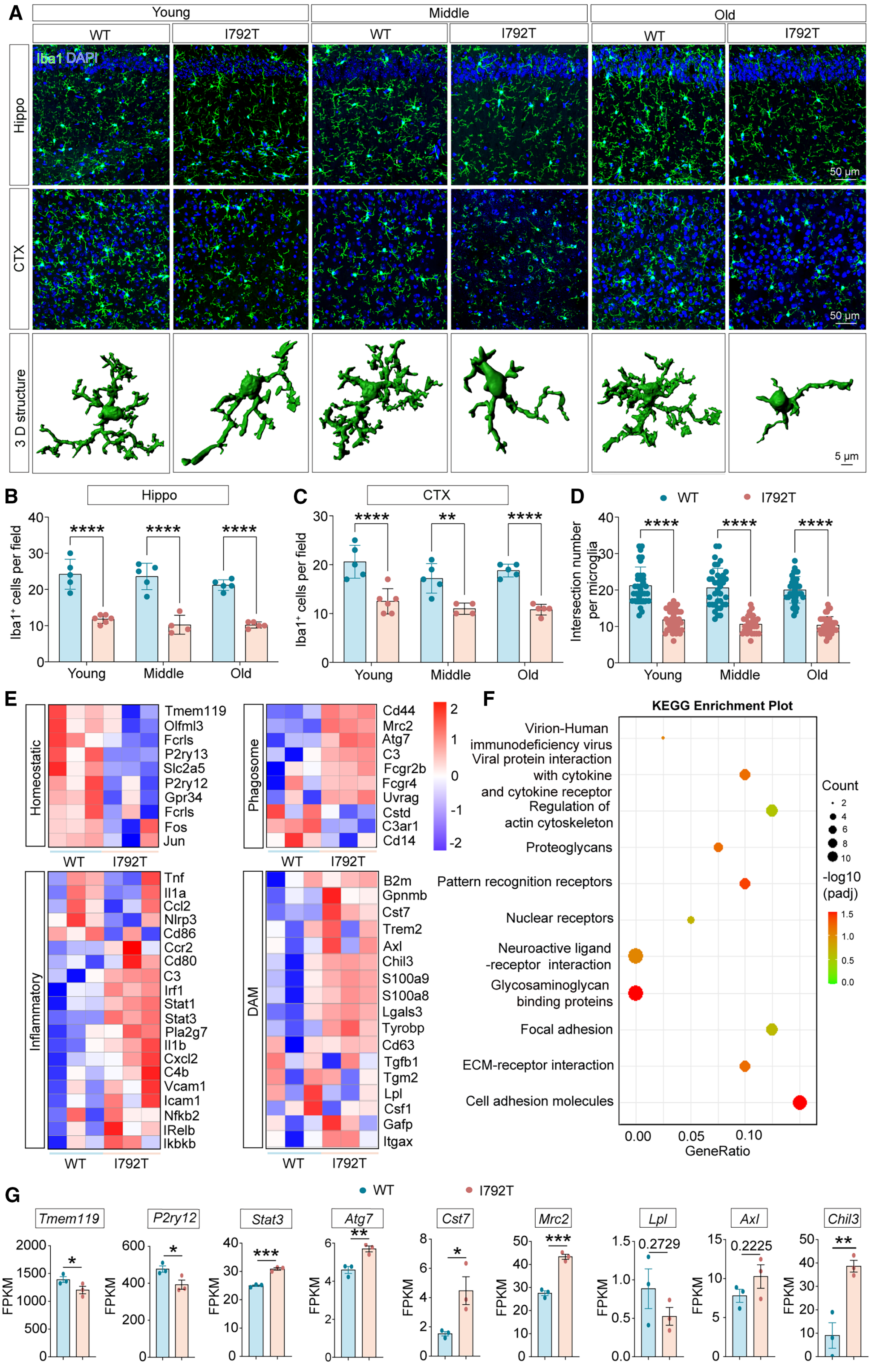

(legend on next page)

Csf1r$^{I792T/+}$ microglia were enriched in pathways related to the disease-associated microglia (DAM), phagosome, inflammation, and cellular homeostasis (Figure 4E). KEGG pathway analysis of gene sets from those *Csf1r*$^{+/+}$ and *Csf1r*$^{I792T/+}$ microglia identified significant enrichment of genes involved in pattern recognition receptors, cytokine-cytokine receptor interaction, and cell adhesion molecules (Figure 4F). Furthermore, the expression of representative inflammatory, remodeling, phagosome, or DAM genes, including *Stat3*, *Mrc2*, *Chil3*, *Atg7*, and *Cst7* levels, were increased, while the homeostatic genes *Tmem119* and *P2ry12* were significantly reduced in 9-month-old *Csf1r*$^{I792T/+}$ microglia relative to WT controls (Figure 4G), suggesting an activated and DAM-like phenotype in microglia from 9-month-old *Csf1r*$^{I792T/+}$ mice relative to WT controls.

Given that elevated early expression of *Csf2* and *Csf3* has been reported in 7-week-old *Csf1r*$^{+/-}$ mice and in CSF1R-RD patients,[27,28] we sought to determine whether a similar dysregulation occurs in 9-month-old *Csf1r*$^{I792T/+}$ mice. Unexpectedly, *Csf2* and *Csf3* levels were significantly reduced or exhibited a downward trend in 9-month-old *Csf1r*$^{I792T/+}$ mouse brains compared to WT controls (Figure S4). Furthermore, given that DAM are a hallmark of neurodegeneration,[29–31] we assessed the expression of DAM-related genes, including *P2ry12*, *Tmem119*, *Axl*, *H2-Ab1*, *Lpl*, and *Cst7* in 9-month-old *Csf1r*$^{I792T/+}$ mouse brains by quantitative reverse-transcription PCR. While *P2ry12*, *Axl*, *Lpl*, and *Cst7* levels remained unchanged, *Tmem119* and *H2-Ab1* expression was significantly reduced in 9-month-old *Csf1r*$^{I792T/+}$ mouse brains relative to WT controls (Figure S4), further indicating an activated and DAM-like phenotype of microglia in *Csf1r*$^{I792T/+}$ mouse brain.

Collectively, these results indicate that the *Csf1r* p.I792T variant reduces microglial number and induces a shift toward an activated, DAM-like phenotype. This dysfunctional microglial state likely disrupts CNS homeostasis, contributing to the structural and functional brain abnormalities characteristic of progressive leukoencephalopathy.

Establishment of the DCMDT strategy for brain-wide microglial transplantation in *Csf1r*$^{I792T/+}$ mice

Given the reduction and dysfunction of microglia in *Csf1r*$^{I792T/+}$ mice, we next investigated whether microglial replacement could reverse the behavioral and pathological deficits associated with this CSF1R-RD model. Building on our recent development of an efficient microglial depletion and transplantation method that restored pathological phenotypes in an Alzheimer's disease mouse model,[32] we optimized transplantation conditions for *Csf1r*$^{I792T/+}$ mice. One-month-old *Csf1r*$^{I792T/+}$ mice were administered PLX3397 (600 mg/kg, orally) for 3, 5, or 7 days to deplete resident microglia,[33] followed by a 1-week recovery period on a regular diet to allow for microglial repopulation (Figure S5A). PLX3397 treatment resulted in near-complete microglial depletion at all time groups (Figures S5B–5D). However, the repopulation capacity of endogenous microglia in *Csf1r*$^{I792T/+}$ mice was significantly impaired compared to WT (*Csf1r*$^{+/+}$) controls, particularly in the cortex and in the 7-day treatment group (Figure S5C).

To assess engraftment efficiency, we transplanted GFP$^+$Iba1$^+$ microglia derived from *Cx3cr1*$^{GFP/+}$ mice into 1-month-old *Csf1r*$^{I792T/+}$ brains immediately following 7 days of PLX3397 treatment (Figure S6A). One month post-transplantation, only 61.2% of the transplanted microglia successfully populated the brain (Figures S6B–S6F). To enhance engraftment, we applied a two-cycle depletion protocol: one-month-old *Csf1r*$^{I792T/+}$ mice received two consecutive 7-day PLX3397 treatments (600 mg/kg), separated by a 7-day regular diet to deplete resident microglia (Figure 5A). GFP$^+$Iba1$^+$ microglia from *Cx3cr1*$^{GFP/+}$ mice were then transplanted into the brain. Immunofluorescent staining (Figures 5B–5D) and GFP$^+$-based flow cytometry (Figures 5E and 5F) revealed robust and widespread colonization (>86%), with the exception of marginal brain regions distal to the injection site.

Thus, for the *Csf1r*$^{I792T/+}$ mouse model of CSF1R-RD, we developed DCMDT, a simple strategy for microglial depletion that can be used to achieve efficient microglia replacement.

Preventive microglial replacement protects against cognitive and pathological deficits in 1-month-old *Csf1r*$^{I792T/+}$ mice

To determine whether DCMDT-based microglial transplantation could prevent the development of cognitive and pathological symptoms, 1-month-old *Csf1r*$^{I792T/+}$ mice underwent two rounds of PLX3397 treatment followed by transplantation of GFP$^+$ microglia (Figure 6A). Behavioral and pathological assessments were conducted 7 months later, at an age when cognitive deficits typically emerge. Flow cytometry (Figures 6B and 6C) and immunofluorescence (Figures 6D–6F) confirmed widespread engraftment of GFP$^+$ microglia (82.9%). Transplanted mice exhibited significant improvements in cognitive performance, as demonstrated by higher nesting scores (Figures 6G and 6H) and improved performance in T-maze tests (Figure 6I).

Figure 4. Adult *Csf1r*$^{I792T/+}$ microglia are reduced and show an activated and DAM-like phenotype

(A) Representative Iba1 (green) immunofluorescent images and corresponding 3D reconstructions of microglia in the hippocampus and cortex of *Csf1r*$^{+/+}$(WT) and *Csf1r*$^{I792T/+}$ (I792T) mice at young (3–5 months), middle (10–14 months), and old (20–24 months) stages. Microglia in *Csf1r*$^{I792T/+}$ brains exhibit dystrophic morphology, characterized by enlarged, rounded soma and reduced branching. Scale bars: 50 and 5 μm.

(B and C) Quantification revealed a significant reduction in Iba1$^+$ microglia in both the hippocampus (B) and cortex (C) of *Csf1r*$^{I792T/+}$ mice.

(D) Sholl analysis demonstrated markedly decreased process complexity in *Csf1r*$^{I792T/+}$ microglia compared to WT. Branch intersections were quantified at 5-μm intervals from the soma.

(E) Heatmap of DEGs from bulk RNA-seq of microglia isolated from 9-month-old *Csf1r*$^{+/+}$ or *Csf1r*$^{I792T/+}$ mice.

(F) KEGG pathway enrichment analysis of DEGs revealed the 11 significantly altered pathways associated with activated microglia.

(G) Representative expression of homeostatic, inflammatory, phagosome, and DAM genes from bulk RNA-seq of microglia isolated from 9-month-old *Csf1r*$^{+/+}$ or *Csf1r*$^{I792T/+}$ mice. FPKM, fragments per kilobase of transcript per million mapped fragments.

Data are presented as mean ± SEM. $n = 4\sim6$ mice per group. Unpaired two-tailed Student's *t* test. *$p < 0.05$, **$p < 0.01$, ***$p < 0.001$, ****$p < 0.0001$. WT, wild-type; I792T, *Csf1r*$^{I792T/+}$.

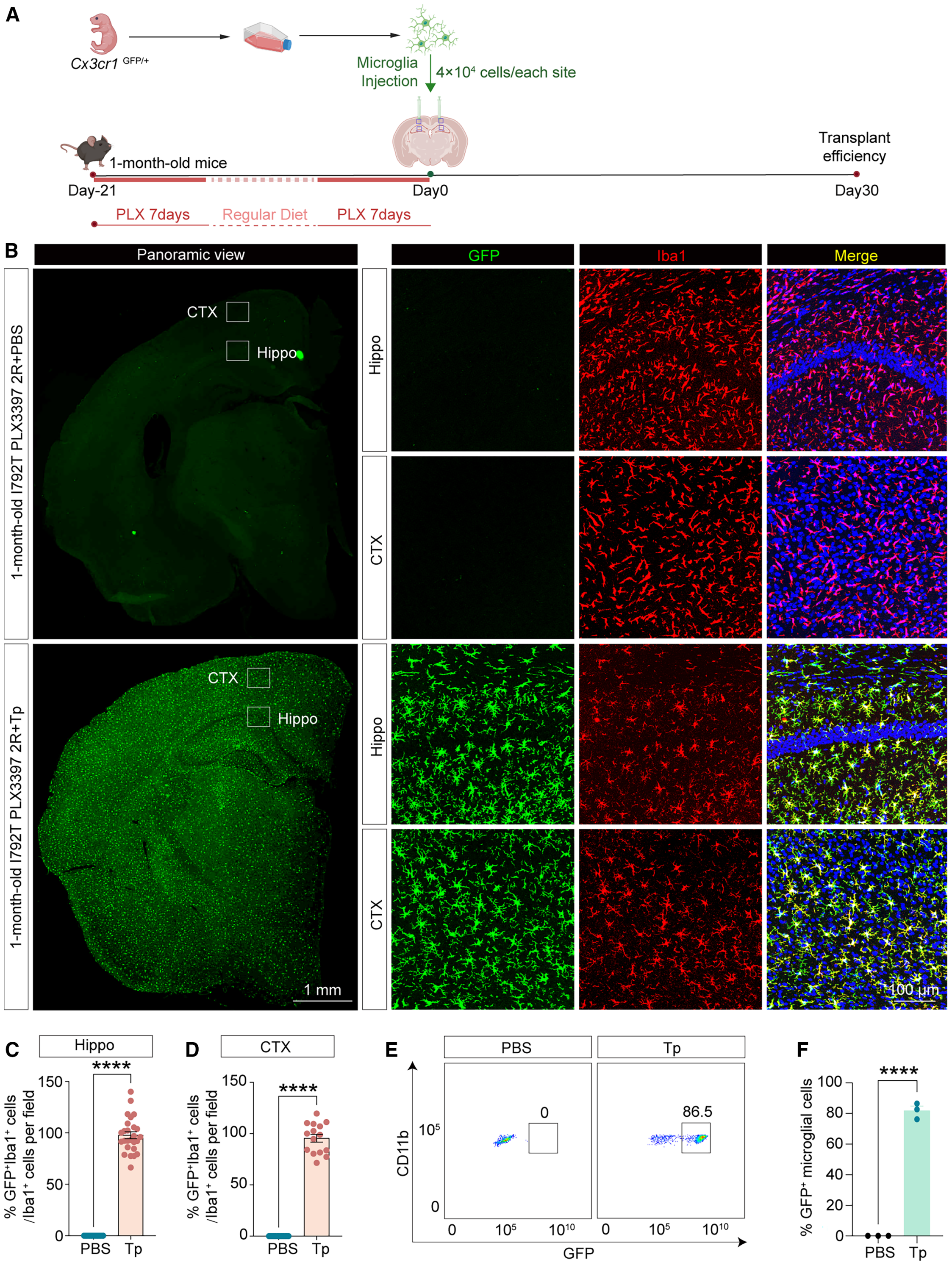

Figure 5. Efficient replacement of endogenous microglia by transplantation in 1-month-old *Csf1r* $^{I792T/+}$ mice

(A) Schematic of the microglia depletion and transplantation protocol. Endogenous microglia in 1-month-old *Csf1r*$^{I792T/+}$ mice were ablated via two 7-day cycles of oral PLX3397 administration.

(B) Immunofluorescent staining demonstrated robust engraftment of GFP$^+$Iba1$^+$ donor microglia from *Cx3cr1*$^{GFP/+}$ mice across nearly the entire brain of *Csf1r*$^{I792T/+}$ recipients. Scale bars: 1 mm and 100 μm.

(C) Quantification of the percentage of GFP$^+$Iba1$^+$ microglia relative to Iba1$^+$ cells per high field in the hippocampus of *Csf1r*$^{I792T/+}$ mouse brains.

(legend continued on next page)

In the MWM, microglia-transplanted *Csf1r* $^{I792T/+}$ (Tp) mice showed significantly reduced escape latencies during the 4-day training session compared to non-transplanted controls (I792T) (Figure 6J). Swim speeds were comparable across groups during the probe trial (Figures 6K and 6L). However, Tp mice exhibited shorter escape latencies (Figure 6M), spent more time in the target quadrant (Figure 6N), and had increased platform crossings (Figure 6O), all indicating improved spatial memory. Importantly, western blot analysis revealed increased MBP in transplanted mice (Figures 6P and 6Q), suggesting prevention of demyelination. Transplantation also ameliorated key pathological hallmarks of CSF1R-RD, including axonal spheroids and cerebral calcification (Figures 6R–6U). Together, these findings demonstrate that early microglial replacement via DCMDT can prevent or delay the onset of cognitive and neuropathological deficits in young *Csf1r*$^{I792T/+}$ mice.

Therapeutic microglial replacement reverses cognitive and pathological deficits in 9-month-old *Csf1r*$^{I792T/+}$ mice

Given the protective effects of preventive microglial replacement and the adult-onset nature of CSF1R-RD, we next assessed whether therapeutic microglial transplantation could reverse established cognitive and pathological deficits in 9-month-old *Csf1r*$^{I792T/+}$ mice. We applied the DCMDT protocol to 9-month-old *Csf1r*$^{I792T/+}$ mice (Figure 7A). Following treatment, transplanted GFP$^+$Iba1$^+$ microglia robustly colonized nearly the entire brain, as confirmed by immunofluorescence (Figures 7B–7D). Behavioral deficits were significantly improved: nesting performance (Figures 7E and 7F) and T/Y-maze scores (Figures 7G and 7H) were restored. While the increase in MBP levels did not reach statistical significance, a trend toward demyelination recovery was observed (Figures 7I and 7J). Furthermore, axonal spheroids and cerebral calcifications were ameliorated in transplanted mice (Figures 7K–7N).

These results indicate that even at advanced disease stages, microglial replacement using the DCMDT strategy can significantly reverse cognitive impairments and key pathological features in the *Csf1r*$^{I792T/+}$ model of CSF1R-RD. These findings highlight the translational potential of therapeutic microglial transplantation for adult-onset microgliopathies.

To determine whether transplanted microglia re-establish a functional phenotype in *Csf1r* $^{I792T/+}$ mice and to elucidate the mechanisms by which microglial transplantation mitigates microgliopathy, we performed RNA-seq analysis on mouse brains (Figure S7A). Strikingly, *Csf1r* $^{I792T/+}$ mice exhibited downregulation of genes associated with inflammation (such as *ApoE*, *Tlr2*, and *Tgfbr1*), axonal regeneration, and memory-related processes (such as *Bex1*, *Bex2*, *Cckbr*, and *Rab3b*) compared to *Csf1r*$^{+/+}$ controls.

To further explore the transcriptional impact of microglial transplantation, we conducted weighted gene co-expression network analysis (WGCNA) and KEGG pathway enrichment on RNA-seq data from the three experimental groups. WGCNA identified two key modules (Blue and Turquoise) that strongly correlated with pathological severity and responded to transplantation (Figure S7B). KEGG analysis revealed that genes within the Blue module were enriched in pathways related to "cellular senescence," "cell cycle," and "virus infection" (Figure S7C), while those in the Turquoise module were associated with "axon guidance," "pathways of neurodegeneration," and "multiple diseases" processes (Figure S7D). These data suggest that microglial transplantation may exert protective effects by reprogramming dysfunctional gene networks implicated in CSF1R-RD.

Notably, transplantation of WT microglia significantly restored or exhibited a recovery trend in the expression of *Csf2/3* and DAM genes, including *Tmem119*, *Cst7*, and *H2-Ab1* in *Csf1r* $^{I792T/+}$ mice (Figure S7E), indicating a transcriptional reversion toward the WT profile. Together, these findings indicate that DCMDT-based microglial transplantation effectively reverses both cognitive impairments and neuropathological hallmarks in the *Csf1r*$^{I792T/+}$ mouse model, highlighting its potential as a therapeutic strategy for CSF1R-RD.

DISCUSSION

CSF1R-RD is increasingly recognized as a globally prevalent condition,[6,20] with numerous variants and cases reported in diverse populations.[7,34–39] A comprehensive review by Dulski et al. detailed the clinical heterogeneity of CSF1R-RD, noting its occurrence across a wide age range, from early onset (<18 years) to late onset (≥18 years), yet sharing a similarly progressive neurodegenerative course.[39] Common clinical manifestations include spasticity, rigidity, cognitive decline, and eventual loss of voluntary movement and speech. Pneumonia and secondary infections are the leading causes of death in affected individuals. To date, over 500 cases have been documented worldwide.[39] Among known mutations, the c.2381T > C (p.I794T) variant has emerged as a mutational hotspot, particularly prevalent in Chinese patients.[7] The high frequency of this variant underscores the urgent need for targeted therapies addressing shared pathogenic mechanisms in CSF1R-RD.

In the present study, we report three probands carrying the CSF1R p.I794T variant. By reviewing all published CSF1R mutations, we confirm that p.I794T is one of the most common disease-causing variants globally. Our analysis of clinical and neuroimaging data from CSF1R p.I794T patients reveals that cognitive impairment is the most frequent initial symptom. In parallel, we generated a *Csf1r*$^{I792T/+}$ knockin mouse model via homologous recombination. This model replicates the clinical and pathological features of CSF1R-RD with early synaptic dysfunction evident at 6 months, preceding detectable cognitive deficits at 9 months, a timeline that aligns with human disease progression. Thus, this model provides a reliable platform for dissecting

(D) Quantification of the percentage of GFP$^+$Iba1$^+$ microglia relative to Iba1$^+$ cells per high field in the cortex of *Csf1r*$^{I792T/+}$ mouse brains.
(E) Representative flow cytometry dot plots showing GFP$^+$CD11b$^+$ donor-derived microglia in transplanted *Csf1r* $^{I792T/+}$ brains.
(F) Transplanted microglia accounted for up to 86.5% of total CD11b$^+$ microglia.
Data are presented as mean ± SEM, $n = 3$ mice per group. ****$p < 0.0001$. Tp, *Csf1r*$^{I792T/+}$ mice transplanted with microglia.

CellPress
OPEN ACCESS

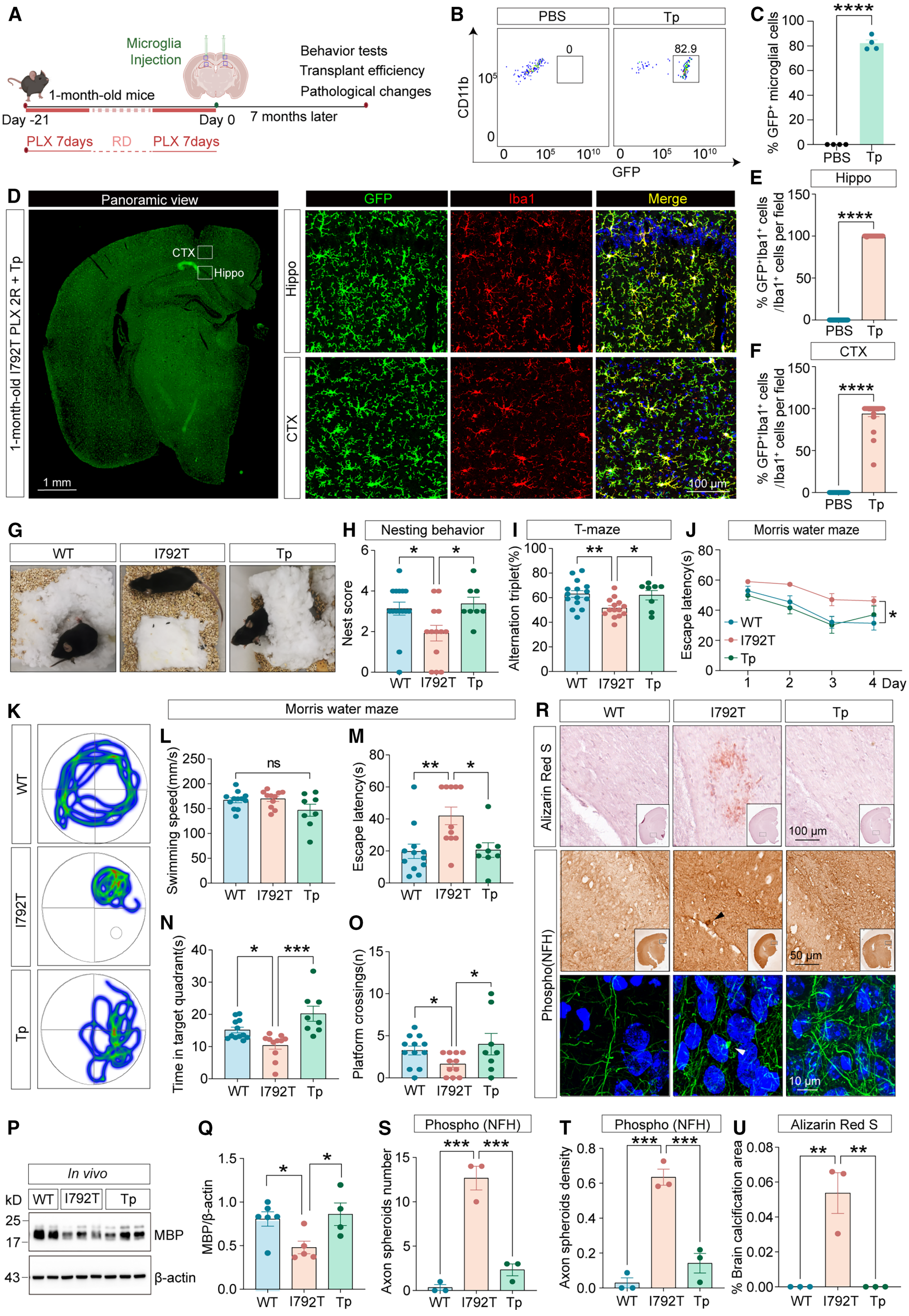

(legend on next page)

the role of microglial dysfunction in CSF1R-RD pathogenesis. The underlying cause of the approximately 50% survival rate observed in *Csf1r*$^{I792T/+}$ mice remains unclear. Interestingly, we found that the surviving mice show significantly reduced CSF1R protein levels at an aged time point (20–24 months, data not shown). This suggests that a subset of mice might possess compensatory mechanisms that allow them to tolerate the chronically low level of CSF1R signaling.

Consistent with findings by Biundo et al., who demonstrated that heterozygous deletion of *Csf1r* in microglia but not in neurons causes neurodegeneration,[10] our results reinforce the classification of CSF1R-RD as a primary microgliopathy. While earlier studies have described microgliosis in *Csf1r*$^{+/-}$ mice and microglial deletion (M*Csf1r*^{het}) mice,[9,10,28] decreased microglial density has also been reported in inducible *Csf1r*$^{+/-}$ models,[11] *Csf1r*$^{E631K/+}$ mice,[23] *Csf1r* mutant zebrafish,[40] and *Csf1r*$^{+/-}$ mice,[12] as well as in CSF1R-RD patients.[9,41–44] Consistent with these studies, our data demonstrate that the p.I794T variant causes a marked reduction in microglial cell numbers.

Although numerous models (mice, rats, and zebrafish) have been employed to study the loss of one *Csf1r* allele or CSF1R mutations, the molecular mechanisms underlying disease pathogenesis remain incompletely understood. Given that CSF1R-RD is a dominantly inherited leukoencephalopathy, we hypothesized that microglial dysfunction may emerge early and drive progressive white matter pathology. To test this, we performed microglial RNA-seq and identified dysregulated molecular pathways in microglia from both 0-to 3-day-old and 9-month-old *Csf1r*$^{I792T/+}$ mice. Notably, altered expression of cell cycle-related genes in 0- to 3-day-old *Csf1r*$^{I792T/+}$ microglia may underlie reduced microglial numbers. Moreover, activated transcriptional signatures in both 0- to 3-day-old and 9-month-old *Csf1r*$^{I792T/+}$ microglia suggest a pathologically primed microglial state, an observation consistent with prior studies in CSF1R-deficient models.[12] Additionally, while Chitu et al. reported elevated *Csf2* and *Csf3* expression in both 7-week-old *Csf1r*$^{+/-}$ mouse model and CSF1R-RD patients,[27,28] this upregulation was not expected to occur in the 9-month-old *Csf1r*$^{I792T/+}$ mice in this study, as *Csf2*, at least, is predominantly expressed by non-microglial cells, secondary to changes in microglia and may develop later.

Pathway analyses (GSEA and KEGG) further revealed that the *Csf1r* p.I792T variant in 0- to 3-day-old microglia activates genes involved in cytokine-receptor interactions, processes known to contribute to phagosome, neuroinflammation, and microglial dysfunction. This neonatal microglial dataset, while not directly applicable to adult pathology, provides valuable developmental insights and a foundation for future studies investigating microglial biology during early brain maturation. Notably, microglia isolated from 9-month-old *Csf1r*$^{I792T/+}$ mice exhibited an activated and DAM-like phenotype, marked by significantly increased *Stat3*, *Mrc2*, *Atg7*, *Chil3*, and *Cst7* expression, as well as reduced *Tmem119* and *P2ry12* expression, consistent with established neurodegenerative profiles.[29–31] Complementary WGCNA and KEGG analysis of whole-brain RNA-seq data in middle-aged *Csf1r*$^{I792T/+}$ mice revealed enrichment in pathways related to cellular senescence, cell cycle, and virus infection, mirroring findings from the microglial transcriptome. These data support the conclusion that the *Csf1r* p.I792T variant induces a proinflammatory, phagocytic microglial phenotype that is central to CSF1R-RD pathogenesis. Furthermore, transcriptional analysis of DAM from whole-brain tissue revealed their heterogeneous nature. The finding of divergent DAM markers in the brain, as confirmed by qPCR, indicates that the *Csf1r* p.I792T mutation may drive microglia into a mixed activation state rather than a canonical DAM phenotype or a homogeneous one. These results are, in fact, consistent with those from a recent publication, in which several microglia/macrophage clusters were identified in the mutant mouse brains by single-cell RNA-seq.[16] This complex response likely manifests as distinct functional subpopulations across the brain. This interpretation also aligns with a recent study reporting loss of homeostatic microglia in white matter

Figure 6. Preventive microglia replacement halts cognitive and pathological decline in *Csf1r* $^{I792T/+}$ mice
(A) Workflow of the preventive microglia replacement strategy.
(B) Representative flow cytometry dot plots of GFP$^+$CD11b$^+$ microglia in transplanted *Csf1r* $^{I792T/+}$ brains.
(C) Donor-derived GFP$^+$CD11b$^+$ cells comprised up to 82.9% of the total CD11b$^+$ cells (Tp mice).
(D) Confocal images showed widespread colonization of GFP$^+$ (green) donor microglia co-labeled with Iba1 (red) throughout Tp brain. Scale bars: 1 mm and 100 μm.
(E and F) Quantification of the percentage of GFP$^+$Iba1$^+$ microglia relative to Iba1$^+$ cells per high field in the hippocampus (E) and cortex (F) of *Csf1r*$^{I792T/+}$ mouse brain (*n* = 3 per group).
(G) Nest-building behavior was assessed in WT, I792T, and Tp mice as an indicator of cognitive performance.
(H) Nesting scores were significantly improved in Tp mice compared to I792T mice (WT, *n* = 15; I792T, *n* = 14; Tp, *n* = 8).
(I) In the T-maze test, Tp mice exhibited significantly increased spontaneous alternation compared to I792T mice (WT, *n* = 15; I792T, *n* = 14; Tp, *n* = 8).
(J) In the MWM, Tp mice, similar to WT controls, showed shorter escape latency by day 4 of training compared to *Csf1r* $^{I792T/+}$ mice (WT, *n* = 15; I792T, *n* = 14; Tp, *n* = 8).
(K) Representative swimming trajectories during the MWM test for WT, I792T, and Tp mice.
(L–O) Analysis of swimming speed (L), escape latency (M), time spent in the target quadrant (N), and number of platform crossings (O) on the test day confirmed improved spatial memory in Tp mice (WT, *n* = 15; I792T, *n* = 14; Tp, *n* = 8).
(P) Representative western blot analysis of MBP expression in *Csf1r*$^{+/+}$, *Csf1r*$^{I792T/+}$, and Tp mouse brains.
(Q) Quantification of MBP protein levels normalized to β-actin (*n* = 5 per group).
(R–U) Representative images of brain calcifications (alizarin red S staining) and axonal spheroids (phospho-NFH immunostaining) in *Csf1r*$^{+/+}$, *Csf1r*$^{I792T/+}$, and Tp mice (R). Scale bars: 100, 50, and 10 μm. Axonal spheroid counts (S) and densities (T), as well as calcification areas (U), were quantified in WT, I792T, and Tp mice (*n* = 3 per group).
Unpaired two-tailed Student's *t* test (two groups); one-way ANOVA post-Dunnett's multiple comparisons test (more than two groups). Data are presented as mean ± SEM. *$p < 0.05$, **$p < 0.01$, ***$p < 0.001$, ****$p < 0.0001$; ns, not significant. I792T, *Csf1r*$^{I792T/+}$; Tp, *Csf1r*$^{I792T/+}$ mice transplanted with microglia.

CellPress
OPEN ACCESS

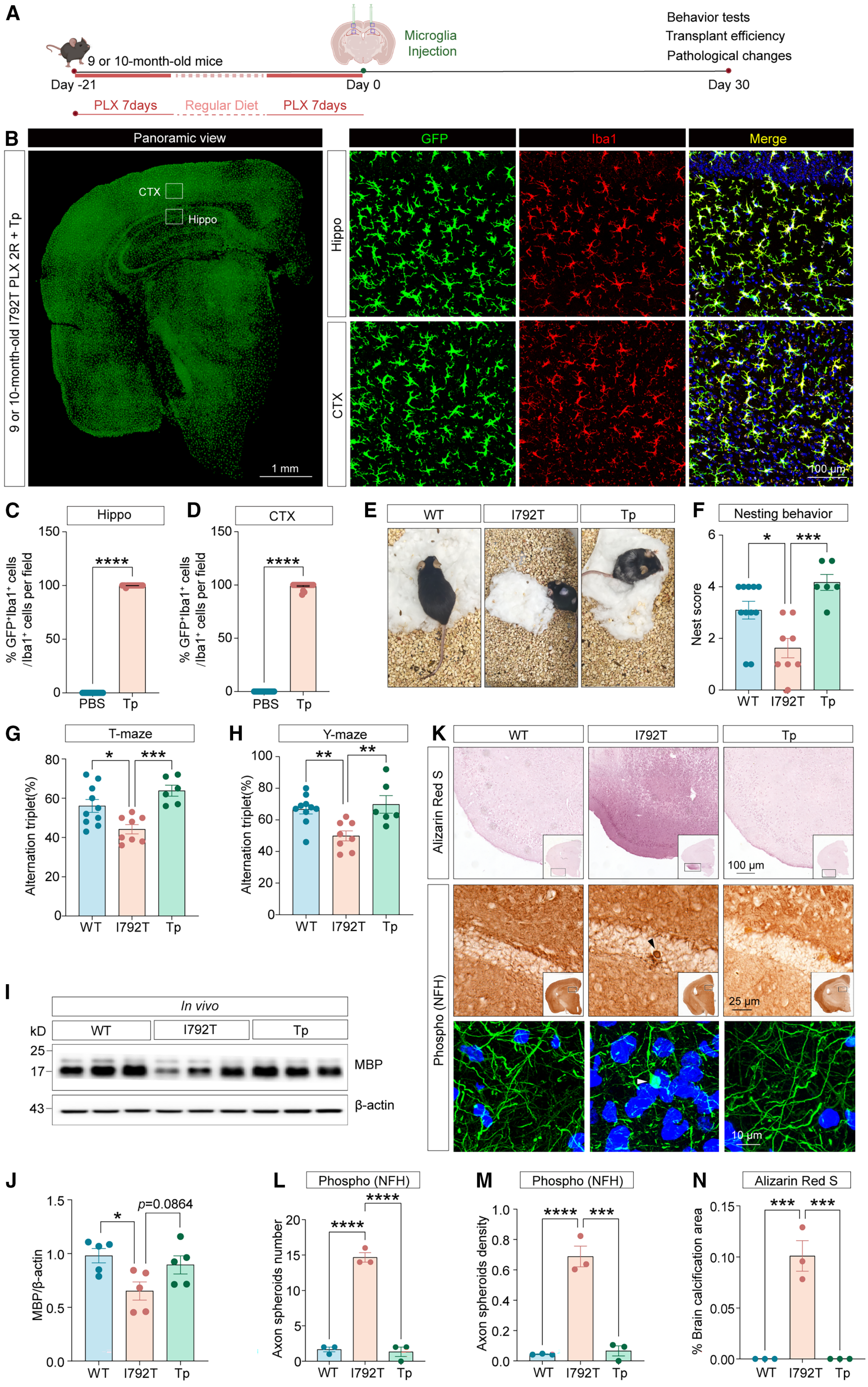

(legend on next page)

from intermediate and late-stage CSF1R-RD postmortem brains.[40] Therefore, future investigations must employ advanced techniques such as single-cell RNA-seq and spatial transcriptomics, which are critical to resolve the precise heterogeneity of mutant microglia, to define the unique molecular states, and to map their distribution across different brain regions. For example, understanding the functional consequences of white versus gray matter microglial heterogeneity for neuronal function represents a compelling frontier for future studies. Altogether, our findings demonstrate that microglial dysfunction alone is sufficient to drive the behavioral deficits and neuropathological features observed in the *Csf1r*$^{I792T/+}$ mouse model of CSF1R-RD.

Due to the rapidly progressive nature of CSF1R-RD, there is an urgent need to develop effective early interventions. While HSCT has been proposed as a disease-modifying therapy,[16] its benefits appear limited, particularly in patients with advanced cognitive decline, yielding variable and often unsatisfactory outcomes.[15] Recently, the transplantation of human induced pluripotent stem cell (iPSC)-derived microglial (iMG) progenitors has emerged as a promising alternative approach.[17] Nevertheless, there remains a significant lack of effective therapeutic options for CSF1R-RD to date. Notably, we found that the endogenous microglial repopulation capacity was markedly impaired in 1-month-old *Csf1r*$^{I792T/+}$ mice, highlighting a proliferation defect intrinsic to the CSF1R p.I794T variant. Given the observed reduction in both microglial numbers and function in the brains of *Csf1r*$^{I792T/+}$ mice, we investigated whether replacing dysfunctional microglia with healthy ones could mitigate disease progression. Our findings demonstrate that early microglial replacement significantly delayed or prevented the onset of cognitive and pathological deficits in young *Csf1r*$^{I792T/+}$ mice. Remarkably, this intervention also reversed behavioral and neuropathological impairments in 9-month-old *Csf1r*$^{I792T/+}$ mice that manifested CSF1R-RD-like symptoms. We noted the discrepancy in the MWM baseline, where the I792T learning deficit appears earlier in the rescue cohort (days 2–3) compared to the initial characterization (day 5). In addition to the microinjection procedure, the experimental and biological variabilities can also contribute to these differences. For example, subtle differences in the testing environment between two experimental batches could influence the sensitivity and performance outcomes of the MWM assay. Moreover, variations in handling, experimenter influence, intertrial intervals, or time of day for testing are all known factors that can alter learning curves. Furthermore, because the I792T mutation might not cause a perfectly uniform phenotype, animals may respond differently to procedural or environmental stressors, leading to apparent timing discrepancies in learning deficits.

Transcriptomic profiling following microglial transplantation revealed, via WGCNA and KEGG analyses, the enrichment of pathways related to axon guidance, neurodegeneration, and other multiple diseases processes, underscoring the therapeutic potential of microglial replacement in attenuating CSF1R-RD neurodegeneration.[28] These results provide a compelling rationale for microglial transplantation as a therapeutic strategy not only for early-stage intervention but also in symptomatic individuals.

To develop a more clinically feasible microglial transplantation approach, we also attempted direct microglial transplantation and a single-round microglia depletion followed by transplantation in 9-month-old *Csf1r*$^{I792T/+}$ mice. We initially tried to isolate microglia from postnatal day 1 mice using FACS for transplantation. However, this approach yielded limited cell numbers with poor viability as demonstrated by CCK-8 assays (Figure S8A). Furthermore, the sorted microglia failed to migrate from the injection site, indicating their unsuitability for transplantation studies (Figures S8B and S8C). We next attempted two transplantation approaches in 9-month-old *Csf1r*$^{I792T/+}$ mice: (1) direct transplantation without prior microglial depletion (Figures S9A–S9C) and (2) transplantation following single microglial depletion (Figures S9D–S9F). Both strategies proved unsuccessful for microglial replacement in aged *Csf1r*$^{I792T/+}$ mice. While a recent study achieved successful transplantation in 16-month-old mice with a 14-day PLX5622 regimen,[16] in this study, a single 7-day administration of PLX3397 failed to achieve complete microglial clearance in 9-month-old *Csf1r*$^{I792T/+}$ mice (data not shown) and was, therefore, inadequate for supporting subsequent efficient engraftment. Two non-mutually exclusive hypotheses may explain the low engraftment rate (1.12%) in 9-month-old symptomatic *Csf1r*$^{I792T/+}$ mice after a 7-day PLX treatment. First, aged microglia themselves may be more resistant to depletion, creating a crowded niche that impedes donor cell entry. Second, the aged brain may constitute a dysfunctional

Figure 7. Therapeutic microglial replacement reverses cognitive and pathological deficits in *Csf1r* $^{I792T/+}$ mice
(A) Schematic overview of the therapeutic microglial transplantation strategy.
(B) Representative confocal images of coronal brain sections showing widespread engraftment of GFP$^+$ (green) Iba1$^+$ (red) microglia in the brains of transplanted *Csf1r*$^{I792T/+}$ mice. Scale bars: 1 mm and 100 μm.
(C and D) Quantification of GFP$^+$Iba1$^+$ microglia as a percentage of total Iba1$^+$ cells in the hippocampus (C) and cortex (D) of *Csf1r*$^{I792T/+}$ mice. n = 4∼5 mice.
(E) Representative images of nests built by *Csf1r*$^{+/+}$, *Csf1r* $^{I792T/+}$, or transplanted *Csf1r* $^{I792T/+}$ mice. Nest quality was used as a proxy for cognitive performance.
(F) Nesting scores quantified across groups: *Csf1r*$^{+/+}$ (n = 10), *Csf1r* $^{I792T/+}$ mice (n = 8), and transplanted *Csf1r* $^{I792T/+}$ mice (n = 6).
(G and H) T/Y-maze tests evaluating spontaneous alternation behavior. The percentage of alternation triplets was calculated for *Csf1r*$^{+/+}$ (n = 10) and *Csf1r* $^{I792T/+}$ mice (n = 8) and I792T mice transplanted with GFP$^+$Iba1$^+$ microglia (n = 6).
(I) Representative western blot analysis of MBP expression in *Csf1r*$^{+/+}$, *Csf1r*$^{I792T/+}$, and transplanted *Csf1r*$^{I792T/+}$ mouse brains.
(J) Quantification of MBP protein levels normalized to β-actin (n = 5 mice).
(K) Representative images of brain calcifications (alizarin red S staining) and axonal spheroids (phospho-NFH immunostaining) in WT, I792T, and Tp mice (n = 3 per group). Scale bars: 100, 25, and 10 μm.
(L–N) Axonal spheroid counts (L) and densities (M), as well as calcification areas (N), were quantified in WT, I792T, or Tp mice.
Unpaired two-tailed Student's t test (two groups); one-way ANOVA post-Dunnett's multiple comparisons test (more than two groups). Data are presented as mean ± SEM. *$p < 0.05$, **$p < 0.01$, ***$p < 0.001$, ****$p < 0.000$. RD, regular diet. WT, wild-type; I792T, *Csf1r*$^{I792T/+}$; Tp, *Csf1r*$^{I792T/+}$ mice transplanted with microglia.

CellPress
OPEN ACCESS

niche, potentially deficient in critical nutrients or trophic factors, thereby impairing the adaptation and survival of transplanted microglia. The failure of these protocols to achieve therapeutic engraftment may highlight the necessity of creating an optimal niche for donor cell engraftment in aged mice. Our data indicate that for more robust efficacy across both preventive and therapeutic settings, two alternating rounds of depletion are required. Therefore, two-phase microglia depletion may ensure complete niche clearance, critical for donor cell engraftment, and may overcome the competitive advantage of resident dysfunctional microglia, a challenge not addressed by single-dose regimens. Thus, the developed depletion and replacement strategy termed "DCMDT" in this study may overcome these limitations and enable efficient microglial turnover in both the young and old *Csf1r*$^{I792T/+}$ mouse model.

Although a recent study demonstrated that transplantation of iMG progenitors could prevent pathological progression in a CSF1R-FIRE-deficient mouse model,[17] and other approaches have also shown promise,[45,46] further clinical trials are essential to evaluate the efficacy and long-term safety of these therapies. Additionally, the recent generation of iMGs from a CSF1R-RD patient carrying a pathogenic variant, achieved by optimizing an existing iPSC-derived microglia protocol, offers tools to explore human-specific disease mechanisms and therapeutic responses.[47] From a translational perspective, future studies should explore alternative delivery methods such as intravenous or intrathecal injection for microglial replacement therapy.

Collectively, our findings identify microglial dysfunction as a central pathogenic mechanism in CSF1R-RD and provide strong evidence that restoring functional microglial populations can ameliorate disease. This study elucidates the molecular underpinnings of CSF1R variant-mediated microglial impairment and establishes microglial replacement as a viable therapeutic strategy. Moreover, the clinical approval status of PLX3397 renders it more suitable for translational applications, thereby potentially accelerating the clinical adoption of DCMDT for treating CSF1R-RD patients. Ultimately, our work lays the groundwork for future efforts aimed at mitigating microglia-associated neurodegeneration in CSF1R-RD.

Limitations of the study

Several limitations warrant consideration in this study. First, CSF1R-RD represents a rare neurodegenerative leukoencephalopathy. Despite efforts to expand the sample number in this study, the limited cohort may impede a comprehensive assessment of the clinical heterogeneity associated with the CSF1R p.I794T mutation. Second, although the *Csf1r*$^{I792T/+}$ mouse model generated via homologous recombination recapitulates core cognitive pathological features observed in human CSF1R-RD, inherent interspecies differences in neuroanatomical structure, immune function, and disease progression may constrain direct translatability between observed phenotypes and clinical pathology. Third, although the DCMDT strategy demonstrates neuroprotective effects in *Csf1r*$^{I792T/+}$ mouse model, its clinical translational potential requires further validation. Before clinical application, key challenges, including immune compatibility, engraftment efficiency, and technical complexity, must be addressed. Finally, transcriptomic analysis suggests a DAM-like phenotype in *Csf1r*$^{I792T/+}$ microglia; however, how *Csf1r*$^{I792T/+}$ microglia contribute to leukoencephalopathy remains to be elucidated and the specific molecular mechanisms need to be fully defined.

RESOURCE AVAILABILITY

Lead contact

Further information and requests for resources and reagents should be directed to and will be fulfilled by the lead contact, Honghua Zheng (honghua@xmu.edu.cn).

Materials availability

This study did not generate new unique reagents.

Data and code availability

- Raw RNA-seq data used in the study are available at https://db.cngb.org/ using accession code CNP0007429. All data reported in this paper will be shared by the lead contact upon request.
- This paper does not report original code.
- Any additional information required to reanalyze the data reported in this paper is available from the lead contact upon request.

ACKNOWLEDGMENTS

This work was supported by grants from the National Natural Science Foundation of China (82271219, 91949129, 32471045, and 82522029) and the program of Lin Gang Laboratory LGL-3142-ADB120102 (to H.Z.). This work was also supported by a grant from the Innovation and Technology Commission (ITCPD/17-9) (to G.B.). The authors thank Zicheng Huang from the Center for Molecular Imaging and Translational Medicine of Xiamen University for her professional help in MRI analysis. The authors also thank the colleagues from the Biomedical Shared Research Platform of Xiamen University for their technical help: Haiping Zheng for flow cytometry, Baoying Xie for behavioral tests, Xiang You and Jingru Huang for imaging, and Luming Yao for electron microscopy.

AUTHOR CONTRIBUTIONS

H.Z., L.Z., Y.-W. Z., and Z.Z. contributed to the conception and design of the study; C.W. provided the pedigree and imaging data of the patients in this study; X.L. and H.F. collected and analyzed the clinical information from all patients carrying the CSF1R I794T mutation, both in this study and those reported in the literature. The behavior test, western blot, immunofluorescence staining, flow cytometry, MRI scanning, and electron microscopy experiments were conducted by X.L. and B.H.; alizarin red S staining and NFH immunohistochemical staining were carried out by X.L.; RNA sequencing data were analyzed by Z.W.; electrophysiological experiments were performed by B.H.; additionally, X.H., L.Z., and Y.L. contributed unpublished reagents/analytic tools; G.B. contributed to conceptual and experimental advice on the project; H.Z., X.L., B.H., C.W., Y.L., X.Z., and L.Z. prepared the figures. X.L. wrote the STAR Methods section. C.W. documented the clinical and imaging characteristics of the patients. H.Z. wrote the main text. All authors commented on the manuscript and approved the final manuscript.

DECLARATION OF INTERESTS

The authors declare no competing interests.

STAR★METHODS

Detailed methods are provided in the online version of this paper and include the following:

- KEY RESOURCES TABLE
- EXPERIMENTAL MODEL AND STUDY PARTICIPANT DETAILS
  - Patients with CSF1R-RD

   - Mice
- METHOD DETAILS
   - Flow cytometry sorting of microglia
   - Primary microglia isolation
   - Microglia depletion and repopulation
   - Microglia transplantation
   - RNA-sequencing
   - Electrophysiological recording
   - T/Y-maze test
   - Open-field test
   - High plus maze test
   - Morris water maze test (MWM)
   - Nesting behavior test
   - *In vivo* MRI scanning
   - Electron microscopy
   - Alizarin red S staining
   - Immunofluorescent staining
   - NFH immunohistochemical staining
   - Western blot
   - RNA extraction and quantitative polymerase chain reaction (qPCR)
   - Statistical analysis

SUPPLEMENTAL INFORMATION

Supplemental information can be found online at https://doi.org/10.1016/j.xcrm.2026.102641.

Received: February 26, 2025
Revised: June 3, 2025
Accepted: January 29, 2026
Published: February 27, 2026

REFERENCES

1. Konno, T., Kasanuki, K., Ikeuchi, T., Dickson, D.W., and Wszolek, Z.K. (2018). CSF1R-related leukoencephalopathy: A major player in primary microgliopathies. Neurology *91*, 1092–1104. https://doi.org/10.1212/WNL.0000000000006642.
2. Chitu, V., and Stanley, E.R. (2017). Regulation of Embryonic and Postnatal Development by the CSF-1 Receptor. Curr. Top. Dev. Biol. *123*, 229–275. https://doi.org/10.1016/bs.ctdb.2016.10.004.
3. Nicholson, A.M., Baker, M.C., Finch, N.A., Rutherford, N.J., Wider, C., Graff-Radford, N.R., Nelson, P.T., Clark, H.B., Wszolek, Z.K., Dickson, D.W., et al. (2013). CSF1R mutations link POLD and HDLS as a single disease entity. Neurology *80*, 1033–1040. https://doi.org/10.1212/WNL.0b013e31828726a7.
4. Adams, S.J., Kirk, A., and Auer, R.N. (2018). Adult-onset leukoencephalopathy with axonal spheroids and pigmented glia (ALSP): Integrating the literature on hereditary diffuse leukoencephalopathy with spheroids (HDLS) and pigmentary orthochromatic leukodystrophy (POLD). J. Clin. Neurosci. *48*, 42–49. https://doi.org/10.1016/j.jocn.2017.10.060.
5. Dulski, J., Muthusamy, K., Lund, T.C., and Wszolek, Z.K. (2024). CSF1R-related disorder: State of the art, challenges, and proposition of a new terminology. Parkinsonism Relat. Disord. *121*, 105894. https://doi.org/10.1016/j.parkreldis.2023.105894.
6. Wade, C., Runeckles, K., Chataway, J., Houlden, H., and Lynch, D.S. (2024). CSF1R-Related Disorder: Prevalence of CSF1R Variants and Their Clinical Significance in the UK Population. Neurol. Genet. *10*, e200179. https://doi.org/10.1212/NXG.0000000000200179.
7. Wu, J., Cheng, X., Ji, D., Niu, H., Yao, S., Lv, X., Wang, J., Li, Z., Zheng, H., Cao, Y., et al. (2024). The Phenotypic and Genotypic Spectrum of CSF1R-Related Disorder in China. Mov. Disord. *39*, 798–813. https://doi.org/10.1002/mds.29764.
8. Chitu, V., Gokhan, Ş., Nandi, S., Mehler, M.F., and Stanley, E.R. (2016). Emerging Roles for CSF-1 Receptor and its Ligands in the Nervous System. Trends Neurosci. *39*, 378–393. https://doi.org/10.1016/j.tins.2016.03.005.
9. Chitu, V., Gokhan, S., Gulinello, M., Branch, C.A., Patil, M., Basu, R., Stoddart, C., Mehler, M.F., and Stanley, E.R. (2015). Phenotypic characterization of a Csf1r haploinsufficient mouse model of adult-onset leukodystrophy with axonal spheroids and pigmented glia (ALSP). Neurobiol. Dis. *74*, 219–228. https://doi.org/10.1016/j.nbd.2014.12.001.
10. Biundo, F., Chitu, V., Shlager, G.G.L., Park, E.S., Gulinello, M.E., Saha, K., Ketchum, H.C., Fernandes, C., Gökhan, Ş., Mehler, M.F., and Stanley, E.R. (2021). Microglial reduction of colony stimulating factor-1 receptor expression is sufficient to confer adult onset leukodystrophy. Glia *69*, 779–791. https://doi.org/10.1002/glia.23929.
11. Arreola, M.A., Soni, N., Crapser, J.D., Hohsfield, L.A., Elmore, M.R.P., Matheos, D.P., Wood, M.A., Swarup, V., Mortazavi, A., and Green, K.N. (2021). Microglial dyshomeostasis drives perineuronal net and synaptic loss in a CSF1R(+/-) mouse model of ALSP, which can be rescued via CSF1R inhibitors. Sci. Adv. *7*, eabg1601. https://doi.org/10.1126/sciadv.abg1601.
12. Li, X., Hu, B., Guan, X., Wang, Z., Zhou, Y., Sun, H., Zhang, X., Li, Y., Huang, X., Zhao, Y., et al. (2023). Minocycline protects against microgliopathy in a Csf1r haplo-insufficient mouse model of adult-onset leukoencephalopathy with axonal spheroids and pigmented glia (ALSP). J. Neuroinflammation *20*, 134. https://doi.org/10.1186/s12974-023-02774-1.
13. Chitu, V., Biundo, F., Oppong-Asare, J., Gökhan, Ş., Aguilan, J.T., Dulski, J., Wszolek, Z.K., Sidoli, S., and Stanley, E.R. (2023). Prophylactic effect of chronic immunosuppression in a mouse model of CSF-1 receptor-related leukoencephalopathy. Glia *71*, 2664–2678. https://doi.org/10.1002/glia.24446.
14. Dulski, J., Stanley, E.R., Chitu, V., and Wszolek, Z.K. (2023). Potential use of glucocorticosteroids in CSF1R mutation carriers - current evidence and future directions. Neurol. Neurochir. Pol. *57*, 444–449. https://doi.org/10.5603/pjnns.97373.
15. Dulski, J., Heckman, M.G., White, L.J., Żur-Wyrozumska, K., Lund, T.C., and Wszolek, Z.K. (2022). Hematopoietic Stem Cell Transplantation in CSF1R-Related Leukoencephalopathy: Retrospective Study on Predictors of Outcomes. Pharmaceutics *14*, 2778. https://doi.org/10.3390/pharmaceutics14122778.
16. Wu, J., Wang, Y., Li, X., Ouyang, P., Cai, Y., He, Y., Zhang, M., Luan, X., Jin, Y., Wang, J., et al. (2025). Microglia replacement halts the progression of microgliopathy in mice and humans. Science *389*, eadr1015. https://doi.org/10.1126/science.adr1015.
17. Chadarevian, J.P., Hasselmann, J., Lahian, A., Capocchi, J.K., Escobar, A., Lim, T.E., Le, L., Tu, C., Nguyen, J., Kiani Shabestari, S., et al. (2024). Therapeutic potential of human microglia transplantation in a chimeric model of CSF1R-related leukoencephalopathy. Neuron *112*, 2686–2707.e8. https://doi.org/10.1016/j.neuron.2024.05.023.
18. Munro, D.A.D., Bestard-Cuche, N., McQuaid, C., Chagnot, A., Shabestari, S.K., Chadarevian, J.P., Maheshwari, U., Szymkowiak, S., Morris, K., Mohammad, M., et al. (2024). Microglia protect against age-associated brain pathologies. Neuron *112*, 2732–2748.e8. https://doi.org/10.1016/j.neuron.2024.05.018.
19. Rademakers, R., Baker, M., Nicholson, A.M., Rutherford, N.J., Finch, N., Soto-Ortolaza, A., Lash, J., Wider, C., Wojtas, A., DeJesus-Hernandez, M., et al. (2011). Mutations in the colony stimulating factor 1 receptor (CSF1R) gene cause hereditary diffuse leukoencephalopathy with spheroids. Nat. Genet. *44*, 200–205. https://doi.org/10.1038/ng.1027.
20. Dulski, J., Baker, M., Banks, S.A., Bayat, M., Bruffaerts, R., Ortiz Cruz, G., Disserol, C.C., Fisher, K.S., Jose, J.N., Kalman, B., et al. (2024). Global Presence and Penetrance of CSF1R-Related Disorder. Neurol. Genet. *10*, e200187. https://doi.org/10.1212/NXG.0000000000200187.
21. Chitu, V., Gokhan, S., and Stanley, E.R. (2021). Modeling CSF-1 receptor deficiency diseases - how close are we? FEBS J. *289*, 5049–5073. https://doi.org/10.1111/febs.16085.

22. Lee, J.Y., Taghian, K., and Petratos, S. (2014). Axonal degeneration in multiple sclerosis: can we predict and prevent permanent disability? Acta Neuropathol. Commun. *2*, 97. https://doi.org/10.1186/s40478-014-0097-7.

23. Stables, J., Green, E.K., Sehgal, A., Patkar, O.L., Keshvari, S., Taylor, I., Ashcroft, M.E., Grabert, K., Wollscheid-Lengeling, E., Szymkowiak, S., et al. (2022). A kinase-dead Csf1r mutation associated with adult-onset leukoencephalopathy has a dominant inhibitory impact on CSF1R signalling. Development *149*, dev200237. https://doi.org/10.1242/dev.200237.

24. Friedman, B.A., Srinivasan, K., Ayalon, G., Meilandt, W.J., Lin, H., Huntley, M.A., Cao, Y., Lee, S.H., Haddick, P.C.G., Ngu, H., et al. (2018). Diverse Brain Myeloid Expression Profiles Reveal Distinct Microglial Activation States and Aspects of Alzheimer's Disease Not Evident in Mouse Models. Cell Rep. *22*, 832–847. https://doi.org/10.1016/j.celrep.2017.12.066.

25. Saeki, K., Pan, R., Lee, E., Kurotaki, D., and Ozato, K. (2024). IRF8 defines the epigenetic landscape in postnatal microglia, thereby directing their transcriptome programs. Nat. Immunol. *25*, 1928–1942. https://doi.org/10.1038/s41590-024-01962-2.

26. Prater, K.E., Green, K.J., Mamde, S., Sun, W., Cochoit, A., Smith, C.L., Chiou, K.L., Heath, L., Rose, S.E., Wiley, J., et al. (2023). Human microglia show unique transcriptional changes in Alzheimer's disease. Nat. Aging *3*, 894–907. https://doi.org/10.1038/s43587-023-00424-y.

27. Biundo, F., Chitu, V., Tindi, J., Burghardt, N.S., Shlager, G.G.L., Ketchum, H.C., DeTure, M.A., Dickson, D.W., Wszolek, Z.K., Khodakhah, K., and Stanley, E.R. (2023). Elevated granulocyte colony stimulating factor (CSF) causes cerebellar deficits and anxiety in a model of CSF-1 receptor related leukodystrophy. Glia *71*, 775–794. https://doi.org/10.1002/glia.24310.

28. Chitu, V., Biundo, F., Shlager, G.G.L., Park, E.S., Wang, P., Gulinello, M.E., Gokhan, Ş., Ketchum, H.C., Saha, K., DeTure, M.A., et al. (2020). Microglial Homeostasis Requires Balanced CSF-1/CSF-2 Receptor Signaling. Cell Rep. *30*, 3004–3019.e5. https://doi.org/10.1016/j.celrep.2020.02.028.

29. Tran, K.M., Kwang, N.E., Butler, C.A., Gomez-Arboledas, A., Kawauchi, S., Mar, C., Chao, D., Barahona, R.A., Da Cunha, C., Tsourmas, K.I., et al. (2025). APOE Christchurch enhances a disease-associated microglial response to plaque but suppresses response to tau pathology. Mol. Neurodegener. *20*, 9. https://doi.org/10.1186/s13024-024-00793-x.

30. Frolov, A., Huang, H., Schütz, D., Köhne, M., Blank-Stein, N., Osei-Sarpong, C., Büttner, M., Elmzzahi, T., Khundadze, M., Zahid, M., et al. (2025). Microglia and CD8+ T cell activation precede neuronal loss in a murine model of spastic paraplegia 15. J. Exp. Med. *222*, e20232357. https://doi.org/10.1084/jem.20232357.

31. Xu, J., Song, W., Xu, Z., Danziger, M.M., Karavani, E., Zang, C., Chen, X., Li, Y., Paz, I.M.R., Gohel, D., et al. (2025). Single-microglia transcriptomic transition network-based prediction and real-world patient data validation identifies ketorolac as a repurposable drug for Alzheimer's disease. Alzheimer's Dement. *21*, e14373. https://doi.org/10.1002/alz.14373.

32. Chen, D., Wang, C., Chen, X., Li, J., Chen, S., Li, Y., Ma, F., Li, T., Zou, M., Li, X., et al. (2025). Brain-wide microglia replacement using a nonconditioning strategy ameliorates pathology in mouse models of neurological disorders. Sci. Transl. Med. *17*, eads6111. https://doi.org/10.1126/scitranslmed.ads6111.

33. Najafi, A.R., Crapser, J., Jiang, S., Ng, W., Mortazavi, A., West, B.L., and Green, K.N. (2018). A limited capacity for microglial repopulation in the adult brain. Glia *66*, 2385–2396. https://doi.org/10.1002/glia.23477.

34. Schmitz, A.S., Raju, J., Köhler, W., Klebe, S., Cheheb, K., Reschke, F., Biskup, S., Haack, T.B., Roeben, B., Kellner, M., et al. (2024). Novel variants in CSF1R associated with adult-onset leukoencephalopathy with axonal spheroids and pigmented glia (ALSP). J. Neurol. *271*, 6025–6037. https://doi.org/10.1007/s00415-024-12557-0.

35. Mikuni, M., Horiuchi, K., Ishikura, A., Kimura, S., Masutani, S., Watanabe, S., Mikami, A., Ishikawa, S., Narita, H., Kusumi, I., and Sasaki, H. (2024). Suspected Postpartum Depression Revealed to be CSF1R-Related Leukoencephalopathy: A Case Report. Case Rep. Neurol. *16*, 281–287. https://doi.org/10.1159/000541551.

36. Beerepoot, S., Wolf, N.I., van der Knaap, M.S., Nierkens, S., and Plantinga, M. (2024). Heterozygous missense CSF1R variants hamper in vitro CD34+-derived dendritic cell generation but not in vivo dendritic cell development. Mol. Immunol. *174*, 41–46. https://doi.org/10.1016/j.molimm.2024.08.002.

37. Pan, J., Fores-Martos, J., Delpirou Nouh, C., Jensen, T.D., Vallejo, K., Cayrol, R., Ahmadian, S., Ashley, E.A., Greicius, M.D., and Cobos, I. (2024). Deciphering glial contributions to CSF1R-related disorder via single-nuclear transcriptomic profiling: a case study. Acta Neuropathol. Commun. *12*, 139. https://doi.org/10.1186/s40478-024-01853-5.

38. Han, Y., Han, J., Li, Z., Chen, S., Liu, J., Zhou, R., Zhao, S., Li, D., Liu, Z., Zhao, Y., et al. (2024). Identification and characterization of a novel intronic splicing mutation in CSF1R-related leukoencephalopathy. CNS Neurosci. Ther. *30*, e14815. https://doi.org/10.1111/cns.14815.

39. Dulski, J., Sundal, C., and Wszolek, Z.K. (1993). CSF1R-Related Disorder. In GeneReviews(®), M.P. Adam, J. Feldman, G.M. Mirzaa, R.A. Pagon, S.E. Wallace, and A. Amemiya, eds. (Copyright © 1993-2025, University of Washington, Seattle. GeneReviews is a registered trademark of the University of Washington, Seattle. All rights reserved).

40. Berdowski, W.M., van der Linde, H.C., Breur, M., Oosterhof, N., Beerepoot, S., Sanderson, L., Wijnands, L.I., de Jong, P., Tsai-Meu-Chong, E., de Valk, W., et al. (2022). Dominant-acting CSF1R variants cause microglial depletion and altered astrocytic phenotype in zebrafish and adult-onset leukodystrophy. Acta Neuropathol. *144*, 211–239. https://doi.org/10.1007/s00401-022-02440-5.

41. Patkar, O.L., Caruso, M., Teakle, N., Keshvari, S., Bush, S.J., Pridans, C., Belmer, A., Summers, K.M., Irvine, K.M., and Hume, D.A. (2021). Analysis of homozygous and heterozygous Csf1r knockout in the rat as a model for understanding microglial function in brain development and the impacts of human CSF1R mutations. Neurobiol. Dis. *151*, 105268. https://doi.org/10.1016/j.nbd.2021.105268.

42. Papapetropoulos, S., Pontius, A., Finger, E., Karrenbauer, V., Lynch, D.S., Brennan, M., Zappia, S., Koehler, W., Schoels, L., Hayer, S.N., et al. (2021). Adult-Onset Leukoencephalopathy With Axonal Spheroids and Pigmented Glia: Review of Clinical Manifestations as Foundations for Therapeutic Development. Front. Neurol. *12*, 788168. https://doi.org/10.3389/fneur.2021.788168.

43. Kempthorne, L., Yoon, H., Madore, C., Smith, S., Wszolek, Z.K., Rademakers, R., Kim, J., Butovsky, O., and Dickson, D.W. (2020). Loss of homeostatic microglial phenotype in CSF1R-related Leukoencephalopathy. Acta Neuropathol. Commun. *8*, 72. https://doi.org/10.1186/s40478-020-00947-0.

44. Du, S., Zhou, Y., Li, D., Lier, J., Cella, M., Tada, M., Hamasaki, H., Wu, J., Cai, Z., Orthmann-Murphy, J.L., et al. (2025). Mutations in the human CSF1R gene impact microglia's maintenance of brain white matter integrity. Nat. Immunol. *26*, 1198–1211. https://doi.org/10.1038/s41590-025-02195-7.

45. Hu, B., Duan, S., Wang, Z., Li, X., Zhou, Y., Zhang, X., Zhang, Y.W., Xu, H., and Zheng, H. (2021). Insights Into the Role of CSF1R in the Central Nervous System and Neurological Disorders. Front. Aging Neurosci. *13*, 789834. https://doi.org/10.3389/fnagi.2021.789834.

46. Han, J., Sarlus, H., Wszolek, Z.K., Karrenbauer, V.D., and Harris, R.A. (2020). Microglial replacement therapy: a potential therapeutic strategy for incurable CSF1R-related leukoencephalopathy. Acta Neuropathol. Commun. *8*, 217. https://doi.org/10.1186/s40478-020-01093-3.

47. Dorion, M.F., Casas, D., Shlaifer, I., Yaqubi, M., Fleming, P., Karpilovsky, N., Chen, C.X.Q., Nicouleau, M., Piscopo, V.E.C., MacDougall, E.J., et al. (2024). An adapted protocol to derive microglia from stem cells and its application in the study of CSF1R-related disorders. Mol. Neurodegener. *19*, 31. https://doi.org/10.1186/s13024-024-00723-x.

48. Subramanian, A., Tamayo, P., Mootha, V.K., Mukherjee, S., Ebert, B.L., Gillette, M.A., Paulovich, A., Pomeroy, S.L., Golub, T.R., Lander, E.S.,

and Mesirov, J.P. (2005). Gene set enrichment analysis: a knowledge-based approach for interpreting genome-wide expression profiles. Proc. Natl. Acad. Sci. USA *102*, 15545–15550. https://doi.org/10.1073/pnas.0506580102.

49. Kanehisa, M., Furumichi, M., Sato, Y., Matsuura, Y., and Ishiguro-Watanabe, M. (2025). KEGG: biological systems database as a model of the real world. Nucleic Acids Res. *53*, D672–D677. https://doi.org/10.1093/nar/gkae909.

50. Topilko, T., Diaz, S.L., Pacheco, C.M., Verny, F., Rousseau, C.V., Kirst, C., Deleuze, C., Gaspar, P., and Renier, N. (2022). Edinger-Westphal peptidergic neurons enable maternal preparatory nesting. Neuron *110*, 1385–1399.e8. https://doi.org/10.1016/j.neuron.2022.01.012.

51. Uekawa, K., Anfray, A., Ahn, S.J., Casey, N., Seo, J., Zhou, P., Iadecola, C., and Park, L. (2024). tPA supplementation preserves neurovascular and cognitive function in Tg2576 mice. Alzheimer's Dement. *20*, 4572–4582. https://doi.org/10.1002/alz.13878.

STAR★METHODS

KEY RESOURCES TABLE

REAGENT or RESOURCE	SOURCE	IDENTIFIER
Antibodies		
Anti-GAPDH antibody	Abcam	Cat. #ab8245; RRID: AB_2630358 RRID: AB_2630358
Anti-CSF1R	R&D Systems	Cat. #AF3818; RRID: AB_884158
Iba1/AIF-1 (E4O4W) XP® Rabbit mAb (17198)	Cell Signaling Technology	Cat. #17198S; RRID: AB_2820254
Donkey anti-Rabbit IgG (H + L) Highly Cross-Adsorbed Secondary Antibody, Alexa Fluor^{TM} 488 Anti-Rabbit Recombinant Secondary Antibody (H + L)	Invitrogen	Cat. #A21206; RRID: AB_2535792
Anti-Neurofilament H Phospho (phos-NFH) Antibody	Biolegend	Cat. #840801; RRID: AB_2565456
Donkey anti-Rabbit IgG (H + L) Highly Cross-Adsorbed Secondary Antibody, Alexa Fluor^{TM} 568 Cross-Adsorbed Secondary Antibody, Alexa Fluor^{TM} 568	Invitrogen	Cat. #A10042; RRID: AB_2534017
MBP antibody (F-6)	Santa_Cruz	Cat. #sc-271524; RRID: AB_10655672
CD11b Monoclonal Antibody (M1/70), APC	Invitrogen	Cat. #17-0112-83; RRID: AB_469344 AB_
CD45 Monoclonal Antibody (30-F11), FITC	Invitrogen	Cat. #11-0451-82; RRID: AB_465050
Iba-1 antibody	Wako	Cat. # 019–19741; RRID: AB_839504
IBA1 antibody guinea pig	SYSY	Cat. # 234 004; RRID: AB_2493179
CD45 Monoclonal Antibody (30-F11), PE-Cyanine7 eBioscience^{TM} (30-F11), PE-Cyanine7, eBioscience^{TM}	Invitrogen	Cat. # A18710; RRID: AB_2535494, RRID: AB_2535494 AB_
Anti-β-actin antibody	Abcam Cat. #ab8226,	Cat. #ab8226; RRID: AB_306371 AB_306371
Goat anti-Guinea Pig IgG (H + L) Highly Cross-Adsorbed Secondary Antibody, Alexa Fluor^{TM} 647	Invitrogen	Cat. #A-21450; RRID: AB_2535867
Chemicals, peptides, and recombinant proteins		
GM-CSF	novoprotein	Cat. #CK02
PLX3397	MedChemExpress	Cat. #HY-16749
Poly-L-lysine	Sigma	Cat. #P1274
Bovine Serum Albumin V	Solarbio	Cat. #A8020
Isoflurane	RWD	Cat. #R510-22-10
DAPI	Yeasen	Cat. #40728ES03
RIPA buffer	LABLEAD	Cat. #R1091
Critical commercial assays		
RNA isolater Total RNA Extraction Reagent	Vazyme	Cat. #R401-01
HIScript III All-in-one RT SuperMix Perfect for qPCR	Vazyme	Cat. #R333-01
HamQ Universal SYBR qPCR Master Mix	Vazyme	Cat. #q711-03
Protein Quantification Kit (BCA Assay)	Abbkine	Cat. #KTD3010-CN
SuperKine ECL detection kit	Abbkine	Cat. #BMU102-CN
Biotin-labeled antibodies, anti-streptomycin peroxidase	MXB	Cat. #Kit-9270
DAB Kit	MXB	Cat. #DAB0031

(Continued on next page)

Continued

REAGENT or RESOURCE	SOURCE	IDENTIFIER
Triton^{TM} X-100	Sigma	Cat. #T8787
Alizarin Red S	Solarbio	Cat. #G1450
100×proteinase inhibitor cocktail	APE×BIO	Cat. #k1007
100×phosphatase inhibitor cocktail	APE×BIO	Cat. #k1015
DMEM	Pricella	Cat. #PM150210)
1% penicillin/streptomycin	BasalMedia	Cat. #S110JV
Fetal bovine serum	ExCell Bio	Cat. #FSD500
**Experimental models: Cell lines**		
Primary microglia<br>This study<br>N/A	This study	N/A
**Experimental models: Organisms/strains**		
Csf1r$^{I792T/+}$ knock-in mice (C57BL/6J)	GemPharmatech	Cat. #GPS00001710
Cx3cr1$^{GFP/+}$ mice	Jackson Laboratory	Cat. #008451
**Deposited data**		
Raw RNA-seq data	This paper; CNP0007429	https://db.cngb.org/
Raw data from Figures 1, 2, 3, 4, 5, 6, and 7	This paper; Mendeley Data	https://doi.org/10.17632/j7n9jhnv5j.1
Raw data from Figures S1–S9	This paper; Mendeley Data	https://doi.org/10.17632/j7n9jhnv5j.1
**Software and algorithms**		
Prism 10.0.2	GraphPad	https://www.graphpad.com
Fiji	National Institute of Health USA	https://imagej.net/software/fiji
Excel	Microsoft Office	N/A
FV10-ASW 4.2 Viewer	Olympus	N/A
ImageScope x64	Leica	N/A
ZEISS ZEN 3.11	ZEISS	N/A
FlowJo_V10.exe	BD Life Sciences	N/A
Imaris 9.2.1	Bitplane	N/A
BioRender	BioRender	https://www.biorender.com/

EXPERIMENTAL MODEL AND STUDY PARTICIPANT DETAILS

Patients with CSF1R-RD

Clinical data from 3 genetically diagnosed probands and their family members who met published criteria for definite CSF1R-RD were obtained from the Department of Neurology, Beijing Tiantan Hospital, Capital Medical University (Medical ethics permission number KY2020-105-02). This observational study adhered to the relevant Strobe guidelines/requirements for cohort studies. Written informed consent was obtained from all participants.

Mice

Csf1r$^{I792T/+}$ knock-in mice (C57BL/6J) were customized by GemPharmatech Co. Ltd using homologous recombination-based gene editing (project number: GPS00001710). Mice were bred by heterozygous self-crossing. *Cx3cr1*$^{GFP/+}$ (stock 008451) mice were purchased from the Jackson Laboratory. All mice were housed in Xiamen University Laboratory Animal Center, with 12 h of alternating light/darkness, an ambient temperature of 18°C–29°C, relative humidity of 40–70%, and free access to water and food. Mice were randomly assigned for biological analyses, and the researchers performed double-blind analysis during the experiments and results evaluation. All animal experiments complied with the relevant regulations of the Laboratory Animal Management and Ethics Committee of Xiamen University (XMULAC20220235).

METHOD DETAILS

Flow cytometry sorting of microglia

For the transcriptomic analysis of adult mouse microglia, 9-month-old WT or *Csf1r*$^{I792T/+}$ mice were perfused with pre-cooled PBS and the single-cell suspensions of the brain tissues were then mechanically prepared using a Dounce homogenizer. The suspensions

CellPress
OPEN ACCESS

were subjected to centrifugation (800 g, 10 min, 4°C) over a 30% Percoll (GE Healthcare) density gradient. The myeloid cell-enriched fractions were harvested from the interphase layer. Cells were stained with CD11b-APC and CD45-FITC antibodies. Microglia ($CD11b^{+}CD45^{low}$) were finally sorted using a MoFlo Astrios EQ 2 (Beckman, USA) cell sorter for subsequent transcriptomic analysis.

For the confirmation of engraftment of GFP^{+} microglia, transplanted mice were perfused with pre-cooled 1×PBS and brain tissues were then dissociated enzymatically and homogenized into a single-cell solution in pre-cooled HBSS mixed with 1% FBS using a glass Dounce homogenizer. Single cell suspensions were centrifuged on a discontinuous Percoll (GE Healthcare) gradient. Monocytes were isolated from the interphase of these layers. Cells were stained with CD11b-APC and CD45-PeCy7 for sorting $CD11b^{+}CD45^{low}$ microglia and $CD11b^{+}CD45^{low}GFP^{+}$ transplanted microglia. Cells were isolated by using CytoFlex S (Beckman, USA).

Primary microglia isolation

Primary microglial cultures were prepared as previously described.[12] Brains from postnatal day 1 (P1) or P2 *Csf1r*$^{GFP/+}$, *Csf1r*$^{+/+}$ or *Csf1r*$^{I792T/+}$ mice were mechanically dissociated. The mixed brain cells were resuspended by DMEM containing 10% FBS and 1% Penicillin/Streptomycin (P/S) (100×) and plated onto a poly-L-lysine-coated 175 cm^2 flask. After two days, the medium was replaced with the complete DMEM containing 25 ng/mL GM-CSF. Subsequently, 5 mL GMCSF-containing medium was added three days later. Primary microglia were harvested by shaking the flask at 200 rpm for 20 min. This step was repeated for further microglia collection every 3 days. Isolated primary microglia were then cultured in a 6-well plate at the density of 2×10^6 cells/well for further experiments. Cells were cultured in a humidified chamber with 5% CO_2 at 37°C. Cells from the first harvest with viability >95% were used for subsequent transplantation experiments.

Microglia depletion and repopulation

To deplete resident microglia in the brain, mice were fed freely with PLX3397-containing chow (SHUYISHUER BIO) at a dose of 600 mg/kg for 3, 5, or 7 days. The microglia were then repopulated by resuming regular diet (RD) for 7 days. Microglia elimination was verified by Iba1 immunofluorescent staining. Mice that subjected to one or two cycles of PLX3397 treatment were used for *Cx3cr1*$^{GFP/+}$ microglia transplantation.

Microglia transplantation

Microglial transplantation was performed as follows. Briefly, 1-month-old or 9-month-old mice received a volume of 2 μL *Cx3cr1*$^{GFP/+}$ microglia at a density of 2×10^4 cells/μL at each injection site (four sites, hippocampus, X/Y/Z, ±1.75/-2.0/-2.0 mm; cortex, X/Y/Z, ±1.75/-2.0/-0.85 mm) for a total of 8×10^4 cells/mouse at a rate of 0.5 μL/min with a 10 μL microinjector. After each injection, the microinjector remained at the injection site for four minutes to ensure that the grafted microglia entered the brain tissue efficiently. Microglia replacement strategies in different figures were listed in Table S4.

RNA-sequencing

The transcriptome RNA libraries were from brain (n = 5 per group) or microglia (n = 3 per group) derived from *Csf1r*$^{+/+}$ or *Csf1r*$^{I792T/+}$ mice. The RNA-seq experiment was performed using the PE150 strategy on the Illumina HiSeq 2500/4000 platform, yielding an average of 10 G reads per sample. Differentially expressed genes (DEGs) between *Csf1r*$^{+/+}$ and *Csf1r*$^{I792T/+}$ microglia were identified and compared using the DESeq2 (v1.30.1) software package (adjusted p-value <0.05, $|\log_2$ fold change$| > 1$). Enrichment analysis of DEGs, including Gene Ontology (GO) analysis, Kyoto Encyclopedia of Genes and Genomes (KEGG), or Gene Set Enrichment Analysis (GSEA), was performed to identify the significant biological pathways or highlighting biological processes with high confidence by using clusterProfiler (v3.18.1) (adjusted p-value <0.05 and false discovery rate (FDR) q-value <0.05) in those genes.[48,49] The ggplot2 (3.5.1) package was used for visualizing the DEGs and enriched pathways. Data from mouse brain samples were used for detailed gene set enrichment analysis and weighted gene co-expression network analysis (WGCNA) (v1.70.3). All analyses were conducted in R version 4.0 or above.

Electrophysiological recording

Mice were deeply anesthetized with isoflurane and the brain was collected and immediately placed in ice-cold artificial cerebrospinal fluid containing the following composition: 64 mM NaCl, 2.5 mM KCl, 1.25 mM NaH_2PO_4, 10 mM $MgSO_4$, 0.5 mM $CaCl_2$, 26 mM $NaHCO_3$, 10 mM D-glucose, and 120 mM Sucrose (pH 7.4, 290–310 mOsm). Coronal brain slices (400 μm) were made with a Leica VT1200S vibrating microtome and transferred to a chamber containing artificial cerebral spinal fluid (126 mM NaCl, 3.5 mM KCl, 1.25 mM NaH_2PO_4, 1.3 mM $MgSO_4$, 2.5 mM $CaCl_2$, 11 mM $NaHCO_3$, and 10 mM Glucose, pH 7.4, 290–300 mOsm) for 1 h at 32°C. The slices were recovered at room temperature (RT) for at least 1 h before recordings were performed. All solutions were bubbled with 5% CO_2/95% O_2. Field potential recordings were recorded by Axon Digidata 1550 (USA) and Diaphragm clamp Amplifier (Axon CNS, Multiclamp 700B, USA). Schafer peripheral inputs in CA3 were stimulated by Concentric Bipolar Electrode (FHC, CBARC75, Inc. Bowdoin, ME, USA). Field excitatory postsynaptic potentials (fEPSPs) of Schaffer lateral pathways in the CA1 were recorded with 800 kΩ-2 MΩ epoxy glass (Sutter Instrument, BF150-86–10, USA). After a 20-min stable baseline recording, long-term potentiation (LTP) was induced by high-frequency stimulation (HFS, two trains of 100-Hz stimuli with an interval of 30 s), followed by continued recording for 60 min.

T/Y-maze test

Mice were allowed to free access to T-maze arms (30 cm × 6 cm × 10 cm) or all three 120° arms of the Y-maze (8 cm × 30 cm × 15 cm) for 5 min. Mice were placed in the center for all tests and the trajectory of mice was collected by Clever Sys system for 5 min. An alternation was defined as a consecutive entry in all three arms and was calculated automatically using CleverSys TopScan Lite, an automated video analysis system (Clever Sys., Inc. Reston, Virginia, USA).

Open-field test

We followed a protocol that has been previously described.[12] Mice were carried to the behavior room at least 30 min before starting the test to habituate to the environment. Mice were placed into the open-field arena (50 cm × 50 cm) to explore freely. Spontaneous activities were monitored for 10 min and the percentage of duration in the center (24 cm × 24 cm) was analyzed using CleverSys TopScan Lite, an automated video analysis system (Clever Sys., Inc. Reston, Virginia, USA).

High plus maze test

The high plus maze contains 2 open arms (35 × 5 cm) and 2 enclosed arms (35 × 5 cm) extending from a central platform measuring (7.5 × 7.5 cm). Tests were carried out in a quiet and dimly lit environment. The apparatus was wiped clean with 75% ethanol between tests. Mice were placed in the center of the plus-maze, facing one of the open arms. Spontaneous activities were monitored for 10 min and the duration in the open arms was analyzed using CleverSys TopScan Lite, an automated video analysis system (Clever Sys., Inc. Reston, Virginia, USA).

Morris water maze test (MWM)

The water maze consists of a pool (110 cm in diameter) (water temperature was kept at 22 ± 1°C) and a platform (10 cm in diameter) submerged 1.0 cm under the water. The MWM test consisted of 6- days of training trials and 1 day of test. During the training trial, mice were allowed to swim for 1 min to arrive at the hidden platform and stay on it for at least 10 s to remember the position of the platform. Mice that were unable to find the platform were guided to it. Mice were trained for 6 consecutive days. On the 7th day, the platform was removed and the test was performed. The duration in the target quarter, platform crossings and swimming speed were recorded and analyzed using CleverSys TopScan Lite, an automated video analysis system (Clever Sys., Inc. Reston, Virginia, USA).

Nesting behavior test

Csf1r$^{+/+}$ or *Csf1r*$^{I792T/+}$ mice were isolated in separate cages prior to the start of the test for 1 day. Nesting behaviors were manually scored according to the standard scale of 0–5.[50,51] 0, no nest shredding (flat nest); 1, not noticeable nest shredding (>90% nestlet untorn); 2, partially nest shredding (50%–90% nestlet untorn); 3, mostly nest shredding (<50% nestlet untorn); 4, recognizable but flat nest shredding (<10% nestlet untorn), 5, high-sided nest with all material shredded ("brooding" nest).

In vivo MRI scanning

Mice were anesthetized with isoflurane and fixed in a magnetic resonance imaging (MRI) matched brain coil (9.4T MRI, Bruker). Mice were maintained anesthesia at 37°C during the scan using 1.5%–2% isoflurane in oxygen/air (50/50, 1 L/min). Two-dimensional 15- to 30-level T2-weighted scans were performed to identify areas where the hippocampus and lateral ventricle were located (0.5 mm slice thickness, image size/data matrix = 256 × 256 pixels, field of view = 30 × 30 mm). The volume of the hippocampus and lateral ventricle were analyzed with the Imaris software (Bitplane, Belfast, UK, version 9.0.1).

Electron microscopy

For electron microscopy, animals were quickly decapitated. The corpus callosum and hippocampus were dissected and shaped into trapezoidal sections. Tissues were then fixed in the electron microscope buffer at 4°C overnight, followed by ethanol dehydration and uranium-saturated solution with Leica EM TP (Leica, Germany). After being embedded with Spurr's resin, ultra-structures of the corpus callosum and hippocampus were visualized and captured by using a transmission electron microscope Hitachi HT-7800 (Japan). The G-ratio of myelinated fiber was quantified by calculating the ratio of the axonal diameter to the myelinated axon diameter by using ImageJ win64 software. At least 100 myelinated axons were calculated and the number of synapses per high-power field (HPF) in each mouse was counted.

Alizarin red S staining

Mice were anesthetized and perfused with pre-cooled PBS, post-fixed in 4% paraformaldehyde (PFA), and then cryo-embedded. Brains were cut coronally into 15-μm-thick slices on a freezing microtome (Leica, CM1950). For alizarin red S staining, samples were incubated in alizarin red S solution for 10 min. The samples were then rinsed with 1×PBS, sealed with resin, and scanned with Leica Aperio Versa 200 to visualize brain calcification.

Immunofluorescent staining

Mouse brain sections (30 μm) were fixed with 4% paraformaldehyde for 30 min, washed with 1×PBS three times, treated with blocking buffer (5% BSA with 0.5% Triton X-100) at room temperature for 1 h, and then incubated with phos-NFH Antibody (1:200 dilution)

or Iba1 antibody (1:200 dilution) overnight at 4°C. Brain sections were then stained with Alexa 488 (green) secondary antibodies (1:500) or Alexa 568 (red) conjugated secondary antibodies (1:500). All fluorescent images were obtained with an Olympus FV1000MPE-B confocal microscope (Japan). The number and the branches of Iba1$^+$ microglia were counted by ImageJ-win64 software (Sholl analysis). The 3D structure of Iba1$^+$ microglial morphology was reconstructed by the Imaris software (Bitplane, Belfast, UK, version 9.0.1).

NFH immunohistochemical staining

Immunohistochemical staining was performed using Biotin-labelled antibodies and an anti-streptomycin peroxidase kit (MXB, Kit-9270) according to the specification. Non-specific binding was blocked by incubating the sections with reagents A and B for ten minutes, respectively. Brain sections were then incubated overnight at 4°C with phos-NFH primary antibody. Sections were then rinsed with 1×PBS and incubated with biotinylated anti-rabbit IgG antibody at room temperature for 10 min (Reagent C). Sections were finally stained with Diaminobenzidine (DAB, MXB, DAB0031, 20 ×) at room temperature. Brain axonal spheroids were visualized and analyzed using Leica Aperio Versa 200.

Western blot

Primary microglia or brain tissues were lysed in RIPA buffer (LABLEAD) containing a 100×proteinase inhibitor cocktail (APE×BIO) and 100×phosphatase inhibitor cocktail (APE×BIO). The total proteins (20 μg for microglia lysates, 50 μg for brain lysates) quantified by BCA Assay kit (Abbkine) were loaded in 8% SDS-PAGE and transferred to 0.22 μm PVDF membranes (Millipore, IPVH00010). The membranes were sequentially incubated with primary or secondary antibodies. Proteins were then visualized by SuperKine ECL detection kit and the blots were quantified by Chemiluminescent Imaging System Azure 300 (USA).

RNA extraction and quantitative polymerase chain reaction (qPCR)

RNA was extracted from brain tissues or primary microglia using RNA isolater Total RNA Extraction Reagent (Vazyme). A total of one microgram RNA was reverse-transcribed into complementary DNA (cDNA) using HIScript III All-in-one RT SuperMix Perfect for qPCR (Vazyme). Target genes were amplified using HamQ Universal SYBR qPCR Master Mix (Vazyme) on the LightCycler 480 SYBR Green I Master (Roche, Mannheim, Germany). Fold change of the target gene mRNA level was calculated using the $2^{-\Delta\Delta CT}$ method with *Actb* for internal control. The primer sequences are listed in Table S5.

Statistical analysis

Graphical and statistical analyses were performed in a double-blinded manner by GraphPad Prism software (San Diego, CA, USA, version 9.5.1). Distributed data are expressed as the mean ± SEM. The unpaired two-tailed Student's *t* test was used for the comparison of two groups. One-way ANOVA post-Dunnett's multiple comparisons test was used for the comparison of more than two groups. p value <0.05 was considered to be statistically significant.

Cell Reports Medicine, Volume *7*

Supplemental information

A strategy of microglia replacement alleviates microgliopathy in a *CSF1R* I794T hotspot mutation mouse model of CSF1R-related disorder

Xin Li, Banglian Hu, Chujun Wu, Ziwei Wang, Hanzheng Fan, Xiaoyan Guan, Sulan Xie, Dadian Chen, Xiaohua Huang, Hao Sun, Yanfang Li, Xian Zhang, Guojun Bu, Zhanxiang Wang, Yun-Wu Zhang, Li Zhong, Zaiqiang Zhang, and Honghua Zheng

Supplementary Figures and figure legends

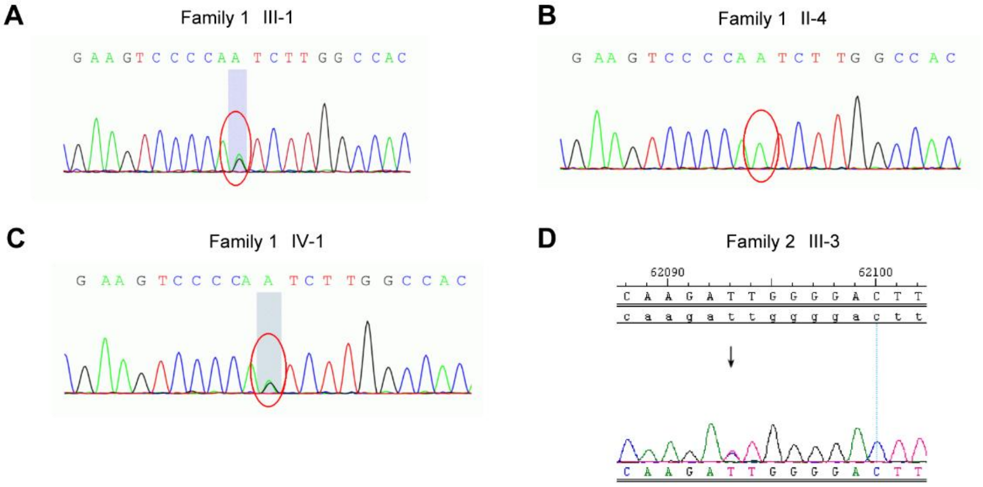

Figure S1. Heterozygous c2381 T>C (p.I794T) missense mutation of the CSF1R gene. Related to Figure 1.

DNA sequencing from four individuals, Family 1 III-1 (A), Family 1 II-4 (B, normal), Family 1 IV-1 (C), and Family 2 III-3 (D) revealed a heterozygous mutation (c2381 T>C/p.I794T) in the *CSF1R* gene. A-C, template strand; D, coding strand.

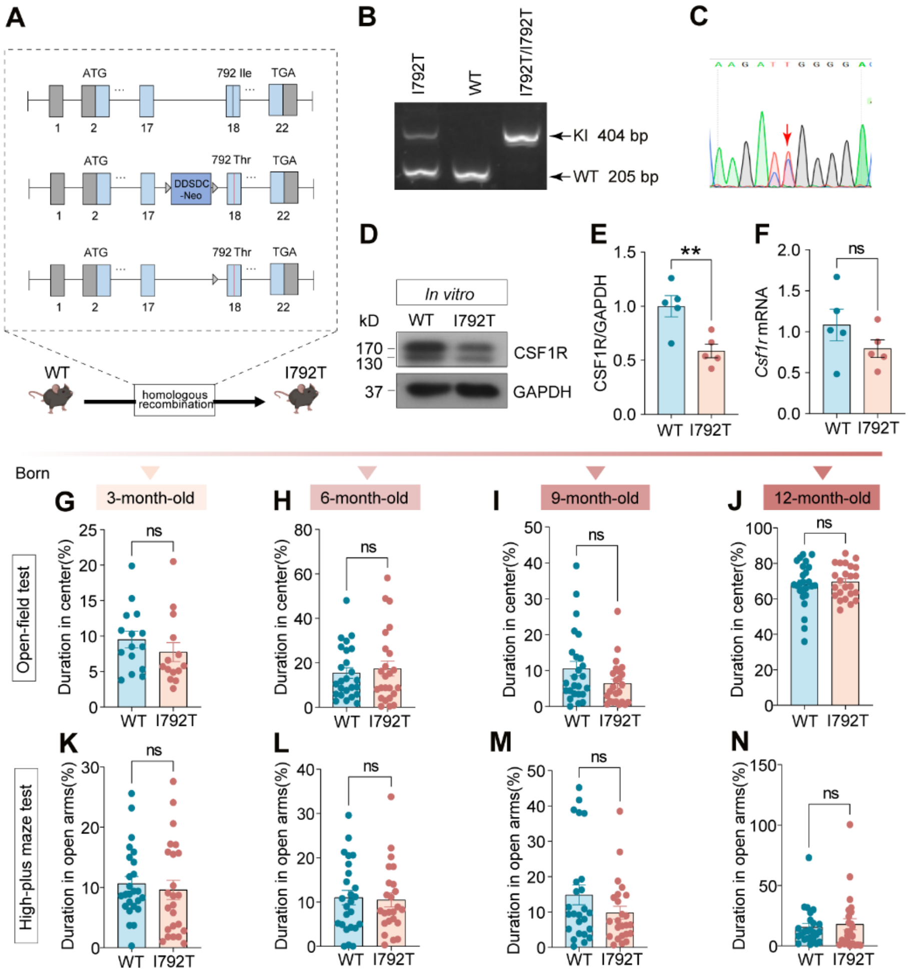

Figure S2. Generation and behavior evaluation of *Csf1r* $^{\mathrm{I792T/+}}$ mice. Related to Figure 2.

(A) Illustration of *Csf1r* $^{\mathrm{I792T/+}}$ mice generation using point mutation homologous recombination-based gene editing.

(B) Genotyping of WT, *Csf1r*$^{\mathrm{I792T/+}}$(I792T) and *Csf1r*$^{\mathrm{I792T/I792T}}$(I792T/I792T) mice by PCR. Wild-type (WT): 205 bp, I792T Knock-in (KI): 404 bp.

(C) DNA sequencing revealed a heterozygous mutation (p.I792T, indicated by a red arrow) in the *Csf1r* gene.

(D) Representative images of Western blot showing the expression of CSF1R in *Csf1r*$^{+/+}$ or *Csf1r*$^{\mathrm{I792T/+}}$ microglia.

(E) CSF1R protein levels quantified by densitometry with GAPDH for comparison, n=5 independent experiments per group, unpaired two-tailed Student's *t*-test.

(F) CSF1R mRNA levels quantified by quantitative PCR with GAPDH for comparison, n=5 independent experiments per group, unpaired two-tailed Student's *t*-test.

(G-J) Behavioral performances in 3-, 6-, 9-, 12-month-old *Csf1r*$^{+/+}$ or *Csf1r*$^{\mathrm{I792T/+}}$ mice assessed by open field test.

(K-N) Behavioral performances in 3-, 6-, 9-, 12-month-old *Csf1r*$^{+/+}$ or *Csf1r*$^{\mathrm{I792T/+}}$ mice assessed by elevated plus maze test. *Csf1r*$^{+/+}$, n =25; *Csf1r*$^{\mathrm{I792T/+}}$, n=24. Unpaired two-tailed Student's *t*-test. All data are presented as mean ± SEM. $^{**}p < 0.01$; ns, not significant.

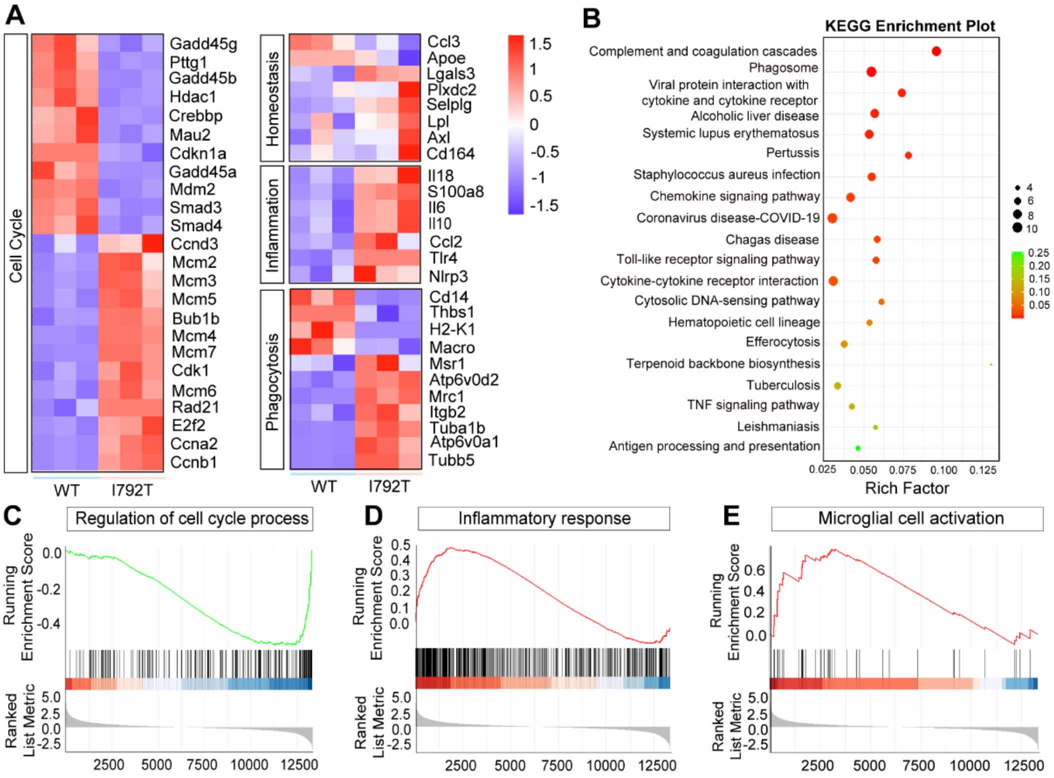

Figure S3. Newborn (0-3 day) *Csf1r* $^{\mathrm{I792T/+}}$ microglia show a phagocytic and proinflammatory phenotype. Related to Figure 4.

(A) Heatmap of differentially expressed genes (DEGs) from bulk RNA-seq of microglia isolated from *Csf1r*$^{+/+}$ or *Csf1r*$^{\mathrm{I792T/+}}$.

(B) Kyoto Encyclopaedia of Genes and Genomes (KEGG) pathway enrichment analysis of DEGs revealed the top twenty significantly altered pathways.

(C-E) Gene set enrichment analysis (GSEA) based on Gene Ontology Biological Process (GOBP) terms showed significant downregulation of cell cycle-related genes (C), and upregulation of genes involved in inflammatory response (D) and microglial activation (E).

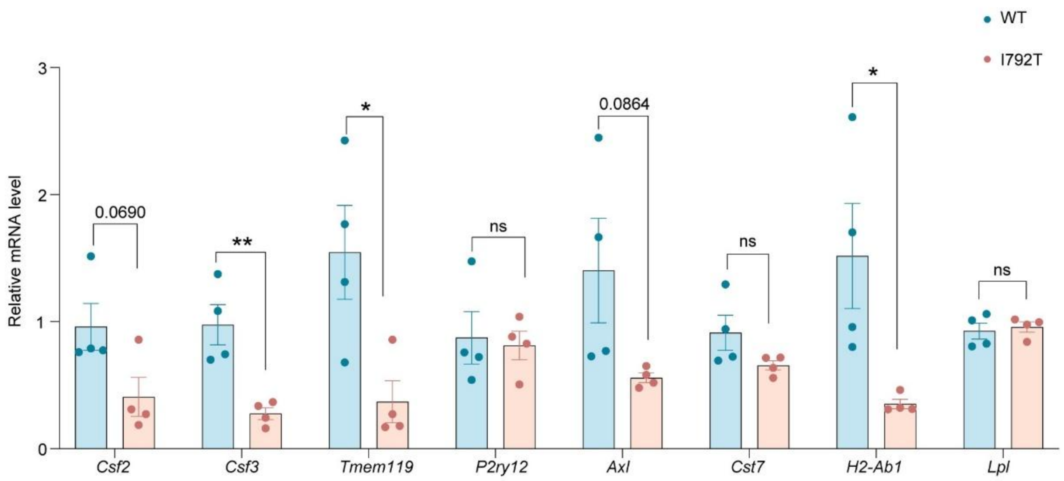

Figure S4. A phenotype of disease-associated microglia was observed in 9-month-old *Csf1r*$^{I792T/+}$ mouse brain. Related to Figure 4.

The mRNA levels of *Csf2*, *Csf3*, and genes indicating DAM including of *P2ry12*, *Tmem119*, *Axl*, *H2-Ab1*, *Lpl*, and *Cst7* were quantified by quantitative PCR. n=4 mice per group, data are presented as mean ± SEM, unpaired two-tailed Student's *t*-test. *p < 0.05; **p < 0.01; ns, not significant.

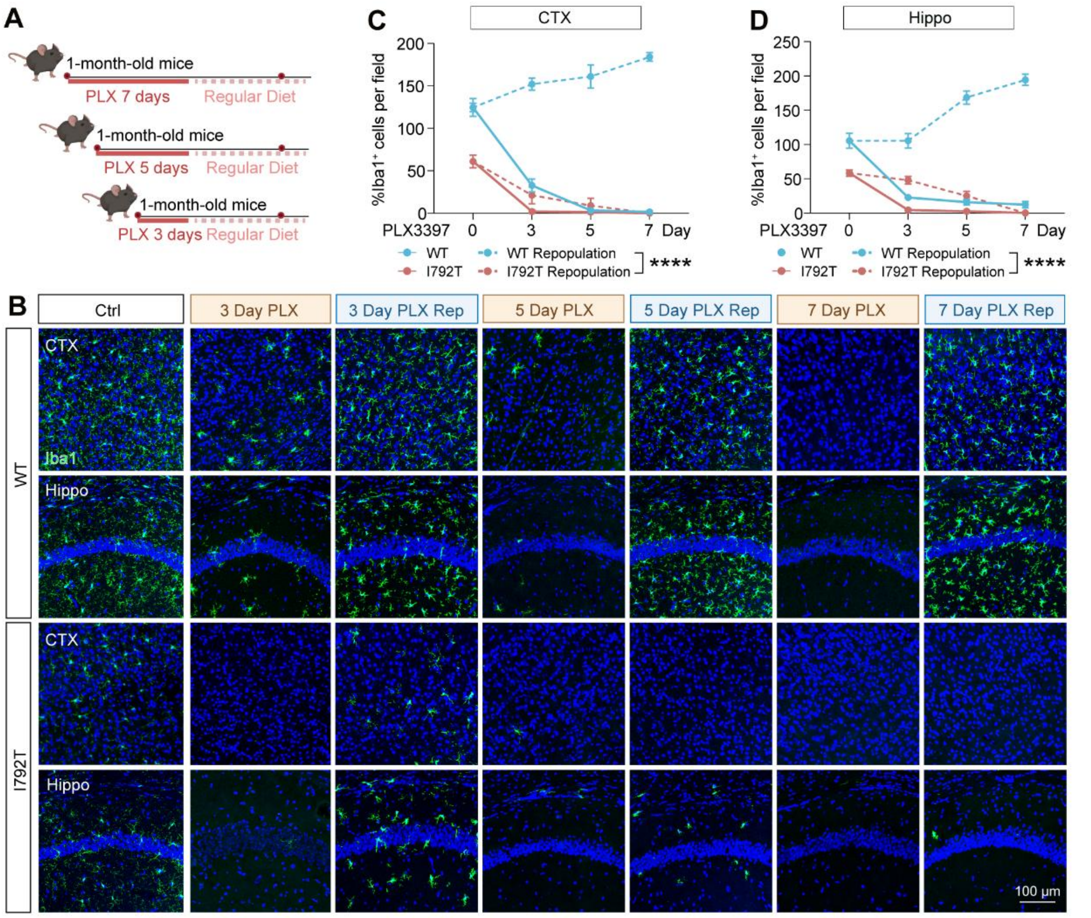

Figure S5. Determination of microglia transplantation window in *Csf1r* $^{I792T/+}$ mouse model. Related to Figure 5.

(A) Schematic diagram of *Csf1r* $^{I792T/+}$(I792T) microglia elimination strategy. One-month-old *Csf1r*$^{+/+}$ or *Csf1r*$^{I792T/+}$ male mice were orally treated with 600 mg/kg PLX3397 for three, five, or seven days, followed by microglia repopulation for one week (7-day regular diet). Mice were sacrificed and the brains were subjected to Iba1 immunofluorescent staining.

(B) Representative microglia (Iba1$^+$, green) immunofluorescent images of coronal brain slices from those one-month-old *Csf1r*$^{+/+}$ or *Csf1r*$^{I792T/+}$ male mice. Scale bar, 100 μm.

(C) The number of Iba1$^+$ microglia was quantified in the cortex of those one-month-old *Csf1r*$^{+/+}$ or *Csf1r* $^{I792T/+}$ male mice.

(D) The number of Iba1$^+$ microglia was quantified in the hippocampus of those one-month-old *Csf1r*$^{+/+}$ or *Csf1r*$^{I792T/+}$ male mice. One-way ANOVA post-Dunnett's multiple comparisons test. ****$p < 0.0001$. n=3 mice per group, data are presented as mean ± SEM.

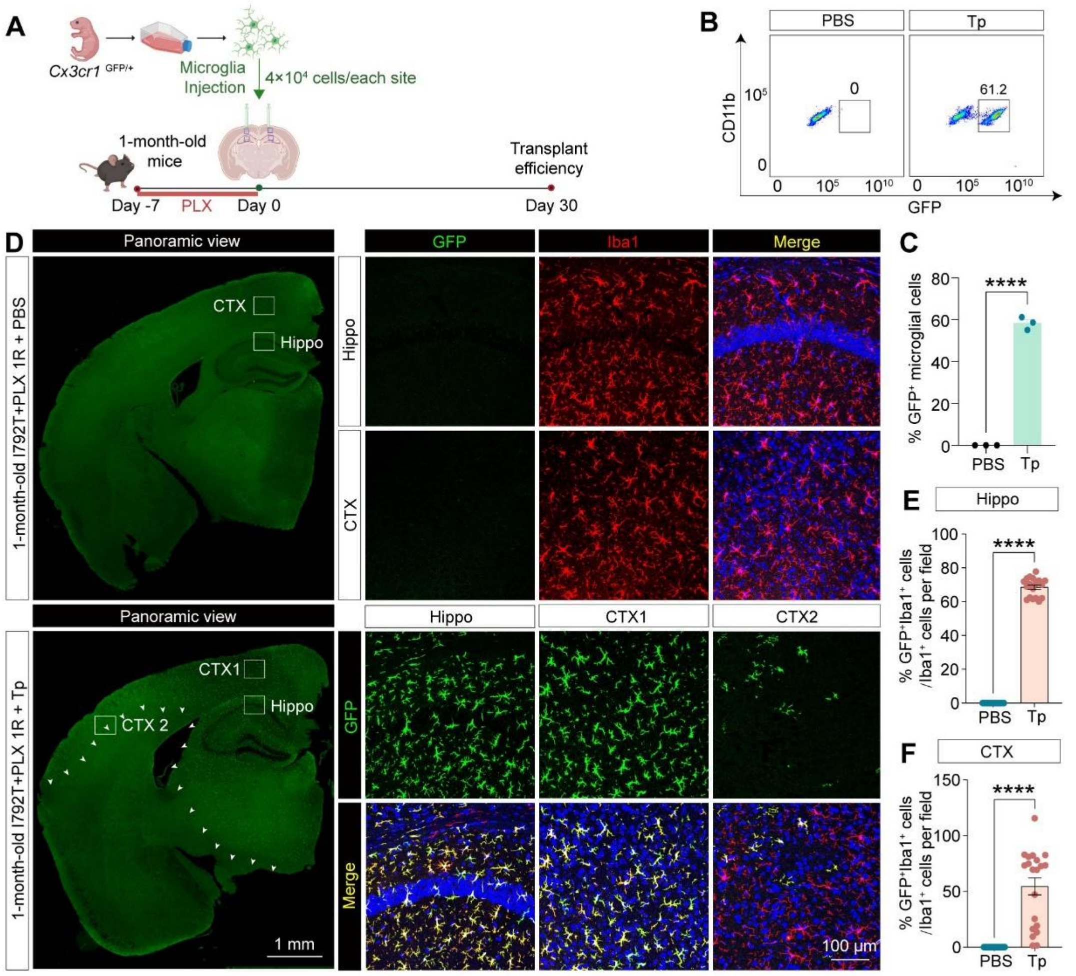

Figure S6. Determination of microglia transplantation efficiency in *Csf1r* $^{I792T/+}$ mouse model. Related to Figure 5.

(A) Workflow of microglia elimination and transplantation strategy. One-month-old *Csf1r*$^{+/+}$ or *Csf1r*$^{I792T/+}$ male mice were orally treated with 600 mg/kg PLX3397 for seven days, followed by microglia repopulation for one week. Mice *Cx3cr1*$^{GFP/+}$ microglia were then transplanted into the brain of those mice. Mice were sacrificed one month later and the brains were subjected to Iba1 immunofluorescent staining (red).

(B) Representative dot plots of GFP$^+$Iba1$^+$ microglia isolated from *Csf1r* $^{I792T/+}$ mouse brain transplanted with GFP$^+$Iba1$^+$ microglia or PBS via flow cytometry.

(C) The percentage of GFP$^+$Iba1$^+$ microglia relative to the total Iba1$^+$ microglia was only up to 61.2% in *Csf1r* $^{I792T/+}$ mouse brain transplanted with GFP$^+$ microglia.

(D) Representative GFP$^+$Iba1$^+$ microglia immunofluorescent images of coronal brain slices of two-month-old *Csf1r*$^{+/+}$ or *Csf1r*$^{I792T/+}$ male mice, one month post- intracerebral injection with PBS, or *Cx3cr1*$^{GFP/+}$ microglia (Tp).

(E) Quantification of the percentage of GFP$^+$Iba1$^+$ microglia relative to Iba1$^+$ cells per field in the hippocampus of *Csf1r*$^{I792T/+}$ mouse brain.

(F) Quantification of the percentage of GFP$^+$Iba1$^+$ microglia relative to Iba1$^+$ cells per field in the cortex of *Csf1r*$^{I792T/+}$ mouse brain. Unpaired two-tailed Student's *t*-test. n=3 mice per group, data are presented

as mean ± SEM. ****$p < 0.0001$.

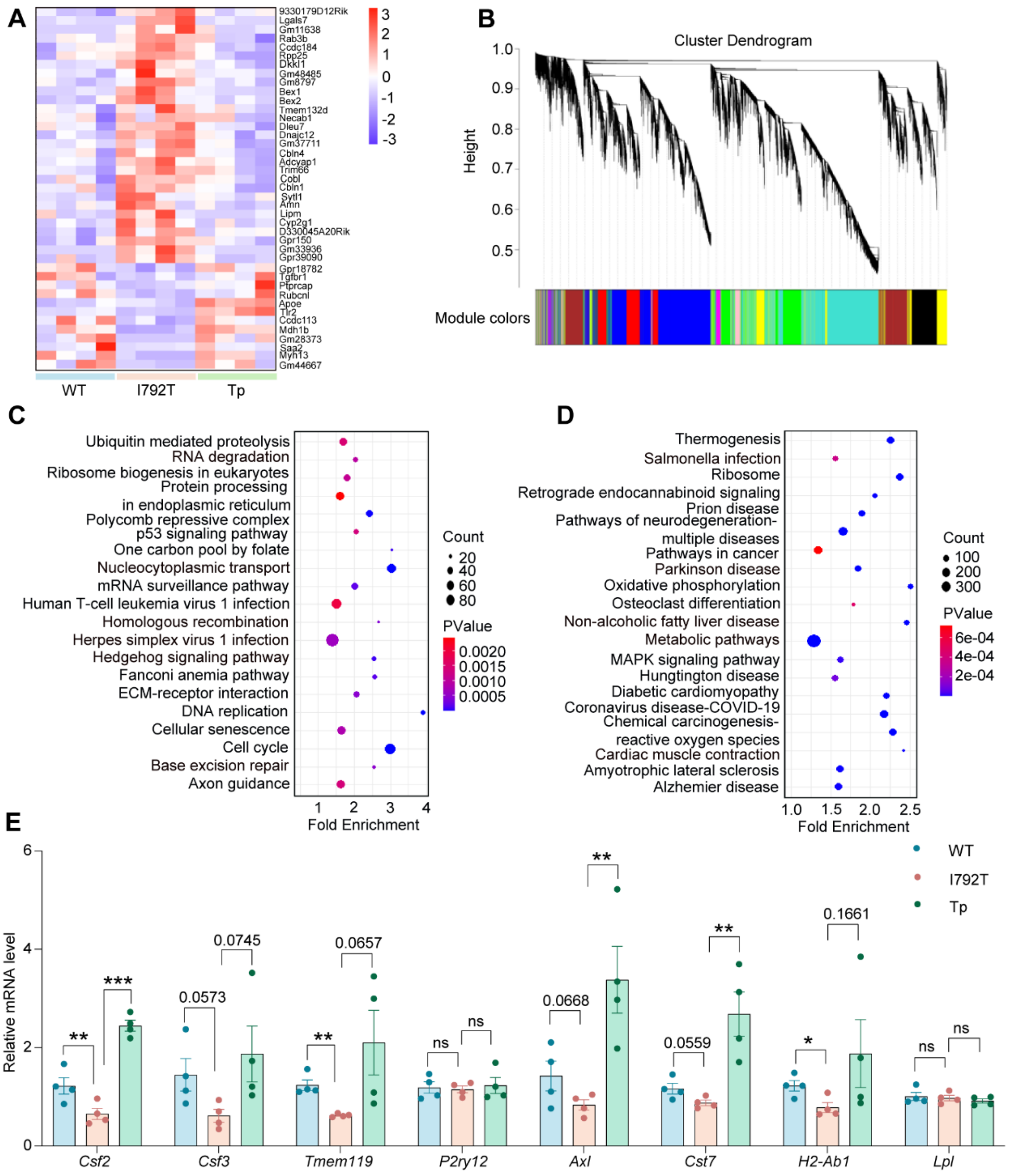

Figure S7. The gene sets in *Csf1r*$^{I792T/+}$ mice transplanted with microglia are similar to those in *Csf1r*$^{+/+}$ mice. Related to Figure 7.

(A) The heatmap of differentially expressed genes (DEGs) in bulk RNA-seq from *Csf1r*$^{+/+}$ brain, *Csf1r* $^{I792T/+}$ brain with or without microglial transplantation. Genes related to disease-associated microglia are highlighted in red.

(B) WGCNA in the forebrain of *Csf1r*$^{+/+}$ brain, *Csf1r* $^{I792T/+}$ brain with or without microglial transplantation. Color bands show gene modules obtained by automatic single-block analysis.

(C-D) KEGG plots of DEGs in the two most representative modules in the WGCNA dendrogram. (C) The top twenty high-confidence KEGG terms in the blue module are shown. (D) The top twenty high-confidence KEGG terms in the turquoise module are shown.

(E) The transcriptional levels of *Csf2/3* and DAM genes including *Tmem119*, *Axl*, *Cst7* in those mice

brains were quantified by RT-qPCR. Indicated decreased genes (*Csf2/3*, *Tmem119*, *Axl*) in *Csf1r*$^{I792T/+}$ brain were reversed by microglial transplantation. WT, *Csf1r*$^{+/+}$ mice; I792T, *Csf1r*$^{I792T/+}$ mice; Tp, *Csf1r*$^{I792T/+}$ mice transplanted with microglia; WGCNA, weighted gene co-expression network analysis; KEGG, Kyoto Encyclopedia of Genes and Genomes. n=4 mice per group, data are presented as mean ± SEM. One-way ANOVA post-Dunnett's multiple comparisons test. *$p < 0.05$; **$p < 0.01$; ***$p < 0.001$; ns, not significant.

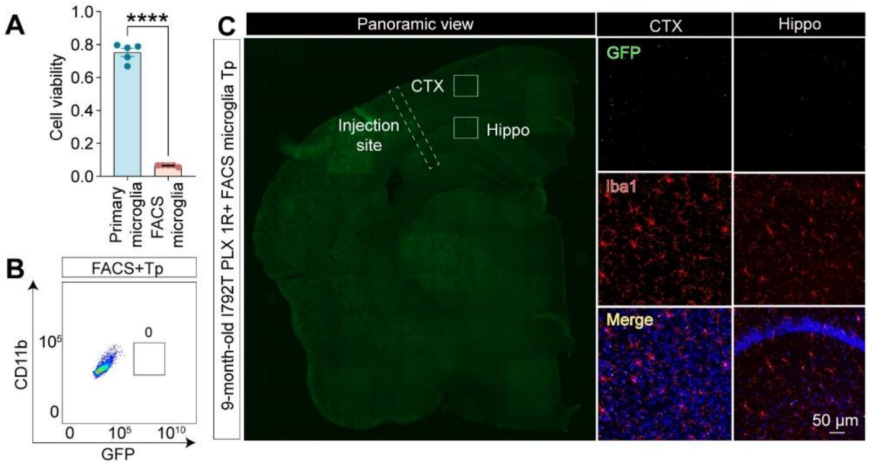

Figure S8. Isolated microglia from P1 mice via fluorescence-activated cell sorting (FACS) were unsuitable for transplantation experiments. Related to Figures 5-7.

(A) The sorted microglia population exhibited low viability in CCK8 assays compared with primary cultured microglia.

(B) GFP^+CD11b^+ microglia were isolated from P1 *Cx3cr1*$^{GFP/+}$ mice via FACS and these microglia were transplanted into *Csf1r* $^{I792T/+}$ mouse brain. One month later, the brains were dissected and no GFP^+CD11b^+ microglia were isolated from those transplanted *Csf1r* $^{I792T/+}$ mouse brain via flow cytometry.

(C) No GFP^+Iba1^+ microglia were observed in the immunofluorescent images of coronal brain slices from those *Csf1r*$^{I792T/+}$ mice, one month post- intracerebral injection with isolated GFP^+Iba1^+ microglia. Unpaired two-tailed Student's t-test. n=5 experiments or mice per group, data are presented as mean ± SEM. ****$p < 0.0001$.

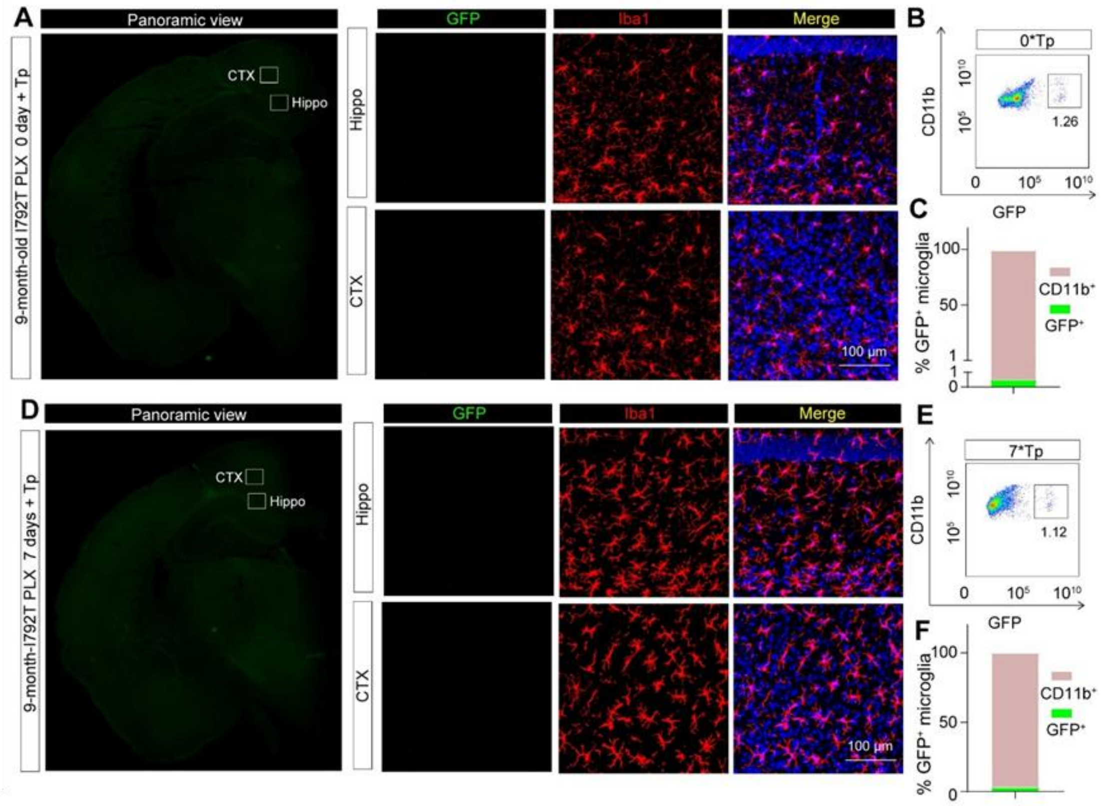

Figure S9. Transplanted GFP$^+$Iba1$^+$ microglia failed to localized into brains of 9-month-old *Csf1r*$^{I792T/+}$ mice with no microglial depletion or with single microglial depletion. Related to Figures 5-7.

(A) GFP$^+$Iba1$^+$ microglia were transplanted into *Csf1r* $^{I792T/+}$ mice without microglial depletion. One month later, the brains were dissected and few GFP$^+$Iba1$^+$ microglia were observed in the immunofluorescent images of coronal brain slices from those *Csf1r*$^{I792T/+}$ mice.

(B-C) Few GFP$^+$CD11b$^+$ microglia were isolated from those transplanted *Csf1r*$^{I792T/+}$ mouse brain without microglial depletion via flow cytometry.

(D) GFP$^+$Iba1$^+$ microglia were transplanted into *Csf1r*$^{I792T/+}$ mice with single microglial depletion. One month later, the brains were dissected and few GFP$^+$Iba1$^+$ microglia were observed in the immunofluorescent images of coronal brain slices from those *Csf1r*$^{I792T/+}$ mice.

(E-F) Few GFP$^+$CD11b$^+$ microglia were isolated from those transplanted *Csf1r*$^{I792T/+}$ mouse brain with single microglial depletion via flow cytometry.

Data are represented as mean ± SEM. 0*Tp: GFP$^+$Iba1$^+$ microglia were transplanted into *Csf1r*$^{I792T/+}$ mice without microglial depletion; 7*Tp: GFP$^+$Iba1$^+$ microglia were transplanted into *Csf1r*$^{I792T/+}$ mice with single microglial depletion.

Table S1. Clinical details of CSF1R-RD patients with CSF1R p.I794T variants. Related to Figure 1.

Patient	Clinically recorded gender	Ethnicity	AAO	AAE	AAD	Disease duration (years)
1 (This study)	M	Chinese	35	36	39	4
2 (This study)	M	Chinese	27	28	Alive	Alive
3 (This study)	M	Chinese	39	42	Alive	Alive
4	F	Chinese	32	34	Alive	Alive
5	F	Chinese	41	NA	46	5
6	M	Chinese	37	NA	39	2
7	M	Chinese	43	NA	Alive	Alive
8	M	Chinese	42	43	Alive	3/Alive
9	F	Chinese	42	44	Alive	2/Alive
10	F	Chinese	35	37	Alive	2/Alive
11	M	Chinese	37	38	Alive	1/Alive
12	M	Chinese	39	39	Alive	1/Alive
13	F	Chinese	35	37	Alive	2/Alive
14	F	Chinese	42	43	Alive	1/Alive
15	F	Chinese	41	42	Alive	14M/Alive
16	M	Chinese	37	38	Alive	1/Alive
17	F	Chinese	41	43	Alive	2/Alive
18	M	Chinese	46	46+4M	Alive	4M/Alive
19	F	Chinese	NA	46	Alive	NA
20	F	Chinese	35	37	Alive	2/Alive
21	M	Chinese	40	42	Alive	2/Alive
22	M	Chinese	51	53	Alive	2/Alive
23	M	Chinese	60	62	Alive	2/Alive
24	F	Chinese	42	43	Alive	1/Alive
25	M	Chinese	31	31	Alive	1/Alive
26	M	Japanese	60	61	Alive	8M/Alive
27	M	Japanese	46	48	Alive	2/Alive
28	F	Japanese	33	36	Alive	3/Alive
29	M	Japanese	49	50	Alive	1/Alive
30	F	Japanese	34	35	Alive	1/Alive
31	F	Japanese	60	70	Alive	Alive
32	M	Japanese	40	46	Alive	6/Alive
33	F	Japanese	55	NA	Alive	14/Alive
34	F	Japanese	28	28+6M	Alive	6M/Alive
35	F	Japanese	27	NA	Alive	Alive
36-41	7NA	Japanese	NA	NA	NA	NA
42	F	Japanese	20	26	Alive	6/Alive

43	F	Japanese	20	20+5M	Alive	5M/Alive
44	M	Japanese	40	46	Alive	6/Alive
45	M	Japanese	40	42	Alive	2/Alive
46	M	Korean	51	55	Alive	4/Alive
47	M	Korean	60	61	Alive	1/Alive
48	F	Korean	51	55	Alive	4/Alive
49	F	Korean	60	61	Alive	1/Alive
50	M	Korean	51	52	Alive	1/Alive
51	M	Indian	40	40+9M	Alive	9M/Alive
52	F	Indian	33	34	Alive	1/Alive
53	F	American	33	36	Alive	3/Alive
54	M	American	43	56	Alive	13/Alive
55	F	American	37	NA	40	3
56	F	American	35	36	Alive	Alive
57	NA	American	35	NA	Alive	Alive
58	M	American	60	63	Alive	3/Alive
59	M	French	57	5	NA	NA
60	M	French	28	32	NA	NA
61	F	French	28	4	NA	NA
62	F	Germany	40	NA	43	3
63	F	Germany	29	29+4M	Alive	4M/Alive
64	M	British	52	NA	Alive	Alive
65	M	The Netherlands	46	NA	49	3
66	M	Ireland	41	41+4M	Alive	4M/Alive

Abbreviations: CSF1R-RD = CSF1R-related disorder; AAO = Age at onset(Y); AAE = Age at examination(Y); AAD = Ag[illegible]
patients were unable to participate in the test.

Cognitive performance		Family history	Initial symptoms	Cognitive impairment	Psychiatric symptoms	Parkinsonism
MMSE	MoCA					
NA	24	+	Cognitive impairment	+	-	-
NA	NA	+	Spastic hemiplegia	-	-	-
3	NA	+	Psychiatric symptoms	+	+	-
23	NA	+	NA	+	+	-
NA	NA	+	Cognitive impairments	+	NA	+
NA	NA	+	Cognitive impairment	+	NA	-
27	23	-	Walking difficulties	-	NA	-
14	12	+	Depression	+	+	-
15	NA	+	Cognitive impairments, gait dysfunction	+	+	+
15	19	+	Dysarthria, gait dysfunction	+	+	+
NA	NA	+	Cognitive decline, gait dysfunction	+	+	-
28	25	+	Memory loss	+	-	-
19	15	NA	Cognitive impairments, gait dysfunction, slurred speech	+	NA	+
15	NA	NA	mpairments, gait (	+	NA	-
NA	NA	-	NA	+	-	-
NA	NA	+	NA	+	+	-
15	13	+	Apathy	+	+	-
NA	NA	NA	Personality change	NA	+	NA
NA	NA	NA	NA	NA	NA	NA
19	15	NA	NA	+	+	+
28	25	NA	NA	+	+	-
21	14	NA	NA	+	-	+
15	12	NA	NA	+	+	-
NA	NA	-	Apathy, gait dysfunction, urinary incontinence, memory loss	+	+	-
NA	23	NA	NA	+	-	-
29	27	+	Gait dysfunction	-	-	-
NA	NA	+	Depression	+	+	-
NA	NA	+	Gait dysfunction	+	-	+
NA	NA	+	Aphasia	+	-	-
NA	NA	-	Cognitive impairment, depression	+	+	+
NA	NA	+	Forgetfulness	+	+	-
NA	NA	-	Cognitive impairment	+	+	-
NA	NA	+	Cognitive impairment	+	+	+
14	NA	-	Gait dysfunction,	+	NA	-
NA	NA	-	NA	+	+	NA
NA	NA	NA	NA	NA	NA	NA
NA	NA	NA	Cognitive impairment	NA	NA	NA

30	NA	+	Progressive right spastic hemiplegia	-	+	-
NA	NA	-	Cognitive impairments	+	-	-
NA	NA	NA	NA	NA	NA	NA
NA	NA	NA	NA	+	+	+
NA	NA	NA	NA	+	+	-
NA	NA	-	Cognitive impairment	+	+	-
NA	NA	-	Cognitive impairment, gait dysfunction	+	-	+
NA	NA	-	Personality change	+	+	+
NA	NA	-	Cognitive impairments	+	+	-
NA	NA	NA	Progressive apathy, executive dysfunction, and memory	-	+	-
NA	NA	-	Weakness in the right hand	+	-	-
NA	NA	-	Apathy	+	NA	-
NA	NA	+	Cognitive impairments	+	+	-
NA	NA	+	Cognitive impairments	+	+	+
NA	NA	NA	Cognitive impairments	+	NA	±
NA	NA	+	Gait dysfunction	+	+	-
NA	NA	+	Slurred speech	NA	NA	NA
NA	NA	+	Gait dysfunction	NA	NA	NA
NA	NA	-	Gait dysfunction	NA	NA	NA
NA	NA	+	Personality change, apathy, clumsiness	NA	+	NA
NA	NA	-	ysarthria and cogı	+	-	-
NA	NA	-	Cognitive impairments	+	NA	+
NA	NA	-	NA	+	-	-
NA	26	-	Aphasia and bifrontal headache	+	-	+

e at death(Y); MMSE = Mini Mental Status Examination; MoCA = Montreal Cognitive Assessment; + means positive; – me

Ataxia	Pyramidal signs	Epilepsy	Ref.
-	+	NA	This study
-	+	NA	This study
-	+	NA	This study
+	+	-	1
NA	-	-	2
NA	+	-	2
NA	+	-	2
-	-	-	3
NA	NA	-	4
NA	NA	-	4
NA	NA	-	4
NA	NA	-	4
+	NA	-	5
NA	NA	-	5
-	+	-	6
NA	NA	-	7
-	-	+	8
NA	NA	NA	9
NA	NA	NA	10
+	NA	NA	11
-	NA	NA	11
-	NA	NA	11
+	NA	NA	11
-	-	-	12
-	-	-	13
-	-	-	14
NA	-	NA	15
NA	-	NA	15
NA	-	NA	15
NA	-	NA	15
NA	NA	-	16
NA	+	+	17
NA	+	+	17
+	+	-	18
NA	+	+	18
NA	NA	NA	19
NA	NA	NA	20

-	+	-	21
-	+	-	22
NA	NA		23
NA	NA	+	24
-	-	+	24
NA	-	-	25
NA	-	+	25
NA	-	+	25
-	-	-	26
NA	NA	NA	27
-	+	-	28
NA	+	+	28
-	-	-	29
NA	NA	+	30
NA	+	-	31
-	NA	-	32
NA	NA	NA	33
NA	NA	NA	33
NA	NA	NA	33
NA	+	+	34
+	+	-	35
NA	+	NA	36
-	-	-	37
-	+	-	38

eans negative; ± means probable; NA = not available, because the

Table S2. Imaging details of CSF1R-RD patients with CSF1R p.I794T variants. Related to Figure 1.

Patient	Clinically recorded gender	Ethnicity	White matter lesions	Dilated lateral ventricles	Corpus callosum atrophy	Cortical atrophy predominantly in frontal/parietal lobes
1 (This study)	M	Chinese	+	+	+	NA
2 (This study)	M	Chinese	+	+	+	NA
3 (This study)	M	Chinese	+	+	+	NA
4	F	Chinese	+	-	-	+
5	F	Chinese	+	-	+	+
6	M	Chinese	+	-	+	+
7	M	Chinese	+	-	+	+
8	M	Chinese	+	+	NA	+
9	F	Chinese	+	NA	+	-
10	F	Chinese	+	NA	+	+
11	M	Chinese	+	NA	+	+
12	M	Chinese	+	NA	-	-
13	F	Chinese	+	+	+	+
14	F	Chinese	+	+	+	+
15	F	Chinese	+	NA	NA	NA
16	M	Chinese	+	-	-	+
17	F	Chinese	+	-	+	+
18	M	Chinese	NA	NA	+	NA
19	F	Chinese	NA	NA	NA	NA
20	F	Chinese	NA	NA	NA	+
21	M	Chinese	NA	NA	NA	+
22	M	Chinese	NA	NA	NA	+
23	M	Chinese	NA	NA	NA	+
24	F	Chinese	+	NA	NA	NA
25	M	Chinese	+	-	+	+
26	M	Japanese	+	+	+	+
27	M	Japanese	±	+	-	NA
28	F	Japanese	+	+	+	NA
29	M	Japanese	+	+	+	NA
30	F	Japanese	+	+	+	NA
31	F	Japanese	+	NA	NA	+
32	M	Japanese	+	NA	+	NA
33	F	Japanese	+	NA	+	NA
34	F	Japanese	+	NA	+	+
35	F	Japanese	NA	NA	+	+
36-41	7NA	Japanese	NA	NA	NA	NA
42	F	Japanese	NA	NA	NA	NA
43	F	Japanese	+	-	NA	+
44	M	Japanese	+	+	+	+
45	M	Japanese	+	+	+	NA
46	M	Korean	+	+	+	+
47	M	Korean	+	+	+	+
48	F	Korean	NA	NA	NA	NA
49	F	Korean	NA	NA	NA	NA
50	M	Korean	NA	NA	NA	NA
51	M	Indian	+	+	+	NA
52	F	Indian	+	-	+	NA
53	F	American	+	NA	-	NA
54	M	American	+	NA	+	NA
55	F	American	+	+	+	+
56	F	American	+	+	NA	+
57	NA	American	NA	NA	NA	NA
58	M	American	+	NA	+	+
59	M	French	NA	NA	NA	NA
60	M	French	NA	NA	NA	NA

61	F	French	NA	NA	NA	NA
62	F	Germany	+	NA	+	NA
63	F	Germany	+	NA	NA	NA
64	M	British	NA	NA	NA	NA
65	M	The Netherlands	+	-	-	NA
66	M	Ireland	+	-	NA	+

Abbreviations: DWI = Diffusion weighted imaging, CT = Computed Tomography, + means positive, – means negative, NA [illegible]
test

Punctate DWI hyperintensities	White matter calcifications (on CT scan)	Fulfilling diagnostic criteria according to Konno et al.[39]	Ref.
+	NA	Probable	This study
+	-	Probable	This study
+	NA	Probable	This study
+	+	Probable	1
+	NA	Probable	2
-	-	Probable	2
+	+	Probable	2
+	NA	Probable	3
NA	-	Probable	4
NA	-	Probable	4
NA	-	Probable	4
NA	-	Probable	4
NA	NA	Probable	5
NA	NA	Probable	5
+	+	Probable	6
NA	NA	Probable	7
+	NA	Probable	8
-	NA	Probable	9
NA	NA	Probable	10
NA	NA	Probable	11
NA	NA	Probable	11
NA	NA	Probable	11
NA	NA	Probable	11
+	NA	Probable	12
+	NA	Probable	13
NA	-	Probable	14
-	+	Possible	15
-	+	Probable	15
-	-	Possible	15
+	+	Probable	15
NA	NA	Probable	16
NA	+	Probable	17
NA	NA	Probable	17
NA	NA	Probable	18
NA	NA	Probable	18
NA	NA	Probable	19
NA	NA	Probable	20
+	NA	Probable	21
NA	+	Probable	22
NA	+	Probable	23
NA	NA	Probable	24
NA	NA	Probable	24
NA	NA	Probable	25
NA	NA	Probable	25
NA	NA	Probable	25
-	NA	Probable	26
+	NA	Probable	27
+	+	Probable	28
+	NA	Probable	28
NA	NA	Probable	29
NA	NA	Probable	30
NA	NA	Probable	31
NA	NA	Probable	32
NA	NA	Probable	33
NA	NA	Probable	33

NA	NA	Probable	33
NA	NA	Probable	34
NA	+	Probable	35
NA	NA	Probable	36
NA	NA	Probable	37
+	NA	Probable	38

[illegible]A = not available, because the patients were unable to participate in the

Table S3. Age of onset and disease duration in non-I794T patients. Related to Figure 1.

No.	Cases	Protein domain	CSF1R	AO	cally recorded ge	Duration
1	1	Signal peptide	p.G17C	25	M	6
	2		p.G17C	NA	NA	NA
2	3	EC	p.P54Q	51	F	NA
3	4	EC	p.S66G	NA	F	NA
4	5	EC	p.A74T	58	F	10/Alive
5	6	EC	p.T79M	86	M	1
6	7	EC	p.A96Pfs*17	NA	NA	NA
7	8	EC	p.P104Lfs*8	22	F	2/Alive
8	9	EC	p.P132L	NA	M	5/Alive
9	10	EC	p.Q176*	55	F	1/Alive
10	11	EC	p.H239fs	49	F	4/Alive
11	12	EC	p.C278*	47	F	2/Alive
12	13	EC	p.V279M	14	M	8/Alive
13	14	EC	p.C653Y	48	F	15
	15		p.C653Y	48	F	15
14	16	EC	p.H362R	60	M	NA
14	17		p.H362R	51	M	NA
14	18		p.H362R	56	M	NA
14	19		p.H362R	51	F	NA
14	20		p.H362R	41	F	NA
14	21		p.H362R	60	F	NA
14	22		p.H362R	25	M	4/Alive
15	23	EC	p.T363N	45	M	5/Alive
16	24	EC	p.V383L	45	M	9M/Alive
17	25	EC	p.E403Rfs*37	47	F	3/Alive
18	26	EC	p.L418X	68	F	5/Alive
19	27	EC	p.Q426fs	54	F	1/Alive
20	28	EC	p.Q433*	22	F	5/Alive
21	29	EC	p.E478K	NA	NA	NA
22	30	EC	p.Q481*	NA	M	5/Alive
23	31	EC	p.T507_H508InsP	NA	NA	NA
24	32	TM	p.L536V	55	F	NA
25	33	JMD	p.Y540*	NA	NA	NA
26	34	JMD	p.R549H	64	NA	NA
27	35	JMD	p.D565N	NA	NA	NA
28	36	JMD	p.T567fs*44	24	F	29
29	37	JMD	p.T567M	34	F	NA
30	38	JMD	p.E573K	NA	M	NA
30	39		p.E573K	78	NA	NA
31	40	JMD	p.R579Q	54	F	NA
31	41		p.R579Q	41	M	14M/Alive
32	42	JMD	p.R579W	39	M	2
33	43	JMD	p.N581Kfs	42	F	3/Alive
34	44	TKD1	p.L582P	45	M	9M
34	45		p.L582P	NA	M	NA
35	46	TKD1	p.G585_K619delinsA	36	F	4
36	47	TKD1	p.K586*	79	M	3
37	48	TKD1	p.G589E	60	M	10
37	49		p.G589E	58	M	3
37	50		p.G589E	47	F	11

37	51		p.G589E	58	M	2/Alive
37	52		p.G589E	47	F	5/Alive
38	53	TKD1	p.G589R	48	F	NA
38	54		p.G589R	37	F	NA
38	55		p.G589R	40	M	NA
38	56		p.G589R	27	F	30M/Alive
38	57		p.G589R	37	F	4/Alive
38	58		p.G589R	44	F	3/Alive
38	59		p.G589R	43	F	3/Alive
38	60		p.G589R	30	M	3/Alive
38	61		p.G589R	39	F	1/Alive
38	62		p.G589R	NA	NA	NA
38	63		p.G589R	NA	NA	NA
38	64		p.G589R	NA	F	NA
38	65		p.G589R	NA	F	NA
39	66	TKD1	p.E596M	25	F	10
40	67	TKD1	p.E598del	43	F	1/Alive
41	68	TKD1	p.A601P	29	F	2/Alive
42	69	TKD1	p.V613L	51	F	NA
43	70	TKD1	p.V613M	46	M	2/Alive
43	71		p.V613M	51	F	3/Alive
44	72	TKD1	p.L618del	39	F	1/Alive
45	73	TKD1	p.S620delins40	28	F	11/Alive
46	74	TKD1	p.K627del	28	F	11/Alive
47	75	TKD1	p.L630R	45	M	7
48	76	TKD1	p.E633K	44	F	6M/Alive
48	77		p.E633K	46	F	8
48	78		p.E633K	42	F	4
48	79		p.E633K	41	F	4/Alive
48	80		p.E633K	29	NA	NA
48	81		p.E633K	NA	F	NA
48	82		p.E633K	73	M	2/Alive
49	83	TKD1	p.L634R	53	F	NA
50	84	TKD1	p.I636N	29	M	2
51	85	TKD1	p.H643Q	12	M	12/Alive
52	86	TKD1	p.V647del	35	M	0.75/Alive
53	87	TKD1	p.G651E	40	M	3M/Alive
53	88		p.G651E	45	F	1/Alive
53	89	TKD1	p.G651E	40	M	3/Alive
54	90	TKD1	p.G651R	40	F	1/Alive
55	91	TKD1	p.A652P	30	F	1/Alive
55	92		p.A652P	NA	NA	NA
56	93	TKD1	p.C653R	43	F	5/Alive
57	94	TKD1	p.P658Sfs*24	NA	M	NA
58	95	TKD1	p.L660P	47	M	2/Alive
59	96	TKD1	p.I662T	40	M	6/Alive
60	97	TKD1	p.E664K	35	F	NA
61	98	TKD1	p.C666R	37	M	12/Alive
62	99	TKD1	p.D670N	53	F	4/Alive
63	100	TKD1	p.R676*	30	F	2
63	101		p.R676*	31	F	1
63	102		p.R676*	30	F	2
64	103	KID	p.S688Efs*13	41	F	13

65	104	KID	p.Q691H	82	F	7
66	105	KID	p.E694K	NA	NA	NA
67	106	KID	p.H703Y	79	NA	3
68	107	KID	p.R710H	NA	NA	NA
69	108	KID	p.G747R	NA	NA	NA
70	109	TKD2	p.L755P	27	F	31M
71	110	TKD2	p.L756P	34	M	16M/Alive
72	111	TKD2	p.F758S	23	F	NA
73	112	TKD2	p.S759F	55	M	7
73	113		p.S759F	NA	NA	NA
73	114		p.S759F	NA	NA	NA
74	115	TKD2	p.A763P	45	M	6
75	116	TKD2	p.G765C	NA	NA	NA
76	117	TKD2	p.G765D	44	M	3/Alive
76	118		p.G765D	37	F	5/Alive
76	119		p.G765D	NA	NA	NA
77	120	TKD2	p.G765S	33	M	1/Alive
78	121	TKD2	p.M766T	40	F	10
78	122		p.M766T	NA	F	NA
78	123		p.M766T	18	F	NA
78	124		p.M766T	18	F	12/Alive
78	125		p.M766T	46	M	3/Alive
79	126	TKD2	p.M766V	39	M	NA
80	127	TKD2	p.F768L	40	M	3/Alive
81	128	TKD2	p.A770P	52	M	11
81	129		p.A770P	NA	NA	NA
82	130	TKD2	p.C774_N814del	50	M	5
83	131	TKD2	p.C774_N814delinsQGLQSHVGPSLPSSSPQAQ	23	F	NA
84	132	TKD2	p.I775N	48	M	NA
85	133	TKD2	p.R777Q	60	F	5
85	134		p.R777Q	21	F	18M
85	135		p.R777Q	24	M	8/Alive
85	136		p.R777Q	38	F	7/Alive
85	137		p.R777Q	40	F	1M/Alive
85	138		p.R777Q	40	F	24/Alive
85	139		p.R777Q	41	F	3
85	140		p.R777Q	43	F	4/Alive
85	141		p.R777Q	33	F	1/Alive
85	142		p.R777Q	22	F	1/Alive
85	143		p.R777Q	33	F	1/Alive
86	144	TKD2	p.R777W	46	F	6
86	145		p.R777W	52	M	4/Alive
86	146		p.R777W	38	M	NA
86	147		p.R777W	40	M	NA
86	148		p.R777W	53	M	56
86	149		p.R777W	NA	NA	NA
86	150		p.R777W	NA	NA	NA
87	151	TKD2	p.D778E	60	F	6/Alive
88	152	TKD2	p.V779M	NA	NA	NA
89	153	TKD2	p.A781E	46	M	4

89	154	TKD2	p.A781E	36	F	3/Alive
90	155	TKD2	p.A781V	54	M	6
90	156		p.A781V	50	M	2/Alive
90	157		p.A781V	44	M	5
90	158		p.A781V	41	F	NA
90	159		p.A781V	32	F	2/Alive
90	160		p.A781V	36	F	2
90	161		p.A781V	30	M	3
90	162		p.A781V	NA	F	NA
90	163		p.A781V	43	F	2/Alive
90	164		p.A781V	38	M	3/Alive
90	165		p.A781V	45	M	2/Alive
90	166		p.A781V	34	F	4/Alive
91	167	TKD2	p.A781V_N783 del	NA	NA	NA
92	168	TKD2	p.I782T	28	F	1/Alive
93	169	TKD2	p.R782C	45	M	2/Alive
93	170		p.R782C	50	M	4
93	171		p.R782C	NA	NA	NA
94	172	TKD2	p.R782G	43	F	NA
94	173		p.R782G	33	F	NA
94	174		p.R782G	NA	F	1
94	175		p.R782G	38	M	2
94	176		p.R782G	37	M	1/Alive
95	177	TKD2	p.R782H	61	M	NA
95	178		p.R782H	44	M	NA
95	179		p.R782H	37	F	5
95	180		p.R782H	51	F	5/Alive
95	181		p.R782H	46	M	NA
95	182		p.R782H	51	F	2/Alive
95	183		p.R782H	NA	F	NA
95	184		p.R782H	61	F	2/Alive
95	185		p.R782H	51	F	NA
95	186		p.R782H	30	F	3M/Alive
95	187		p.R782H	46	F	2/Alive
96	188	TKD2	p.R782L	50	F	1
97	189	TKD2	p.R782W	51	F	1
98	190	TKD2	p.N783D	43	F	1/Alive
99	191	TKD2	p.V784M	39	F	6/Alive
100	192	TKD2	p.A792D	41	M	2/Alive
100	193		p.A792D	35	M	2/Alive
100	194		p.A792D	NA	NA	NA
100	195		p.A792D	41	M	5/Alive
101	196	TKD2	p.K793E	29	F	0.5/Alive
101	197		p.K793E	32	M	2/Alive
102	198	TKD2	p.K793T	40	M	1/Alive
102	199		p.K793T	41	M	1
102	200		p.K793T	41	M	6M/Alive
102	201		p.K793T	NA	NA	NA
103	202	TKD2	p.I794F	56	M	1/Alive
104	203	TKD2	p.I794N	29	F	0.5/Alive
105	204	TKD2	p.G795V	45	F	2/Alive
106	205	TKD2	p.F797C	40	M	1/Alive

107	206	TKD2	p.F797I	31	F	1/Alive
108	207	TKD2	p.F797L	51	F	1/Alive
109	208	TKD2	p.G798A	NA	NA	NA
110	209	TKD2	p.Y809S	37	M	1/Alive
111	210	TKD2	p.L817P	21	M	15
112	211	TKD2	p.L817Q	43	F	9/Alive
113	212	TKD2	p.K820N	45	F	1/Alive
114	213	TKD2	p.W821*	56	M	5/Alive
115	214	TKD2	p.W821C	35	F	15M/Alive
116	215	TKD2	p.W821R	32	F	NA
117	216	TKD2	p.M822I	44	F	1/Alive
118	217	TKD2	p.A823T	46	F	1/Alive
119	218	TKD2	p.A823D	NA	M	NA
119	219	TKD2	p.A823D	47	M	2
120	220	TKD2	p.A823V	50	F	NA
120	221	TKD2	p.A823V	46	M	NA
120	222	TKD2	p.A823V	51	F	18M/Alive
121	223	TKD2	p.P824S	45	F	1/Alive
122	224	TKD2	p.P824R	NA	NA	NA
123	225	TKD2	p.E825K	42	F	NA
123	226	TKD2	p.E825K	NA	NA	NA
123	227	TKD2	p.E825K	NA	NA	NA
124	228	TKD2	p.E825Q	46	F	2/Alive
125	229	TKD2	p.I827N	36	M	2/Alive
126	230	TKD2	p.I827T	42	F	2/Alive
127	231	TKD2	p.F828S	39	F	NA
127	232	TKD2	p.F828S	47	F	NA
128	233	TKD2	p.D829fs	48	M	1/Alive
129	234	TKD2	p.T833K	30	F	NA
130	235	TKD2	p.T833M	2	F	10
130	236	TKD2	p.T833M	1M	M	9
131	237	TKD2	p.T833R	41	M	2/Alive
131	238	TKD2	p.T833R	51	F	2/Alive
132	239	TKD2	p.D837H	47	M	18M/Alive
133	240	TKD2	p.D837Y	43	F	2/Alive
134	241	TKD2	p.V838L	51	M	4/Alive
134	242	TKD2	p.V838L	53	M	2/Alive
134	243	TKD2	p.V838L	54	M	2/Alive
134	244	TKD2	p.V838L	NA	M	NA
134	245	TKD2	p.V838L	NA	M	NA
135	246	TKD2	p.W839C	46	M	3/Alive
136	247	TKD2	p.Y841C	47	F	NA
137	248	TKD2	p.Y841H	NA	NA	NA
138	249	TKD2	p.Y841S	53	M	3/Alive
139	250	TKD2	p.G842V	60	M	NA
140	251	TKD2	p.I843_L844delinsGI	42	F	1/Alive
141	252	TKD2	p.I843F	55	M	4/Alive
142	253	TKD2	p.I843N	40	F	3/Alive
143	254	TKD2	p.I843T	42	F	2/Alive
144	255	TKD2	p.L845P	29	F	2/Alive
144	256	TKD2	p.L845P	36	M	NA
145	257	TKD2	p.W846L	41	M	2/Alive

146	258	TKD2	p.E847D	44	F	5/Alive
146	259		p.E847D	32	F	NA
146	260		p.E847D	NA	NA	NA
147	261	TKD2	p.E847K	34	M	1/Alive
147	262		p.E847K	49	F	12
148	263	TKD2	p.E847V	33	F	2/Alive
149	264	TKD2	p.F849del	44	F	NA
149	265		p.F849del	63	F	4
149	266		p.F849del	24	F	16M/Alive
150	267	TKD2	p.F849I	42	M	1/Alive
151	268	TKD2	p.F849S	46	F	5
151	269		p.F849S	58	F	1/Alive
152	270	TKD2	p.S850L	47	F	1/Alive
153	271	TKD2	p.L851P	NA	F	NA
153	272		p.L851P	28	F	7/Alive
153	273		p.L851P	27	F	8/Alive
153	274		p.L851P	24	F	3
154	275	TKD2	p.L851R	56	M	5
155	276	TKD2	p.G852Dfs	43	F	7/Alive
156	277	TKD2	p.N854K	44	F	5/Alive
156	278		p.N854K	29	F	NA
157	279	TKD2	p.P855T	41	F	8
157	280		p.P855T	34	M	NA
157	281		p.P855T	50	F	1
157	282		p.P855T	30	F	NA
158	283	TKD2	p.Y856H	39	F	3
158	284		p.Y856H	42	F	NA
159	285	TKD2	p.Y856S	37	F	2/Alive
160	286	TKD2	p.P857L	38	F	NA
161	287	TKD2	p.L868P	60	M	NA
161	288		p.L868P	55	F	8
162	289	TKD2	p.L868R	64	M	11
163	290	TKD2	p.V869G	45	M	NA
163	291		p.V869G	39	F	1/Alive
164	292	TKD2	p.M875I	40	F	43
165	293	TKD2	p.M875R	NA	NA	NA
166	294	TKD2	p.M875T	NA	F	NA
166	295		p.M875T	58	M	8
167	296	TKD2	p.A876Vfs	55	F	1
168	297	TKD2	p.Q877*	28	M	2/Alive
169	298	TKD2	p.P878H	53	F	5/Alive
170	299	TKD2	p.P878A	57	M	5/Alive
170	300		p.P878A	60	M	4/Alive
171	301	TKD2	p.P878S	45	M	10/Alive
171	302		p.P878S	45	F	NA
172	303	TKD2	p.P878T	39	F	10
173	304	TKD2	p.A881fs	73	M	1/Alive
174	305	TKD2	p.A881V	44	M	NA
175	306	TKD2	p.P882Pfs*70	24	M	1/Alive
176	307	TKD2	p.K883del	42	F	1/Alive
177	308	TKD2	c.2652T>G	40	F	1/Alive
178	309	TKD2	p.Y886Qfs*55	46	M	1/Alive
179	310	TKD2	c.889+1G>C	26	M	1/Alive

180	311	TKD2	p.C892_A894del	52	M	4/Alive
180	312		p.C892_A894del	52	M	7/Alive
181	313	TKD2	p.894_897del	38	M	1/Alive
182	314	TKD2	p.895_897del	37	M	2/Alive
183	315	TKD2	p.H899fs	42	M	1/Alive
184	316	TKD2	p.R900K	60	NA	NA
184	317		p.R900K	51	M	2/Alive
184	318		p.R900K	52	F	1/Alive
185	319	TKD2	p.R900T	45	F	2/Alive
186	320	TKD2	p.P901S	20	NA	25
186	321		p.P901S	39	F	2/Alive
187	322	TKD2	p.T902Sfs	49	F	2/Alive
188	323	TKD2	p.I906T	36	M	1/Alive
189	324	TKD2	p.L910P	36	F	1
190	325	TKD2	p.A914T	NA	NA	NA
191	326	TKD2	p.E916K	NA	NA	NA
192	327	TKD2	p.E920D	NA	NA	NA
193	328	TKD2	p.G936S	29	M	1/Alive
194	329	TKD2	p.G957R	49	M	8
195	330	Others	p.F971Sfs*7	46	M	NA
196	331	Others	c.49 G>T	26	M	2/Alive
197	332	Others	c.1754-3C>G	43	F	0.75/Alive
198	333	Others	c.1754-1G>C	0	M	10M
199	334	Others	c.1754-10 T > A	42	M	2M/Alive
200	335	Others	c.1858+1G>T	37	M	7M/Alive
201	336	Others	c.1858+5G>A	41	F	3/Alive
201	337		c.1858+5G>A	44	F	2/Alive
202	338	Others	c.1969+115_1969+116del	23	M	5
203	339	Others	c.2133_2919del heterozygous	37	F	3/Alive
204	340	Others	c.2319+1C>A	40	M	3
204	341		c.2319+1C>A	54	M	4
205	342	Others	c.2320-2A>G	50	M	5
206	343	Others	c.2442+1G>A	43	NA	NA
206	344		c.2442+1G>A	20	F	2/Alive
206	345		c.2442+1G>A	22.9	F	1.75/Alive
206	346		c.2442+1G>A	38.5	F	1.01/Alive
206	347	Others	c.2442+1G>T	47	F	NA
206	348		c.2442+1G>T	53	M	4
206	349		c.2442+1G>T	NA	NA	NA
207	350	Others	c.2442+2T>C	39	M	5
208	351	Others	c.2442+4A>G	38	F	2/Alive
209	352	Others	c.2442+5G>A	27	F	1/Alive
209	353		c.2442+5G>A	58	M	3/Alive
209	354		c.2442+5G>A	60	F	1/Alive
209	355		c.2442+5G>A	51	F	2/Alive
210	356	Others	c.2442+5G>C	23	F	1/Alive
211	357	Others	c.2442+2_2442+3dupT	41	F	NA
212	358	Others	c.2654+1G>A	39	M	4/Alive
213	359	Others	c.2563C＞A	45	M	18M/Alive

214	360	Others	c.2654_2654+3 del	43	F	7/Alive
215	361	Others	c.2655-1G>C	42	F	1/Alive
215	362		c.2655-1G>C	28	M	1/Alive
216	363	Others	c.2655-2A>G	50	F	2
216	364		c.2655-2A>G	42.5	F	1.5/Alive
217	365	Others	c.2763+1G>T (chr5:14943388 4C>A)	9	F	1/Alive
218	366	Others	c2909_2910ins AT^cAC	29	M	NA
218	367		c2909_2910ins AT^cAC	30	M	2/Alive
219	368	Others	delCTC	39	F	9M/Alive
220	369	Others	Deletion #1	43	F	6/Alive
221	370	Others	Deletion #2	37	M	4/Alive

EC: extracellular domain; TM: transmembrane domain; JMD: Juxtamembrane domain; TKD1/2: tyrosine kinase domain

Ref
2
40
41
42
42
43
15
28
44
42
6
11
6
16
45
41
41
41
41
41
41
28
42
46
15
6
42
47
43
44
43
41
48
49
50
37
51
52
49
2
53
42
11
46
54
30
43
55
30
56

57
57
58
59
59
60
57
61
43
15
42
19
19
62
62
63
42
47
2
64
64
42
44
44
37
65
37
30
46
63
52
66
36
4
67
42
40
42
5
47
57
62
68
44
42
28
66
69
42
4
5
5
17

50
50
50
30
52
70
71
72
73
62
19
63
19
28
17
62
47
55
74
30
57
66
75
64
30
62
30
30
30
37
76
77
34
78
79
43
47
42
42
42
37
80
16
81
81
19
19
28
19
4
17

82
34
75
75
83
4
5
54
42
42
42
42
84
18
85
43
19
58
58
78
78
15
59
59
75
73
36
74
86
87
73
88
47
89
42
15
90
91
92
19
20
42
47
93
81
93
19
28
42
64
42
42

42
19
11
37
69
15
42
94
2
95
15
4
5
58
59
96
17
84
63
19
19
42
97
37
29
29
42
98
99
100
11
47
101
30
102
34
46
54
103
64
33
19
42
33
104
68
68
47
5
33
11
37

105
19
106
107
108
59
30
6
15
30
1
42
4
109
42
42
42
7
110
111
112
40
40
40
37
33
42
36
55
30
50
113
15
114
19
30
115
42
34
66
28
66
28
59
30
6
64
70
15
116
28
117
24

118
11
42
119
120
121
122
6
37
42
42
68
42
43
50
50
42
50
59
12
42
67
123
124
125
126
127
64
128
42
40
63
129
116
116
130
17
19
131
42
57
57
24
47
30
2
126
6
7

42
47
37
42
132
2
12
6
15
15
6

1/2; AO: Age of

Table S4. Microglia replacement strategies in different figures. Related to Figures 5-7.

Figure	Age of mice at baseline	Microglia depletion	Transplanted microglia derivation	Brain collection
**5B**	One-month old	Two rounds of orally administrating PLX3397 for 7 days	Cultured GFP^+ microglia from newborn *Cx3cr1*$^{GFP/+}$ mice	Mouse brains were collected one-month after transplantation
**S4D**	One-month old	Orally administrating PLX3397 for seven days	Cultured GFP^+ microglia from newborn *Cx3cr1*$^{GFP/+}$ mice	Mouse brains were collected one-month after transplantation
**6B**	One-month old	Two rounds of orally administrating PLX3397 for 7 days	Cultured GFP^+ microglia from newborn *Cx3cr1*$^{GFP/+}$ mice	Mouse brains were collected seven-months after transplantation
**7B**	Nine or ten-month old	Two rounds of orally administrating PLX3397 for 7 days	Cultured GFP^+ microglia from newborn *Cx3cr1*$^{GFP/+}$ mice	Mouse brains were collected one-month after transplantation
**S7C**	Nine or ten-month old	Orally administrating PLX3397 for seven days	FACS-isolated GFP^+ microglia from newborn *Cx3cr1*$^{GFP/+}$ mice	Mouse brains were collected one-month after transplantation
**S8A**	Nine or ten-month old	0*Tp: Without PLX3397 administration	Cultured GFP^+ microglia from newborn *Cx3cr1*$^{GFP/+}$ mice	Mouse brains were collected one-month after transplantation
**S8D**	Nine or ten-month old	7*Tp: Orally administrating PLX3397 for seven days	Cultured GFP^+ microglia from newborn *Cx3cr1*$^{GFP/+}$ mice	Mouse brains were collected one-month after transplantation

Table S5. The primer sequences for qRT-PCR experiments. Related to STAR Methods. Related to Figure 4.

Name	GenBank	Forward primer (5’−3’)	Reversed primer (5’−3’)
Actb	NM_11461	TCTTGGGTATGGAATCCTGTGGCA	TCTTGGGTATGGAATCCTGTGGCA
Csf1r	NM_12978	GGTTGTAGAGCGGGTGAAA	AAGAGTGGGCCGATCTTTG
Csf3	NM_ 12985	TATAAAGGCCCCCTGGAGCTG	GCTGCAGGGCCATTAGCTTC
Csf2	NM_12981	GGCATTGTGGTCTACAGCCT	TGAAATTGCCCCGTAGACCC
Tmem119	NM_231633	GTCACTCCATCCCAGTTTCAC	CAGGGGACCATGTTGAGCTAT
P2ry12	NM_70839	CACTTTCCCGTATCCAGGGT	GCCTTGAGTGTTTCTGTAGGGTA
Axl	NM_26362	TCCTCCAAGGGATGGGTCAT	CTCACAGTGGGGAAACCGAA
Cst7	NM_13011	TGCTTACCAGCGAGCAGATT	GCTTCCCACACTACCACCTT
H2-Ab1	NM_14961	GCTTGAACAGCCCAATGTCG	CGCACTTTGATCTTGGCTGG
Lpl	NM_16956	AGGCATACAGGTGCAACTCC	TAGGGCATCTGAGAGCGAGT

Supplemental References

1. Lan, M.Y., Liu, J.S., Chang, C.C., Chen, Y.F., Su, C.S., Peng, C.H., and Chang, Y.Y. (2016). Clinicopathologic and Genetic Studies of 2 Patients With Hereditary Diffuse Leukoencephalopathy With Axonal Spheroids. Alzheimer Dis Assoc Disord *30*, 73-76. 10.1097/WAD.0000000000000067.
2. Chu, M., Wang, D.X., Cui, Y., Kong, Y., Liu, L., Xie, K.X., Xia, T.X., Zhang, J., Gao, R., Zhou, A.H., et al. (2021). Three novel mutations in Chinese patients with CSF1R-related leukoencephalopathy. Ann Transl Med *9*, 1072. 10.21037/atm-21-217.
3. Bai, Y., Lu, L., Cui, Y., Li, J., Liu, Y., Liu, L., Dong, J., Wang, Q., Qin, W., Wu, L., and Jia, J. (2018). Analysis of clinical and neuroimaging features in a Chinese family with hereditary diffuse leukoencephalopathy with neuroaxonal spheroids. Chinese journal of neurology *51*, 877-881.
4. Tian, W.T., Zhan, F.X., Liu, Q., Luan, X.H., Zhang, C., Shang, L., Zhang, B.Y., Pan, S.J., Miao, F., Hu, J., et al. (2019). Clinicopathologic characterization and abnormal autophagy of CSF1R-related leukoencephalopathy. Transl Neurodegener *8*, 32. 10.1186/s40035-019-0171-y.
5. Zhan, F.X., Zhu, Z.Y., Liu, Q., Zhou, H.Y., Luan, X.H., Huang, X.J., Liu, X.L., Tian, W.T., Wang, S.G., Song, X.X., et al. (2021). Altered structural and functional connectivity in CSF1R-related leukoencephalopathy. Brain Imaging Behav *15*, 1655-1666. 10.1007/s11682-020-00360-0.
6. Mao, C., Zhou, L., Zhou, L., Yang, Y., Niu, J., Li, J., Huang, X., Ren, H., Zhao, Y., Peng, B., and Gao, J. (2020). Biopsy histopathology in the diagnosis of adult-onset leukoencephalopathy with axonal spheroids and pigmented glia (ALSP). Neurol Sci *41*, 403-409. 10.1007/s10072-019-04116-7.
7. Xie, J.J., Ni, W., Wei, Q., Ma, H., Bai, G., Shen, Y., and Wu, Z.Y. (2020). New clinical characteristics and novel pathogenic variants of patients with hereditary leukodystrophies. CNS Neurosci Ther *26*, 567-575. 10.1111/cns.13284.
8. Liu, Q., Guo, X.N., Liu, C.Y., and Xu, W.H. (2020). A proposed synergistic effect of CSF1R and NMUR2 variants contributes to binge eating in hereditary diffuse leukoencephalopathy with spheroids. Ann Transl Med *8*, 7. 10.21037/atm.2019.11.30.
9. 王康, 彭国平, and 罗本燕 (2015). CSF1R 基因突变所致的伴球状体遗传性弥漫性白质脑病一例. held in 中国浙江舟山, pp. 2.
10. Wu, J., Tian, W., Zhan, F., Luan, X., and Cao, L. (2021). Generation of an human induced pluripotent stem cell JTUi007-A from a patient with CSF1R-related leukoencephalopathy carrying heterozygous p.Ile794Thr mutation in CSF1R gene. Stem Cell Res *57*, 102593. 10.1016/j.scr.2021.102593.
11. Wu, J., Cao, Y., Li, M., Li, B., Jia, X., and Cao, L. (2022). Altered intrinsic brain activity in patients with CSF1R-related leukoencephalopathy. Brain Imaging Behav *16*, 1842-1853. 10.1007/s11682-022-00646-5.
12. Wu, L., Liu, J., Sha, L., Wang, X., Li, J., Dong, J., and Jia, J. (2017). Sporadic Cases with Novel Mutations and Pedigree in Hereditary Leukoencephalopathy with Axonal Spheroids. J Alzheimers Dis *56*, 893-898. 10.3233/JAD-161193.
13. Wu, D., Zhao, J., and Zheng, L. (2024). Adult-onset leukoencephalopathy with persistent diffusion restriction dot lesions. Neurological Sciences *45*, 1797-1798. 10.1007/s10072-023-07212-x.

14. Komatsu, T., Takahashi, M., Omoto, S., and Iguchi, Y. (2023). Asymmetric focal cortical atrophy in CSF1R-related leukoencephalopathy; case report. Acta Neurol Belg *123*, 2001-2003. 10.1007/s13760-022-02065-1.

15. Ishiguro, T., Konno, T., Hara, N., Zhu, B., Okada, S., Shibata, M., Saika, R., Kitano, T., Toko, M., Nezu, T., et al. (2023). Novel partial deletions, frameshift and missense mutations of CSF1R in patents with CSF1R-related leukoencephalopathy. Eur J Neurol *30*, 1861-1870. 10.1111/ene.15796.

16. Mitsui, J., Matsukawa, T., Ishiura, H., Higasa, K., Yoshimura, J., Saito, T.L., Ahsan, B., Takahashi, Y., Goto, J., Iwata, A., et al. (2012). CSF1R mutations identified in three families with autosomal dominantly inherited leukoencephalopathy. Am J Med Genet B Neuropsychiatr Genet *159B*, 951-957. 10.1002/ajmg.b.32100.

17. Konno, T., Tada, M., Tada, M., Koyama, A., Nozaki, H., Harigaya, Y., Nishimiya, J., Matsunaga, A., Yoshikura, N., Ishihara, K., et al. (2014). Haploinsufficiency of CSF-1R and clinicopathologic characterization in patients with HDLS. Neurology *82*, 139-148. 10.1212/WNL.0000000000000046.

18. Saitoh, B.Y., Yamasaki, R., Hayashi, S., Yoshimura, S., Tateishi, T., Ohyagi, Y., Murai, H., Iwaki, T., Yoshida, K., and Kira, J. (2013). A case of hereditary diffuse leukoencephalopathy with axonal spheroids caused by a de novo mutation in CSF1R masquerading as primary progressive multiple sclerosis. Mult Scler *19*, 1367-1370. 10.1177/1352458513489854.

19. Kondo, Y., Matsushima, A., Nagasaki, S., Nakamura, K., Sekijima, Y., and Yoshida, K. (2020). Factors predictive of the presence of a CSF1R mutation in patients with leukoencephalopathy. Eur J Neurol *27*, 369-375. 10.1111/ene.14086.

20. Hamatani, M., Yamashita, H., Ochi, H., Ashida, S., Hashi, Y., Okada, Y., Fujii, C., Kawamura, K., Kitazawa, R., Nakagawa, M., et al. (2020). Altered features of monocytes in adult onset leukoencephalopathy with axonal spheroids and pigmented glia: A clue to the pathomechanism of microglial dyshomeostasis. Neurobiol Dis *140*, 104867. 10.1016/j.nbd.2020.104867.

21. Kitani-Morii, F., Kasai, T., Tomonaga, K., Saito, K., Mizuta, I., Yoshioka, A., Nakagawa, M., and Mizuno, T. (2014). Hereditary diffuse leukoencephalopathy with spheroids characterized by spastic hemiplegia preceding mental impairment. Intern Med *53*, 1377-1380. 10.2169/internalmedicine.53.1932.

22. 田中賢, 田中晋, 田中弘, 五十嵐勝, and 池内健 (2018). 上顎洞内に骨破壊と異所性石灰化像を認めた神経軸索スフェロイドを伴う遺伝性白質脳症 (HDLS) の 1 症例.

23. 池内, 健. (2014). 神経軸索スフェロイド形成をともなう遺伝性びまん性白質脳症（HDLS）の臨床像と画像所見. 臨床神経学 *54*, 1158-1161. 10.5692/clinicalneurol.54.1158.

24. Kim, M., Lee, H., Cho, H.J., Young Chun, S., Shin, J.H., Kim, E.J., Woo Ahn, J., Huh, G.Y., Baek, S.Y., and Lee, J.H. (2017). Pathologic Correlation of Paramagnetic White Matter Lesions in Adult-Onset Leukoencephalopathy With Axonal Spheroids and Pigmented Glia. J Neuropathol Exp Neurol *76*, 924-928. 10.1093/jnen/nlx086.

25. Kim, S.J., Cho, W., Kim, H.J., Na, D.L., Seo, S.W., Jung, N.Y., Lee, J.H., Lee, M.J., Kang, H., Seong, J.K., and Kim, E.J. (2024). Distinct patterns of white matter hyperintensity and cortical thickness of CSF1R-related leukoencephalopathy compared with subcortical ischemic vascular dementia. PLoS One *19*, e0308989. 10.1371/journal.pone.0308989.

26. Reddy Tallapalli, A.V., Nashi, S., Kamath, S.D., Srijithesh, P.R., Kulkarni, G.B., and Alladi, S.

(2022). A Rare Genetic Cause of Young Onset Rapidly Progressive Dementia- First Report from India. Neurol India *70*, 781-783. 10.4103/0028-3886.344666.

27. Rudrabhatla, P., Sabarish, S., Ramachandran, H., and Nair, S.S. (2021). Teaching NeuroImages: Rare Adult-Onset Genetic Leukoencephalopathy. Neurology *96*, e2561-e2562. 10.1212/WNL.0000000000011233.
28. Miura, T., Mezaki, N., Konno, T., Iwasaki, A., Hara, N., Miura, M., Funayama, M., Unai, Y., Tashiro, Y., Okita, K., et al. (2018). Identification and functional characterization of novel mutations including frameshift mutation in exon 4 of CSF1R in patients with adult-onset leukoencephalopathy with axonal spheroids and pigmented glia. J Neurol *265*, 2415-2424. 10.1007/s00415-018-9017-2.
29. Kleinfeld, K., Mobley, B., Hedera, P., Wegner, A., Sriram, S., and Pawate, S. (2013). Adult-onset leukoencephalopathy with neuroaxonal spheroids and pigmented glia: report of five cases and a new mutation. J Neurol *260*, 558-571. 10.1007/s00415-012-6680-6.
30. Rademakers, R., Baker, M., Nicholson, A.M., Rutherford, N.J., Finch, N., Soto-Ortolaza, A., Lash, J., Wider, C., Wojtas, A., DeJesus-Hernandez, M., et al. (2011). Mutations in the colony stimulating factor 1 receptor (CSF1R) gene cause hereditary diffuse leukoencephalopathy with spheroids. Nat Genet *44*, 200-205. 10.1038/ng.1027.
31. Van Gerpen, J.A., Wider, C., Broderick, D.F., Dickson, D.W., Brown, L.A., and Wszolek, Z.K. (2008). Insights into the dynamics of hereditary diffuse leukoencephalopathy with axonal spheroids. Neurology *71*, 925-929. 10.1212/01.wnl.0000325916.30701.21.
32. Sharma, R., Graff-Radford, J., Rademakers, R., Boeve, B.F., Petersen, R.C., and Jones, D.T. (2019). CSF1R mutation presenting as dementia with Lewy bodies. Neurocase *25*, 17-20. 10.1080/13554794.2019.1601230.
33. Codjia, P., Ayrignac, X., Mochel, F., Mouzat, K., Carra-Dalliere, C., Castelnovo, G., Ellie, E., Etcharry-Bouyx, F., Verny, C., Belliard, S., et al. (2018). Adult-Onset Leukoencephalopathy with Axonal Spheroids and Pigmented Glia: An MRI Study of 16 French Cases. AJNR Am J Neuroradiol *39*, 1657-1661. 10.3174/ajnr.A5744.
34. Karle, K.N., Biskup, S., Schule, R., Schweitzer, K.J., Kruger, R., Bauer, P., Bender, B., Nagele, T., and Schols, L. (2013). De novo mutations in hereditary diffuse leukoencephalopathy with axonal spheroids (HDLS). Neurology *81*, 2039-2044. 10.1212/01.wnl.0000436945.01023.ac.
35. Meyer-Ohlendorf, M., Braczynski, A., Al-Qaisi, O., Gessler, F., Biskup, S., Weise, L., Steinbach, J.P., Wagner, M., Mittelbronn, M., and Bahr, O. (2015). Comprehensive diagnostics in a case of hereditary diffuse leukodystrophy with spheroids. BMC Neurol *15*, 103. 10.1186/s12883-015-0368-3.
36. Lynch, D.S., Rodrigues Brandao de Paiva, A., Zhang, W.J., Bugiardini, E., Freua, F., Tavares Lucato, L., Macedo-Souza, L.I., Lakshmanan, R., Kinsella, J.A., Merwick, A., et al. (2017). Clinical and genetic characterization of leukoencephalopathies in adults. Brain *140*, 1204-1211. 10.1093/brain/awx045.
37. Guerreiro, R., Kara, E., Le Ber, I., Bras, J., Rohrer, J.D., Taipa, R., Lashley, T., Dupuits, C., Gurunlian, N., Mochel, F., et al. (2013). Genetic analysis of inherited leukodystrophies: genotype-phenotype correlations in the CSF1R gene. JAMA Neurol *70*, 875-882. 10.1001/jamaneurol.2013.698.
38. Molloy, A., Williams, L., Farrell, M., and O'Riordan, S. (2014). Hereditary Diffuse Leucoencephalopathy and Spheroids Resulting From a Mutation in CSF1R: A Rare Cause of

Parkinsonism. Mov Disord Clin Pract *1*, 132-133. 10.1002/mdc3.12033.

39. Konno, T., Yoshida, K., Mizuta, I., Mizuno, T., Kawarai, T., Tada, M., Nozaki, H., Ikeda, S.I., Onodera, O., Wszolek, Z.K., and Ikeuchi, T. (2018). Diagnostic criteria for adult-onset leukoencephalopathy with axonal spheroids and pigmented glia due to CSF1R mutation. Eur J Neurol *25*, 142-147. 10.1111/ene.13464.

40. Zhuang, L.P., Liu, C.Y., Li, Y.X., Huang, H.P., and Zou, Z.Y. (2020). Clinical features and genetic characteristics of hereditary diffuse leukoencephalopathy with spheroids due to CSF1R mutation: a case report and literature review. Ann Transl Med *8*, 11. 10.21037/atm.2019.12.17.

41. Giau, V.V., Senanarong, V., Bagyinszky, E., An, S.S.A., and Kim, S. (2019). Analysis of 50 Neurodegenerative Genes in Clinically Diagnosed Early-Onset Alzheimer's Disease. Int J Mol Sci *20*. 10.3390/ijms20061514.

42. Wu, J., Cheng, X., Ji, D., Niu, H., Yao, S., Lv, X., Wang, J., Li, Z., Zheng, H., Cao, Y., et al. (2024). The Phenotypic and Genotypic Spectrum of CSF1R-Related Disorder in China. Mov Disord *39*, 798-813. 10.1002/mds.29764.

43. Tsai, P.C., Fuh, J.L., Yang, C.C., Chang, A., Lien, L.M., Wang, P.N., Lai, K.L., Tsai, Y.S., Lee, Y.C., and Liao, Y.C. (2021). Clinical and genetic characterization of adult-onset leukoencephalopathy caused by CSF1R mutations. Ann Clin Transl Neurol *8*, 2121-2131. 10.1002/acn3.51467.

44. Guo, L., Bertola, D.R., Takanohashi, A., Saito, A., Segawa, Y., Yokota, T., Ishibashi, S., Nishida, Y., Yamamoto, G.L., Franco, J., et al. (2019). Bi-allelic CSF1R Mutations Cause Skeletal Dysplasia of Dysosteosclerosis-Pyle Disease Spectrum and Degenerative Encephalopathy with Brain Malformation. Am J Hum Genet *104*, 925-935. 10.1016/j.ajhg.2019.03.004.

45. Riku, Y., Ando, T., Goto, Y., Mano, K., Iwasaki, Y., Sobue, G., and Yoshida, M. (2014). Early pathologic changes in hereditary diffuse leukoencephalopathy with spheroids. J Neuropathol Exp Neurol *73*, 1183-1190. 10.1097/NEN.0000000000000139.

46. Schuberth, M., Levin, J., Sawalhe, D., Schwarzkopf, R., von Baumgarten, L., Ertl-Wagner, B., Rominger, A., Arzberger, T., Kretzschmar, H.A., Frobose, T., et al. (2014). [Hereditary diffuse leukencephalopathy with spheroids: a microgliopathy due to CSF1 receptor impairment]. Nervenarzt *85*, 465-470. 10.1007/s00115-014-4052-4.

47. Hu, B., Zhou, Y., Wu, C., Xiao, N., Li, J., Li, X., Li, Y., Zhang, X., Huang, X., Song, Y., et al. (2025). Evaluation of Soluble Colony Stimulating Factor 1 Receptor (CSF1R) in Peripheral Blood as a Diagnostic Marker of CSF1R-Related Disorder (CSF1R-RD) in a Murine Model and CSF1R-RD Patients. Ann Neurol *97*, 397-403. 10.1002/ana.27147.

48. Monies, D., Maddirevula, S., Kurdi, W., Alanazy, M.H., Alkhalidi, H., Al-Owain, M., Sulaiman, R.A., Faqeih, E., Goljan, E., Ibrahim, N., et al. (2017). Autozygosity reveals recessive mutations and novel mechanisms in dominant genes: implications in variant interpretation. Genet Med *19*, 1144-1150. 10.1038/gim.2017.22.

49. Sassi, C., Capozzo, R., Hammer, M., Zecca, C., Federoff, M., Blauwendraat, C., Bernstein, N., Ding, J., Gibbs, J.R., Price, T., et al. (2021). Exploring dementia and neuronal ceroid lipofuscinosis genes in 100 FTD-like patients from 6 towns and rural villages on the Adriatic Sea cost of Apulia. Sci Rep *11*, 6353. 10.1038/s41598-021-85494-x.

50. Sassi, C., Nalls, M.A., Ridge, P.G., Gibbs, J.R., Lupton, M.K., Troakes, C., Lunnon, K., Al-Sarraj, S., Brown, K.S., Medway, C., et al. (2018). Mendelian adult-onset leukodystrophy genes in Alzheimer's disease: critical influence of CSF1R and NOTCH3. Neurobiol Aging *66*, 179

e117-179 e129. 10.1016/j.neurobiolaging.2018.01.015.
51. Chen, Z., Tan, Y.J., Lian, M.M., Tandiono, M., Foo, J.N., Lim, W.K., Kandiah, N., Tan, E.K., and Ng, A.S.L. (2021). High Diagnostic Utility Incorporating a Targeted Neurodegeneration Gene Panel With MRI Brain Diagnostic Algorithms in Patients With Young-Onset Cognitive Impairment With Leukodystrophy. Front Neurol *12*, 631407. 10.3389/fneur.2021.631407.
52. Konno, T., Miura, T., Harriott, A.M., Mezaki, N., Edwards, E.S., Rademakers, R., Ross, O.A., Meschia, J.F., Ikeuchi, T., and Wszolek, Z.K. (2018). Partial loss of function of colony-stimulating factor 1 receptor in a patient with white matter abnormalities. Eur J Neurol *25*, 875-881. 10.1111/ene.13611.
53. Ghadiri, M., Buckland, M.E., Sutton, I.J., Al Jahdhami, S., Flanagan, S., Heard, R., Barnett, Y., Brennan, J., and Barnett, M.H. (2014). Progressive neuropsychiatric symptoms and motor impairment. JAMA Neurol *71*, 794-798. 10.1001/jamaneurol.2013.6308.
54. Hofer, T.P., Zawada, A.M., Frankenberger, M., Skokann, K., Satzl, A.A., Gesierich, W., Schuberth, M., Levin, J., Danek, A., Rotter, B., et al. (2015). slan-defined subsets of CD16-positive monocytes: impact of granulomatous inflammation and M-CSF receptor mutation. Blood *126*, 2601-2610. 10.1182/blood-2015-06-651331.
55. Blauwendraat, C., Pletnikova, O., Geiger, J.T., Murphy, N.A., Abramzon, Y., Rudow, G., Mamais, A., Sabir, M.S., Crain, B., Ahmed, S., et al. (2019). Genetic analysis of neurodegenerative diseases in a pathology cohort. Neurobiol Aging *76*, 214 e211-214 e219. 10.1016/j.neurobiolaging.2018.11.007.
56. Fujioka, S., Broderick, D.F., Sundal, C., Baker, M.C., Rademakers, R., and Wszolek, Z.K. (2013). An adult-onset leukoencephalopathy with axonal spheroids and pigmented glia accompanied by brain calcifications: a case report and a literature review of brain calcifications disorders. J Neurol *260*, 2665-2668. 10.1007/s00415-013-7093-x.
57. Konno, T., Broderick, D.F., Mezaki, N., Isami, A., Kaneda, D., Tashiro, Y., Tokutake, T., Keegan, B.M., Woodruff, B.K., Miura, T., et al. (2017). Diagnostic Value of Brain Calcifications in Adult-Onset Leukoencephalopathy with Axonal Spheroids and Pigmented Glia. AJNR Am J Neuroradiol *38*, 77-83. 10.3174/ajnr.A4938.
58. Abe, T., Kawarai, T., Fujita, K., Sako, W., Terasawa, Y., Matsuda, T., Sakai, W., Tsukamoto-Miyashiro, A., Matsui, N., Izumi, Y., et al. (2017). MR Spectroscopy in Patients with Hereditary Diffuse Leukoencephalopathy with Spheroids and Asymptomatic Carriers of Colony-stimulating Factor 1 Receptor Mutation. Magn Reson Med Sci *16*, 297-303. 10.2463/mrms.mp.2016-0016.
59. Kim, J.R., Lee, S., Seo, S.W., Jang, J.H., Suh, Y.L., Park, J.H., Lee, S.Y., Son, H.J., Kwon, H.J., Kim, E.J., et al. (2025). Clinical spectrum of adult-onset leukoencephalopathy with axonal spheroids and pigmented glia in individuals of Korean ancestry. Sci Rep *15*, 1857. 10.1038/s41598-024-84665-w.
60. Ho, V.M., Hovsepian, D.A., and Shieh, P.B. (2019). Myelopathy in a patient with leukodystrophy due to CSF1R mutation. Neurol Genet *5*, e376. 10.1212/NXG.0000000000000376.
61. Daida, K., Nishioka, K., Li, Y., Nakajima, S., Tanaka, R., and Hattori, N. (2017). CSF1R Mutation p.G589R and the Distribution Pattern of Brain Calcification. Intern Med *56*, 2507-2512. 10.2169/internalmedicine.8462-16.
62. Konno, T., Yoshida, K., Mizuno, T., Kawarai, T., Tada, M., Nozaki, H., Ikeda, S.I., Nishizawa,

M., Onodera, O., Wszolek, Z.K., and Ikeuchi, T. (2017). Clinical and genetic characterization of adult-onset leukoencephalopathy with axonal spheroids and pigmented glia associated with CSF1R mutation. Eur J Neurol *24*, 37-45. 10.1111/ene.13125.

63. Lynch, D.S., Jaunmuktane, Z., Sheerin, U.M., Phadke, R., Brandner, S., Milonas, I., Dean, A., Bajaj, N., McNicholas, N., Costello, D., et al. (2016). Hereditary leukoencephalopathy with axonal spheroids: a spectrum of phenotypes from CNS vasculitis to parkinsonism in an adult onset leukodystrophy series. J Neurol Neurosurg Psychiatry *87*, 512-519. 10.1136/jnnp-2015-310788.

64. Schmitz, A.S., Raju, J., Kohler, W., Klebe, S., Cheheb, K., Reschke, F., Biskup, S., Haack, T.B., Roeben, B., Kellner, M., et al. (2024). Novel variants in CSF1R associated with adult-onset leukoencephalopathy with axonal spheroids and pigmented glia (ALSP). J Neurol *271*, 6025-6037. 10.1007/s00415-024-12557-0.

65. Bayat, M., Shekhrajka, N., and Bayat, A. (2019). Hereditary leukodystrophy with axonal spheroids (HDLS) presenting subacutely: a CNS-vasculitis mimic. Acta Neurol Belg *119*, 633-635. 10.1007/s13760-019-01096-5.

66. Chitu, V., Biundo, F., Shlager, G.G.L., Park, E.S., Wang, P., Gulinello, M.E., Gokhan, S., Ketchum, H.C., Saha, K., DeTure, M.A., et al. (2020). Microglial Homeostasis Requires Balanced CSF-1/CSF-2 Receptor Signaling. Cell Rep *30*, 3004-3019 e3005. 10.1016/j.celrep.2020.02.028.

67. Oosterhof, N., Chang, I.J., Karimiani, E.G., Kuil, L.E., Jensen, D.M., Daza, R., Young, E., Astle, L., van der Linde, H.C., Shivaram, G.M., et al. (2019). Homozygous Mutations in CSF1R Cause a Pediatric-Onset Leukoencephalopathy and Can Result in Congenital Absence of Microglia. Am J Hum Genet *104*, 936-947. 10.1016/j.ajhg.2019.03.010.

68. Battisti, C., Di Donato, I., Bianchi, S., Monti, L., Formichi, P., Rufa, A., Taglia, I., Cerase, A., Dotti, M.T., and Federico, A. (2014). Hereditary diffuse leukoencephalopathy with axonal spheroids: three patients with stroke-like presentation carrying new mutations in the CSF1R gene. J Neurol *261*, 768-772. 10.1007/s00415-014-7257-3.

69. Gelfand, J.M., Greenfield, A.L., Barkovich, M., Mendelsohn, B.A., Van Haren, K., Hess, C.P., and Mannis, G.N. (2020). Allogeneic HSCT for adult-onset leukoencephalopathy with spheroids and pigmented glia. Brain *143*, 503-511. 10.1093/brain/awz390.

70. Du, Q., Chen, H., Shi, Z., Zhang, Y., Wang, J., and Zhou, H. (2019). A novel mutation in the CSF1R gene causes hereditary diffuse leukoencephalopathy with axonal spheroids. Neurol Sci *40*, 1287-1290. 10.1007/s10072-018-3693-7.

71. Huang, H., Cao, L., and Chen, H. (2021). Dynamic analysis of CSF1R-related leukoencephalopathy on magnetic resonance imaging: a case report. BMC Neurol *21*, 156. 10.1186/s12883-021-02182-z.

72. Konno, T., Kasanuki, K., Ikeuchi, T., Dickson, D.W., and Wszolek, Z.K. (2018). CSF1R-related leukoencephalopathy: A major player in primary microgliopathies. Neurology *91*, 1092-1104. 10.1212/WNL.0000000000006642.

73. Kinoshita, M., Yoshida, K., Oyanagi, K., Hashimoto, T., and Ikeda, S. (2012). Hereditary diffuse leukoencephalopathy with axonal spheroids caused by R782H mutation in CSF1R: case report. J Neurol Sci *318*, 115-118. 10.1016/j.jns.2012.03.012.

74. Nicholson, A.M., Baker, M.C., Finch, N.A., Rutherford, N.J., Wider, C., Graff-Radford, N.R., Nelson, P.T., Clark, H.B., Wszolek, Z.K., Dickson, D.W., et al. (2013). CSF1R mutations link

POLD and HDLS as a single disease entity. Neurology *80*, 1033-1040. 10.1212/WNL.0b013e31828726a7.

75. Kim, E.J., Shin, J.H., Lee, J.H., Kim, J.H., Na, D.L., Suh, Y.L., Hwang, S.J., Lee, J.H., Lee, Y.M., Shin, M.J., et al. (2015). Adult-onset leukoencephalopathy with axonal spheroids and pigmented glia linked CSF1R mutation: Report of four Korean cases. J Neurol Sci *349*, 232-238. 10.1016/j.jns.2014.12.021.

76. Hoffmann, S., Murrell, J., Harms, L., Miller, K., Meisel, A., Brosch, T., Scheel, M., Ghetti, B., Goebel, H.H., and Stenzel, W. (2014). Enlarging the nosological spectrum of hereditary diffuse leukoencephalopathy with axonal spheroids (HDLS). Brain Pathol *24*, 452-458. 10.1111/bpa.12120.

77. Inui, T., Kawarai, T., Fujita, K., Kawamura, K., Mitsui, T., Orlacchio, A., Kamada, M., Abe, T., Izumi, Y., and Kaji, R. (2013). A new CSF1R mutation presenting with an extensive white matter lesion mimicking primary progressive multiple sclerosis. J Neurol Sci *334*, 192-195. 10.1016/j.jns.2013.08.020.

78. Foulds, N., Pengelly, R.J., Hammans, S.R., Nicoll, J.A., Ellison, D.W., Ditchfield, A., Beck, S., and Ennis, S. (2015). Adult-Onset Leukoencephalopathy with Axonal Spheroids and Pigmented Glia Caused by a Novel R782G Mutation in CSF1R. Sci Rep *5*, 10042. 10.1038/srep10042.

79. Yokote, A., Ouma, S., Takahashi, K., Hara, F., Yoshida, K., and Tsuboi, Y. (2020). [A case of hereditary diffuse leukoencephalopathy with spheroids and pigmented glia presenting with long-term mild psychiatric symptoms]. Rinsho Shinkeigaku *60*, 420-424. 10.5692/clinicalneurol.60.cn-001370.

80. Lubomski, M., Buckland, M.E., Sy, J., Wei, H., Tan, I.Y.L., Kane, B., and Spring, P.J. (2018). Adult-onset leukoencephalopathy with neuroaxonal spheroids and pigmented glia mimicking systemic lupus erythematosus cerebral vasculitis. J Neurol Sci *395*, 25-28. 10.1016/j.jns.2018.09.025.

81. Kinoshita, M., Kondo, Y., Yoshida, K., Fukushima, K., Hoshi, K., Ishizawa, K., Araki, N., Yazawa, I., Washimi, Y., Saitoh, B., et al. (2014). Corpus callosum atrophy in patients with hereditary diffuse leukoencephalopathy with neuroaxonal spheroids: an MRI-based study. Intern Med *53*, 21-27. 10.2169/internalmedicine.53.0863.

82. Ahmed, R., Guerreiro, R., Rohrer, J.D., Guven, G., Rossor, M.N., Hardy, J., and Fox, N.C. (2013). A novel A781V mutation in the CSF1R gene causes hereditary diffuse leucoencephalopathy with axonal spheroids. J Neurol Sci *332*, 141-144. 10.1016/j.jns.2013.06.007.

83. Prieto-Morin, C., Ayrignac, X., Ellie, E., Tournier-Lasserve, E., and Labauge, P. (2016). CSF1R-related leukoencephalopathy mimicking primary progressive multiple sclerosis. J Neurol *263*, 1864-1865. 10.1007/s00415-016-8197-x.

84. Delaney, C., Farrell, M., Doherty, C.P., Brennan, K., O'Keeffe, E., Greene, C., Byrne, K., Kelly, E., Birmingham, N., Hickey, P., et al. (2021). Attenuated CSF-1R signalling drives cerebrovascular pathology. EMBO Mol Med *13*, e12889. 10.15252/emmm.202012889.

85. 于晓攀, 崔荣荣, 孙静, 刘潇, 李福锦, and 周畅 (2017). 遗传性弥漫性白质脑病合并轴索球样变的临床和影像学特点(附 1 例报告). 临床神经病学杂志 *30*, 464-467.

86. Robinson, J.L., Suh, E., Wood, E.M., Lee, E.B., Coslett, H.B., Raible, K., Lee, V.M., Trojanowski, J.Q., and Van Deerlin, V.M. (2015). Common neuropathological features underlie distinct clinical presentations in three siblings with hereditary diffuse leukoencephalopathy with

spheroids caused by CSF1R p.Arg782His. Acta Neuropathol Commun *3*, 42. 10.1186/s40478-015-0219-x.
87. Stoiloudis, P., Parissis, D., Smyrni, N., Stardeli, T., Afrantou, T., Konstantinopoulou, E., Grigoriadis, N., and Ioannidis, P. (2021). Hereditary diffuse leukoencephalopathy with spheroids mimicking primary progressive aphasia: report of a Greek case. Neurol Sci *42*, 3431-3433. 10.1007/s10072-021-05257-4.
88. Shu, Y., Long, L., Liao, S., Yang, J., Li, J., Qiu, W., Yang, Y., Bao, J., Wu, A., Hu, X., and Lu, Z. (2016). Involvement of the optic nerve in mutated CSF1R-induced hereditary diffuse leukoencephalopathy with axonal spheroids. BMC Neurol *16*, 171. 10.1186/s12883-016-0694-0.
89. Bonvegna, S., Straccia, G., Golfre Andreasi, N., Elia, A.E., Marucci, G., Di Bella, D., Cilia, R., and Eleopra, R. (2020). Parkinsonism and Nigrostriatal Damage Secondary to CSF1R-Related Primary Microgliopathy. Mov Disord *35*, 2360-2362. 10.1002/mds.28290.
90. La Piana, R., Webber, A., Guiot, M.C., Del Pilar Cortes, M., and Brais, B. (2014). A novel mutation in the CSF1R gene causes a variable leukoencephalopathy with spheroids. Neurogenetics *15*, 289-294. 10.1007/s10048-014-0413-1.
91. Ueda, S., Yamashita, H., Hikiami, R., Sawamoto, N., Yoshida, K., and Takahashi, R. (2015). A novel A792D mutation in the CSF1R gene causes hereditary diffuse leukoencephalopathy with axonal spheroids characterized by slow progression. eNeurologicalSci *1*, 7-9. 10.1016/j.ensci.2015.07.001.
92. Zur-Wyrozumska, K., Kaczmarska, P., and Mensah-Glanowska, P. (2021). Adult-onset leukoencephalopathy with axonal spheroids and pigmented glia associated with an A792D mutation in the CSF1R gene in a Polish patient. Neurol Neurochir Pol *55*, 322-324. 10.5603/PJNNS.a2021.0012.
93. Kondo, Y., Kinoshita, M., Fukushima, K., Yoshida, K., and Ikeda, S. (2013). Early involvement of the corpus callosum in a patient with hereditary diffuse leukoencephalopathy with spheroids carrying the de novo K793T mutation of CSF1R. Intern Med *52*, 503-506. 10.2169/internalmedicine.52.8879.
94. Chen, J., Luo, S., Li, N., Li, H., Han, J., and Ling, L. (2020). A Novel Missense Mutation of the CSF1R Gene Causes Incurable CSF1R-Related Leukoencephalopathy: Case Report and Review of Literature. Int J Gen Med *13*, 1613-1620. 10.2147/IJGM.S286421.
95. Coomans, C., Sieben, A., Lammens, M., Ceuterick-de Groote, C., Vandenbroecke, C., Goethals, I., Van Melkebeke, D., and Hemelsoet, D. (2018). Early-onset dementia, leukoencephalopathy and brain calcifications: a clinical, imaging and pathological comparison of ALSP and PLOSL/Nasu Hakola disease. Acta Neurol Belg *118*, 607-615. 10.1007/s13760-018-1023-8.
96. Terasawa, Y., Osaki, Y., Kawarai, T., Sugimoto, T., Orlacchio, A., Abe, T., Izumi, Y., and Kaji, R. (2013). Increasing and persistent DWI changes in a patient with hereditary diffuse leukoencephalopathy with spheroids. J Neurol Sci *335*, 213-215. 10.1016/j.jns.2013.08.027.
97. Garg, D., Vainganka r, A., Gupta, A., Rajan, R., Garg, A., Agarwal, A., Mustafa, F., Radhakrishnan, D.M., Pandit, A.K., Vishnu, V.Y., et al. (2025). CSF1R-related adult-onset leukoencephalopathy with axonal spheroids: A case series of four Asian Indian patients. J Mov Disord. 10.14802/jmd.25004.
98. Mochel, F., Delorme, C., Czernecki, V., Froger, J., Cormier, F., Ellie, E., Fegueux, N., Lehericy, S., Lumbroso, S., Schiffmann, R., et al. (2019). Haematopoietic stem cell transplantation in

CSF1R-related adult-onset leukoencephalopathy with axonal spheroids and pigmented glia. J Neurol Neurosurg Psychiatry *90*, 1375-1376. 10.1136/jnnp-2019-320701.

99. Tamhankar, P.M., Zhu, B., Tamhankar, V.P., Mithbawkar, S., Seabra, L., Livingston, J.H., Ikeuchi, T., and Crow, Y.J. (2020). A Novel Hypomorphic CSF1R Gene Mutation in the Biallelic State Leading to Fatal Childhood Neurodegeneration. Neuropediatrics *51*, 302-306. 10.1055/s-0040-1702161.

100. Daghagh, H., Rahbar Kafshboran, H., Daneshmandpour, Y., Nasiri Aghdam, M., Talebian, S., Nouri Nojadeh, J., Hamzeiy, H., Biskup, S., and Sakhinia, E. (2023). Homozygous mutation in CSF1R causes brain abnormalities, neurodegeneration, and dysosteosclerosis (BANDDOS). Bioimpacts *13*, 183-190. 10.34172/bi.2022.23528.

101. Misirocchi, F., Zilioli, A., Benussi, A., Capellari, S., Mutti, C., Florindo, I., Spallazzi, M., and Parrino, L. (2023). A Novel CSF1R Mutation Mimicking Frontotemporal Dementia: A Glimpse into a Microgliopathy. Can J Neurol Sci *50*, 642-644. 10.1017/cjn.2022.265.

102. Hayer, S.N., Schelling, Y., Hoeflinger, P., Hauser, S., and Schols, L. (2018). Generation of an induced pluripotent stem cell line from a patient with adult-onset leukoencephalopathy with axonal spheroids and pigmented glia (ALSP): HIHCNi003-A. Stem Cell Res *30*, 206-209. 10.1016/j.scr.2018.06.011.

103. Levin, J., Tiedt, S., Arzberger, T., Biskup, S., Schuberth, M., Stenglein-Krapf, G., Kreth, F.W., Hogen, T., la Fougere, C., Linn, J., et al. (2014). Diffuse leukoencephalopathy with spheroids: biopsy findings and a novel mutation. Clin Neurol Neurosurg *122*, 113-115. 10.1016/j.clineuro.2014.04.022.

104. Kraya, T., Quandt, D., Pfirrmann, T., Kindermann, A., Lampe, L., Schroeter, M.L., Kohlhase, J., Stoevesandt, D., Hoffmann, K., and Villavicencio-Lorini, P. (2019). Functional characterization of a novel CSF1R mutation causing hereditary diffuse leukoencephalopathy with spheroids. Mol Genet Genomic Med *7*, e00595. 10.1002/mgg3.595.

105. Blume, J., and Weissert, R. (2017). Suspected Perinatal Depression Revealed to be Hereditary Diffuse Leukoencephalopathy with Spheroids. J Mov Disord *10*, 59-61. 10.14802/jmd.16050.

106. Di Donato, I., Stabile, C., Bianchi, S., Taglia, I., Mignarri, A., Salvatore, S., Giorgio, E., Brusco, A., Simone, I., Dotti, M.T., and Federico, A. (2015). A Novel CSF1R Mutation in a Patient with Clinical and Neuroradiological Features of Hereditary Diffuse Leukoencephalopathy with Axonal Spheroids. J Alzheimers Dis *47*, 319-322. 10.3233/JAD-150097.

107. Kim, S.I., Jeon, B., Bae, J., Won, J.K., Kim, H.J., Yim, J., Kim, Y.J., and Park, S.H. (2019). An Autopsy Proven Case of CSF1R-mutant Adult-onset Leukoencephalopathy with Axonal Spheroids and Pigmented Glia (ALSP) with Premature Ovarian Failure. Exp Neurobiol *28*, 119-129. 10.5607/en.2019.28.1.119.

108. Gore, E., Manley, A., Dees, D., Appleby, B.S., and Lerner, A.J. (2016). A young-onset frontal dementia with dramatic calcifications due to a novel CSF1R mutation. Neurocase *22*, 257-262. 10.1080/13554794.2016.1175635.

109. Wang, M., and Zhang, X. (2019). A novel CSF-1R mutation in a family with hereditary diffuse leukoencephalopathy with axonal spheroids misdiagnosed as hydrocephalus. Neurogenetics *20*, 155-160. 10.1007/s10048-019-00579-0.

110. Granberg, T., Hashim, F., Andersen, O., Sundal, C., and Karrenbauer, V.D. (2016). Hereditary diffuse leukoencephalopathy with spheroids - a volumetric and radiological comparison with multiple sclerosis patients and healthy controls. Eur J Neurol *23*, 817-822. 10.1111/ene.12948.

111. Sundal, C., Baker, M., Karrenbauer, V., Gustavsen, M., Bedri, S., Glaser, A., Myhr, K.M., Haugarvoll, K., Zetterberg, H., Harbo, H., et al. (2015). Hereditary diffuse leukoencephalopathy with spheroids with phenotype of primary progressive multiple sclerosis. Eur J Neurol *22*, 328-333. 10.1111/ene.12572.
112. Cheng, X., Shen, W., Zou, H., Shen, L., Gu, X., Huang, D., Sun, Y., Wang, B., Tian, Q., and Xu, J. (2015). [Analysis of CSF1R gene mutation in a Chinese family with hereditary diffuse leukoencephalopathy with neuroaxonal spheroids]. Zhonghua Yi Xue Yi Chuan Xue Za Zhi *32*, 208-212. 10.3760/cma.j.issn.1003-9406.2015.02.012.
113. Oboudiyat, C., Bigio, E.H., Bonakdarpour, B., Baker, M.C., Rademakers, R., Weintraub, S., and Mesulam, M.M. (2015). Diffuse leukoencephalopathy with spheroids presenting as primary progressive aphasia. Neurology *85*, 652-653. 10.1212/WNL.0000000000001851.
114. Tipton, P.W., Stanley, E.R., Chitu, V., and Wszolek, Z.K. (2021). Is Pre-Symptomatic Immunosuppression Protective in CSF1R-Related Leukoencephalopathy? Mov Disord *36*, 852-856. 10.1002/mds.28515.
115. Sundal, C., Van Gerpen, J.A., Nicholson, A.M., Wider, C., Shuster, E.A., Aasly, J., Spina, S., Ghetti, B., Roeber, S., Garbern, J., et al. (2012). MRI characteristics and scoring in HDLS due to CSF1R gene mutations. Neurology *79*, 566-574. 10.1212/WNL.0b013e318263575a.
116. Saitoh, B.Y., Yamasaki, R., Hiwatashi, A., Matsushita, T., Hayashi, S., Mitsunaga, Y., Maeda, Y., Isobe, N., Yoshida, K., Ikeda, S.I., and Kira, J.I. (2019). Discriminative clinical and neuroimaging features of motor-predominant hereditary diffuse leukoencephalopathy with axonal spheroids and primary progressive multiple sclerosis: A preliminary cross-sectional study. Mult Scler Relat Disord *31*, 22-31. 10.1016/j.msard.2019.03.008.
117. Kunii, M., Doi, H., Ishii, Y., Ohba, C., Tanaka, K., Tada, M., Fukai, R., Hashiguchi, S., Kishida, H., Ueda, N., et al. (2018). Genetic analysis of adult leukoencephalopathy patients using a custom-designed gene panel. Clin Genet *94*, 232-238. 10.1111/cge.13371.
118. Kim, E.J., Kim, Y.E., Jang, J.H., Cho, E.H., Na, D.L., Seo, S.W., Jung, N.Y., Jeong, J.H., Kwon, J.C., Park, K.H., et al. (2018). Analysis of frontotemporal dementia, amyotrophic lateral sclerosis, and other dementia-related genes in 107 Korean patients with frontotemporal dementia. Neurobiol Aging *72*, 186 e181-186 e187. 10.1016/j.neurobiolaging.2018.06.031.
119. Shi, T., Li, J., Tan, C., and Chen, J. (2019). Diagnosis of hereditary diffuse leukoencephalopathy with neuroaxonal spheroids based on next-generation sequencing in a family: Case report and literature review. Medicine (Baltimore) *98*, e15802. 10.1097/MD.0000000000015802.
120. Cochran, J.N., McKinley, E.C., Cochran, M., Amaral, M.D., Moyers, B.A., Lasseigne, B.N., Gray, D.E., Lawlor, J.M.J., Prokop, J.W., Geier, E.G., et al. (2019). Genome sequencing for early-onset or atypical dementia: high diagnostic yield and frequent observation of multiple contributory alleles. Cold Spring Harb Mol Case Stud *5*. 10.1101/mcs.a003491.
121. Kortvelyessy, P., Krageloh-Mann, I., Mawrin, C., Heinze, H.J., Bittner, D., Wieland, I., Zenker, M., and Nestor, P. (2015). Hereditary diffuse leukoencephalopathy with spheroids (HDLS) with a novel CSF1R mutation and spinal cord involvement. J Neurol Sci *358*, 515-517. 10.1016/j.jns.2015.09.370.
122. Chen, L., Xu, H., and Lu, Z. (2025). Late-onset CSF1R-related Disorder: A Case Report. Cogn Behav Neurol *38*, 16-20. 10.1097/WNN.0000000000000385.
123. Wu, X., Sun, C., Wang, X., Liu, Y., Wu, W., and Jia, G. (2022). Identification of a de novo splicing mutation in the CSF1R gene in a Chinese patient with hereditary diffuse

leukoencephalopathy with spheroids. Neurol Sci *43*, 3265-3272. 10.1007/s10072-021-05755-5.
124. Yang, X., Huang, P., Tan, Y., and Xiao, Q. (2019). A Novel Splicing Mutation in the CSF1R Gene in a Family With Hereditary Diffuse Leukoencephalopathy With Axonal Spheroids. Front Genet *10*, 491. 10.3389/fgene.2019.00491.
125. Shixing, X., Wei, W., Xueyan, H., and Wei, T. (2022). Pathogenicity analysis and a novel case report of intronic mutations in CSF1R gene. Neurocase *28*, 251-257. 10.1080/13554794.2022.2071625.
126. Jiang, J., Li, W., Wang, X., Du, Z., Chen, J., Liu, Y., Li, W., Lu, Z., Wang, Y., and Xu, J. (2022). Two Novel Intronic Mutations in the CSF1R Gene in Two Families With CSF1R-Microglial Encephalopathy. Front Cell Dev Biol *10*, 902067. 10.3389/fcell.2022.902067.
127. Beerepoot, S., Verbeke, J., Plantinga, M., Nierkens, S., Pouwels, P.J.W., Wolf, N.I., Simons, C., and van der Knaap, M.S. (2024). Leukoencephalopathy with calcifications, developmental brain abnormalities and skeletal dysplasia due to homozygosity for a hypomorphic CSF1R variant: A report of three siblings. Am J Med Genet A *194*, e63800. 10.1002/ajmg.a.63800.
128. Leng, C., Lu, L., Wang, G., Zhang, Y., Xu, Y., Lin, X., Shen, N., Xu, X., Qun, S., Sun, M., and Ge, W. (2019). A novel dominant-negative mutation of the CSF1R gene causes adult-onset leukoencephalopathy with axonal spheroids and pigmented glia. Am J Transl Res *11*, 6093-6101.
129. Saitoh, B.Y., Yoshida, K., Hayashi, S., Yamasaki, R., Sato, S., Kamada, T., Suzuki, S.O., Murai, H., Iwaki, T., Ikeda, S.I., and Kira, J.I. (2013). Sporadic hereditary diffuse leukoencephalopathy with axonal spheroids showing numerous lesions with restricted diffusivity caused by a novel splice site mutation in the CSF1R gene. Clinical and Experimental Neuroimmunology *4*, 76-81. 10.1111/cen3.12076.
130. Lee, D., Yun, J.Y., Jeong, J.H., Yoshida, K., Nagasaki, S., and Ahn, T.B. (2015). Clinical evolution, neuroimaging, and volumetric analysis of a patient with a CSF1R mutation who presented with progressive nonfluent aphasia. Parkinsonism Relat Disord *21*, 817-820. 10.1016/j.parkreldis.2015.04.010.
131. Kawakami, I., Iseki, E., Kasanuki, K., Minegishi, M., Sato, K., Hino, H., Shibuya, K., Fujisawa, K., Higashi, S., Akiyama, H., et al. (2016). A family with hereditary diffuse leukoencephalopathy with spheroids caused by a novel c.2442+2T>C mutation in the CSF1R gene. J Neurol Sci *367*, 349-355. 10.1016/j.jns.2016.06.013.
132. Kindis, E., Simsek-Kiper, P.O., Kosukcu, C., Taskiran, E.Z., Gocmen, R., Utine, E., Haliloglu, G., Boduroglu, K., and Alikasifoglu, M. (2021). Further expanding the mutational spectrum of brain abnormalities, neurodegeneration, and dysosteosclerosis: A rare disorder with neurologic regression and skeletal features. Am J Med Genet A *185*, 1888-1896. 10.1002/ajmg.a.62179.
